# Correction: Pharmacodynamic Effects of Canagliflozin, a Sodium Glucose Co-Transporter 2 Inhibitor, from a Randomized Study in Patients with Type 2 Diabetes

**DOI:** 10.1371/journal.pone.0110069

**Published:** 2014-09-30

**Authors:** The PLOS ONE Staff

## Notice of Republication

This article was republished on September 15, 2014, to correct typesetting errors involving missing minus symbols throughout the text and tables. The publisher apologizes for these errors. Please download this article again to view the article. The originally published, uncorrected article and the republished, corrected article are provided here for reference.

## Supporting Information

File S1 **Originally published, uncorrected article**
PDF

File S2 **Republished, corrected article**
PDF
