# Supplementary material for: Pharmacodynamic Effects of Canagliflozin, a Sodium Glucose Co-Transporter 2 Inhibitor, from a Randomized Study in Patients with Type 2 Diabetes
Source: PLoS One. 2014 Aug 28;9(8):e105638. doi: 10.1371/journal.pone.0105638 (PMC4148334; doi:10.1371/journal.pone.0105638)
Supplement: Protocol S1 — (PDF) [file pone.0105638.s002.pdf]

Johnson & Johnson Pharmaceutical Research & Development*

Clinical Protocol

A DOUBLE-BLIND, RANDOMIZED, PLACEBO-CONTROLLED STUDY TO EVALUATE THE SAFETY, TOLERABILITY, PHARMACOKINETICS AND PHARMACODYNAMICS OF SINGLE AND MULTIPLE ASCENDING ORAL DOSES OF JNJ-28431754 IN TYPE 2 DIABETES MELLITUS PATIENTS

Protocol 28431754NAP1002; Phase I

JNJ-28431754

Amendment INT-4

* Johnson & Johnson Pharmaceutical Research & Development (J&JPRD) is a global organization that operates through different legal entities in various countries. Therefore, the legal entity acting as the sponsor for studies of Johnson & Johnson Pharmaceutical Research & Development may vary, such as, but not limited to Johnson & Johnson Pharmaceutical Research & Development L.L.C. or Janssen-Cilag International N.V. The term "sponsor" is used throughout the protocol to represent these various legal entities; the sponsor is identified on the Contact Information page that accompanies the protocol.

This study will be conducted under Food & Drug Administration IND regulations (CFR Part 312).

Issue/Report Date: 26 SEPTEMBER 2007
Prepared by: Johnson & Johnson Pharmaceutical Research & Development, L.L.C. Division of Janssen-Cilag Ltd.
Department: Drug Development
Document No.: EDMS-PSDB-6657018:6.0

Confidentiality Statement

The information in this document contains trade secrets and commercial information that are privileged or confidential and may not be disclosed unless such disclosure is required by applicable law or regulations. In any event, persons to whom the information is disclosed must be informed that the information is *privileged* or *confidential* and may not be further disclosed by them. These restrictions on disclosure will apply equally to *all* future information supplied to you that is indicated as *privileged* or *confidential*.

INVESTIGATOR AGREEMENT

I have read this protocol and agree that it contains all necessary details for carrying out this study. I will conduct the study as outlined herein and will complete the study within the time designated.

I will provide copies of the protocol and all pertinent information to all individuals responsible to me who assist in the conduct of this study. I will discuss this material with them to ensure that they are fully informed regarding the study drug and the conduct of the study.

Coordinating Investigator (where required):

Name (typed or printed): ____________________

Institution and Address: ____________________

____

____

____

Signature: ____________________ Date: ____________________
(Day Month Year)

Principal (Site) Investigator:

Name (typed or printed): ____________________

Institution and Address: ____________________

____

____

____

Signature: ____________________ Date: ____________________
(Day Month Year)

Sponsor's Responsible Medical Officer:

Name (typed or printed): Sue Sha

Institution: Johnson & Johnson Pharmaceutical Research & Development, L.L.C.
Division of Janssen-Cilag Ltd.

Signature: [signature] Date: 24 Sep. 2007
(Day Month Year)

* If the address or telephone number of the investigator changes during the course of the study, written notification will be provided by the investigator to the sponsor, and a protocol amendment will not be required.

TABLE OF CONTENTS

PROTOCOL AMENDMENTS 6

SYNOPSIS 14

TIME AND EVENTS SCHEDULE 21

ABBREVIATIONS 24

1. INTRODUCTION 27
1.1. Background 28
1.1.1. Pre-clinical Data 28
1.2. Overall Rationale for the Study 46

2. OBJECTIVES 46

3. OVERVIEW OF STUDY DESIGN 47
3.1. Study Design 47
3.2. Study Design Rationale 50
3.2.1. Rationale for DNA Collection 55
3.3. Subject Safety and Stopping Criteria 56

4. STUDY POPULATION 57
4.1. General Considerations 57
4.2. Inclusion Criteria 57
4.3. Exclusion Criteria 58
4.4. Prohibitions and Restrictions 61

5. RANDOMIZATION AND BLINDING 62
5.1. Overview 62
5.2. Procedures 62

6. DOSAGE AND ADMINISTRATION 64

7. COMPLIANCE 65

8. CONCOMITANT THERAPY 65

9. STUDY EVALUATIONS 65
9.1. Study Procedures 65
9.1.1. Overview 66
9.1.2. Screening Phase 67
9.1.3. Predosing Phase 68
9.1.4. Double-Blind Dosing Phase 72
9.1.5. Outpatient Phase 75
9.1.6. Posttreatment Phase (Follow-Up) 76
9.2. Pharmacokinetic Evaluations 76
9.2.1. Sample Collection and Handling 76
9.2.1.1. Plasma: 76
9.2.1.2. Urine 77
9.2.2. Analytical Procedures 78
9.2.3. Pharmacokinetic Parameters 78
9.2.3.1. Plasma 78
9.2.3.2. Urine 80
9.3. Pharmacodynamic Evaluations 80

TABLE OF CONTENTS (CONTINUED)

9.3.1. Primary 80
9.3.2. Secondary: 80
9.4. Pharmacogenomics 82
9.4.1. Analyses Related to the Trial (Part A) 82
9.4.2. DNA Storage for Future Analyses (Part B) 83
9.5. Safety Evaluations 84
9.5.1. Adverse Events 84
9.5.2. Clinical Laboratory 84
9.5.3. Cardiac Monitoring 88
9.5.4. Vital Signs 89
9.5.5. Physical Examination 90
9.5.6. Monitoring Blood Glucose Levels 90
9.5.7. Monitoring for Skin Reactions 91

10. SUBJECT COMPLETION/WITHDRAWAL 91
10.1. Completion 91
10.2. Discontinuation of Treatment 91
10.3. Withdrawal From the Study 91

11. STATISTICAL METHODS 93
11.1. Sample Size Determination 93
11.2. Pharmacokinetics 93
11.3. Pharmacodynamic Analyses 93
11.4. Safety Analyses 94
11.5. Interim Analyses 96

12. ADVERSE EVENT REPORTING 97
12.1. Definitions 97
12.1.1. Adverse Event Definitions and Classifications 97
12.1.2. Attribution Definitions 98
12.2. Procedures 99
12.2.1. All Adverse Events 99
12.2.2. Serious Adverse Events 100
12.2.3. Pregnancies 101
12.3. Contacting Sponsor Regarding Safety 101

13. STUDY DRUG INFORMATION 101
13.1. Physical Description of Study Drug(s) 101
13.2. Packaging 101
13.3. Labeling 102
13.4. Preparation and Handling 102
13.5. Drug Accountability 102

14. STUDY-SPECIFIC MATERIALS 103

15. ETHICAL ASPECTS 103
15.1. Study-Specific Design Considerations 103
15.2. Regulatory Ethics Compliance 106
15.2.1. Investigator Responsibilities 106
15.2.2. Independent Ethics Committee or Institutional Review Board (IEC/IRB) 106
15.2.3. Informed Consent 108
15.2.4. Privacy of Personal Data 109

TABLE OF CONTENTS (CONTINUED)

16. ADMINISTRATIVE REQUIREMENTS 110
16.1. Protocol Modifications 110
16.2. Regulatory Documentation 111
16.2.1. Regulatory Approval/Notification 111
16.2.2. Required Prestudy Documentation 111
16.3. Subject Identification Register and Subject Screening Log 112
16.4. Case Report Form Completion 112
16.5. Data Quality Assurance 113
16.6. Record Retention 113
16.7. Monitoring 114
16.8. Study Completion/Termination 115
16.8.1. Study Completion 115
16.8.2. Study Termination 115
16.9. On-Site Audits 116
16.10. Use of Information and Publication 116

17. REFERENCES 118

ATTACHMENTS 120
Attachment 1: Pharmacokinetic Sample Collection and Handling 121
Attachment 2: Labeling Instructions for Pharmacokinetic Samples 123
Attachment 3: Shipment of Pharmacokinetic Samples 124
Attachment 4: Pharmacogenomic Sample Collection and Shipment Procedure 126
Attachment 5: Pharmacodynamic Sample Collection and Shipment Procedure 128
Attachment 6: Shipment of Pharmacodynamic Samples 130
Attachment 7: Grading and Treatment of Hypoglycemic Events 131
Attachment 8: Standard Meals 133
Attachment 9: Medications with Potential for CYP3A4 Inhibition and/or Induction Properties 134
Attachment 10: Concomitant Medications 135
Attachment 11: Visual Analog Scale Questionnaire for Appetite and Satiety 137
Attachment 12: Patient Reported Outcomes Questionnaire 138

LAST PAGE 138

PROTOCOL AMENDMENTS

Original Protocol issued 12 April 2007

Amendments are listed beginning with the most recent amendment.

Amendment INT-4 (26 Sep 2007)
This amendment is considered to be **substantial** based on the criteria set forth in Article 10(a) of Directive 2001/20EC of the European Parliament and the Council of the European Union

Applicable Section(s)	Text Changes (new text in **bold**; deleted text in ~~strikeout~~)	Description of Change / Rationale for Change
Synopsis; Time and Events Schedule; 9.1.3 Predosing; 9.1.4 Double-blind; 9.5.3 Cardiac Monitoring	Continuous Lead II ECG monitoring (telemetry) will be conducted from 30 minutes prior to dosing until ~~6~~ **8** hours post dosing on Days -1, 1, **6,** 8 and 15.	Plasma drug concentration data from early cohorts (30 and 100mg dose) indicates that peak plasma concentration is reached at 0.5 to 6 hours post dose (Tmax). Telemetry monitoring will be extended from 6 to 8 hours to cover the range of Tmax. In addition, telemetry monitoring on Day 6 will be added to provide additional cardiac safety assessment at steady state drug exposure.

Amendment INT-3 (10 Sep 2007)
This amendment is considered to be **substantial** based on the criteria set forth in Article 10(a) of Directive 2001/20EC of the European Parliament and the Council of the European Union

Applicable Section(s)	Text Changes (new text in **bold**; deleted text in ~~strikeout~~)	Description of Change / Rationale for Change
Synopsis; Time and Events Schedule; Abbreviations; 9.1.3 Predosing; 9.1.4 Double-blind; 9.1.5 Outpatient; 9.1.6 Post Treatment; 9.5.2 Clinical Laboratory	**Serum 1,25-dihydroxy Vitamin D, and parathyroid hormone (PTH) will be assessed on Days -2, -1, 5, 11, 17, 22 and at the Follow-up visit.**	Based on animal toxicology data, the study drug might affect bone metabolism and electrolyte balance in humans. In addition to the bone markers already defined in the protocol, these 2 additional blood tests will allow further evaluation of any potential effects on bone metabolism and electrolyte balance.
9.1.1 Overview; 15.1 Study Specific Design Considerations	The total volume of blood drawn for laboratory evaluations throughout this study is approximately **482** mL for each subject and **518** mL for those subjects participating in twice-daily dosing cohorts.	Total blood volume will increase by 7 mLs as a result of adding 1,25-dihydroxy Vitamin D and PTH.

PROTOCOL AMENDMENTS (CONTINUED)

Amendment INT-2 (29 Jun 2007)
This amendment is considered to be **substantial** based on the criteria set forth in Article 10(a) of Directive 2001/20EC of the European Parliament and the Council of the European Union

Applicable Section(s)	Text Changes (new text in **bold**; deleted text in ~~strikeout~~)	Description of Change / Rationale for Change
Synopsis; 3.1 Study Design; 5.2 Procedures	At least **10** ~~15~~ subjects are required to complete each treatment period prior to a decision to escalate to the next higher dose level. If fewer than **10** ~~15~~ subjects complete any cohort, a separate Data Review Committee (DRC) may be convened to review unblinded data and to provide a recommendation regarding dose escalation.	The original statement requiring 15 subjects in a cohort to complete, prior to a dose escalation decision, was based on a randomization block size of 20. With that block size, and a 16:4 ratio of treated:placebo subjects, there would be at least 1 placebo subject included in 15 or more completed subjects in a given cohort so that unblinding would not be necessary for a dose-escalation decision. However, because more than one study center will participate in this study, and not all subjects in a given cohort may be studied at the same center, a block size of 20 might allow one center to study a small number of subjects that did not include placebo subjects. Thus, reducing the randomization block size from 20 to 5, will allow each center to enroll at least 5 subjects that will include at least 1 placebo subject. As a result, reducing the block size to 5 will minimize a potential center effect for clinical safety assessments in each cohort, ie, this will avoid the possibility that for a given cohort, a small number of subjects that includes no placebo subjects are enrolled in a given site. With this change, dose escalation decisions must be based on a minimum of 8 completing subjects on treatment and 2 completing subjects on placebo.

PROTOCOL AMENDMENTS (CONTINUED)

Applicable Section(s)	Text Changes (new text in **bold**; deleted text in ~~strikeout~~)	Description of Change / Rationale for Change
Synopsis; 3.1 Study Design; 3.2 Study Design Rationale	The maximum predicted total plasma JNJ-28431754 exposure (AUC_{0-24h} at steady-state) will not exceed the NOAEL established in the 2-week GLP toxicology study in rats (AUC_{0-24h}:~ 80 µg.h/mL; ~~Cmax: ~ 6 µg/mL~~), **based on the incidence of hyperostosis (minimal to mild changes in distal end of femur & proximal end tibia) at 150 mg/kg,** without an amendment to this protocol.	The original statement in this protocol regarding the drug exposure limit (specifying both Cmax and AUC at steady-state) was technically imprecise. In the first Phase I single dose study in healthy subjects (Study 28431754 NAP1001), the average Cmax value at the 600 mg dose was 5.9 ug/mL (slightly below the average Cmax at the NOAEL in the 14-day rat toxicology study). Due to normal, inter-subject PK variances around the mean, some subjects in that first study had Cmax values slightly higher than 6 ug/mL. In the current multiple-dose study, it is expected that some subjects receiving a 600 mg dose, the planned maximum dose, will also have steady-state Cmax values that slightly exceed 6 ug/mL, due to normal inter-subject PK variability. It should be noted that based on the PK data from the first single dose study (Study 28431754 NAP1001), the projected mean Cmax value at steady-state is not expected to be meaningfully different from the mean Cmax value following single dose administration.<br><br>Therefore, in this amended protocol, only the mean AUC value (~ 80 ug.h/mL) at the NOAEL of the 14-day rat toxicology study will be specified as the exposure limit. That NOAEL in the 14-day rat toxicology study was based on the incidence of hyperostosis (minimal to mild changes in distal end of femur & proximal end tibia) at the dose of 150 mg/kg/day. Although it is not clear that the hyperostosis observed in the 2-week rat toxicology study is Cmax or AUC related, Cmax values in some subjects that slightly exceed the NOAEL Cmax value during a 14-day short treatment period in the current study are not considered a clinical safety concern. In addition, biomarkers for bone turnover are being closely monitored in all cohorts of this study.

PROTOCOL AMENDMENTS (CONTINUED)

Applicable Section(s)	Text Changes (new text in **bold**; deleted text in ~~strikeout~~)	Description of Change / Rationale for Change
Synopsis; 3.1 Study Design; 3.2 Study Design Rationale	Although subjects of Asian ethnicity have not been explicitly enlisted for the current protocol, based on the likely study demographics, relatively few Asian subjects are likely to be enrolled in each cohort. Since the incidence of T2DM is rapidly increasing in Asia, this new drug may be developed for use in Asian countries. To support potentially larger and longer clinical trials in Asian diabetics, an additional cohort will be studied that is comprised exclusively of Asian diabetics to allow exploring safety and efficacy in this population. Based on preclinical data related to JNJ-28431754 metabolism and clearance mechanisms, the PK and metabolism profiles of JNJ-28431754 in Asian subjects are not expected to be markedly different from that in Caucasians or other ethnic groups.<br>The dose selected for this additional Asian cohort will be a dose that has been already evaluated in a prior cohort as a safe and potentially efficacious dose.	Adding one cohort of Asian subjects in this study allows for the exploration of drug effect (safety and efficacy) in Asian subjects. Results from this cohort will support further clinical development in this population. The dose level chosen for this Asian cohort will have been previously tested in an earlier cohort and considered to be well tolerated.
Synopsis, 9.1.3 Predosing Phase; 9.1.4 Double-Blind Dosing Phase; 9.3.2 Secondary	Administration of visual analogue scale (VAS) questionnaires to assess appetite and satiety at 5 minutes prior to breakfast, 30, 60, 90, 120, 150, 180, 210 and 240 minutes after **starting** breakfast, 5 minutes prior to lunch and immediately after lunch	Clarification that the post breakfast time points are to be measured from the start of breakfast.

PROTOCOL AMENDMENTS (CONTINUED)

Applicable Section(s)	Text Changes (new text in **bold**; deleted text in ~~strikeout~~)	Description of Change / Rationale for Change
9.1.4 Double-Blind dosing Phase; 9.5.6 Monitoring Blood Glucose Levels	Real time blood glucose concentrations will be determined prior to each standardized ~~meal~~ **breakfast** and 2 hours post each lunch using a glucose analyzer.	Clarification that glucose concentrations will be measured prior to breakfast and after lunch on Days -3 to 20.
Attachment 8	BMI-Adjustment<br><br>BMI 19 - <25 approx. 2100-2400 kcal<br>BMI 25 - <30 approx. 240**1**-2700 kcal<br>BMI 30 - <35 approx. 270**1**-2900 kcal<br><br>BMI > =35 approx. 290**1**-3200 kcal	BMI and kcal figures adjusted to eliminate overlap between levels

Amendment INT-1 (11 June 2007)
This amendment is considered to be **substantial** based on the criteria set forth in Article 10(a) of Directive 2001/20EC of the European Parliament and the Council of the European Union

Applicable Section(s)	Text Changes (new text in **bold**; deleted text in ~~strikeout~~)	Description of Change / Rationale for Change
Synopsis; 3.1 Study Design	Twice-daily dosing may also be evaluated in one or more of the cohorts or ~~one~~ additional cohort**s** of up to 20 subjects may be added. For any dose chosen for a possible twice-daily dosing regimen, the maximum predicted total plasma JNJ-28431754 exposure (AUC_{0-24} hr at steady-state) will not exceed the **NOAEL established in the 2-week GLP toxicology study in rats (AUC_{0-24}: ~ 80 μg.h/mL; Cmax: ~ 6 μg/mL) without an amendment to this protocol.** ~~steady-state JNJ-28431754 exposure already assessed as well-tolerated in a prior cohort~~.	Text changed to allow for the addition of more than one cohort and to maintain consistency within the protocol.

PROTOCOL AMENDMENTS (CONTINUED)

Applicable Section(s)	Text Changes (new text in **bold**; deleted text in ~~strikeout~~)	Description of Change / Rationale for Change
Synopsis; 3.1 Study Design; 4.2 Inclusion Criteria No. 2	Subjects must have been diagnosed with Type 2 diabetes at least 12 months prior to study start and will have been stably managed for at least 3 months prior to the Screening visit on metformin, or a sulfonylurea, or a meglitinide (e.g., repaglininide or nateglinide), or a DPP-IV inhibitor (e.g., sitagliptin or vildagliptin), or on an alpha-glucosidase inhibitor (e.g., Acarbose) **or a combination of two anti-diabetic medications**.	Text added to allow the inclusion of subjects on anti-diabetic combination therapy.
4.3 Exclusion criteria	Clinically significant abnormal values for hematology, coagulation, fasting clinical chemistry or urinalysis (elevated HbA1c, fasting blood glucose values or mild abnormality in lipid profiles are allowed for diabetic subjects) at screening or **Day -2** (Note: Subjects who have fasting triglyceride levels greater than 5.1 mmol/L are excluded)	Text changed to clarify the laboratory assessments will be performed on Day -2 not upon admission to the unit.
Synopsis; 9.3.2 Secondary Pharmacodynamic Evaluations	Plasma glucagon-like peptide-1 (GLP-1) levels (active and/or total on Days -1, 1 and ~~17~~ **16** at -0.5 predose and 0.5, 1, 1.5 and 2.0 postdose	Text changed to clarify an inconsistency.
4.3 Exclusion Criteria 13	Clinically significant abnormality in physical examination, vital signs, or 12-lead electrocardiogram (ECG) at screening or ~~admission~~ **Day -1**.	Text changed to clarify the ECG is performed on Day -1 not upon admission to the unit.
9.1.4 Double-blind Dosing Phase	~~"except item 1"~~	Text changed to clarify the inconsistencies regarding urine collection.
Synopsis; 9.3.2 Secondary Pharmacodynamic Evaluations	Renal threshold (Rt), which is defined as the plasma glucose concentration at which maximum urinary glucose reabsorption is achieved and above which glucose is excreted in the urine, will be determined on **Days –1, 1 and 16**.	Predefined time when the Rt will be determined has been added.
9.5.2 Clinical Laboratory	**GFR calculated using the MDRD equation: GFR = 175 × (standardized Scr) $^{-1.154}$ × (age) $^{-0.203}$ × 0.742 (if the subject is female) or × 1.212 (if the subject is black). GFR is expressed in milliliters per minute per 1.73 m^2 and race is either black or not.**	The MDRD GFR equation revised to include the component for females
9.5.4 Vital Signs	**Orthostatic vital signs will also be measured in triplicate.**	Text added to clarify procedure.

PROTOCOL AMENDMENTS (CONTINUED)

Applicable Section(s)	Text Changes (new text in **bold**; deleted text in ~~strikeout~~)	Description of Change / Rationale for Change
Attachment 8	**BMI-Adjustment**<br><br>**BMI 19-24**<br>**approx. 2100-2400 kcal**<br>**BMI 25-30**<br>**approx. 2400-2700 kcal**<br>**BMI 30-35**<br>**approx. 2600-2900 kcal**<br>**BMI > 35**<br>**approx. 2900-3200 kcal**<br><br>**The standard meals used for this study will be in accordance with the recommendations of the American Diabetes Association (American Diabetes Association: Evidence-based nutrition principles and recommendations for the treatment and prevention of diabetes and related complications which foresee 50-60% of calorie intake to come from carbohydrates, 15-20% from protein and 25-35% from fat.**<br><br>**− Breakfast (or dinner for BID dosing) within 10 minutes after dosing Approximately 30% of total calorie intake**<br><br>**− Lunch at 4.5 hours after dosing Approximately 35% of total calorie intake**<br>**− Dinner at 10.5 hours after dosing Approximately 35% of total calorie intake**	Text added to clarify meal composition and BMI adjustment.
9.1.3 Predosing Phase	Urinary drug abuse, serum pregnancy and breath alcohol test **(-2 day window allowed)**	The window will allow the drug screen, pregnancy test and alcohol test to be performed earlier ensuring the results are available prior to Day 1 dosing.
9.1.3 Predosing Phase	~~One aliquot will be stored at 70 C as a backup.~~	The site is to follow local laboratory requirements
9.2.1.2 Urine	Day 16:<br>Urine samples for analysis of JNJ-28431754 and its metabolites concentration will be collected at ~~predose~~,	Text deleted to clarify that a predose urine PK sample is not required on Day 16
9.5.2 Clinical Laboratory	**Drug/Alcohol Screen:**<br>**A drug and alcohol screen will be performed at screening and Day -2.**	This text was missing from Section 9.5.2

PROTOCOL AMENDMENTS (CONTINUED)

Applicable Section(s)	Text Changes (new text in **bold**; deleted text in ~~strikeout~~)	Description of Change / Rationale for Change
9.5.2 Clinical Laboratory	**Serum glucose** replaced plasma glucose	Correction to clinical laboratory panel
9.5.2 Clinical Laboratory	24-hour creatinine clearance (CLcr) on Day –1, 3, 5, 8, 11, 14, **16**, 17, 18 and **19**	Text changed to clarify creatinine clearance calculation days
9.5.2 Clinical Laboratory	HbA1c **(screening only)**	Text added to clarify HbA1c is required at screening only
References	**Reference No. 13 will be changed to " Polidori D. SIARaA: A Robust Algebraic Method for Determining Insulin Sensitivity and Glucose Absorption Rates from Oral Glucose Tolerance Tests or Mixed Meals. Internal Report. Johnson & Johnson Pharmaceutical Research and Development. December 12, 2006**<br><br>Reference No. 14 will be the previous No. 13<br><br>**Reference No. 15 will be changed to "Lesley A et al. Assessing kidney function - measured and estimated glomerular filtration rate. N Engl J Med 2006; 354:2473-83"**<br><br>References No 16-19 will be the previous 15-18 (for ECG references)<br><br>Reference No 20 will the previous No 19.<br>**Reference No. 21"Evidence-based nutrition principles and recommendations for the treatment and prevention of diabetes and related complications Diabetes Care 2002; 25:202-212"**	Corrections made to reference list
Attachment 1	samples will be frozen and stored upright immediately at ~~-18~~ **-20** °C or below.	PK sample storage conditions clarified
Attachment 3	**Marken** or World Courier	Additional courier added
9.1.1 Overview; 15.1 Study Specific Design Considerations	**475 mL/511 mL**	Blood volumes corrected

A DOUBLE-BLIND, RANDOMIZED, PLACEBO-CONTROLLED STUDY TO EVALUATE THE SAFETY, TOLERABILITY, PHARMACOKINETICS AND PHARMACODYNAMICS OF SINGLE AND MULTIPLE ASCENDING ORAL DOSES OF JNJ-28431754 IN TYPE 2 DIABETES MELLITUS SUBJECTS

SYNOPSIS

JNJ-28431754 is a potent, selective, and reversible inhibitor of the renal sodium glucose co-transporter (SGLT2). It is being investigated for the treatment of Type 2 diabetes mellitus (T2DM).

OBJECTIVES:

To evaluate the safety, tolerability, pharmacokinetics (PK) and pharmacodynamics (PD) of JNJ-28431754 after single and multiple ascending oral doses of JNJ-28431754 in subjects with Type 2 diabetes mellitus (T2DM).

OVERVIEW OF STUDY DESIGN:

This is a randomized, double-blind, placebo-controlled, single and multiple (14 days) ascending dose, parallel group study. Five cohorts of subjects with T2DM (20 subjects per cohort) will be studied. One dose level will be evaluated in each cohort.

Sixteen (16) subjects will be randomized to JNJ-28431754 and four (4) to matching placebo within each cohort. Doses planned for evaluation are 30, 100, 300 and 600 mg per day. An additional cohort of Asian subjects will also be evaluated at a dose level, which was previously tested in a prior cohort and considered to be well tolerated. Following initial screening (conducted within 35 days prior to Day -3 of the study), eligible subjects will be instructed to discontinue their previous antidiabetic medications for 16 days prior to dosing on Day 1. Blood glucose levels will be monitored daily during the 16-day washout period to ensure their blood glucose levels remain within a well-tolerated range. Eligible subjects will be admitted to the Clinical Research Unit (CRU) on Day – 3. On Days –2 and -1, all subjects will receive placebo once daily in a single-blind fashion (subjects blinded) and undergo baseline safety and PD assessments. Eligible subjects will then be randomized to a double-blind treatment with JNJ-28431754 or placebo. A single dose of study medication will be administered on Day 1, followed by periodic safety, PK and PD assessments for 48 hours. Daily dosing will resume on Day 3 and continue through Day 16. Each daily dose will be administered at approximately the same time each day (between 8 AM and 9 AM) followed by a standardized breakfast within 10 minutes after dosing. Standardized lunch and dinner will be provided at 4.5 and 10.5 hours post dose, respectively. Subjects will be discharged from the CRU on Day 20 (96 hours after last dosing) and return to the CRU for safety assessments and PK sample collections on the mornings of Days 21 to 22. Subjects will also return within 7-10 days following the Day 22 outpatient visit for a final Follow-up visit.

Subjects will resume their pre-study oral anti-diabetic medication regimen upon completion of the out-patient visit on Day 22 or potentially at any earlier time during the study if their confirmed fasting blood glucose levels rise above 15 mM (270 mg/dL) or postprandial glucose levels rise above 22.2 mM (400 mg/dL) following the lunch. Subjects who need to restart anti-diabetic medications during the study will stop receiving study medication but continue with study related safety assessments and PK samples (relative to predose time point only) for at least 3 days after their final dose. The Principal Investigator will determine the dose at which to reinitiate the subjects' prior anti-diabetic medications and any subsequent dose adjustments.

The selected dose level for each subsequent cohort defined in this protocol may be modified if indicated based on evaluation of the safety, tolerability, pharmacokinetics or pharmacodynamics of prior cohorts. Modifications of any next dose can be a de-escalation, a reduced escalation step, or an increased escalation step. Twice-daily dosing may also be evaluated in one or more of the cohorts or additional cohorts of up to 20 subjects may be added. For any dose chosen for a possible twice-daily dosing regimen, the maximum predicted total plasma JNJ-28431754 exposure ($AUC_{0\text{-}24h}$ at steady-state) will not exceed the NOAEL established in the 2-week GLP toxicology study in rats ($AUC_{0\text{-}24h}$: ~ 80 µg.h/mL) based on the incidence of hyperostosis (minimal to mild changes in distal end of femur & proximal end tibia) at 150 mg/kg, without an amendment to this protocol. Pharmacokinetic and pharmacodynamic sampling time points may be

SYNOPSIS (CONTINUED)

adjusted to accommodate a twice-daily dosing regimen, but the total blood volume per subject specified in this protocol will not be exceeded. For all cohorts, JNJ-28431754 dose levels that are predicted to produce steady state JNJ-28431754 exposures above the NOAEL in the 2-week GLP toxicology study in rats (AUC_{0-24h}: ~ 80 µg.h/mL) will not be exceeded without an amendment to this protocol.

Planned JNJ-28431754 Dose Levels					
**Cohort[1]**	**N**				
**1**	16+4[2]	30 mg or placebo			
**2**	16+4[2]		100 mg or placebo[3]		
**3**	16+4[2]			300 mg or placebo[3]	
**4**	16+4[2]				600 mg or placebo[3]
**5**	16+4[2]				30, 100 or 300 mg or placebo[4]

[1] Initiation of dosing for each subsequent cohort at a higher dose level will be at least 17 days following the initiation of dosing in the prior cohort.

[2] Sixteen on JNJ-28431754; four on placebo

[3] The actual dose (mg) level selected for each cohort may be modified based on evaluation of preliminary safety, pharmacokinetic and pharmacodynamic data from previous cohorts.

4 A fifth cohort will assess the safety, tolerability and effects of multiple doses of JNJ-28431754 on male and female Asian subjects at a dose level which was previously tested in an earlier cohort and considered to be well tolerated

Successive cohorts at a higher dose level will be started with at least a 17-day interval relative to the start of dosing in the preceding cohort, after review of adverse events, vital signs, electrocardiograms (ECGs) and laboratory tests of the preceding cohort collected up to and including Day 17 for preliminary safety data and up to Day 11 for PK data. Each subsequent escalated dose level shall be performed if, in the judgement of the Principal Investigator and the Sponsor's Safety Physician, the results of the safety analysis of the preceding dose level are satisfactory. At least 10 subjects are required to complete each treatment period prior to a decision to escalate to the next higher dose level. If fewer than 10 subjects complete any dose level, a separate Data Review Committee (DRC) may be convened to review unblinded data and to provide a recommendation regarding dose escalation.

STUDY POPULATION:

Five cohorts of 20 (total 100) male and post-menopausal or surgically sterilized female Type 2 diabetic subjects, age 25 to 65 years, body mass index (BMI) between 20.0 and 39.9 kg/m^2 inclusive, meeting all protocol inclusion/exclusion criteria will be enrolled in the study. Within each cohort at least 3 subjects will be female. No subject will participate in more than one cohort.

Female subjects must be of non-childbearing potential, i.e., either post-menopausal, or pre-menopausal with documented surgical sterilization. Post-menopausal is defined as either no menses for at least 18 months prior to study start, or as no menses for 6 to 18 months prior to the start of this study and plasma FSH ≥ 40 mlU/mL and estradiol ≤ 20 pg/mL in 3 separate measurements (at Screening, on Day -17, and on Day -2 (-2 day window allowed).

All subjects will have HbA1c levels at Screening within the range of 7.0 to 10.0% and will also have fasting blood glucose at Day –2 within the range: ≥7.8 mM and ≤15.0 mM (140-270 mg/dl). In addition, during the 16 day washout period prior to Day 1, subjects will monitor their blood glucose levels daily and no subject will continue in the study if, during the washout period, fasting blood glucose levels are confirmed to rise above 15 mM (270 mg/dL) or postprandial glucose levels are confirmed to rise above 22.2 mM (400 mg/dL) following lunch.

SYNOPSIS (CONTINUED)

Subjects must have been diagnosed with Type 2 diabetes at least 12 months prior to study start and will have been stably managed for at least 3 months prior to the Screening visit on metformin, or a sulfonylurea, or a meglitinide (e.g., repaglininide or nateglinide), or a DPP-IV inhibitor (e.g., sitagliptin or vildagliptin), or on an alpha-glucosidase inhibitor (e.g., Acarbose) or a combination of two anti-diabetic medications. Subjects will not have used any of the following anti-diabetic medications within 3 months of study start: exenatide, thiazolidinediones (e.g., rosiglitazone, or pioglitazone), or insulin.

Subjects with hypertension will be allowed in this study if their blood pressure has been successfully treated on a stable anti-hypertensive drug regimen for at least 3 months prior to study start with allowed anti-hypertensive medications (See Attachment 10), and there is no expectation that their anti-hypertensive medication regimen would change during the study. Subjects with dyslipidemias may be allowed in this study if they have been on a stable regimen of commonly prescribed dyslipidemic agents (e.g., statins, fibrates: See Attachment 10) for at least 3 months prior to this study, and their dyslipidemic drug regimen is not expected to change during this study. No subjects requiring concomitant medications for the continued treatment of obesity will be enrolled. The Principal Investigator and the Sponsor will review the inclusion of subjects on other medications (including non-prescription, herbal remedies or other over-the-counter agents) on a case-by-case basis. No alcohol consumption will be allowed from 72 hours prior to dosing until completion of the follow-up visit.

Eligible subjects for this study will return for an outpatient visit around Day -17, to be counseled by a registered dietician to follow a weight maintaining diet for approximately 2 weeks prior to admission and throughout the entire study except Days –3 to 20 when subjects will receive standardized meals (See Attachment 8). (The weight maintaining diet will follow standard recommendations of the American Diabetes Association, comprising ~55-60% carbohydrate, 10-20% protein, and ~30% fat, with total daily caloric intake individually adjusted for BMI). In addition, subjects will receive instruction in signs and symptoms of hypoglycemia, use of a portable home fingerstick blood glucose analyzer and reporting of results. During the washout phase and during the post-treatment phase (between discharge and prior to the Follow-up visit), subjects will record their fasting blood glucose levels and the postprandial blood glucose levels after lunch on a diary card which will be provided to them and report the results to the clinic weekly, or at any time values exceed prespecified limits. This information will be collected by the clinic and recorded in the eCRF.

DOSAGE AND ADMINISTRATION:

Compound:	JNJ-28431754
Strength:	5 and 50 mg/mL
Dosage form:	liquid, suspension of JNJ-28431754 in 0.5% hypromellose
Placebo:	0.5% hypromellose

PHARMACOKINETIC EVALUATIONS:

Day 1:

Plasma samples for measurement of JNJ-28431754 concentration will be collected at predose, 0.5, 1, 1.5, 2, 2.5, 3, 4, 6, 8, 10, 12, 24 and 48 hours postdose (total 14 samples).

Sample collection times for later cohorts may be modified as preliminary pharmacokinetic data becomes available during the study from initial cohorts. If a twice-daily dosing regimen is evaluated, 6 additional PK plasma samples may be collected at 10.5 (prior to dinner), 13, 14, 16, 19 and 22 hours after the morning dose. However, for all dose levels or dosing regimens, the total blood volume collected from each subject for all analyses (PK, PD, safety) will not exceed 550 mL over 8 weeks in this study.

Urine samples for measurement of JNJ-28431754 concentrations will be collected at predose, 0-2, 2-4.5 (prior to lunch), 4.5-7, 7-10.5 (prior to dinner), 10.5-13, 13-24 and 24-48 hours post dose.

Days 3 to 15:

Blood samples will be collected at pre-dose in the morning on Days 3, 5, 7, 9, 11, 13 and 15 to obtain trough JNJ-28431754 concentrations (total 7 samples)

Day 16:

Plasma samples for measurement of JNJ-28431754 concentration will be collected at predose, 0.5, 1, 1.5, 2, 2.5, 3, 4, 6, 8, 10, 12, 24, 48, 72, 96, 120 and 144 hours postdose (total 18 samples). If a twice-daily dosing regimen is evaluated, 6 additional PK plasma samples may be collected at 10.5 (prior to dinner), 13, 14, 16, 19 and 22 hours post the morning dose.

SYNOPSIS (CONTINUED)

Sample collection times for later cohorts may be modified as preliminary pharmacokinetic data becomes available during the study from initial cohorts, but the total blood volume collected from each subject for all analyses (PK, PD, safety) will not exceed 550 mL over 8 weeks in this study.

Plasma and urine samples will be stored for potential identification/analysis of JNJ-28431754 metabolites or concomitant medication pharmacokinetic analysis and will be reported separately.

Urine samples for analysis of JNJ-28431754 will be collected at predose, 0-2, 2-4.5 (prior to lunch), 4.5-7, 7-10.5 (prior to dinner), 10.5-13, 13-24, 24-48, 48-72 and 72-96 hours post dose.

Volumes will be recorded and aliquots taken after mixing for storage at -20°C and subsequent assay. Aliquots will also be taken for assay of glucose, electrolytes and creatinine (See below)

PHARMACODYNAMIC EVALUATIONS:

Primary:

1. Change from baseline for mean 24 hour plasma glucose concentration on Days 1 and 16, which is defined as the area under the plasma glucose concentration time curve over 0-24 hours, divided by 24 hours (Glucose AUC 0-24/24h). Glucose AUC 0-24/24h determined on Day –1 will be used as the baseline values

2. Change from baseline urine glucose excretion (UGE)

    a. Cumulative amount (grams of glucose) over each 24 hours on Days 1, 2, 8, 12, 16, 17, 18, 19; cumulative amount over 24 hours on Day –1 will be used as the baseline value

    b. UGE rate (grams of glucose per hour), defined as the UGE amount in each urine collection fraction, divided by the hour duration of each collection interval on Days 1, 2, 8, 12, 16, 17, 18, 19

Secondary:

1. Renal threshold (Rt), which is defined as the plasma glucose concentration at which maximum urinary glucose reabsorption is achieved and above which glucose is excreted in the urine, will be determined on Days -1, 1 and 16. The Rt is calculated as a function of time for each subject based on measured plasma glucose, urinary glucose excretion, and glomerular filtration rate (GFR) (See Section 9.3.2)
2. Change from baseline mean 24 hour insulin concentration on Days 1 and 16, which is defined as the area under the plasma insulin time concentration time curve at 0-24 hours divided by 24 hours (Insulin AUC 0-24/24h). Insulin AUC 0-24/24h determined on Day –1 will be used as the baseline value
3. Changes from baseline morning fasting plasma glucose (FPG) and insulin (FPI) on Days 1 and 17. FPG and FPI values determined on Day –1 will be used as the baseline values. FPG and FPI on Days –1, 1 and 16 will be calculated by averaging predose values at –30, -15 and 0 minutes. On Days 2 to 15 and 17 to 20, one FPG and FPI will be determined within 30 minutes prior to dosing on dosing days or prior to breakfast on non-dosing days.
4. Change from baseline plasma glucose and insulin excursions: after each breakfast, defined as the difference between the maximum glucose and insulin values observed during the 4-h post meal period minus the mean of the premeal measurements at –30, -15 and 0 min on Days 1 and 16. Glucose and insulin excursions on Day –1 will be used as the baseline value.
5. Glucose AUC 0-2 on Days 1, and 16; Glucose AUC 0-2 on Days –1 will be used as the baseline value.
6. Insulin sensitivity (See Section 9.3.2)
7. Beta-cell function (See Section 9.3.2)
8. Plasma glucagon-like peptide-1 (GLP-1) levels (active and/or total on Days -1, 1 and 16 at -0.5 predose and 0.5, 1, 1.5 and 2.0 postdose

SYNOPSIS (CONTINUED)

9. Assessment of Visual Analogue Scale (VAS) questionnaires to assess appetite and satiety at 5 minutes prior to breakfast, 30, 60, 90, 120, 150, 180, 210 and 240 minutes after starting breakfast, 5 minutes prior to lunch and immediately after lunch on Days –2, 3, 7, 11 and Day 15. The VAS questionnaire schedule may be modified if a twice-daily dosing regimen is tested.
10. Morning fasting body weight will be assessed at Screening and on Days -1 through 20 and at the Follow-up visit using a calibrated scale.

Blood samples for plasma glucose, insulin and C-peptide will be collected on Day –1 at T-0.5, T-0.25, T0, T0.5, T1, T1.25, T1.5, T2.0, T2.5, T3.0, T4.5 (prior to lunch), T5.0, T5.5, T6.0, T6.5, T7.0, T8.0, T9.0, T10.5 (prior to dinner), T11, T12.0, T12.5, T13.0, T14.0 T16.0, T19.0, T22.0, and T24 hours where T0 corresponds to the dosing time on dosing days, and on Day 1 and 16 at -0.5, -0.25, 0, 0.5, 1, 1.25, 1.5, 2.0, 2.5, 3.0, 4.5 (prior to lunch), 5.0, 5.5, 6.0, 6.5, 7.0, 8.0, 9.0, 10.5 (prior to dinner), 11, 12.0, 12.5, 13.0, 14.0 16.0, 19.0, 22.0, and 24 hours postdose.

Morning fasting plasma glucose, insulin and C-peptide concentrations will be obtained daily on Days 2 to 15 and 17 to 20 within 30 minutes prior to dosing on dosing days or prior to breakfast on non-dosing days.

Blood samples for glucagon-like peptide-1 (GLP-1; active and total) measurement will be collected on Days -1, 1 and 16 at -0.5 predose and 0.5, 1, 1.5 and 2.0 hours following the morning dose.

Collection times for blood samples in later cohorts may be modified as preliminary PD data become available during the study from initial cohorts, but the total blood volume collected from each subject for all analyses (PK, PD, safety) will not exceed 550 mL over 8 weeks in this study.

All urine will be collected, within specified intervals: on Day –1 at T0–T2, T2–T4.5 (prior to lunch), T4.5–T7, T7-10.5 (prior to dinner), T10.5-13, and T13-24 hours, where T0 corresponds to the dosing time on dosing days, on Day 1 at 0-2, 2-4.5 (prior to lunch), 4.5-7, 7-10.5 (prior to dinner), 10.5-13, 13-24 and 24-48 hours postdose; on Day 16 at 0-2, 2-4.5 (prior to lunch), 4.5-7, 7-10.5 (prior to dinner), 10.5-13, 13-24, 24-48, 48-72 and 72-96 hours after the last dose; and on Days 3 to 15 at 0-24 hours.

PHARMACOGENOMICS:

A pharmacogenomic blood sample (10 mL) will be collected from subjects who give separate written informed consent for this component of the study. This will allow for pharmacogenomic research, as necessary. Participation in this pharmacogenomic research is optional.

SAFETY EVALUATIONS:*

Physical examinations including thorough whole body skin examination will be performed at Screening and on Days –2, 15, 20 and at the follow up visit.

Vital signs (resting blood pressure and heart rate) and 12-lead ECGs will be performed at Screening, and at approximately T0, T2, T6 and T12 hours on Day –1 where T0 corresponds to the dosing time on dosing days. The vital signs and 12-lead ECGs will be performed at 0 (predose), 2, 6, 12, 24, 48, 72 and 96 hours post dose on Days 1 and 16, and at 2 hours postdose on Days 3 to 15 and once at the follow-up visit. At each specified times except at Screening and the follow-up visit, the vital signs and 12-lead ECG measurementwill be performed in triplicate, approximately 2 minutes apart. . The average of the triplicate measurements at each time point on Day –1 will serve each subject's time-matched baseline value for comparison to the corresponding parameters measured on Days 1 to 20. All morning vital signs and 12-lead ECGs will be performed prior to dosing on study drug administration days and prior to breakfast on non-dosing days.

Continuous Lead II ECG monitoring (telemetry) will be conducted from 30 minutes prior to dosing until 8 hours post dosing on Days -1, 1, 6, 8 and 15. These telemetry ECG data will not be recorded in the database and are for visual monitoring. Any abnormality detected by the device or the investigator will be printed out and retained as source data. Any clinically significant abnormalities will be recorded as adverse events.

* This section of the protocol has been revised. Please refer to the section of this document titled PROTOCOL AMENDMENTS (Amendment INT-4, 26 September 2007) for a detailed description of the specific changes.

SYNOPSIS (CONTINUED)

Orthostatic vital signs will be measured on Day –2 to 20 at 4 and 8 hours post dose.

Body temperature will be measured at Screening, on the morning of Days –2 to 20 and at the follow-up visit.

Height and weight will be measured at the Screening visit and will be utilized to calculate BMI and BSA. Weight will be measured at Screening, on Days –1 to 20 and at the follow-up visit on a calibrated scale. Weekly calibration records will be stored in study files.

Adverse events will be recorded during the entire study and will be specifically queried on each dosing day at predose, 3, 6 and 12 hours post dose, once on Days 2, 20, 21, 22 and at the follow up visit.

Fasting samples for hematology, clinical chemistry and urinalysis dipstick will be collected at Screening, on Days -2, 2, 3, 5, 8, 11, 14, 17, 18, 20, and 22 and at the follow up visit. Samples for measurement of TSH to assess thyroid function will be collected at Screening, and for coagulation including International normalized ratio (INR) and activated partial thromboplastin time (APTT) will be collected at Screening and Day 20.

For each urine collection interval, total urine volume will be recorded, and, samples will be taken for the following analyses:

-glucose

-creatinine

-electrolytes: sodium, chloride, potassium, phosphate, calcium and magnesium

-pH, specific gravity and osmolarity

-albumin

-N-acetyl-beta-glucosaminidase(NAG), and beta-2-microglobulin

All fluid intake, including water and all other beverages consumed at dosing and during meals, urine volume and frequency of urination over each 24-hour period will be recorded.

Urine levels of collagen cross-linked N-telopeptides (NTX) and urine deoxypyridinolines (DPD), serum bone specific alkaline phosphatase and serum osteocalcin (OC) will be assessed on Days –2, -1, 2, 3, 5, 8, 11, 14, 17, 18, 20, and 22 and at the follow up visit. Serum 1,25-dihydroxy Vitamin D, and parathyroid hormone (PTH) will be assessed on Days -2, -1, 5, 11, 17, 22 and at the Follow-up visit. The investigational staff will monitor the subjects for symptoms of hypoglycemia throughout the in-clinic residency. Should symptoms possibly indicative of hypoglycemia appear (e.g. altered mental status, dizziness/lightheadedness, diaphoresis, tachycardia), blood glucose will be immediately measured using a glucose analyzer and confirmed by a conventional venous blood sample glucose determination. Subjects will also measure their fasting (morning) and postprandial (post lunch) blood glucose levels daily throughout the study (during the prestudy washout, inpatient and follow-up phases).

Subjects will be domiciled continuously from Days -3 through Day 20 and instructed to avoid direct exposure to sunlight or artificial sources of intense light for at least 96 hours post the last dose to minimize the risk of potential photosensitivity. In addition, subjects will be closely monitored for potential skin reactions with a thorough examination of the whole body skin by a physician or his/her designee at Screening, on Days -1 through Day 20 at each dose level.

PATIENT REPORTED OUTCOMES EVALUATION:

A short questionnaire related to urination frequency will be administered at approximately the same time each morning on Days -2, 4, 7 and 14 to each subject (Attachment 12).

STATISTICAL METHODS:

PK: Pharmacokinetic analyses will be performed for all subjects receiving at least 1 dose of JNJ-28431754. Results will be summarized and descriptive statistics will be generated for each dose level.

PD: Mixed effect ANOVA modeling will be used to assess the treatment effects on 24-hour mean plasma glucose and urine glucose excretion (amount and rate). The estimated least-squares means and appropriate 95% confidence intervals for the difference of the mean pharmacodynamic parameters will be obtained for pharmacodynamic evaluations.

SYNOPSIS (CONTINUED)

Safety: All data will be fully listed. Unless otherwise noted, all the measures will be summarized with descriptive statistics for each dose level and all placebo observations grouped together. All statistical analyses will be considered exploratory and interpreted as such. No corrections will be made for multiple comparisons.

It is estimated that a sample size of 20 (16 receiving active JNJ-28431754 and 4 receiving placebo) subjects with T2DM should be sufficient to detect a 15% reduction in 24-hour mean AUC with 80% power, assuming a one-sided test and a coefficient of variation of 18 percent.

TIME AND EVENTS SCHEDULE*

	Screen[c]	D-17[b]	D-2	D-1	Days 1 & 16																														D2	D3	D4	D5	D6-7	D8	D9-10	D11	D12-13	D14	D15	D17	D18	D19	D20	D21	D22	Follow-up[e]
Time					-0.5	-0.25	0	0.25	0.5	1	1.25	1.5	2	2.5	3	4	4.5	5	5.5	6	6.5	7.0	8	9	10	10.5	11	12	12.5	13	14	16	19	22																		
Resident in clinic[d]			x------																																														------x			
Informed consents	x																																																			
Diet instruction		x																																																		
Medical history	x																																																			
Physical exam	x		x																																										x				x			x
Height	x																																																			
Weight[e]	x			x	x																														x	x	x	x	x	x	x	x	x	x	x	x	x	x	x			x
Temperature	x		x		x																														x	x	x	x	x	x	x	x	x	x	x	x	x	x	x			x
Serology[f]	x																																																			
Pregnancy[g]	x	x	x																																																	
Drug-alcohol screen[h]	x		x																																																	
Vital signs[i]	x			x			x						x							x								x							x	x	x	x	x	x	x	x	x	x	x	x	x	x	x			x
Orthostatic VS[j]			x													x							x												x	x	x	x	x	x	x	x	x	x	x	x	x	x	x			

* This section of the protocol has been revised. Please refer to the section of this document titled PROTOCOL AMENDMENTS (Amendment INT-4, 26 September 2007) for a detailed description of the specific changes.

TIME AND EVENTS SCHEDULE (CONTINUED)

	Screen[a]	D-17 washout	D-2	D-1	Days 1 & 16																														D2	D3	D4	D5	D6-7	D8	D9-10	D11	D12-13	D14	D15	D17	D18	D19	D20	D21	D22	Follow-up
Time					-0.5	-0.25	0	0.25	0.5	1	1.25	1.5	2	2.5	3	4	4.5	5	5.5	6	6.5	7.0	8	9	10	10.5	11	12	12.5	13	14	16	19	22																		
12-Lead ECG[k]	x			x			x						x							x								x							x	x	x	x	x	x	x	x	x	x	x	x	x	x	x			x
Oral dose[l]			x	x			x																													x	x	x	x	x	x	x	x	x	x							
PK sample collection[m]						x			x	x		x	x	x	x	x				x			x		x			x							x	x		x	x		x	x	x		x	x	x	x	x	x	x	
Urine collection[n]				x	x	x	x	x	x	x	x	x	x	x	x	x	x	x	x	x	x	x	x	x	x	x	x	x	x	x	x	x	x	x	x	x	x	x	x	x	x	x	x	x	x	x	x	x	x		x	
PD sample collection[o]				x	x	x	x		x	x	x	x	x	x	x		x	x	x	x	x	x	x	x		x	x	x	x	x	x	x	x	x	x	x	x	x	x	x	x	x	x	x	x	x	x	x	x	x	x	
Safety labs[p]	x		x	x																															x	x		x		x		x		x		x	x		x		x	x
PGx sample[q]							x																																													
Visual analogue scale[r]			x																																	x			x			x			x							
Fluid input-output[s]			x	x	x																														x	x	x	x	x	x	x	x	x	x	x	x	x	x	x			
Skin assessment[t]	x			x	x																														x	x	x	x	x	x	x	x	x	x	x	x	x	x	x			x
Telemetry[u]				x	x	x	x	x	x	x	x	x	x	x	x	x	x	x	x	x	x	x	x																x	x					x							
Hypoglycemia assessments[v]	ongoing throughout the study																																																			
AE/conmed recording	ongoing throughout the study																																																			
PRO questionnaire			x																																		x		x					x								

a Within 35 days prior to Day -3

b Existing anti-diabetic medication use discontinued on Day -16; subjects begin weight maintaining diet as instructed by the dietician and receive instruction in signs and symptoms of hypoglycemia, use of glucose analyzer and reporting of results on Day-17

c 7-10 days following the Day 22 visit

d Subjects will be admitted to the clinic on Day -3 but, no assessments will be performed

e Determine weight, BSA, and BMI at Screening. Fasting body weight on Days -1 through 20 and at the follow-up visit

f HBsAg, HIV and HCV

g (1) Post-menopausal is defined as no menses for at least 18 months prior to study start or no menses for 6 to 18 months prior to the start of this study, and plasma FSH must be ≥ 40 mIU/mL and estradiol ≤ 20 pg/mL in 3 separate measurements which will occur at Screening, Day -17, and Day -2 (a -2 day window is allowed for Day -2); (2) Pre-menopausal surgically sterilized subjects must have a negative β-HCG pregnancy test at Screening and at Day –2

h Urine drug screening, alcohol breath test

i Vital signs (blood pressure and heart rate) will be performed at Screening, and at T0, T2, T6 and T12 on Day –1 where T corresponds to the dosing time on dosing days. The vital signs will also be performed at 0 (predose), 2, 6, 12, 24, 48, 72 and 96 hours post dose on Days 1 and 16, and at 2, 6 and 12 hours on Days 3 to 15 and once at the follow-up visit.

j Orthostatic vital signs will be measured on Day –2 and Days 1 to 20 at 4 and 8 hours post dose or corresponding to dosing time.

k 12-lead ECGs will be performed at Screening, and at T0, T2, T6 and T12 on Day –1 where T corresponds the dosing time on dosing days. The 12-lead ECGs will also be performed at 0 (predose), 2, 6, 12, 24, 48, 72 and 96 hours post dose on Days 1 and 16, and at 2 hours on Days 3 to 15 and once at the follow-up visit. The 12-lead ECGs will be measured in triplicate 2 minutes apart at each predefined point. The average of the triplicate measurements at each time point on Day –1 will serve each subject's time-matched baseline value for the corresponding parameters on Days 1 to 20. All morning vital signs and 12-lead ECGs will be performed prior to dosing on study drug administration days and prior to breakfast on non-dosing days.

l Subjects are randomized on Day 1, then receive study medication after at least a 10 hour fast; on Day 1; study medication is resumed on Days 3-16; all subjects receive a placebo dose on Days -2 and -1; meal times must be recorded on the CRF

m Day 1: Plasma samples for measurement of JNJ-28431754 concentration will be collected at predose, 0.5, 1, 1.5, 2, 2.5, 3, 4, 6, 8, 10, 12, 24 and 48 hours postdose (total 14 samples). Days 3 to 15: Blood samples will be collected at pre-dose in the morning on Days 3, 5, 7, 9, 11, 13 and 15 to obtain trough JNJ-28431754 concentrations (total 7 samples) Day 16: Plasma samples for measurement of JNJ-28431754 concentration will be collected at predose, 0.5, 1, 1.5, 2, 2.5, 3, 4, 6, 8, 10, 12, 24, 48, 72, 96, 120 and 144 hours postdose (total 18 samples).

n PK Day 1: Urine samples for analysis of JNJ-28431754 will be collected at predose, 0-2, 2-4.5, 4.5-7, 7-10.5, 10.5-13, 13-24 and 24-48 hours post dose. Day 16: Urine samples for analysis of JNJ-28431754 will be collected at 0-2, 2-4.5, 4.5-7, 7-10.5, 10.5-13, 13-24, 24-48, 48-72 and 72-96 hours post dose.

PD:All urine will be collected, within specified intervals: on Day –1 at T0–T2, T2–T4.5, T4.5–T7, T7-10.5, T10.5-13, and T13-24, where T0 corresponds to the dosing time on dosing days. All urine will also be collected within specified intervals: 0-2, 2-4.5, 4.5-7, 7-10.5, 10.5-13, 13-24 and 24-48 hours postdose on Days 1 and 16, 48-72 and 72-96 on Day 16 and 0-24 hours on Days 3 to 15.

o Blood samples for plasma glucose, insulin and C-peptide will be collected on Day –1 at T-0.5, T-0.25, T0, T0.5, T1, T1.25, T1.5, T2.0, T2.5, T3.0, T4.5 (prior to lunch), T5.0, T5.5, T6.0, T6.5, T7.0, T8.0, T9.0, T10.5 (prior to dinner), T11, T12.0, T12.5, T13.0, T14.0 T16.0, T19.0, T22.0, and T24 hours where T0 corresponds to the dosing time on dosing days, and on Day 1 and 16 at -0.5, -0.25, 0, 0.5, 1, 1.25, 1.5, 2.0, 2.5, 3.0, 4.5 (prior to lunch), 5.0, 5.5, 6.0, 6.5, 7.0, 8.0, 9.0, 10.5 (prior to dinner), 11, 12.0, 12.5, 13.0, 14.0 16.0, 19.0, 22.0, and 24 hours postdose. Morning fasting plasma glucose, insulin and C-peptide concentrations will be obtained on Days 3 to 22. Blood samples for GLP-1 active and total will be collected on Days -1, 1 and 16 at -0.5 predose and 0.5, 1, 1.5 and 2.0 postdose

p Fasting samples for hematology, clinical chemistry and urinalysis dipstick will be collected at Screening, on Days -2, 2, 3, 5, 8, 11, 14, 17, 18, 20, and 22 and at the follow up visit. A fasting sample for serum creatinine clearance on Day -1 will be obtained. A sample for TSH is collected at screening. Samples for INR and aPTT will be collected at Screening and Day 20. Urine NTX and urine deoxypyridinolines (DPD), bone specific alkaline phosphatase and serum osteocalcin (OC) will be assessed on Days –2, -1, 2, 3, 5, 8, 11, 14, 17, 18, 20, and 22 and at the follow up visit. Serum 1,25-dihydroxy Vitamin D, and parathyroid hormone (PTH) will be assessed on Days -2, -1, 5, 11, 17, 22 and at the Follow-up visit.

q A 10 mL blood sample (Day 1 only) will be collected only from subjects who give informed consent for the pharmacogenomic part of the study

r Administration of visual analogue scale (VAS) questionnaires to assess appetite and satiety at 5 minutes prior to breakfast, 30, 60, 90, 120, 150, 180, 210 and 240 minutes after starting breakfast, 5 minutes prior to lunch and immediately after lunch on Days –2, 3, 7, 11 and Day 15.

s All fluid intake (water and all other beverages), urine volume and frequency of urination over each 24-hour period will be recorded in the eCRFs

t subjects will be closely monitored for potential skin reactions with a thorough examination of the whole body skin for skin reactions (e.g. erythema, rash) by a physician or his/her designee at Screening, on Days -1 through Day 20 at each dose level.

u Continuous Lead II ECG monitoring (telemetry) will be conducted from 30 minutes prior to dosing until 8 hours post dosing on Day -1, 1, 6, 8 and 15.

v Investigational staff will monitor subjects for symptoms possibly indicative of hypoglycemia throughout the in-residence period. Blood glucose will be immediately measured using a glucose analyzer and confirmed by a conventional venous blood sample, should symptoms possibly indicative of hypoglycemia appear.

w Adverse events will be recorded during the entire study and will be specifically queried on each dosing day at predose, 3, 6 and 12 hours post dose, once on Days 2, 20, 21, 22 and at the follow up visit.

ABBREVIATIONS

ADME	absorption, distribution, metabolism, excretion
Ae	amount excreted into the urine
Ae (% dose)	% of the administered dose excreted in urine as unchanged drug
AE(s)	adverse event(s)
ALT	alanine aminotransferase
ANOVA	analysis of variance
anti - HCV	serum antibody against Hepatitis C virus
AST	aspartate aminotransferase
AUC	area under the curve
AUC_{∞}	area under the curve from zero to infinite time
AUC_{t1-t2}	area under the curve from time 1 to time 2
BID	twice daily
BMI	Body Mass Index
BSA	Body Surface Area
BUN	blood urea nitrogen
bw	body weight
°C	degree Celsius
CI	confidence interval
CL/F	total clearance of drug after extravascular administration, corrected for absolute bioavailability
CL_R	renal clearance
C_{max}	maximum observed plasma concentration
CPK	creatine phosphokinase
CR	creatinine
CRF	case report form
CRU	Clinical Research Unit
CYP	Cytochrome P450 oxidase
DNA	deoxyribonucleic acid
DRC	Data Review Committee
DRF	dose range-finding
ECG	electrocardiogram
eCRF	electronic case report form
EDC	electronic data capture
FDA	Food Drug Administration
FIH	first in human
FOBT	fecal occult blood test
FPG	fasting plasma glucose
FSH	follicle stimulating hormone
GCP	Good Clinical Practice
GGT	gamma-glutamyl transpeptidase
GLP	Good Laboratory Practice
GLP-1	glucagon like peptide-1
GLUT1	glucose transporter 1
GMP	Good Manufacturing Practice

ABBREVIATIONS (CONTINUED)

h	hour
HbA1c	hemoglobin A1c
HBsAg	hepatitis B surface antigen
hCG	human chorionic gonadotrophin
HED	human equivalent dose
HIV	Human Immunodeficiency Virus
HPLC	high-performance liquid chromatography
i.p.	intraperitoneal
i.v.	intravenous
IC50	inhibiting concentration at 50%
ICH	International Conference on Harmonization
IEC	Independent Ethics Committee
IRB	Institutional Review Board
J&JPRD	Johnson & Johnson Pharmaceutical Research & Development
kg	kilogram
LC-MS	liquid chromatography coupled to mass spectrometry detection
LC-MS/MS	liquid chromatography coupled to tandem mass spectrometry detection
LOAEL	lowest observed adverse effect level
λ_ζ	elimination rate constant
MedDRA	Medical Dictionary for Regulatory Activities
µg	microgram
mg	milligram
mL	milliliter
mRNA	messenger ribonucleic acid
MRSD	maximum recommended starting dose
MS	mass spectrometry
n	number (size of a sub-sample)
N	number (total sample size)
NAG	N-acetyl-ß-D-glucosaminidase
ng	nanogram
NOAEL	No Observed Adverse Effect Level
OC	osteocalcin
OGTT	oral glucose tolerance test
PD	pharmacodynamic
PG	pharmacogenomic
PI	Principal Investigator
PK	pharmacokinetic
PTH	parathyroid hormone
QTc	heart-rate corrected QT time interval in ECG
QTcB	corrected QT interval based on Bazett’s method
QTcF	corrected QT interval based on Fridericia’s method
RBC	red blood cell
SAD	single ascending dose

ABBREVIATIONS (CONTINUED)

SGLT	sodium glucose co-transporter
SOP(s)	standard operating procedure(s)
$t_{½}$	terminal half life
T2DM	Type 2 Diabetes Mellitus
t_{max}	time to attain maximum observed plasma concentration
USP	United States Pharmacopeia
UV	ultra violet
UVA	ultra violet A light
VAS	visual analog scale
Vd/F	apparent volume of distribution following oral administration
vs.	versus
WBC	white blood cell
WHO	World Health Organization
ZDF	Zucker diabetic fatty

1. INTRODUCTION

JNJ-28431754 is a potent, selective and reversible inhibitor of the renal sodium glucose co-transporter (SGLT2). It is being investigated for the treatment of Type 2 diabetes mellitus (T2DM).

T2DM is a metabolic disorder characterized by 2 primary defects: decreased insulin secretion by the pancreas, and resistance to insulin action in peripheral tissues (muscle, liver and adipose), which results in impaired glucose uptake and hyperglycemia. Chronic hyperglycemia leads to progressive impairment of insulin secretion and further insulin resistance of peripheral tissues (so-called glucose toxicity[1, 2]), which further worsens control of blood glucose. In addition, chronic hyperglycemia is a major risk factor for diabetic complications, including heart disease[3], retinopathy[4], nephropathy[5] and neuropathy[6]. Aggressive glycemic control decreases microvascular[7] and perhaps macrovascular complications.[8]

Although existing antidiabetic therapies can be effective, it can still be difficult to maintain optimal glycemic control in many diabetics. High rates of morbidity and mortality persist in the diabetic population. Thus, new antidiabetic agents that provide improved efficacy, safety and tolerability and that can be used in combination with existing therapies will be valuable additions for the treatment of Type 2 diabetes.

There are at least four isoforms of the SGLTs: SGLT1, SGLT2, SGLT3, and SGLT4. In healthy humans, the renal glomerula filter approximately 180 g of glucose from plasma each day. Most all of the filtered glucose is reabsorbed in the convoluted proximal tubule by SGLT2, a low-affinity, high-capacity co-transporter with a Na^+-glucose transport ratio of 1:1. Only a small proportion of the filtered glucose is absorbed in the straight proximal tubule by SGLT1. Both SGLT1 and SGLT2 are located in the apical plasma membranes of the tubular epithelium and derive their energy from the inward sodium gradient created by the Na^+/K^+ ATPase pumps located on the basolateral membrane. Once reabsorbed across the apical membrane, the elevated cytosolic glucose is then transported across the basolateral membrane, into the interstitial space by facilitated glucose transporters (GLUT1 and GLUT2).[9, 10]

In normal mice, rats and dogs and several rodent models of diabetes, JNJ-28431754 promotes urinary glucose excretion. Treatment with

JNJ-28431754 in rodent models of diabetes led to improved glucose tolerance and reduced fasting and postprandial serum glucose concentrations and HbA1c levels. In the two-week rat and dog toxicology studies, JNJ-28431754 increased urinary glucose secretion, and at higher exposures, decreased serum glucose concentrations. However, there were no significant changes in electrolyte balance and renal function[11]

This will be the first study of JNJ–28431754 in diabetic subjects. This study will evaluate the safety, tolerability, pharmacokinetics and pharmacodynamics of multiple oral ascending doses of JNJ-28431754 in male and postmenopausal or surgically sterilized female T2DM subjects. Pre-clinical and also clinical data (from the first single oral dose clinical study of JNJ-28431754) that support this multiple ascending dose study are summarized in Section 1.1. of this protocol. More detailed information regarding JNJ–28431754 can be found in the JNJ–28431754 Investigator's Brochure.[11]

The term Sponsor used throughout this document is referred to the entities listed in the Contact Information page(s), provided as a separate document.

1.1. Background

1.1.1. Pre-clinical Data

Pharmacology[11]

JNJ-28431754 is a potent, selective SGLT2 inhibitor in cultured Chinese Hamster Ovary CHOK1 cells expressing either human SGLT1 or SGLT2. In these cells, JNJ-28431754 inhibits Na^+-dependent C-α-methylglucoside uptake with an IC_{50} of 4.1 nM (1.8 ng/mL) and 664 nM (295 ng/mL) against SGLT2 versus SGLT1, respectively. A similar IC_{50} of 2.0 nM (0.9 ng/mL) was obtained for murine SGLT2. JNJ-28431754 had no significant inhibitory activity against the rat skeletal muscle GLUT1 (IC_{50} > 10000 nM).

Single oral doses of JNJ-28431754 administered prior to an oral glucose challenge produced a dose-dependent increase in urinary glucose excretion and urine volume in normal mice, diabetic rats and non-diabetic obese dogs. These pharmacological activities were related to drug exposure ($AUC_{0\text{-}24h}$) of JNJ-28431754 in plasma. Chronic treatment for 4 weeks with JNJ-28431754 resulted in a dose-dependent lowering of fed and fasted blood glucose levels as well as HbA1c in diabetic mice and rats. Moreover, chronic

treatment with JNJ-28431754 improved beta-cell function as reflected in a dose-dependent increase in plasma insulin levels in diabetic mice.

In addition, following chronic administration with JNJ-28431754 in ob/ob or diet-induced obese mice for 4 weeks, or in Sprague-Dawley rats for 2 weeks, JNJ-28431754 caused dose dependent reduction in body weight gain in the ob/ob mice and Sprague Dawley rats witthout significant increase in food intake, and caused body weight loss in diet-induced obese mice.

Safety Pharmacology and Toxicology[11]

Receptor Binding/ Selectivity

JNJ-28431754 was tested for in vitro inhibition of binding of relevant ligands to 50 recombinant human receptors. At 10 μM (free drug concentration of about 4.4 μg/mL; total drug concentration of about 220 μg/mL), JNJ-28431754 inhibited ligand binding to the NE transporter by 34% and inhibited ligand binding to 5-HT2A receptors by 37%. Binding to all other receptors was inhibited by less than 20%.

Safety Pharmacology

Nonclinical cardiovascular and pulmonary safety pharmacology studies suggested that JNJ-28431754 has a low potential for inducing adverse cardiovascular or pulmonary effects. JNJ-28431754 did not reduce IKr current in hERG-transfected HEK293 cells at the highest tested plasma concentration of 3 μM (free drug concentration of about 1.3 μg/mL; total drug concentration of about 65 μg/mL). In Langendorff-perfused rabbit hearts, JNJ-28431754 had no notable effect on electrophysiological parameters at the highest tested plasma concentration of 1 μM (free drug concentration of about 0.4 μg/mL; total concentration of about 20 μg/mL). In anesthetized guinea pigs, JNJ-28431754 had no drug-related cardiovascular effects detected at cumulative intravenous doses up to 9.86 mg/kg (a plasma drug concentration of approximately 13 μg/mL). In conscious dogs, oral doses up to 400 mg/kg (mean plasma concentration at 24 h post-dose of approximately 55 μg/mL) of JNJ-28431754 were not associated with notable cardiovascular and pulmonary effects. JNJ-28431754 did not elicit notable neurobehavioral effects in male rats at single doses up to 1000 mg/kg.

Toxicology

In single dose studies in mice and rats over an oral dose range of 250 to 2000 mg/kg, or over an i.p. dose range of 62.5 to 500 mg/kg, JNJ-28431754 was well tolerated in mice. The maximum non-lethal doses of JNJ-28431754 in mice were 2000 mg/kg and 500 mg/kg after a single oral and i.p dose, respectively. One of five female rats died after oral dosing (2,000 mg/kg), and 1/5 and 3/5 male rats died after receiving i.p. doses of 250 and 500 mg/kg, respectively. General gastrointestinal findings of abnormal feces were noted in both mice and rats after both routes of administration. The maximum non-lethal doses of JNJ-28431754 in rats were 1000 mg/kg and 125 mg/kg after a single oral and i.p dose, respectively.

In a 5-day dose finding (dose range: 10, 50, 250 and 500 mg/kg; N=5/sex/group) study in rats, one female rat at 500 mg/kg dose was terminated in moribund condition on Day 4; the cause of moribund condition for this animal was undetermined. There was a slight increase in stress associated peracute gastric erosions seen histologically in rats dosed at ≥ 50 mg/kg/day.

In 14-day GLP toxicology studies in rats (dose range: 3, 20 and 150 mg/kg; N=10/sex/group) slight increases in serum BUN, ALT, and AST were noted in rats dosed at ≥ 20 mg/kg/day. These changes were only slightly higher than controls, and had no histopathologic correlate; they are not considered toxicologically relevant. Microscopically, minimal to mild, acute, superficial gastric erosions in the glandular portion of the stomach were noted in all dose groups including controls: 1/10 female control rat, 2/10 female rats at 3 mg/kg and in both male and female rats at 20 mg/kg (3/10 and 2/10) and 150 mg/kg (4/10 and 4/10), respectively. Although this appears to be indicative of a dose-response, subsequent investigative toxicology studies have demonstrated that these are peracute responses, attributed to the stress of the combination of overnight fast and the bleeding procedure on the morning of necropsy. The increased incidence with dose in this study is thought to be related to the added effect of the additional stress associated with the compound such as the low blood glucose levels observed at the high dose in the study. These acute changes to the gastric mucosa are not considered primary, direct effects of the compound. The no observed adverse effect levels (NOAEL) for this study were determined to be 20 mg/kg for males and females based on the incidence of hyperostosis

(minimal to mild changes in distal end of femur & proximal end tibia) at 150 mg/kg. All other changes noted in the study were not considered to be toxicologically significant. The C_{max} and AUC_{0-24} values at the NOAEL were 4,690 ng/mL and 68,644 ng.h/mL in males and 5,795 ng/mL and 80,355 ng.h/mL in females, respectively. The lowest observed adverse effect level (LOAEL) associated with the observed hyperostosis were 32,850 ng/mL and 511,759 ng.h/mL in males and 45,775 ng/mL and 748463 ng.h/mL in females, respectively.

In a 5-day dog toxicology study (dose range:25, 100, 400 and 800 mg/kg; one dog of each gender at each dose level), a female dog at 800 mg/kg dose was euthanized on Day 5. Key toxicity findings from the dead animal include marked hypoglycemia (0.26x of control serum glucose), dehydration, high urine ketone, and lung findings suggestive of possible aspiration of emesis. No significant toxic effect was seen in dogs treated for 2 weeks with oral doses up to the NOAEL of 400 mg/kg (C_{max}: 40,200/76,800 ng/mL, M/F; AUC_{0-24h}: 539,359/1,067,058 ng.h/mL, M/F).

In the 2-week GLP toxicity study in dogs (dose range:4, 40 and 400 mg/kg, N=3/sex/group), minimal signs of toxicity were noted. The NOAEL for this study was 400 mg/kg/day. The corresponding C_{max} and AUC values at the NOAEL were 40,200 ng/mL and 539,359 ng.h/mL in males, and 76,800 ng/mL and 1,067,058 ng,h/mL in females.

In repeat dose studies up to 2 weeks in both rats (dose range 10-500 mg/kg/day) and dogs (dose range 4-800 mg/kg/day), pharmacological effects of JNJ-28431754 were evident in the clinical chemistry and urinalysis data. Elevated urinary glucose excretion along with increased urine volume and minimal to moderate decreases in serum glucose levels were observed. There were no meaningful changes in serum and urine electrolytes except that urinary excretion of calcium was increased up to 10 fold in rats and 4 fold in dogs.

In most dogs receiving 200 mg/kg/day in an ongoing 3 month GLP toxicology study, excessive diarrhea/loose stools and emesis were observed during the second week of the study. In this same group (n = 10; 5 dogs/sex), evidence of blood in stools was observed in 3/5 males. In general the animals of this group were depressed, inactive and some not eating and some appeared dehydrated. Upon lowering the dose to 100 mg/kg/day, the dogs

regained their appetites and, no longer show blood in stools, but the other fecal changes and emesis continue to occur. Occasionally mucoid feces and emesis have been observed in mid dose dogs (30 mg/kg/day, n = 6; 3/sex). These signs are probably related to the watery stools that were observed in an earlier study in dogs treated orally for 5 days with JNJ-2841754 at doses greater than or equal to 100 mg/kg/day.

Phototoxicity

In vitro cytotoxicity and phototoxicity of JNJ-28431754 were assessed by neutral red uptake assays in cultures of normal Balb/c 3T3 mouse fibroblasts following exposure to ultraviolet A light (UVA). Results indicated that JNJ-28431754 was phototoxic in vitro. A confirmatory in-vivo phototoxicity study was subsequently conducted in Sprague-Dawley rats following a single oral dose administration of JNJ-28431754 at 5, 50 and 500 mg/kg dose levels. Results from this study indicated that JNJ-28431754 was not phototoxic at the lowest dose, but was phototoxic to the skin (mild erythema) of female rat (in 1 of 5 female rats) at the mid dose, and to the skin of both males (mild erythema and edema in 1 of 5 male rats) and females (mild erythema and mild to moderate edema in 3 of 5 female rats) at the highest dose. These skin reactions occurred one day after the study drug administration and a UVA and UVB radiation (UVR) exposure. JNJ-28431754 was not associated with eye phototoxicity at any dose level tested in this study based on clinical, ophthamological and histopathological assessments.

Carcinogenicity, Mutagenicity

Genotoxicity studies completed thus far on JNJ 28431754 include the Ames test (negative in 5 strains tested: Salmonella strains: TA98, TA100, TA1535, TA1537; E. Coli strain: WP2uvrA), the in vitro mouse lymphoma assay (negative in the absence of S9 microsomes but positive in the presence of S9 microsome fraction from induced rat livers), the in vivo, single dose rat micronucleus test (negative) and the in vivo, single dose COMET study in rats (negative in rat liver). Mean plasma concentrations of JNJ-28431754 measured at 2 and 6 hours post dose were in the range of 125-136 mg/mL, which is approximately 25 fold higher than the Cmax observed at the maximum dose of 800 mg (Cmax 5.2 mg/mL) administered in the first in human study. From the panel of studies conducted, the in vivo COMET

study is currently considered a good indicator of in vivo genotoxic potential and is the most appropriate test to determine the relevance of the positive in vitro findings in the mouse lymphoma test. The negative outcome of the COMET test proves that JNJ-28431754 even after metabolic activation in vivo is not DNA-reactive to rat hepatocytes. Based on the negative findings in the COMET assay, and all other negative results in the in vitro and in vivo tests, the overall genotoxic potential of this compound is considered minimal towards humans.

Other toxicity studies

In an invitro bovine corneal opacity-permeability (BCOP) assay, JNJ-28431754 as a 20 % (w/w) suspension induced no increase in corneal opacity and a small increase in permeability. From this assay, a score of 3.2 ± 0.4 was calculated, which classified the RWJ-416457 formulation as a borderline non to mild eye irritant.

In a study to evaluate the skin sensitization potential of JNJJ-28431754 in mice at concentrations of 2.5, 15, 30 and 100 g, JNJ-28431754 did not cause redness or swelling of the ear of the mice, and radioactivity measurements in mice treated with JNJ 28431754 were not different from control animals. Thus, the stimulation index (SI) was less than 3.0 indicating the test article is not a contact sensitizer under the conditions of the study.

Based on available data from JNJ-28431754 safety and toxicity studies, potential dose-limiting human intolerability or toxicities may include, but are not limited to, osmotic diuresis due to increased urinary glucose excretion, changes in serum or urine electrolytes, GI intolerability, hypoglycemia, changes in bone formation and or in bone resorption, abnormalities in hepatic and renal functions, and phototoxicity.

Pharmacokinetic Profile[11]

Following single oral dose administration of JNJ-28431754 in mice, rats, dogs and monkeys, mean peak concentrations (t_{max}) were reached at 0.5 to 5.5 hours post-dose. The mean $t_{1/2}$ ranged from 3 to 8 hours with absolute oral bioavailability ranging from approximately 34% to 137%.

In the 2-week rat toxicology studies, mean C_{max} and $AUC_{0\text{-}24h}$ values increased more than proportionately with increased doses after single dose administration. For doses in a ratio of 1:7:50, the C_{max} and $AUC_{0\text{-}inf}$ values increased in a ratio of about 1:8:80 and 1:10:135, respectively. However,

following multiple dose administrations, the C_{max} and $AUC_{0\text{-}24h}$ values increased dose proportionately. Mean t_{max} and $t_{1/2}$ values ranged approximately from 2 to 9 hours and 7 to 11 hours, respectively. At the high dose (150 mg/kg), C_{max} and AUC values decreased about 2 fold after 2-weeks of dosing compared to the first dose.

Following 2-week dosing of JNJ-28431754 in dogs, increases in C_{max} and $AUC_{0\text{-}24h}$ values were less than dose proportional, likely due to emesis or absorption saturation. The drug exposure ($AUC_{0\text{-}24h}$) was up to 2.5 fold higher on Day 14 after multiple dosing, than the initial single dosing on Day 1, suggesting possible drug accumulation at steady state. Mean t_{max} and $t_{1/2}$ values ranged from 1 to 3 hours and 9 to 14 hours, respectively. C_{max} and AUC values were generally higher in females than in males in both rats and dogs. The difference in Cmax and AUC values rangedabout 10 to 90%. JNJ-28431754 has a plasma protein binding of 98% in all species tested, and the protein binding was concentration independent. The predominant plasma protein responsible for binding was albumin.

In vitro, unchanged JNJ-28431754 accounted for approximately 50% of drug-derived components in hepatocytes of rat, dog, and human. The major metabolic pathway of JNJ-28431754 in both rat and dog species was oxidation to a carboxy metabolite, while the major metabolite in human hepatocytes was from glucuronidation of the hydroxyl functionality on 2-hydroxymethyl-tetrahydro-pyran-3, 4, 5-triol moiety (detected for a total of 42%). All the metabolites identified in the human hepatocytes were also found in rat and/or dog hepatocytes. JNJ 28431754 was moderately metabolized in rats and dogs in vivo. The metabolic profile was qualitatively similar but quantitatively different between these two species. In rats and dogs, unchanged drug was the major drug-related material in systemic circulation. Mono oxygenation (aromatic hydroxylation) and alcohol oxidation were the predominant biotransformation pathways in the rat, while O-glucuronidation and mono-oxygenation (aromatic hydroxylation) pathways appeared to be more pronounced in the dog

In human hepatocyte cultures, JNJ-28431754 did not cause induction of mRNA or enzyme activity of CYPs 3A4, 2C9, 2C19 and 1A2 at concentrations up to 10 μM (4.4 μg/mL). The ability of JNJ-28431754 to inhibit major human cytochrome P450 (CYP) enzymes was evaluated in

vitro using human hepatic microsomes pooled from adult males and females. JNJ-28431754 showed a weak inhibition of CYP3A4 (testosterone) and CYP2C9 (tolbutamide) with IC50 values of 27 μM (12 μg/mL) and 80 μM (35.6 μg/mL), respectively. The calculated IC50 values for other CYP isoforms were >100 μM. JNJ-28431754 did not exhibit any potential for mechanism-based inactivation of the screened CYPs 1A2, 2C19, 2C9, 2D6, and 3A4. Overall, the drug has a low potential for involvement in CYP-based drug-drug interactions.

JNJ-28431754 is both a substrate and a weak inhibitor of p-glycoprotein (encoded by the MDR1 gene) and the multi-drug resistance associated proteins (MRP2) with IC50 of 19.3 μM (8.5 μg/mL) and 21.5 μM (9.5 μg/mL), respectively.

Following oral administration of 14C-JNJ-28431754 in male rats, a greater percentage of radioactivity was excreted in feces than urine at 25 hours after administration.

Clinical Data

Phase I Clinical Trials of JNJ-28431754

A First-in-Human Study to Evaluate Safety, Tolerability, Pharmacokinetics and Pharmacodynamics of Single Escalating Oral Doses of JNJ-28431754 in Healthy Adult Male Subjects (Protocol 28431754NAP1001)

The first Phase I study (Protocol 28431754NAP1001) to evaluate the safety, tolerability, pharmacokinetics (PK) and pharmacodynamics (PD) of single escalating oral doses of JNJ-28431754 is ongoing at a single study center in the Netherlands.

Part 1 of this study is a double-blind, randomized, placebo-controlled, sequential parallel groups, ascending single oral dose study in healthy normal male subjects. In each group (for each evaluated dose level), 6 subjects are randomized to JNJ-28431754 and 2 subjects to placebo. Subjects are admitted to the Clinical Research Unit (CRU) on Day –2 for baseline safety and PD assessments. On Day 1, after an overnight fast of ~10 hours, subjects receive study medication at ~8 AM, and have a standardized breakfast, lunch, and dinner at 0.5h, 4.5h, and 10.5h, respectively, post-dose. Subjects are domiciled in the CRU for frequent safety, tolerability, PK, and

PD assessments at specified time points until Day 5, when they may be discharged. Subjects return to the CRU for the final safety follow-up visit at 10-14 days post-dose. Assessments in Part 1 include:

Safety and tolerability

Adverse events, physical examination including whole body skin exam, vital signs, daily fluid intake and urine volume, telemetry and 12-lead ECGs, laboratory tests [routine hematology, clinical chemistry, urinalysis (including electrolytes, osmolality, protein, amino acids, tubular enzymes), and fecal occult blood tests (FOBTs)].

Pharmacokinetics

Blood and urine sampling for determination of plasma and urine JNJ-28431754 concentrations and potential metabolite metabolite profiling and/or metabolite quantification.

Pharmacodynamics

Cumulative urinary glucose excretion (UGE, in grams) over each 24-hour period (UGE/24h), UGE rate (grams/hr) over each timed urine collection interval, and 24-hour plasma glucose and insulin profiles.

In Part 2 of this study, the effect of co-administering JNJ-28431754 together with a standard high-fat breakfast, compared to administration in the fasting condition, on the PK and PD of JNJ-28431754 will be evaluated in 8 healthy male subjects. Part 2 is a balanced, 2-way crossover design with a washout period of at least 14 days between doses. Safety and tolerability will also be assessed as in Part 1.

As of March 21, 2007, 7 cohorts (total of 55 healthy men) have been dosed in Part 1 of this study at doses of 10, 30, 100, 200, 400, 600 and 800 mg of JNJ-28431754 or placebo (in the 800 mg cohort, 7 subjects have been dosed). A summary of safety, tolerability, PK and PD based on blinded, preliminary data from the 7 completed cohorts of Part 1 are included below.

Safety and Tolerability

Single oral doses of JNJ-28431754 at 10, 30, 100, 200, 400, 600 and 800 mg as a liquid suspension (5 or 50 mg/mL in 0.5% hypromellose vehicle) or matching placebo (0.5% hypromellose) have been generally well-tolerated.

To date, there have been no deaths, serious adverse events, or discontinuations due to adverse events.

Adverse Events

In the 7 cohorts of Part 1 of this study, 22 (40 %) of 55 subjects experienced at least 1 treatment-emergent adverse event (See blinded listing, Table 1). The majority of adverse events were either mild (43 [96%] of 45) or moderate (2 [4%] of 45) in severity. The most frequent adverse events were fatigue and headache (each 4 [9%] of 45), followed by postural dizziness (3 [7%] of 45). Twenty-four (24) of 45 (53%) adverse events were considered possibly related to the study drug. The remaining adverse events were considered either not related (14 [31%] of 45), or of doubtful relationship to study drug (7 [16%] of 45). No hypoglycemia was observed at any dose level. There was no apparent relationship between dose level and the type, severity or incidence of adverse events.

In Cohort 1 (10 mg or placebo), Subject 1005 reported moderate chest discomfort at 1-hour post dose, which lasted about 17 minutes, but without changes in vital signs or ECG parameters. This subject also developed mild, localized cutaneous erythema on the anterior surface of both knees 5 days after dosing, which had resolved by the follow-up visit. Subject 1002 developed transient postural hypotension accompanied with dizziness, which lasted about 2 minutes, at 2 hours post dose [Supine systolic blood pressure: 115 mmHg, Standing systolic blood pressure: 92 mmHg, Systolic blood pressure (Standing-Supine): -23 mmHg; Supine diastolic pressure: 55 mmHg, Standing diastolic blood pressure: 33 mmHg, Diastolic blood pressure (Standing-Supine): -22 mmHg; Supine pulse rate: 64 bpm, Standing pulse rate: 75 bpm, Pulse rate (Standing-Supine): 11 bpm].

In Cohort 2 (30 mg or placebo), Subject 1016 reported persistent whole body dry skin, mild in severity, on Day 3 post dose. The dry skin resolved at the follow up visit.

In Cohort 3 (100 mg or placebo), Subject 1020 was observed to have a mild, multi focal macular rash on the dorsal surface of both feet beginning at about 3 days post dose. The rash was accompanied by mild pruritis. The rash and pruritis resolved on the following day.

In Cohort 4 (200 mg or placebo), Subject 1028 complained of a burning skin sensation over his whole body approximately 30 minutes post dose. The sensation lasted about 1 hour. There was no skin abnormality observed. Subject 1029 reported fatigue about 2 hours post dose, which resolved 3.5 hours post dose. There were no clinically meaningful changes in any of his clinical chemistry lab test results, including plasma glucose and serum electrolytes on Day 1.

In Cohort 5 (400 mg or placebo), Subject 1033 reported fatigue that started 1-hour post dose and lasted 21 hours. The subject also reported abdominal fullness on Day 2, which resolved by his follow-up visit. The subject excreted 62 and 39 g of glucose in the urine over 0-24 and 24-48 hours post-dose, respectively. There were no significant changes in his urine volume, urine sodium excretion and serum electrolytes post dose compared to his baseline values. Subject 1036 experienced mild myalgia beginning at 48 hours post dose that resolved during his follow-up visit. The subject had normal CPK and LFT values post dose. His UGE/24 values were 72 and 33 g on Days 1 and 2, respectively. The subject's urine volume and urine sodium excretion were not significantly changed post dose compared with his baseline values. There were slight decreases in his serum sodium (131 mmol/L) and calcium (2.08 mmol/L) levels on Day 1 compared to his baseline Day -1 values (serum sodium: 138 mmol/L; serum calcium: 2.18 mmol/L). His serum sodium and calcium values returned to within the normal range on Day 2. Subject 1040 experienced a warm feeling and moderate headache about 6 hours post dose, which lasted about 7 hours. There were no clinically significant abnormalities in his vital signs and ECGs. The subject excreted 86 and 55 g of glucose in the urine over 0-24 hour and 24-48 hours, respectively. There were no significant changes in his urine volume, urine sodium excretion and serum electrolytes post dose compared with the baseline values.

In Cohort 6 (600 mg or placebo), Subject 1043 developed postural hypotension (Note: This adverse event was reported by the investigator as postural dizziness) 4-hours post dose [Supine systolic blood pressure: 118 mmHg, Standing systolic blood pressure: 84 mmHg, Systolic blood pressure (Standing-Supine): -34 mmHg; Supine diastolic pressure: 63 mmHg, Standing diastolic blood pressure: 53 mmHg, Diastolic blood pressure (Standing-Supine): -10 mmHg; Supine pulse rate: 75 bpm, Standing

pulse rate: 105 bpm, Pulse rate (Standing-Supine): 30 bpm]. The postural hypotension was accompanied by a transient dizziness, which lasted 4 minutes. The subject excreted about 90 and 63 grams of glucose in the urine over the 0-24 and 24-48 hour urine collection intervals. There were no increases in his urine volume and sodium excretion post dose compared with the baseline values. His serum electrolytes on Days 1 and 2 were comparable to his baseline Day –1 values. Two subjects reported adverse skin reactions. Subject 1044 experienced a 4-5 cm diameter area of cutaneous erythema on his neck 3 days post dose. The erythema was accompanied by mild pruritis and resolved during the follow up period. Subject 1045 developed a few acne-like pustules on his back and extremities on Day 2. The pustules resolved on Day 5. Three subjects experienced mild adverse gastrointestinal reactions. Subject 1044 reported abdominal cramps beginning at about 2 hours post dose, which lasted about 2 hours. Subjects 1043 and 1045 reported loose stool at about 5 and 2 hours post dose, lasting about 2 days and 5.5 hours, respectively.

In Cohort 7 (800 mg or placebo), all events were mild in intensity. Subject 1052 experienced whole body dry skin, mild in severity, on Day 2 post dose. The dry skin lasted about one day. Subject 1056 developed mild diarrhea (loose stool 5 times) beginning at about 1 hour post dose, which lasted about 12 hours. The diarrhea was not accompanied by any other symptom.

Table 1: Blinded Incidence of Treatment Emergent Adverse Events by System Organ Class and Preferred Term
(Study JNJ28431754-NAP1001-Part 1: Preliminary Safety Analysis Set)

**Body System or Organ Class**<br>Preferred Term	10mg or placebo (N=8)<br>n (%)	30mg or Placebo (N=8)<br>n (%)	100mg or placebo (N=8)<br>n (%)	200mg or placebo (N=8)<br>n (%)	400mg or placebo (N=8)<br>n (%)	600mg or placebo (N=8)<br>n (%)	800mg or placebo (N=7)<br>n (%)
**Total Number of Subjects With Adverse Events**	4 (50)	1 (13)	2 (25)	2 (25)	4 (50)	6 (75)	3 (43)
**Gastrointestinal disorders**	1 (13)	0	0	0	2 (25)	2 (25)	1 (14)
Abdominal distension	0	0	0	0	2 (25)	0	0
Abdominal pain	1 (13)	0	0	0	0	1 (13)	0
Constipation	0	0	0	0	1 (13)	0	0
Diarrhoea	0	0	0	0	0	1 (13)	1 (14)
**General disorders and administration site conditions**	3 (38)	0	0	1 (13)	3 (38)	2 (25)	0
Catheter site related reaction	1 (13)	0	0	0	1 (13)	0	0
Chest discomfort	1 (13)	0	0	0	0	0	0
Fatigue	0	0	0	1 (13)	2 (25)	1 (13)	0
Feeling hot	1 (13)	0	0	0	1 (13)	1 (13)	0
**Infections and infestations**	1 (13)	0	1 (13)	0	1 (13)	1 (13)	0
Nasopharyngitis	1 (13)	0	1 (13)	0	1 (13)	0	0
Rash pustular	0	0	0	0	0	1 (13)	0
**Musculoskeletal and connective tissue disorders**	0	0	0	0	1 (13)	1 (13)	0
Back pain	0	0	0	0	0	1 (13)	0
Myalgia	0	0	0	0	1 (13)	0	0
**Nervous system disorders**	2 (25)	0	1 (13)	1 (13)	1 (13)	2 (25)	1 (14)
Dizziness	1 (13)	0	1 (13)	0	0	0	0
Dizziness postural	1 (13)	0	0	0	0	2 (25)	0

Note: Percentages calculated with the number of subjects in each group as denominator.
Coding Dictionary Version: MEDDRA V9.1

Table 1: Blinded Incidence of Treatment Emergent Adverse Events by System Organ Class and Preferred Term (Continued)
(Study JNJ28431754-NAP1001-Part 1: Preliminary Safety Analysis Set)

Body System or Organ Class<br>Preferred Term	10mg or placebo (N=8) n (%)	30mg or Placebo (N=8) n (%)	100mg or placebo (N=8) n (%)	200mg or placebo (N=8) n (%)	400mg or placebo (N=8) n (%)	600mg or placebo (N=8) n (%)	800mg or placebo (N=7) n (%)
**Nervous system disorders (continued)**							
Headache	0	0	1 (13)	1 (13)	1 (13)	0	1 (14)
Paraesthesia	1 (13)	0	0	0	0	0	0
**Respiratory, thoracic and mediastinal disorders**	0	0	0	0	0	1 (13)	1 (14)
Pharyngolaryngeal pain	0	0	0	0	0	1 (13)	1 (14)
**Skin and subcutaneous tissue disorders**	1 (13)	1 (13)	1 (13)	1 (13)	0	1 (13)	2 (29)
Dry skin	0	1 (13)	0	0	0	0	1 (14)
Erythema	1 (13)	0	0	0	0	1 (13)	0
Pruritus	0	0	0	0	0	1 (13)	0
Rash macular	0	0	1 (13)	0	0	0	0
Skin burning sensation	0	0	0	1 (13)	0	0	0
Skin irritation	0	0	0	0	0	0	1 (14)
**Vascular disorders**	1 (13)	0	0	0	0	0	0
Orthostatic hypotension	1 (13)	0	0	0	0	0	0

See footnotes on the first page of the table.

Clinical laboratory tests

No clinically significant treatment-emergent changes were observed in hematology, clinical chemistry and urinary analyses. Although values for some parameters were slightly outside the normal range at isolated time points for all treatments, these changes were transient and were not considered clinically significant. Except for dose-dependent increases in urinary excretion of glucose - reflecting the expected pharmacological effect of the study drug - there were no dose-dependent increases or decreases in serum electrolytes and osmolality, urinary excretion of electrolytes, creatinine, albumin and tubular enzymes. Daily Fecal Occult Blood Tests (FOBTs) were negative for all subjects.

Vital signs

There were no indications of any consistent treatment effect on supine or standing systolic and diastolic blood pressure or pulse rate following administration of the study drug. Two subjects developed mild, transient postural hypotension accompanied by dizziness: Subject 1002 at 2-hour post dose (10 mg) and Subject 1043 at 4-hour post dose (600 mg) as described in the adverse event section, above.

There were no indications of any consistent, or dose-dependent treatment effects on daily fluid intake and urinary volume, despite significant, dose-related increases in UGE over the 0-24 hr and 24-48 hr post-dose periods.

12-lead electrocardiograms

There were no consistent or dose-related changes in the 12-lead ECG parameters (heart rate, PR interval, QRS interval, QT interval, QTcB [Bazett's correction] or QTcF [Fridericia's correction] following administration of JNJ-28431754. There were no abnormalities noted during the post-dose telemetry monitoring period (0-6 hours post dose).

Pharmacokinetics

Preliminary plasma concentration time profiles and PK parameters for JNJ-28431754 following single oral doses of 10 to 800 mg are presented in Figure 1 and 2 and Table 2. JNJ-28431754 was rapidly absorbed into the systemic circulation with median t_{max} values in the range of 1.25 to 1.75 hours. Mean C_{max} and $AUC_{0-\infty}$ values increased with increasing dose up

to 600 mg. Normalization of individual C_{max} or $AUC_{0-\infty}$ values with the administered dose also demonstrated consistent systemic exposures up to 600 mg dose. Thereafter an increase in dose from 600 mg to 800 mg resulted in less than proportional increase in the AUC values. The Cmax values were comparable between 600 mg and 800 mg doses. These results suggest that the gastrointestinal absorption of JNJ-28431754 may have saturated at the 600-800 mg dose level.

The elimination of JNJ-2841754 appeared to be biphasic, with mean (SD) apparent terminal elimination half-lives (t1/2) ranging from 8.39 (4.20) to 13.6 (1.49) hours across the dose range. The mean (SD) clearance and apparent volume of distribution values of JNJ-2841754 across all doses ranged from 15.1 (2.62) to 22.4 (5.59) L/h, and from 240 (8.3) to 310 (37.4) L, respectively.

Figure 1: Mean plasma concentration-time profiles of JNJ-2841754 in healthy normal male subjects following a single oral dose of JNJ-2841754-linear scale (preliminary results, Protocol JNJ-2841754 NAP1001) (Note: only a 24-hour profile included for the 800 mgdose group)

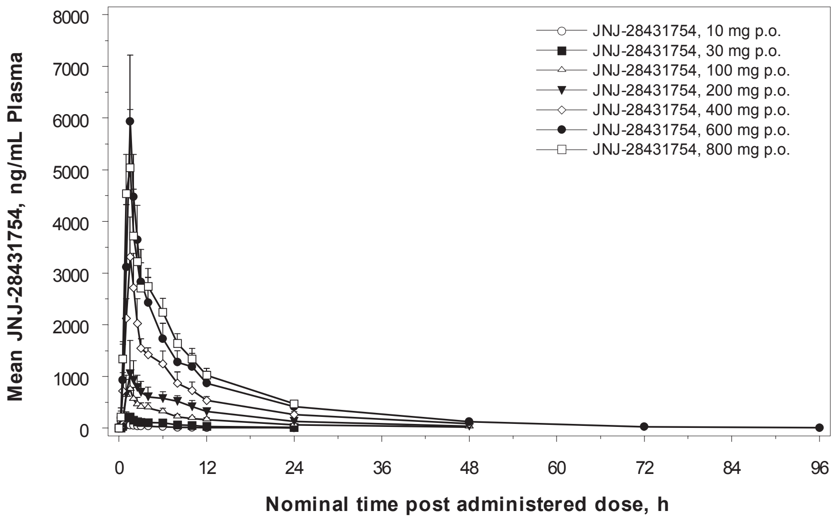

Figure 2: Mean plasma concentration-time profiles of JNJ-2841754 in healthy normal male subjects following a single oral dose of JNJ-2841754-logarithmic scale (preliminary results, Protocol JNJ-2841754 NAP1001) (Note: only a 24-hour profile included for the 800 mg dose group)

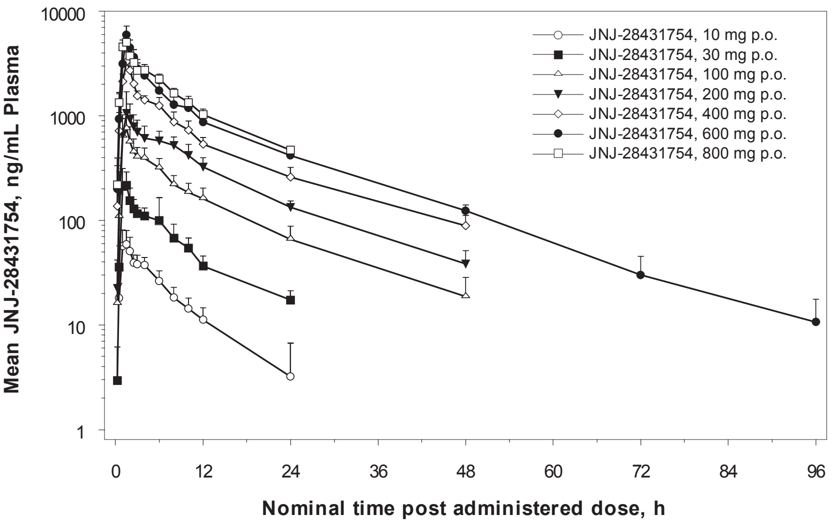

Table 2: Mean (SD) plasma JNJ-28431754 pharmacokinetic parameters in healthy normal male subjects following a single oral dose of JNJ-2841754 (Preliminary Results, Protocol 28431754-NAP-1001)

Dose	N	$AUC_{0\text{-}24\ h}$	AUC_{∞}	C_{max}	t_{max}[a]	$t_{1/2}$	CL/F	Vd/F
(mg)		ng.h/mL	ng.h/mL	ng/mL	h	h	L/h	L
10	6	408 (70.0)	471 (118)	67.5 (24.9)	1.50 (1.00-2.00)	8.39 (4.20)	22.4 (5.59)	245 (84.3)
30	6	1428 (188)	1685 (298)	254 (49.9)	1.25 (1.00-6.00)	9.52 (1.99)	18.2 (2.99)	245 (30.7)
100	6	5094 (1169)	6426 (1772)	755 (313)	1.50 (1.50-4.00)	11.3 (1.34)	16.4 (3.54)	263 (40.1)
200	6	9315 (707)	12052 (1065)	1164 (523)	1.75 (1.50-8.00)	11.7 (1.70)	16.7 (1.50)	280 (32.1)
400	6	19702 (2439)	25665 (3862)	3322 (759)	1.50 (1.00-1.50)	13.6 (1.49)	15.9 (2.38)	310 (37.4)
600	6	31771 (6500)	40639 (6204)	5935 (1288)	1.50 (1.50-1.50)	13.2 (3.21)	15.1 (2.62)	297 (129)
800	6	35643 (3.10)	41630 (4.10)	5210 (20.8)	1.50 (1.00-1.50)	8.66 (10.5)	19.2 (4.10)	240 (8.30)

[a]Data presented as Median (Min-Max)

Pharmacodynamics (PD)

Preliminary PD results summarized below are based on blinded data from Cohort 1 to Cohort 7 (10 mg to 800 mg JNJ-2841754). The PD parameters evaluated were the amount of urine glucose excretion (UGE), the rate of urine glucose excretion, plasma glucose concentrations, and plasma insulin concentrations.

UGE increased with higher doses, but in a less than dose-proportional (or plasma JNJ-2841754 AUC-proportional) manner. Also, the difference in the mean UGE over the first 24 hours post dose was minimal between the 400 and 800 mg doses, indicating that the UGE response in healthy male subjects may be maximal at or near the 400-800 mg dose level (Table 3; Note: As the mean UGE values in Table 3 include the placebo subjects, the reported UGE values are expected to be lower than that for the treated subjects).

Elevated urine glucose excretion occurred 2 to 7 hours after dosing at all dose levels. The maximal UGE rate was approximately 3.5 g/h at the 400 - 800 mg dose range. The mean total UGE values on Day 2 post dose (in 24-48-hr urine collection interval) became substantial over the dose range of 100 mg - 800 mg. At these dose levels, the UGE rates achieved were about 2-3 g/h over the first 13 hours post dose and about 0.5-2 g/h over 13 to 48 hours post dose.

Table 3: Mean (SD) Amount Urine Glucose Excreted (UGE) For All Subjects
(Preliminary results: Protocol JNJ-28431754-NAP-1001)

Dose	n		Urine Glucose Excreted (g)	
			0-24 hrs	24-48 hrs
10 mg/Placebo	8	Mean	6.16	0.532
		(SD)	(4.40)	(1.31)
30 mg/Placebo	8	Mean	13.1	0.648
		(SD)	(10.2)	(0.990)
100 mg/Placebo	8	Mean	32.7	9.20
		(SD)	(21.0_)	(6.49)
200 mg/Placebo	8	Mean	36.5	14.5
		(SD)	(23.7)	(11.7)
400 mg/Placebo	8	Mean	47.9	30.0
		(SD)	(31.5)	(21.4)
600 mg/Placebo	8	Mean	51.9	35.9
		(SD)	(33.6)	(24.5)
800 mg/Placebo	7	Mean	56.1	49.8
		(SD)	(27.3)	(26.5)

In Part 1 of this single ascending dose study, blood samples for assessment of 24-hour plasma glucose and insulin profiles were obtained at predefined time points over 24 hours post dose. Mean postprandial plasma glucose excursions appeared to decrease in a dose dependent manner after a single oral administration of JNJ-28431754, particularly during the morning meal.

Mean postprandial plasma insulin levels also decreased in a dose dependent manner after a single oral administration of JNJ-28431754.

No hypoglycemia was observed in any subject at all dose levels. The absence of hypoglycemia is consistent with the anticipated mechanism of pharmacological action for this drug, ie, plasma glucose is not expected to fall below the renal glucose threshold in healthy or diabetic subjects

1.2. Overall Rationale for the Study

This will be the first multiple dose study of JNJ-28431754, a potent, orally available, selective and reversible inhibitor of the renal sodium glucose co-transporter (SGLT2) in subjects with Type 2 diabetes mellitus (T2DM).

Results from the ascending dose study in healthy men indicated that single oral doses of JNJ-28431754 up to 800 mg have been generally well tolerated, as summarized in Section 1.1 of this protocol. There were no serious adverse events, no subjects were discontinued from the study, and there were no clinically significant changes or treatment-related trends in clinical laboratory values, physical examinations, vital signs, or ECG parameters. Those observations support further evaluation of JNJ-28431754 in T2DM subjects. Type 2 diabetes affects both men and women, and thus evaluating the safety, tolerability and effects of multiple JNJ-28431754 dosing in women as well as in men, is warranted to support further clinical development of this new agent. The data from this study will be used to design and guide dose selection for future studies of JNJ-28431754 in T2DM subjects involving treatment durations greater than 14 days.

2. OBJECTIVES

The overall objectives are to evaluate the safety, tolerability, pharmacokinetics (PK) and pharmacodynamics (PD) of JNJ-28431754 after single and multiple ascending oral doses of JNJ-28431754 in subjects with Type 2 Diabetes Mellitus (T2DM). It is hypothesized that daily oral administration of JNJ-28431754 for 2 weeks, at safe and well-tolerated dose levels, will lower mean plasma glucose concentrations ($AUC_{0\text{-}24hr\text{-}glucose}$) in diabetic patients to a clinically meaningful extent, compared to baseline and/or to placebo administration.

3. OVERVIEW OF STUDY DESIGN

Five (5) Cohorts, total of 100 subjects, 20 in each cohort, will participate in this study, with potential for an additional cohort of 20 subjects.

A blood sample will be collected on Day 1 from subjects consenting to the pharmacogenomic component of the study. This will allow for pharmacogenomic analysis, as necessary. Subject participation in pharmacogenomic research is optional and requires separate informed consent. Refusal to consent for this component does not exclude a subject from participation in the clinical study.

3.1. Study Design

This is a randomized, double-blind, placebo-controlled, single and multiple (14 days) ascending dose, parallel group study. Five cohorts of subjects with T2DM (20 subjects per cohort) will be studied. One dose level will be evaluated in each cohort. The study will be conducted at multiple sites.

Sixteen (16) subjects will be randomized to JNJ-28431754 and 4 subjects to matching placebo within each cohort. Doses planned for evaluation are 30, 100, 300 and 600 mg/day. An additional cohort of Asian subjects will also be evaluated at a dose level, which was previously tested in a prior cohort and considered to be well tolerated. Following initial screening (conducted within 35 days prior to Day -3 of the study), eligible subjects will be instructed to withdraw from their previous antidiabetic medications for 16 days prior to dosing on Day 1. Blood glucose levels will be monitored daily during the 16-day washout period. Eligible subjects will be admitted to the Clinical Research Unit (CRU) on Day – 3. On Days –2 and -1, subjects will receive placebo once daily in a single-blind fashion (subjects blinded) and undergo baseline safety and PD assessments. At least fifty percent of subjects in each cohort should be targeted to have fasting blood glucose ≥170 mg/dL (9.45 mM) and a MDRD calculated GFR ≥ 80 (mL/min/1.73 m^2). Eligible subjects will then be randomized to a double-blind treatment with JNJ-28431754 or placebo. A single dose of study medication will be administered on Day 1, followed by periodic safety, PK and PD assessments. Daily dosing will resume on Day 3 and continue through Day 16 (14 days of dosing). Each daily dose will be administered at approximately 8 AM followed by a standardized breakfast within 10 minutes after dosing. Standardized lunch and dinner will be provided at 4.5 and 10.5 hours post

dose, respectively. Subjects will be discharged from the CRU on Day 20 (96 hours post last dosing) and return to the CRU for safety assessments and PK sample collections on the mornings of Days 21 to 22. Subjects will also return within 7-10 days following the Day 22 outpatient visit for a final Follow-up visit.

Subjects must have been diagnosed with Type 2 diabetes at least 12 months prior to study start and will have been stably managed for at least 3 months prior to the screening visit on oral antidiabetic (metformin, or a sulfonylurea, or a meglitinide (e.g., repaglininide or nateglinide), or a DPP-IV inhibitor (e.g., sitagliptin or vildagliptin), or alpha-glucosidase inhibitor (e.g., acarbose) or a combination of two anti-diabetic medications. Subjects will not have used any of the following anti-diabetic medications within 3 months of study start: exenatide, thiazolidinediones (e.g., rosiglitazone, or pioglitazone), or insulin.

Eligible subjects for this study will return for an outpatient visit around Day -17, to be counseled by a registered dietician to follow a standard weight maintaining diet for about 2 weeks prior to admission and throughout the entire study except on Days –3 to 20 when subjects will receive standardized meals (See Attachment 8) (The weight maintaining diet will follow standard recommendations of the American Diabetes Association, comprising ~55-60% carbohydrate, 10-20% protein, and ~30% fat, with total daily caloric intake individually adjusted for BMI). In addition, subjects will receive instruction in signs and symptoms of hypoglycemia, use of a portable home fingerstick blood glucose analyzer and reporting of results. During the 16-daywashout phase and during the post-treatment phase (between discharge and prior to the Follow-up visit), subjects will record their fasting blood glucose levels and the postprandial blood glucose levels after lunch on a diary card which will be provided to them and report the results to the clinic weekly, or at any time values exceed prespecified limits. This information will be collected by the clinic and recorded in the eCRF.

Subjects will resume their pre-study oral anti-diabetic medication regimen upon completion of the outpatient visit on Day 22 or potentially at any earlier time during the study if their confirmed fasting blood glucose levels rise above 15 mM (270 mg/dL) or postprandial glucose levels rise above 22.2 mM (400 mg/dL) following lunch. Subjects who need to restart anti-

diabetic medications during the study will stop receiving study medication but continue with study related safety procedures and daily PK samples (relative to predose time point only) for at least 3 days after the final dose. The Principal Investigator will determine the dose at which to reinitiate the subjects' medications and any subsequent dose adjustments.

The escalation of doses defined in this protocol may be modified if indicated based on evaluation of the safety, tolerability, pharmacokinetics or pharmacodynamics of prior cohorts. Modifications of any next dose can be a de-escalation, a reduced escalation step, or an increased escalation step. Twice-daily dosing may also be evaluated in one or more of the cohorts or an additional cohort of 20 subjects may be added. For any dose chosen for a possible twice-daily dosing regimen, the maximum predicted total plasma JNJ-28431754 exposure ($AUC_{0\text{-}24h}$ at steady-state) will not exceed the NOAEL established in the 2-week GLP toxicology study in rats ($AUC_{0\text{-}24h}$: ~ 80 μg.h/mL) based on the incidence of hyperostosis (minimal to mild changes in distal end of femur & proximal end tibia) at 150 mg/kg, without an amendment to this protocol. Pharmacokinetic and pharmacodynamic sampling time points may be adjusted to accommodate a twice-daily dosing regimen but, the total blood volumes specified in the protocol will not be exceeded. For all cohorts, JNJ-28431754 dose levels that are predicted to produce steady state JNJ-28431754 exposures above the NOAEL in the 2-week GLP toxicology study in rats ($AUC_{0\text{-}24h}$: ~ 80 μg.h/mL) will not be exceeded without an amendment to this protocol.

Planned JNJ-28431754 Dose Levels					
Cohort[1]	N				

1	16+4[2]	30 mg or placebo			
2	16+4[2]		100 mg or placebo[3]		
3	16+4[2]			300 mg or placebo[3]	
4	16+4[2]				600 mg or placebo[3]
5	16+4[2]				30, 100 or 300 mg or placebo[4]

1 Initiation of dosing for each subsequent cohort at a higher dose level will be at least 17 days following the initiation of dosing in the prior cohort.

2 Sixteen on JNJ-28431754; four on placebo

3 The actual dose (mg) level selected for each cohort may be modified based on evaluation of preliminary safety, pharmacokinetic and pharmacodynamic data from previous cohorts.

4 A fifth cohort will assess the safety, tolerability and effects of multiple doses of JNJ-28431754 on male and female Asian subjects at a dose level which was previously tested in an earlier cohort and considered to be well tolerated

Successive cohorts at a higher dose level will be started with at least a 17-day interval relative to the start of dosing in the preceding cohort, after review of adverse events, vital signs, electrocardiograms (ECGs) and laboratory tests of the preceding cohort collected up to and including Day 17 for preliminary safety data and Day 11 for PK data. Each subsequent escalated dose level shall be performed if, in the judgement of the Investigator and the J&JPRD Safety Physician, the results of the safety analysis of the preceding dose level are satisfactory. If a single dose at a given dose level is not well tolerated by individual subjects, the single dose may be repeated at a lower dose level, or the multiple dosing may start at a lower dose for those subjects or the entire cohort, in the judgement of the Investigator and the J&JPRD Safety Physician. At least 10 subjects are required to complete each treatment period prior to a decision to escalate to the next higher dose level. If fewer than 10 subjects complete any cohort, a separate Data Review Committee (DRC) may be convened to review unblinded data and to provide a recommendation regarding dose escalation.

3.2. Study Design Rationale

This is the first single and multiple dose study with JNJ-28431754 in Type 2 diabetic subjects.

This will be a double-blind study in order to avoid subjective bias in the assessment of safety/tolerability and pharmacological effects of the study drug. Placebo will be administered as a control in order to establish the

frequency and/or magnitude of changes in clinical endpoints that may occur in the absence of active drug treatment.

Based on the mechanism of action of an SGLT-2 inhibitor, the effects of JNJ-28431754 on urinary glucose excretion and plasma glucose concentration are expected to be greater in hyperglycemic diabetic subjects as compared in healthy, euglycemic subjects. Enrolling diabetic subjects in this study allows early assessment of safety and efficacy of JNJ-28431754 in the intended target population under safe and well-controlled condition, and thus will guide subsequent clinical development.

Type 2 diabetes affects both men and women, and thus evaluating the safety, tolerability and effects of multiple JNJ-28431754 dosing in women as well as in men, is warranted to support further clinical development of this new agent. In the present multiple dose study, since reproductive toxicology studies have not yet been completed, only women of non-childbearing potential (post-menopausal, or surgically sterile) are to be enrolled initially. That restriction will limit the number of female Type 2 diabetics who would otherwise be eligible to participate in this study. In the 2-week GLP toxicology studies in both rats and dogs, systemic exposure of JNJ-28431754 was generally higher in females (up to 2 fold) than in males. Female subjects of non-child bearing potential will be enrolled in this study to allow exploring potential differences in safety and PK between males and females.

Although subjects of Asian ethnicity have not been explicitly enlisted for the current protocol, based on the likely study demographics, relatively few Asian subjects are likely to be enrolled in each cohort. Since the incidence of T2DM is rapidly increasing in Asia, this new drug may be developed for use in Asian countries. To support potentially larger and longer clinical trials in Asian diabetics, an additional cohort will be studied that is comprised exclusively of Asian diabetics to allow exploring safety and efficacy in this population. Based on preclinical data related to JNJ-28431754 metabolism and clearance mechanisms, the PK and metabolism profiles of JNJ-28431754 in Asian subjects are not expected to be markedly different from that in Caucasians or other ethnic groups.

The dose selected for this additional Asian cohort will be a dose that has been already evaluated in a prior cohort as a safe and potentially efficacious dose.

Since this is the first study of JNJ-28431754 in Type 2 diabetic subjects, subjects in each cohort will first receive a single dose of JNJ-28431754 followed by frequent and carefully monitored safety assessments for 2 days, prior to initiating the 14-day multiple dosing period on Day 3.

The planned dose range for this study is 30, 100, 300 and 600 mg/day of JNJ-28431754. The starting dose is based on safety, tolerability and PK and PD data from the ongoing SAD study (Protocol JNJ-28431754 NAP-1001) in healthy male subjects. In that study, with JNJ-28431754 doses of 10 mg up to 800 mg, the mean urine glucose excretion (UGE) over 24 hours post dose (UGE/24h) increased less than dose proportionally, from 6 g to 56 g (including placebo subjects). At all dose levels, the increased UGE was not associated with clinically significant abnormalities in fluid or electrolyte balance. No hypoglycemia was observed at any dose level. Systemic plasma drug exposure following single doses of JNJ-28431754 increased dose proportionally as doses increased from 10 to 600 mg ($AUC_{0\text{-}inf}$: 0.5 to 41 μg.h/mL at doses 10 to 600 mg). The terminal half-life ranged from 8 to 14 hours. Based on the observed single dose PK parameters, no major drug accumulation (less than 2 fold) is anticipated following repeated QD administration of JNJ-28431754. Thus, 30 mg is considered a safe starting dose for the present multiple dose study. The selected dose range of 30 to 600 mg is expected to cover a potentially efficacious dose range with respect to increases in UGE and decreases in plasma glucose concentration (sufficient for expecting HbA1c reduction of at least 0.8% in longer duration trials). The predicted mean exposure ($AUC_{0\text{-}24h}$ at steady state) at the maximum dose for this study will not exceed the mean plasma exposure at the NOAEL dose (20 mg/kg) in the 2-week GLP toxicology study in rats ($AUC_{0\text{-}24h}$: ~ 80 μg.h/mL), an exposure at which no dose limiting organ toxicity was observed (mildly higher AST, BUN, minimally lower serum calcium and mildly to moderately lower blood glucose).

Preliminary data from the single ascending oral dose study in healthy normal men (Protocol JNJ-28431754NAP1001) suggests that at dosages of 400 mg and higher, JNJ-28431754 may have a greater effect than expected to lower prandial glucose excursions (potentially by slowing the rate of gastrointestinal glucose absorption). Therefore, a BID regimen, with dose levels of 400 mg, given with the morning and evening meals, might have superior glucose lowering efficacy as compared to a QD regimen. To

characterize this effect in diabetic subjects, a BID dose regimen may be studied in the present multiple dose study, at the high end of the planned dose range, assuming the planned QD dose levels are well tolerated.

Superficial and small (~1 mm diameter) gastric erosions had been observed in some animals in a 2-week rat toxicology study. The first-in-human single ascending oral dose Phase I trial (JNJ-28431754 NAP-1001) therefore had fecal occult blood tests (FOBTs) performed daily on Days 1-4 post-dose. Subsequent investigative rat toxicology studies indicate that the histologically very acute gastric erosions noted in rats are attributable to acute stresses induced by certain study procedures conducted just prior to necropsy. Those rat erosions are therefore no longer considered as primary, direct effects of JNJ-2843175. On that basis, and also since in the single ascending oral dose study (JNJ-28431754 NAP-1001) all FOBTs at every dose level have been negative, FOBTs are not included in the present trial. Nonetheless, all T2DM subjects in this study will be closely monitored for signs and symptoms of adverse gastrointestinal effects. Preliminary results of a currently ongoing 3-month rat toxicology study with JNJ-28431754 (using modified pre-terminal necropsy procedures) are anticipated to become available during early stage of the present study. Any unexpected results from that 3-month rat toxicology study that are potentially related to adverse GI effects will be evaluated in relation to the clinical monitoring procedures in the present multiple-dose study.

At the highest planned dose of 600 mg/day in this multiple-dose study, mean plasma JNJ-28431754 exposures at steady-state are expected to approximate the JNJ-28431754 exposures attained at the highest tested, 800 mg dose (which was evaluated as being well-tolerated) in the single ascending oral dose study (JNJ-28431754 NAP-1001). Recently, in an ongoing 3-month dog toxicology study, during the first week at the highest tested dose of 200 mg/kg/day only, 3 of 5 male dogs in each gender had blood in stools. This adverse GI effect resolved upon dose reduction to 100 mg/kg/day. No abnormal microscopic or gross pathological findings were observed in the previous 2-week GLP toxicology study in dogs, even at daily doses up to 400 mg/kg/day. In the ongoing 3-month GLP toxicology study in dogs, the mean plasma JNJ-28431754 steady-state exposures at 100 mg/kg/day are conservatively expected to be in the range of approximately 264,000-445,000 ng.hr/mL and 32,000-40,000 ng/mL, for AUC_{0-24} and $C_{max,}$

respectively. Compared to those plasma exposures in dogs, human plasma JNJ-28431754 exposures in the last cohort at the highest escalated dose of 600 mg/day in this study are expected to substantially lower, by about 6- to 11-fold (for AUC_{0-24}) and about 5- to 7-fold (for C_{max}). Nonetheless, at all dose levels in this study, subjects will be closely monitored for signs and symptoms of adverse GI effects. Preliminary results from the ongoing 3-month toxicology study in dogs are scheduled to become available during early stage of the present study, and any unexpected results from that 3-month dog toxicology study which are potentially related to adverse GI effects will also be evaluated in relation to the clinical monitoring procedures in the present multiple-dose study.

Diabetic subjects who participate in this study will temporarily discontinue their prior anti-diabetic medication(s) in order to clearly demonstrate the urine glucose excretion and blood glucose lowering effects of monotherapy with JNJ-28431754. In addition discontinuing their prior anti-diabetic medications will minimize potential drug-drug interactions that have not been investigated. A 16-day washout period preceding dosing is adequate to allow washout of the drug exposures and pharmacological effects of the piror anti-diabetic medication(s) of the eligible subjects. This washout period also allows subjects to establish modestly elevated and stabilized fasting blood glucose levels (7.8 to 15 mM or 140 to 270 mg/mL) such that effect of JNJ-28431754 on plasma glucose concentration can be adequately determined. Subjects will be closely monitored for their blood glucose levels throughout the study, including during the 16-day washout period. Any subject will be discontinued from the study and be treated by the investigator with appropriate hypoglycemic agents if his/her confirmed (by conventional venous sample) fasting blood glucose level is above 15 mM (270 mg/dL), or postprandial glucose level rises above 22.2 mM (400 mg/dL) following lunch. It will be possible to rapidly resume anti-diabetic therapy. While chronically elevated (for years) and/or uncontrolled hyperglycemia in T2DM patients can lead to serious diabetic complications, short-term discontinuation of oral anti-diabetic medications (for a maximum of approximately 6 weeks in this study) with modest blood glucose elevations and with daily medical monitoring is not expected to result in clinically meaningful short- or long-term consequences for the study subjects.

Because SGLT-2 inhibitors cause the net loss of calories from the body in the form of glucose, these compounds also have potential to reduce body weight. However, the increase in urinary glucose excretion might lead to a compensatory increase in food intake. In this study, food intake cannot be directly measured, as subjects will receive standard meals to ensure reliable determination of drug effect on the 24-hour plasma glucose profile, the primary end point. Visual Analogue Scales (VAS) are commonly used to assess appetite and satiety sensations, which correlate well with food intake. In this study, the VAS will be assessed at baseline and post dose at predefined times using a validated study procedure.[12]

Based on available data from preclinical JNJ-28431754 toxicology studies in rats and dogs, potential adverse human effects may occur. These include, but are not limited to, osmotic diuresis due to increased urinary glucose excretion, changes in serum or urine electrolytes, GI intolerability, hypoglycemia, changes in bone formation and or in bone resorption, abnormalities in hepatic and renal functions, and phototoxicity. Subjects will be closely monitored during study using well-established clinical procedures and laboratory tests, including monitoring of daily fluid intake and urine volume, serum and urine electrolytes for monitoring body fluid and electrolyte imbalance, self-monitoring using a glucometer as well laboratory assessment of plasma glucose levels to monitor potential hypo-/hyperglycemia, serum and urine biomarkers for monitoring bone turnover and protection from sunlight exposure during the study.

3.2.1. Rationale for DNA Collection

It is recognized that genetic variation within the population can be an important contributory factor to inter-individual differences in drug distribution and response and can also serve as a marker for disease susceptibility and prognosis. An association between a genetic polymorphism and clinical outcome may help to explain inter-individual variability in that outcome and may help to identify population subgroups that respond differently to a drug. The overall goal of the pharmacogenomic component is to allow for the identification of genetic factors that may influence pharmacokinetics, pharmacodynamics or safety/tolerability of JNJ-28431754. DNA samples will be collected in this clinical study in order to help address emerging clinical issues and to enable the development of

safer, more effective, and ultimately individualized therapies in the future. Further details are provided in Section 9.4.

3.3. Subject Safety and Stopping Criteria

After each cohort, the decision to continue with the next higher dose will be made jointly by the Principal Investigator and the Sponsor after review of all available blinded clinical safety and tolerability data, and pharmacokinetic and pharmacodynamic data. Blinded interim safety and pharmacokinetic data will be provided to the J&JPRD Safety Physician or designee prior to the planned dose escalation meeting with the Principal Investigator. Dose escalation decisions will take into account the incidence, severity and duration of all adverse events reported in prior cohorts.

This study will enroll diabetic subjects who will discontinue temporarily their current oral anti-diabetic medications in order to investigate the blood glucose lowering effects of JNJ-28431754. A 16-day washout preceding dosing in this study is adequate to allow washout of the plasma concentrations and pharmacological effects of sulfonylurea, meglitinide, metformin and DPP-IV inhibitor treatments, and to establish relatively stable blood glucose levels in these subjects, prior to Day 1. During that 16-day washout period, subjects will be trained to measure their fasting and postprandial blood glucose levels after lunch daily using a standard glucometer, and report those values to the study center for evaluation by the Principal Investigator, to ensure that no subject develops excessively high fasting blood glucose levels above 15 mM (270 mg/dL) and high postprandial values after lunch above 400 mg/dL during the washout period. Eligible study subjects will maintain a daily record of their fasting and prandial blood glucose levels during the pre-study washout period and the post-treatment phase. This information will be collected by the clinic and recorded. On Day –2 of this study, the fasting blood glucose levels in each subject must be within a well-tolerated hyperglycemic range of 7.8 – 15 mM (140-270 mg/dL) to continue in the study. Subjects will continue to measure their fasting (morning) and postprandial (post lunch) blood glucose levels throughout the study (during inpatient and outpatient phases), and any subject with a confirmed fasting blood glucose elevated above 15 mM (270 mg/dL) or postprandial glucose levels above 22.2 mM (400 mg/dL) following lunch will not be further administered study medication, and will

be treated with appropriate hypoglycemic therapy and monitored until blood glucose levels have stabilized.

In addition, if > 50 percent of subjects in any cohort experience an adverse event classified as moderate or severe by the Principal Investigator or one subject experiences a serious adverse event where the relationship to the study drug can not be excluded by the Principal Investigator, further dose escalation will not occur.

The sponsor's Safety Physician may request an internal Data Review Committee (DRC) to review unblinded data to decide the next dose or whether to stop or continue the study. This committee, with membership independent of the principal investigator, the safety physician and other members of the study team, will indicate their decision based on decision rules agreed upon prior to unblinding. The confidentiality of any unblinded information will be maintained until the study is terminated and the database is locked.

4. STUDY POPULATION

4.1. General Considerations

The specific inclusion and exclusion criteria for enrolling subjects in this study are described in the following sections.

4.2. Inclusion Criteria

Subjects must satisfy the following criteria to be enrolled in the study:

1. T2DM subjects who have been diagnosed for at least 12 months prior to study screening
2. T2DM subjects who have been taking a stable dose regimen of **oral** antidiabetic **monotherapy or a combination of two anti-diabetic medications** except exenatide and thiazolidinedione (TZD) for at least 3 months prior to study screening.
3. Males or postmenopausal/surgically sterile females, age 25 to 65 years inclusive

[Note: (1) Post-menopausal is defined as no menses for at least 18 months prior to study start or no menses for 6 to 18 months prior to the start of this study, and plasma FSH must be ≥ 40 mlU/mL and estradiol ≤ 20 pg/mL in 3 separate measurements which will occur at screening, Day -17, and Day -2 (a -2 day window is allowed at Day -2); (2) Pre-menopausal surgically sterilized subjects must have a negative β-HCG pregnancy test at screening and at Day –2.

4. At screening: body mass index (BMI = weight in kg/height in m^2) of 20 to 39.9 kg/m^2; HbA1c of ≥ 7% and ≤ 10%
5. On Day –2, fasting blood glucose (FBG) concentrations between 7.8 mM (140 mg/dL) and 15.0 mM (270 mg/dL)
6. Subjects who have not smoked more than 10 cigarettes per day in the last 6 months prior to screening.
7. Subjects may enter who have well-controlled hypertension, but they must be on a stable regimen of allowed anti-hypertensive medications (see Section 8, and Attachment 10) for at least 3 months prior to screening.
8. At screening and Day -2: a) systolic BP of ≥95 and ≤ 160 mm Hg, supine, b) diastolic BP ≥ 50 and ≤ 100 mm Hg, supine, c) HR ≥ 50 beats per minute, supine, d) pulse rate increase of ≤ 30 beats per minute and a decrease of systolic BP of ≤ 20 mm Hg when standing from a supine position.
9. Subjects may enter with dyslipidemias, but they must be on a stable regimen of allowed dyslipidemic medications (see Attachment 10) for at least 3 months prior to screening.
10. Competency in speaking and comprehending the languages where the study will be conducted.
11. Willing to adhere to the prohibitions and restrictions specified in this protocol (Section 4.4).
12. Subjects must have signed an informed consent document indicating that they understand the purpose and the procedures of the study and are willing to participate in the study.
13. To participate in the optional pharmacogenomic component of this study, subjects must have signed the informed consent form for pharmacogenomic research indicating willingness to participate in the pharmacogenomic component of the study (where local regulations permit). Refusal to consent for this component does not exclude a subject from participation in the clinical study.

4.3. Exclusion Criteria

Potential subjects who meet any of the following criteria will be excluded from participating in the study:

1. Subjects who have a history of Type 1, “brittle” diabetes or secondary forms of diabetes
2. Subjects who have a known history of repeated severe hypoglycemic episodes as defined in Attachment 7
3. History of clinically significant diabetic complications, including: retinopathy, nephropathy, neuropathy, gastroparesis, or ketoacidosis

4. History of, or currently active, significant illness including (but not limited to) cardiovascular disease (including cardiac arrhythmias, myocardial infarction, stroke, peripheral vascular disease), hematological disease, respiratory disease, hepatic or gastrointestinal disease, endocrine/metabolic disorders (excluding Type 2 diabetes), neurologic or psychiatric disease, malignant neoplasms (other than adequately treated cutaneous basal or squamous cell cancers), or any other illness that the Investigator considers should exclude the subject
5. History of having taken insulin, TZDs, exenatide, thiazide diuretics, or beta-blockers, within 3 months of the screening visit
6. History of having taken oral steroids within 3 months of the screening visit, or anticipates a need to take oral steroids during the course of the study
7. History of having chronically taken aspirin (greater than 100 mg/day), NSAIDs, anticoagulants or other drugs known to interfere with blood clotting within 3 months of study start, or anticipates a need to take any of these during the course of the study
8. History of recent major surgery (within 6 months)
9. History or currently active renal diseases, nephrolithiasis, upper or lower urinary tract infections or prostatitis
10. History, or family history, of bleeding or coagulation disorders
11. History of gastritis and GI ulcer and resultant complications, e.g. GI bleeding, perforation and obstruction.
12. History of eating disorder (e.g. anorexia, bulimia) or recent significant changes in body weight due to dieting or nutritional treatment
13. Clinically significant abnormality in physical examination, vital signs, or 12-lead electrocardiogram (ECG) at screening or Day -1.
14. Clinically significant abnormal values for hematology, coagulation, fasting clinical chemistry or urinalysis (elevated HbA1c, fasting blood glucose values or mild abnormality in lipid profiles are allowed for diabetic subjects) at screening or Day -2 (Note: Subjects who have fasting triglyceride levels greater than 5.1 mmol/L are excluded)
15. History of disorders that are potential causes of GI bleeding including, but not limited to:
    - GI mass lesions, obstruction, perforation and diverticula
    - Inflammation (e.g., oesophagitis, gastritis, ulcer at any site of GI tract, ulcerative colitis and Crohn's disease)
    - Vascular disorders (vascular ectasia at any site of GI tract, e.g., internal hemorrhoids, portal hypertensive gastropathy or colonopathy, varices at any site of GI tract, haemangioma, GI vascular malformations)

- Chronic GI infections (e.g., worm infestations, tuberculous enterocolitis, amoebiasis
- Surreptitious bleeding (e.g., recurrent or chronic hemoptysis, oropharyngeal bleeding, or epistaxis)

16. Currently active, clinically significant skin disorders
17. History of hypersensitivity to sunlight or artificial source of intense light, especially UV light
18. History of recent travel (within 6 months) to locations that may predispose to the acquisition of communicable illnesses (e.g., parasitic or water-borne illnesses in developing tropical regions)
19. Tested positive for serology: hepatitis B surface antigen (HBsAg), hepatitis C antibodies (anti-HCV) or human immunodeficiency virus (HIV) antibodies.
20. Recent history of alcohol or drug abuse within 6 months prior to screening
21. History of or currently positive for alcohol and/or drugs of abuse (including barbiturates, opiates, cocaine, cannabinoids, amphetamines and benzodiazepines) at screening and Day -2
22. Clinically significant acute illness within 14 days prior to study drug administration
23. History of drug and/or food allergies, including allergies or intolerance
24. Donation of 1 or more units (approximately 450 mL) of blood or acute loss of an equivalent amount of blood within 90 days prior to study drug administration
25. Participated in any research study within the past 4 weeks or received an experimental drug or used an experimental medical device within 90 days prior to study drug administration
26. Use of any prescription or over-the-counter medications (including herbal medications, homeopathic remedies, or vitamins or mineral supplements (other than once-daily multivitamins approved by the PI) within 14 days prior to study drug administration [not including allowed concomitant medications or occasional use of paracetamol/acetaminophen (Section 8, Attachment 10)
27. Male subjects who are not sterile or not willing to abstain from sexual intercourse for the duration of the study (and until 90 days after the last dose of study medication), ensure that their partner practices a highly effective method of birth control such as implants, injectables, combined oral contraceptives, or hormonal IUDs (intrauterine devices) according to Note for Guidance on Non-Clinical Safety Studies for the Conduct of Human Trials for Pharmaceuticals (CPMP/ICH/286/95, modification).

28. Any condition that in the opinion of the Principal Investigator would complicate or compromise the study, or the well being of the subject

4.4. Prohibitions and Restrictions

Potential subjects must be willing to adhere to the following prohibitions and restrictions during the course of the study to be eligible for participation.

1. Weight maintaining diet must be adhered to from 2 weeks prior to admission (Day –3) and throughout the study as instructed by a registered dietician designated by the Principal Investigator. Willing to consume entirely the 3 same standard meals provided on Days –1, 1, and 16.
2. Strenuous exercise (e.g., long distance running ≥5 km/day, weight lifting, or any physical activity to which the subject is not accustomed) is to be avoided throughout the study, from screening through the follow-up visit
3. Alcohol consumption must be avoided for at least 72 hours prior to study drug administration until the final Follow-up visit.
4. Methylxanthine containing products (e.g., coffee, cola, tea, chocolate) and quinine-containing products (e.g., tonic water) are not permitted from 48 hours prior to study drug administration until at least 48 hours following the last administration of study drug.
5. Grapefruit, grapefruit juice, cranberry juice, Seville oranges, St John's wort, phlorizin, charcoal broiled foods, and any medication with CYP3A4 inhibition or induction properties (to allow a sufficient wash out/recovery period, particularly if the medication is a CYP3A4 mechanism-based inhibitor such as some of the macrolide antibiotics [e.g. erythromycin]; see Attachment 9) must not be consumed from 14 days prior to study drug administration, or while confined to the clinical study center and continuing to the Follow-up visit.
6. Subjects will be instructed to abstain from poppy seed consumption at least 72 hours prior to the screening visit and prior to admission to the Unit.
7. Subjects must be advised not to donate blood for at least 90 days after completion of the study.
8. There is no information about the effect of JNJ-28431754 on sperm or its production in the body; nor is there information about any possible effects on the development of the fetus. It is important that male subjects' partners should not become pregnant during the study. As a precaution, male subjects should use appropriate contraception (e.g. condoms) during the time interval between taking the first dose and 3 months after taking the last dose of study medication. Subjects

will be advised that partners should also use highly effective contraceptives (i.e. those with less than a 1% failure rate per year) which include implants, injectables, combined oral contraceptives, hormonal IUDs, sexual abstinence or vasectomized partner) according to Note for Guidance (CPMP/ICH/286/95, modification) during this period. Subjects should inform the principal investigator or the study physician if their partner becomes pregnant during the study.

9. Subjects must be advised not to donate sperm during the time interval between taking the first dose and for at least 90 days after completion of the study.
10. While in the study center subjects will not be allowed additional sugar in beverages or on cereal, though limited use of other sweeteners is allowed.
11. Following administration of the first dose of the study medication, subjects will be advised to avoid direct exposure to sunlight or artificial sources of intense light, especially UV light, up to 96 hours post the last drug administration, or if sunlight exposure cannot be avoided, to use protective clothing and broad-spectrum (UVA/UVB) sunscreens and sun block lipsticks that have a skin protection factor (SPF) of at least 15.

5. RANDOMIZATION AND BLINDING

5.1. Overview

Randomization will be used to avoid bias in the assignment of subjects to treatment, to increase the likelihood that known and unknown subject attributes (e.g., demographic and baseline characteristics) are evenly balanced across treatment groups, and to enhance the validity of statistical comparisons across treatment groups. Blinded treatment will be used to reduce potential bias during data collection and evaluation of clinical endpoints.

5.2. Procedures

Prior to the study drug administration on the morning of Day 1 subjects will be assigned subject numbers as described below.

Cohort 1	1001 through 1020
Cohort 2	1021 through 1040
Cohort 3	1041 through 1060
Cohort 4	1061 through 1080
Cohort 5	1081 through 1100

Within each cohort (1 through 5) subjects will be randomly assigned to receive JNJ-28431754 (n=16) or placebo (n=4). It is preferable (but not

required) that an approximately equal number of subjects in each cohort will be included from each site. At least 2 additional subjects will be admitted on Day –3 and will undergo all assessments leading up to dosing to ensure that at least 20 subjects are dosed in each treatment cohort. Subjects who withdraw will be replaced at the discretion of the sponsor as soon as study logistics allow. Withdrawn subjects for whom the blind was broken will not be replaced. If subjects need to be replaced after dosing for any reason the replacement will be numbered with the prefix 2, whereby subject 2001 replaces 1001, etc.

At least 10 subjects are required to complete each cohort for the dose-escalation decision based on the preliminary blinded data, ensuring that in each cohort at least 1 subject would have received placebo. If less than 10 subjects complete a cohort and both the principal investigator (PI) and the Sponsor still wish to consider dose escalation, or a safety concern arises for a given cohort, an internal Data Review Committee (internal DRC; comprised of Sponsor staff separate from the Clinical Study Team) may be asked to review the unblinded clinical data. Details of the internal DRC decision-making process will be outlined in the DRC charter.

The computer generated randomization schedule will be prepared by J&JPRD prior to the study using randomized permuted blocks. A subject who withdraws from the study may be replaced at the discretion of the Sponsor.

Based on the randomization codes, the unblinded pharmacist or properly trained designee will prepare the study drug for the oral administration.

Only the pharmacist or properly trained designee will be unblinded to the randomization codes. With the exception of the above, the individual subject randomization schedule will not be revealed to study subjects, investigators and clinical staff, or the J&JPRD study team until all subjects have completed the double-blind phase of the study and the database has been finalized by J&JPRD. The investigator will receive a sealed envelope for each subject containing information on the study drug identification. These sealed envelopes will be kept together, in a limited access area that is accessible 24 hours per day. Under normal circumstances, the blind should not be broken. The blind should be broken only if specific emergency

treatment would be dictated by knowing the treatment status of the subject, or upon written agreement with the J&J PRD safety physician or designee. In such cases, the investigator must contact the sponsor. If the investigator is unable to contact the sponsor, the investigator may in an emergency determine the identity of the treatment by opening the sealed envelope. The sponsor must be informed as soon as possible. The date, time and reason for the unblinding must be documented on the appropriate page of the case report form (CRF) and in the source document. At the completion of dosing for each cohort, the study team may unblind the data on a cohort-by-cohort basis in order to evaluate the complete information to aid in decision making regarding future dosing. Unblinding at the completion of each cohort is warranted since the subjects are no longer participating in the study and a fully informed decision can be made regarding subsequent study conduct.

All randomization codes, whether opened or sealed, will be collected at the end of the study.

A second set of sealed randomization envelopes will be kept at the sponsor site in a safe and limited access area.

6. DOSAGE AND ADMINISTRATION

Subjects will be admitted to the CRU on Day –3. Following an overnight fast for at least 10 hours subjects will be administered a placebo dose on Days –2 and –1 and JNJ-28431754 or placebo on Day 1 and Days 3 to 16. The study medication will be administered in the sitting position between 8:00 to 9:00 am. The exact dosing time must be recorded in the CRFs. Study drug will be administered as a single oral dose via an oral liquid dispenser directly into subject's mouth by the investigator or the designated study personnel. Subjects will drink 240 mL of water immediately after dosing. Within 10 minutes after the study drug administration, subjects will receive a standardized breakfast. Standardized lunch and dinner will be provided at 4.5 and 10.5 hours, respectively, post-dose. Each meal should be completely finished within 30 minutes. Subjects will be encouraged to complete their meals. Incompletion of a meal should be recorded in the eCRFs. The three meals served on Days -1, 1 and 16 will be of the exact same composition on all days for all subjects. Water will be allowed ad libitum throughout the study. All fluid intake (i.e., volume), including that taken during meal times, will be recorded in the CRFs from Day-2 through discharge from the CRU

on Day 20. Subjects will receive standardized meals in the CRU starting Day –3 through Day 20. Detailed information on the standardized meals is provided in Attachment 8. Mealtime (start and end) in the CRU, starting from admission through Day 20, will be recorded in the CRF.

7. COMPLIANCE

The investigator or designated study personnel will maintain a log of all study drug dispensed and returned. Drug supplies will be inventoried and accounted throughout the trial. The investigator or designee will supervise administration of all study medication. The exact time of each drug administration for each subject will be recorded in the CRF.

8. CONCOMITANT THERAPY

The subjects are not allowed to take any prescription or non-prescription over-the-counter (including non-steroidal anti-inflammatory drugs) or herbal medications (including herbal tea), vitamins or mineral supplements or organic/nutritional supplements within 14 days prior to study drug administration and for the duration of the study. Exceptions to this will be, as specified in Attachments 8 and 9: 1) stable doses of once daily multivitamins if approved by the PI, 2) stable doses (at least 3 months) of anti-hypertensive agents, 3) drugs for treating dyslipidemia, 4) low dose aspirin (≤100 mg/day) and 5) occasional intake of paracetamol or acetaminophen (maximum 1 g every 6 hours; 4 g/day), which is permitted periodically throughout the study if approved by the Principal Investigator. Need for excessive or continued use of paracetamol or acetaminophen could lead to exclusion from the trial.

If the administration of any concomitant therapy becomes necessary (including allowed concomitant therapy), it must be reported in the appropriate section of the CRF. The sponsor must be notified in advance (or as soon as possible thereafter) of any instances in which prohibited therapies are administered

9. STUDY EVALUATIONS

9.1. Study Procedures

Study procedures must be performed according to this protocol. In the case of multiple assessments at a given time point the sequence of events should be: adverse event questioning, 12-lead ECG, blood pressure and pulse rate, body temperature, blood sampling for PK, PD, and clinical laboratory

testing. All procedures should be initiated early enough to ensure that the PK and PD blood samples are collected at the protocol-specified times.

9.1.1. Overview

The Time and Events Schedule included in the Synopsis summarizes the frequency and timing of safety, tolerability, PK, PD, or other measurements. Venous blood will be collected for all blood-based analysis. The exact dates and times of blood drawn and study drug administration must be recorded in the CRF. Attachments 1 through 6 provide further information regarding handling of biological samples.

Adverse events and concomitant medications must be recorded on a continuous basis, starting with signing of consent and ending with the last study procedure (Follow-up). During the treatment phase study staff will periodically ask the subjects “how are you feeling?” at the time points specified in the Time and Events Schedule. In the event subjects develop symptoms of hypoglycemia, blood glucose will be assessed with a glucose analyzer and the suggested treatments listed in Attachment 7 are to be implemented as considered appropriate by the investigator. If fasting blood glucose levels are confirmed to be above 15 mM or postprandial glucose levels above 22.2 mM (400 mg/dL) after lunch, the subject will be discontinued from study medication and treated with rescue medication.

The total volume of blood drawn for laboratory evaluations throughout this study is approximately 482 mL for each subject and 518 mL for those subjects participating in twice-daily dosing cohorts. A 10 mL blood sample will be collected from subjects who consent to pharmacogenomic research; this will be taken on Day 1 following study drug administration. In the event of DNA extraction failure, a replacement pharmacogenomic blood sample may be requested from the subject. Signed informed consent will be required to obtain a replacement sample. All blood samples will be collected by individual venipuncture. An indwelling catheter (kept open with a low-rate infusion of 0.9% saline) may be allowed on intensive blood sampling days.

**Parameter**	Vol. Per Sample (mL)	Total # of Samples	Total Volume (mL)
Serology	2	1	2
Endocrinology	2	3	6
Clinical Biochemistry	4	13	52
-serum creatinine	1	1	1
Hematology	2	13	26
Coagulation	2	2	4
Bone markers	1	13	13
-1,25-dihydroxy Vitamin D and PTH	1	7	7
Pharmacokinetics	3	38	114
-twice daily cohort	3	12	36
Pharmacodynamics			
-glucose, insulin, C-peptide	2	101	202
-GLP-1 active and total	3	15	45
Pharmacogenomics	10	1	10
**Total Blood Volume**			**482**
**-**twice daily cohort			**518**

9.1.2. Screening Phase

Subjects will report to the CRU for eligibility screening within 35 days prior to Day -3. Before any study specific procedures are conducted the subjects must read, understand (as judged by the Principal Investigator or designee) and sign a written Informed Consent Form. Adverse events and concomitant medication recording will start at the signing of informed consent and continue until the last study related procedure.

The following will be performed:

- Informed consent process
- Complete separate informed consent form for the optional pharmacogenomic research component of the study.
- Review inclusion/exclusion criteria

- Obtain relevant medical history and demography
- Complete physical examination including a thorough whole body skin assessment by a physician
- Measure height and body weight to determine BMI and BSA
- Measure supine vital signs (after 5 minutes in a supine position) including blood pressure and pulse rate.
- Body temperature
- 12-lead ECG
- Clinical laboratory tests including hematology, clinical chemistry, coagulation and urinary analysis using both dipstick and microscopic assessments. [Note: (1) subjects must have an overnight fast of at least 8 hours, (2) GFR calculated using the MDRD equation defined in Section 9.5.2. for details of the listed tests]
- Measure serum thyrotropin (TSH)
- Collect blood samples for HBsAg, anti-HCV antibody, HIV 1 and 2 antibodies
- Collect β-HCG pregnancy test for all females
- Collect FSH and estradiol blood samples in postmenopausal females who have had no menses for 6 to 18 months prior to study start
- Urinary drug abuse and breath alcohol test
- Record adverse events from signing of informed consent and concomitant medication usage

9.1.3. Predosing Phase*

Subjects who have successfully completed the screening assessments, who meet all inclusion criteria and do not demonstrate any exclusion criteria will be instructed to withdraw their existing anti-diabetic medications 16 days prior to dosing on Day 1, then asked to return to the CRU for admission on Day –3.

Day -17 to Day -3

Candidate subjects for this study will return for an outpatient visit on Day -17 for the following instruction.

* This section of the protocol has been revised. Please refer to the section of this document titled PROTOCOL AMENDMENTS (Amendment INT-4, 26 September 2007) for a detailed description of the specific changes.

- Discontinue use of existing anti-diabetic medication on Day -16
- Subjects will be counseled by a registered dietician designated by the investigators to follow a weight maintaining diet for 2 weeks prior to study admission. In addition, the study subjects will discuss about the status of the weight maintaining diet with the dietician by telephone on Day –9. The weight maintaining diet will follow standard recommendations of the American Diabetes Association, comprising ~55-60% carbohydrate, 10-20% protein, and ~30% fat, with total daily caloric intake individually adjusted for BMI.
- Subjects will be provided with glucose analyzers and instructed to monitor their blood glucose at least once each morning (fasting sample), and post lunch. The results will be recorded on a diary card and reported to the study center on Day -9 for entry into the eCRF. The subject will be instructed to report any signs or symptoms of hypoglycemia, fasting blood glucose values > 15 mM (270 mg/dL), post lunch values > 22.2 mM (400 mg/dL) and blood glucose values < 2.5 mM (45 mg/dL) to the study center. Subjects will be instructed to resume taking their previous oral anti-diabetic agent at any time during the washout period if confirmed fasting blood glucose levels rise above 15.0 mM (270 mg/dL) or postprandial glucose levels rise above 22.2 mM (400 mg/dL) following the lunch. Subjects will discontinue from the study after they safely resume their previous anti-diabetic medication at the discretion of the investigator.
- Collect FSH and estradiol blood samples in postmenopausal females who have had no menses for 6 to 18 months prior to study start

Day –3 to Day –1.

Subjects will be admitted to the study center on Day –3. After admission, inclusion/exclusion criteria, prohibitions/restrictions and diary cards will be reviewed. Eligible subjects will remain in the clinic until discharge on Day 20.

Subjects must fast overnight for 10 hours prior to the anticipated time of placebo dosing on Days –2 and –1 (between 8:00 to 9:00 AM) in a single blind fashion (subjects will be blinded).

The exact times for each blood draw or assessment will be recorded in the CRF.

The following assessments will be performed on Day –2:

- Physical examination including a thorough whole body skin assessment by a physician

- Body temperature
- Orthostatic vital signs at 4 and 8 hours post dose
- Urinary drug abuse, serum pregnancy and breath alcohol test (-2 day window allowed)
- Fasting clinical laboratory profiles: hematology, urinalysis and biochemistry (see Section 9.5.2. for details)
- Collect FSH and estradiol blood samples in postmenopausal females who have had no menses for 6 to 18 months prior to study start (-2 day window allowed)
- Urine NTX and urine deoxypyridinolines (DPD), serum bone specific alkaline phosphatase, serum osteocalcin (OC), 1,25-dihydroxy Vitamin D and PTH
- Administration of visual analogue scale (VAS) questionnaires to assess appetite and satiety at 5 minutes prior to breakfast, 30, 60, 90, 120, 150, 180, 210 and 240 minutes after starting breakfast, 5 minutes prior to lunch and immediately after lunch on Day –2 (Each subject will complete his/her VAS assessment in a separate area and will not be allowed to talk to others or exchange messages with others. (Attachment 11)
- A four-item patient reported outcomes questionnaire will be administered upon rising in the morning (Attachment 12). (Each subject will complete his/her outcome questionnaire in a separate area and will not be allowed to communicate or exchange messages with others.
- Record 24 hour fluid intake and urine output volume. The times and volumes of fluid intake or urine output volumes should be recorded in the eCRFs.
- Frequency of urination over each 24 hours except Days –1, 1 and 16 when fractionated urine samples will be collected, should also be recorded
- Record adverse events and concomitant medication usage

Following completion of the predose assessments, a dose of placebo will be administered in the sitting position between 08:00 to 09:00 AM in a single blind fashion (subjects will be blinded), and the exact time of dosing will be recorded in the CRF. Subjects will receive standardized breakfast starting within 10 minutes postdose, lunch and dinner at 4.5, 10.5 hours, respectively, post morning study drug administration. Water will be allowed ad libitum throughout Days –2 to 20. Fluid intake including those taken during each meal, urine volume and frequency over each 24-hour period will be recorded in the eCRFs.

The following assessments will be performed on Day –1:

- Fasting serum creatinine
- Whole body skin assessment by a physician
- Vital signs (blood pressure and heart rate, supine) and 12-lead ECGs will be performed at T0, T2, T6 and T12 where T0 corresponds to the dosing time on dosing days. Vital signs and 12-lead ECGs will be measured in triplicates in 2 minutes apart. Triplicate recordings, each 2 minutes apart, at each predefined point. The average of the triplicate measurements at each time point on Day –1 will serve each subject's time-matched baseline value for the corresponding parameters on Days 1 to 20. All morning vital signs and 12-lead ECGs will be performed prior to breakfast.
- Continuous Lead II ECG monitoring (telemetry) will be conducted from 30 minutes prior to till 8 hours post dosing.
- Urine samples will be collected on Day –1 at T0–T2, T2–T4.5, T4.5–T7, T7-10.5, T10.5-13, and T13-24, where T0 corresponds to the dosing time on dosing days. Urine volume of each collection interval will be recorded in the eCRFs. Two 10 ml aliquots of urine samples will be taken from from each collection interval. The otherwill be used for measurements fo for the following:
    - glucose
    - creatinine
    - electrolytes: sodium, potassium, chloride, phosphate, calcium and magnesium
    - pH, specific gravity and osmolarity
    - albumin
    - N-acetyl-beta-glucosaminidase, and beta-2-microglobulin
- 24-hour plasma glucose, insulin and C-peptide levels will be collected at T-0.5, T –0.25, T0 (predose), T0.5, T1, T1.25, T1.5, T2.0, T2.5, T3.0, T4.5 (prior to lunch), T5.0, T5.5, T6.0, T6.5, T7.0, T8.0, T9.0, T10.5 (prior to dinner), T11, T12.0, T12.5, T13.0, T14.0 T16.0, T19.0, T22.0, and T24 hours where T0 corresponds the dosing time (hours) on each dosing days
- Blood samples for GLP-1 active and total will be collected on Day -1 predose at -0.5 and 0.5, 1, 1.5 and 2.0 postdose
- Urine NTX and urine deoxypyridinolines (DPD), serum bone specific alkaline phosphatase, serum osteocalcin (OC), 1,25-dihydroxy Vitamin D and PTH
- Body weight will be assessed using a calibrated scale prior to breakfast
- Record 24 hour fluid intake and frequency of urination

9.1.4. Double-Blind Dosing Phase*

Days 1-22

Following completion of the Day –2 and –1 safety and PD baseline assessments, subjects will be randomized to receive JNJ-28431754 or placebo in a double-blind fashion between 08:00 to 09:00 AM on Day 1 and Days 3 through 16, and the exact time of dosing will be recorded in the CRF. No study drug will be administered on Day 2. Subjects will receive standardized breakfast within 10 minutes postdose, lunch and dinner at 4.5 and 10.5 hours, respectively, post morning study drug administration. Water will be allowed ad libitum. Fluid intake including those taken during each meal and urine volume and urine frequency over each 24-hour period will be recorded in the eCRFs.

The following assessments will be performed on Days 1-16. The exact times for each blood draw or assessment will be recorded in the CRF. Where assessments occur at the same time point, the blood sample for pharmacokinetic analysis must always be taken as close to the scheduled time as possible. For pharmacokinetic timepoints up to and including the 4-hour sample a window of ± 5 minutes is allowed and for postdose time points greater than the 8-hour sample a window of ±15 minutes is allowed. The order of multiple assessments within 1 protocol time point should also be the same throughout the study.

- Plasma samples for measurement of JNJ-28431754 and its metabolite concentrations will be collected for measurement of JNJ-28431754 concentration at predose, 0.5, 1, 1.5, 2, 2.5, 3, 4, 6, 8, 10, 12, 24 and 48 hours postdose on Days 1 and 16, and at pre-dose on Days 3, 5, 7, 9, 11, 13 and 15 and at 72, 96, 120 and 144 hours postdose on Day 16.
- Urine samples will be collected at predose, 0-2, 2-4.5, 4.5-7, 7-10.5, 10.5-13, 13-24 and 24-48 post dose on Day 1 and 0-2, 2-4.5, 4.5-7, 7-10.5, 10.5-13, 13-24, 24-48, 48-72 and 72-96 on Day 16. Four aliquots (10 mL each) will be taken from each urine collection fraction. One aliquot will be stored for potential future determination of additional analytes. One will be used for measurement of JNJ-28431754. One will be stored as a back-up sample. Another will be used to determine the following:
    - glucose

* This section of the protocol has been revised. Please refer to the section of this document titled PROTOCOL AMENDMENTS (Amendment INT-4, 26 September 2007) for a detailed description of the specific changes.

- creatinine
- electrolytes: sodium, chloride, potassium, phosphate, calcium and magnesium
- pH, specific gravity and osmolarity
- albumin
- N-acetyl-beta-glucosaminidase, and beta-2-microglobulin

- 0-24 hour urine samples will be collected on Days 3 to 15. For each urine collection, the above urinary assessments will be conducted. Urine volume collected in each time intervals will be recorded and aliquots taken after mixing for storage at -20°C and subsequent assay.
- 24-hour plasma glucose, insulin and C-peptide levels will be collected at –0.5, -0.25, 0 (predose), 0.5, 1, 1.25, 1.5, 2.0, 2.5, 3.0, 4.5 (prior to lunch), 5.0, 5.5, 6.0, 6.5, 7.0, 8.0, 9.0, 10.5 (prior to dinner), 11, 12.0, 12.5, 13.0, 14.0 16.0, 19.0, 22.0, and 24 hours on Days 1 and 16
- Blood samples for GLP-1 active and total will be collected on Days 1 and 16 at -0.5 predose and 0.5, 1, 1.5 and 2.0 hours postdose
- Morning fasting plasma glucose, insulin and C-peptide on Days 3 to 22
- Administration of visual analogue scale (VAS) questionnaires to assess appetite and satiety at 5 minutes prior to breakfast, 30, 60, 90, 120, 150, 180, 210 and 240 minutes after starting breakfast, 5 minutes prior to lunch and immediately after lunch on Days 3, 7, 11 and Day 15. (Each subject will complete his/her VAS assessment in a separate area and will not be allowed to talk to others or exchange messages with others. (Attachment 11)
- Morning fasting body weight will be assessed on Days 1 through 20 using a calibrated scale.
- Physical examinations will be performed on Days 15 and 20
- Body temperature will be measured in the morning at Days 1 to 20
- Whole body skin assessments on Days 1 to 20 by a physician
- Vital signs (blood pressure and heart rate, supine) and 12-lead ECGs will be performed at 0 (predose), 2, 6, 12, 24, 48, 72 and 96 hours post dose on Days 1 and 16; vital signs will also be measured at 2, 6 and 12 hours on Days 3 to 15, ECGs at 2 hours post dose on Days 3 to 15.. Vital signs and 12-lead ECGs will be measured in triplicate 2 minutes apart. Triplicate recordings, each 2 minutes apart, at each predefined point. The average of the triplicate measurements at each time point on Day –1 will serve each subject's time-matched baseline value for the corresponding parameters on Days 1 to 20. All morning vital signs and 12-lead ECGs will be performed prior to dosing on study drug administration days and prior to breakfast on non-dosing days.

- Continuous Lead II ECG monitoring (telemetry) will be conducted from 30 minutes prior to till 8 hours post dosing on Day 1, 6, 8 and 15.
- Orthostatic vital signs (blood pressure and heart rate) will be measured after 3 minutes standing following the supine vital sign measurements at 4 and 8 hours post dose or corresponding to dosing time on Days 1 to 20.
- Adverse events will be recorded during the whole study and will be specifically queried on each dosing day at predose, at 3, 6 and 12 hours post dose, on the mornings of Days 2, 20, 21, 22 and at the follow up visit.
- Fasting samples for hematology, clinical chemistry and urinalysis will be collected on Days 2, 3, 5, 8, 11, 14, 17, 18, 20, and 22.
- A coagulation sample will be collected on Day 20.
- Urine NTX and urine deoxypyridinolines (DPD), serum bone specific alkaline phosphatase and serum osteocalcin (OC) will be assessed on Days 2, 3, 5, 8, 11, 14, 17, 18 and 20.
- Serum 1,25-dihydroxy Vitamin D, and parathyroid hormone (PTH) will be assessed on Days -2, -1, 5, 11 and 17.
- A four-item patient reported outcomes questionnaire will be administered on Days 4, 7 and 14 to each subject upon rising in the morning (Attachment 12). (Each subject will complete his/her assessment in a separate area and will not be allowed to talk to others or exchange messages with others. (Attachment 12)
- All fluid intake, urine volume and frequency of urination over each 24 hour period will be recorded in the eCRFs throughout Days 1 to 19
- A 10 mL blood sample will be collected from subjects who gave separate informed consent for the pharmacogenomic component of the study, on Day 1.
- Concomitant medications will be recorded throughout the study.
- Real time blood glucose concentrations will be determined prior to each standardized breakfast and 2 hours post each meal using a glucose analyzer throughout Days 1 to 20. Investigational staff will monitor subjects for symptoms possibly indicative of hypoglycemia throughout the in-clinic residence. Blood glucose will be immediately measured using a glucose analyzer should hypoglycemia symptoms appear and confirmed by conventional venous sampling. Attachment 7 provides the clinical signs, symptoms, grading scale, and treatment for hypoglycemia. Should hypoglycemia occur it should be entered in the eCRF adverse event page (see Attachment 7).

On Day 20 following completion of all postdose assessments, subjects may be discharged, unless there are clinically significant adverse events in which case the subject will be asked to remain in the clinic. When discharged,

subjects will be instructed that they have to follow the restrictions as outlined in Section 4.4 (Prohibitions and Restrictions).

- Subjects will be given a contact number for the on-call physician at the clinic that can be called 24 hours a day 7 days a week in case an adverse event occurs. Subjects will be instructed to return to the clinic daily in the morning on Days 21 to 22. They will also return for a Follow-up visit 7 to 10 days post the Day 22 visit.

9.1.5. Outpatient Phase

Days 21 to 22

Assessments should be performed relative to the previous study drug administration time.

- Plasma samples for measurement of JNJ-28431754 and its metabolites at 120 (Day 21) and 144 hours (Day 22) post dose
- Morning fasting plasma glucose, insulin and C-peptide concentrations will be obtained on Days 21 and 22.
- Adverse events will be recorded during the whole study and will be specifically queried on the mornings of Days 21 and 22.
- Fasting samples for hematology, clinical chemistry and urinalysis will be collected on Day 22
- Urine NTX and urine deoxypyridinolines (DPD), serum bone specific alkaline phosphatase, serum osteocalcin (OC), serum 1,25-dihydroxy Vitamin D and PTH will be assessed on Day 22.
- Concomitant medications will be recorded throughout the study.

Subjects will be instructed to resume taking their previous oral anti-diabetic agent on Day 22, or at any time during the study if confirmed fasting blood glucose levels rise above 15.0 mM or postprandial glucose levels rise above 22.2 mM (400 mg/dL) following lunch. Instruction will also include reinforcement of signs and symptoms of hypoglycemia. Subjects will be instructed to monitor their blood glucose via fingerstick using a glucose meter at least once each morning (fasting sample) and post lunch.. The results will be recorded on a diary card and reported to the study center for entry into the eCRF. The subject will be instructed to report any signs or symptoms of hypoglycemia, fasting blood glucose values > 15 mM (270 mg/dL), post lunch values > 22.2 mM (400 mg/dL) and blood glucose values < 2.5 mM to the study center. For the safety of the study subjects, they will remain in the clinic for approximately 6 hours after re-starting their anti-diabetic medications.

9.1.6. Posttreatment Phase (Follow-Up)

Subjects will return to the clinical study center 7 to 10 days following the Day 22 visit for a Follow-up visit. The following assessments will be performed:

- Physical examination including thorough whole body skin assessment
- Vital signs (blood pressure and heart rate) and 12-lead ECG
- Body temperature
- Morning fasting body weight
- Adverse events will be recorded during the entire study and will be specifically queried during the follow-up visit
- Fasting samples for hematology, clinical chemistry and urinalysis dipstick
- Urine NTX and urine deoxypyridinolines (DPD), serum bone specific alkaline phosphatase, serum osteocalcin (OC), serum 1,25-dihydroxy Vitamin D and PTH will be assessed.

9.2. Pharmacokinetic Evaluations

9.2.1. Sample Collection and Handling

9.2.1.1. Plasma:

Day 1:

Venous blood samples (3 mL) for measurement of JNJ-28431754 and its metabolites concentration will be collected at predose, 0.5, 1, 1.5, 2, 2.5, 3, 4, 6, 8, 10, 12, 24 and 48 hours postdose (total 14 samples). If a twice-daily dosing regimen is administered, 6 additional PK plasma samples will be collected at 10.5 (prior to dinner), 13, 14, 16, 19 and 22 hours post the morning dose.

Days 3 to 15:

Venous blood samples (3 mL) will be collected at pre-dose in the morning on Days 3, 5, 7, 9, 11, 13 and 15 to obtain trough JNJ-28431754 and its metabolites concentrations

Day 16:

Venous blood samples (3 mL) for measurement of JNJ-28431754 and its metabolites concentration will be collected at predose, 0.5, 1, 1.5, 2, 2.5, 3, 4, 6, 8, 10, 12, 24, 48, 72, 96, 120 and 144 hours postdose (total 18 samples). If a twice-daily dosing regimen is administered, 6 additional PK plasma

samples will be collected at 10.5 (prior to dinner), 13, 14, 16, 19 and 22 hours post the morning dose.

Collection times may be modified as pharmacokinetic data become available during the study, but total blood volume collection for each subject for all analysis (PK, PD, safety) will not exceed 550 mL over 8 weeks in this study.

Plasma and urine samples will be stored following analysis for potential identification/analysis of further JNJ-28431754 metabolites or concomitant medication pharmacokinetic analysis and will be reported separately.

The exact dates and times of blood sampling must be recorded in the CRF. Blood samples will be collected from an intravenous cannula or by direct venipuncture if required or if the cannula is not functioning. If blood samples are collected via an indwelling cannula, the following procedure will be used. The saline infusion will be stopped and 2 mL of blood drawn into a syringe attached to a stopcock. The required volume of blood is drawn into a second syringe according to instructions provided by the laboratory. The 2 mL of blood and saline in the first syringe will be reinjected and the slow saline infusion continued.

Refer to Attachments 1, 2 and 3 for further information regarding handling, shipment and labeling of biological samples.

9.2.1.2. Urine

Day 1:

Urine samples for analysis of JNJ-28431754 and its metabolites will be collected at predose, 0-2, 2-4.5 (prior to lunch), 4.5-7, 7-10.5 (prior to dinner), 10.5-13, 13-24 and 24-48 hours post dose.

Day 16:

Urine samples for analysis of JNJ-28431754 and its metabolites concentration will be collected at 0-2, 2-4.5 (prior to lunch), 4.5-7, 7-10.5 (prior to dinner), 10.5-13, 13-24, 24-48, 48-72 and 72-96 hours post dose.

Subjects will be instructed to follow the standard urine collection procedure. Subjects will void their bladders just before drug administration. To ensure a complete urine collection during each collection interval, subjects should completely empty their bladders at the end of each interval. The urine

collection time (start/end) and the total collected volume (or weight) during each collection interval must be recorded on CRF. Samples of each collection interval need to be well mixed, within 1 hour after each interval, and before sampling the aliquot for the PK, PD, and Safety Lab analysis. Two 10 mL aliquots of each collection interval will be stored at -20°C for the measurement of JNJ-28431754 and its metabolite levels in the urine. Aliquots will also be taken for assay of urine PD and safety samples (See Sections 9.3 and 9.5). Attachments 1-3 provide further information regarding sample handling, labeling, and shipment.

9.2.2. Analytical Procedures

Pharmacokinetic plasma and urine samples will be analyzed for concentrations of JNJ-28431754 using validated, selective and sensitive liquid chromatography-mass spectrometry (LC-MS/MS) methods under the responsibility of the Bioanalytical Group at J&JPRD

After quantification of JNJ-28431754, the remaining plasma and urine samples will be stored at -20°C for future metabolite profiling and/or metabolite quantification as deemed necessary by the sponsor (to be reported separately from this study).

Samples collected from the placebo subjects will not be analyzed except 2 samples: one predose and the other around the expected t_{max}. This is to ensure from a safety perspective that no placebo subject could have been incorrectly dosed with JNJ-28431754.

9.2.3. Pharmacokinetic Parameters

9.2.3.1. Plasma

Based on the individual plasma concentration-time data, using the actual sampling times, the following pharmacokinetic parameters of JNJ-28431754 and its metabolites will be estimated:

Day 1

C_{max} peak plasma concentration

t_{max} time to reach the peak plasma concentration

CL/F — Apparent systemic clearance following oral administration, calculated as: D/AUC_{τ} ; for the parent compound, JNJ-28630368, only

AUC_{24hr} — area under the curve from 0 to 24 hours post dosing, calculated by trapezoidal summation

AUC_{∞} — AUC_t extrapolated to infinity, calculated as $AUC_t + C_{last}/\lambda_z$

λ_z — elimination rate constant, determined by linear regression of the terminal points of the ln-linear plasma concentration-time curve

$t_{½}$ — terminal half-life, defined as $0.693/\lambda_z$

Day 3-15

C_{trough} trough plasma concentration prior to dosing or at the end of the dosing interval of any dose other than the first dose

Day 16

$C_{max,\ ss}$ — maximum plasma concentration during a dosing interval at steady state

$AUC_{\tau,ss}$ — area under the plasma concentration-time curve during a dosing interval (τ) at steady-state

CL/F — Apparent systemic clearance following oral administration, calculated as: D/AUC_{τ} ; for the parent compound, JNJ-28630368, only

Vd_{ss}/F — apparent volume of distribution at steady-state following oral administration, calculated as MRT*CL; for the parent compound, JNJ-28630368, only.

FI — fluctuation index, i.e., percentage fluctuation (variation between peak and trough at steady-state), calculated as $100*[(C_{max,\ ss}- C_{min,\ ss})/C_{avg,ss}]$

Acc Ratio — accumulation index, calculated by using the following equation: $Acc\ Ratio = \frac{AUC_{\tau,ss}}{AUC_{\tau,sd}}$

where $AUC_{\tau,ss}$ and $AUC_{\tau,sd}$ are the AUCs over the dosing interval (τ) at steady-state (ss) and following a single dose (sd), respectively.

$t_{½}$ terminal half-life, defined as $0.693/\lambda_z$, where λ_z is the elimination rate constant

Additional plasma PK parameters may be determined as appropriate.

9.2.3.2. Urine

Based on the urinary excretion data (Days 1 and 16), the following parameters will be calculated, as appropriate for JNJ–28431754

Aet total urinary recovery of unchanged drug over interval 0-t hours

CLR renal clearance calculated as the cumulative amount excreted in urine (Aet) divided by the AUCt

Ae (% dose) percentage of the dose excreted in the urine unchanged calculated as (Ae/Dose)*100

Additional urine PK parameters may be determined as appropriate.

9.3. Pharmacodynamic Evaluations

9.3.1. Primary

1. Change from baseline mean 24-hour plasma glucose concentration on Days 1 and 16, which is defined as the area under the plasma glucose time concentration time curve at 0-24 hours divided by 24 hours (Glucose AUC 0-24/24h). Glucose AUC 0-24/24h determined on Day –1 will be used as the baseline values.
2. Change from baseline urine glucose excretion (UGE)
   a. Cumulative amount over each 24 hours on Days 1, 2, 8, 12, 16, 17, 18, 19; cumulative amount over 24 hours on Day –1 will be used a the baseline value.
   b. UGE rate, which is defined as the UGE amount in each fraction divided by hours of each collection interval on Days 1, 2, 8, 12, 16, 17, 18, 19

9.3.2. Secondary:

1. Renal threshold (R_T), which is defined as the plasma glucose level at which maximum urinary glucose reabsorption is achieved and above which glucose is excreted in the urine will be determined on Days -1,

1 and 16. The R_T is calculated as a function of time for each subject based on measured plasma glucose, urinary glucose excretion, and glomerular filtration rate (GFR)

The equations used to calculate R_T between times t1 and t2 are

$$GFR\int_{t_1}^{t_2} f(G,R_t)dt = UGE(t_1,t_2)\text{; (1)}$$

$$f(G,R_T) = \begin{cases} G - R_T & \text{if } G > R_T \\ 0 & \text{if } G < R_T \end{cases} \text{ (2)}$$

Where G is the plasma glucose concentration, UGE (t1,t2) is the amount of glucose excreted in the urine between times t1 and t2. During each time interval that UGE is collected, the integral is calculated using the trapezoidal method and the mean value of R_T over this interval is calculated

2. Change from baseline mean 24-hour insulin concentration on Days 1 and 16, which is defined as the area under the plasma insulin time concentration time curve at 0-24 hours divided by 24 hours (Insulin AUC 0-24/24h). Insulin AUC 0-24/24h determined on Day –1 will be used as the baseline values.
3. Changes from baseline morning fasting plasma glucose (FPG) and insulin (FPI) on Days 1 and 16. FPG and FPI values determined on Day –1 will be used as the baseline value. FPG and FPI on Days –1, 1 and 16 will be calculated by averaging predose values at –30, -15 and 0 minutes. On Days 2 to 15 and 17 to 20, one FPG and FPI will be determined within 30 minutes prior to dosing on dosing days or prior to breakfast on non-dosing days.
4. Change from baseline glucose and insulin excursions: after each breakfast, defined as the difference between the maximum glucose and insulin values observed during the 4-h post meal period minus the mean of the premeal measurements at –30, -15 and 0 min on Days 1 and 16. Glucose and insulin excursion on Day –1 will be used as the baseline value.
5. Glucose AUC 0-2 on Days 1, and 16; Glucose AUC 0-2 on Days –1 will be used as the baseline value.
6. Insulin sensitivity (IS) will be calculated based on plasma glucose and insulin concentration time curves post breakfast on Day –1, 1 and 16 using a robust algebraic method, namely S_IAR_aA.[13]
7. Beta-cell function

Two measures of beta-cell function will be calculated from the glucose and insulin and C-peptide curves post breakfast on Days –1, 1 and 16.

Insulinogenic index, a measure of early insulin release and is $= \frac{I(30) - I(0)}{G(30) - G(0)}$

Insulin secretion rate (ISR), ISR will be calculated based on measured C-peptide and glucose concentrations using a modified method of Mari et al.[14]

8. Active and total glucagon-like peptide-1 (GLP-1) levels on Days -1, 1 and 16 at -0.5 predose and 0.5, 1, 1.5 and 2.0 postdose
9. Assessment of Visual Analogue Scale (VAS) questionnaires to assess appetite and satiety at 5 minutes prior to breakfast, 30, 60, 90, 120, 150, 180, 210 and 240 minutes after starting breakfast, 5 minutes prior to lunch and immediately after lunch on Days –2, 3, 7, 11 and Day 15. The VAS questionnaire schedule may be modified if a twice-daily dosing regimen is tested. (Each subject will complete his/her VAS assessment in a separate area and will not be allowed to talk to others or exchange messages with others. (Attachment 11)
10. Morning fasting body weight will be assessed at Screening and on Days -1 through 20 and at the Follow-up visit using a calibrated scale.

9.4. Pharmacogenomics

A 10 mL blood sample will be collected from subjects who consent to the pharmacogenomic component of the study. Instructions on sample handling, labeling and shipment are provided in Attachment 4. There are two parts to the pharmacogenomic component of this study:

9.4.1. Analyses Related to the Trial (Part A)

This part of pharmacogenomic research allows for the analysis of genes that may be relevant to JNJ-28431754 or Type 2 diabetes mellitus. Candidate genes will only be genotyped, as necessary, if it is hypothesized that this may help resolve issues with the clinical data. The genes that are currently hypothesized to potentially be relevant to JNJ-28431754 or Type 2 diabetes mellitus are provided below. Genotyping of any of the candidate genes listed below would be performed on identifiable samples.

ADME related genes: *ABCB1, ABCB4, ABCC1, ABCC2, ADH gene family, AHR, ALDH gene family, ARNT, CYP1A1, CYP1A2, CYP1B1, CYP2A6, CYP2B6, CYP2C19, CYP2C8, CYP2C9, CYP2D6, CYP2E1, CYP3A4,*

CYP3A5, CYP4B1, EPHX1, EPHX2, FMO1, FMO2, FMO3, FMO4, GSTM1, GSTP2, GSTT1, MPO, NAT1, NAT2, NFE2L2, NR1I2, SULT1A1, SULT1A2, SULT2A1, TPMT, UGT1A1, UGT1A3, UGT1A4, UGT1A6, UGT1A7, UGT1A8, UGT1A9, UGT2B15, UGT2B4, UGT2B7, SLC22A1-5 (OCT gene family), SLC22A6-8 and SLC22A6-11 (OAT gene family), SLC21A3, SLC21A9, SLC21A11 and SLC21A12 (OATP gene family).

Target related genes: *SGLT gene family (SLC5 family), GLUT gene family (SLC2 family)*.

Diabetic related genes: ABCA1, ABCC8, ACE, ACP1, ACTN4, ADA, ADIPOQ, ADRB3, AKR1B1, ALB, ALMS1, ANGPTL4, APOA1, APOA4, APOB, APOC3, APOE, ARG1, ASIP, BBS4, BCHE, CAPN10, CART, CCK, CCKAR, CCL2, CD36, CD59, CIDEA, CPE, CTLA4, CYP19A1, DF, DIANPH, DPP4, ENPP1, EPHX2, ESR1, FABP10, FABP2, FABP4, FABP5, FASN, FOXC2, FXN, GAD1, GAD2, GAL, GCG, GCGR, GCK, GCKR, GDF8, GH1, GH2, GHRL, GIP, GIPR, GNB3, GPD2, GPKOW, GYS1, HBA1, HFE, HK1, HK2, HK3, HLA-DQA1, HLA-DQB1, HMGA2, HNF4A, HSD11B1, HSD17B7, IAPP, IDE, IGF1, IGF1R, IKBKB, IL6, INS, INSR, IPF1, IRS1, IRS2, IRS4, ISL1, JPH3, KCNJ11, KCNJ9, LEP, LEPR, LIPC, LIPE, LMNA, LPA, LPAL2, LPL, LRPAP1, MAPK8, MAPK8IP1, MC3R, MC4R, MKKS, NEUROD1, NEUROG3, NFKB1, NOS3, NPY, NR0B2, NR3C1, NUCB2, PBX1, PCSK1, PDE3B, PGC, PGR, PLG, PLIN, POMC, PON1, PON2, PPARA, PPARD, PPARG, PPARGC1A, PPARGC1B, PPP1R1A, PPP1R3A, PRL, PTPN1, PTPRN, RBP4, REG1A, RETN, RPS6KB1, SCARB1, SERPINE1, SIM1, SLC2A1, SLC2A10, SLC2A2, SLC2A4, SORBS1, SPINK1, SREBF1, SST, TCF1, TCF2, TCF7L2, TGFB1, TH, TNF, TRAPPC2, TUB, TULP2, UCN, UCP1, UCP2, UCP3, VDR, WFS1, WRN, XBP1, and ZDHHC23.

9.4.2. DNA Storage for Future Analyses (Part B)

This part of the pharmacogenomic research allows for the storage of DNA samples for future genetic research related to JNJ-28431754 or the indications for which it is developed. Stored DNA samples and relevant clinical data will be held in a non-identifiable format, whereby the study subject identifier is replaced with a new number, thus limiting the possibility of linking genetic data to a subject's identity. Samples will be made non-identifiable after the Clinical Study Report has been issued.

Subjects will be given the option to participate in Part A, Part B, both parts, or neither part of the pharmacogenomic component of this study (where local regulations permit).

9.5. Safety Evaluations

9.5.1. Adverse Events

Spontaneously reported adverse events will be recorded for the entire duration of the study i.e., from the time a signed and dated informed consent form is obtained until completion of the last study-related procedure at the safety follow-up visit. Adverse events will also be specifically queried using non-directive questioning (e.g. study staff will ask the subjects "How are you feeling?") on Day -2 to 1 and 3 to 16 at predose and at 3, 6 and 12 hours post dose (placebo dose on Days -2 and -1), on the mornings of Days 2, 20, 21, 22 and at the follow-up visit. Specific details on adverse event reporting are provided in Section 12.

9.5.2. Clinical Laboratory

Clinical safety laboratory tests (hematology, clinical chemistry, urinary analysis using both dipstick and microscopic assessments) will be conducted at the fasting condition at screening, on Days -2, 2, 3, 5, 8, 11, 14, 17, 18, 20, and 22 and at the final follow-up visit 7 to 10 days post Day 22. Coagulation (INR and aPTT) will be assessed at screening and Day 20. TSH will be done at screening. The Principal Investigator or his/her designees must review all laboratory reports; document his reviews and record any clinically relevant changes occurring during the study in the adverse event section of the eCRF. The following tests will be performed:

<u>Hematology Panel</u>

Hemoglobin	Platelet count
Hematocrit	Percent reticulocytes
Red blood cell (RBC) count	HbA1c (screening only)
White blood cell (WBC) count with differential	

Clinical Chemistry Panel

Serum electrolytes: sodium, potassium, calcium, magnesium, chloride, inorganic phosphate, bicarbonates, uric acid	Aspartate aminotransferase (AST)
Serum creatinine, GFR*	Alanine aminotransferase (ALT)
Alkaline phosphatase	Gamma-glutamyltransferase (GGT)
Lactic dehydrogenase (LDH)	Total bilirubin
Albumin	Total cholesterol
Total protein	LDL- cholesterol
Blood urea nitrogen (BUN)	HDL- cholesterol
Creatine phosphokinase (CPK)	triglycerides
Serum osmolality	serum glucose
Anion gap**	

*Glomerular filtration rate (GFR) calculated using the MDRD equation defined as: GFR = 175 x (standardized Scr)$^{-1.154}$ x (age) $^{-0.203}$ x 0.742 (if the subject is female) x 1.212 (if the subject is black). GFR is expressed in milliliters per minute per 1.73 m^2 and race is either black or not.[15]

** The anion gap (AG) formula: AG = Na – (Cl + HCO^3)

Urinalysis

Dipstick	Sediment
specific gravity	RBC
pH	WBC
glucose	epithelial cells
protein	crystals
blood	casts
ketones	bacteria
bilirubin	
urobilinogen	
nitrite	
leukocyte esterase	

Renal safety assessments:

Urine samples will be collected at predose, 0-2, 2-4.5 (pre lunch), 4.5-7, 7-10.5 (pre dinner), 10.5-13 and 13-24 hours post dose on Day -1 at 0-2, 2-4.5 (pre lunch), 4.5-7, 7-10.5 (pre dinner), 10.5-13, 13-24 and 24-48 hours post dose on Day 1 and at 0-2, 2-4.5 (pre lunch), 4.5-7, 7-10.5 (pre dinner), 10.5-13, 13-24, 24-48, 48-72 and 72-96 hours post dose on Day 16. Urine samples will also be collected at 0-24 hour interval post each dose on Days 3 to 15. For each urine collection, the followings will be measured:

1. glucose
2. creatinine
3. electrolytes: sodium, potassium, chloride, phosphate, calcium and magnesium
4. pH, specific gravities and osmolarity
5. albumin
6. N-acetyl-beta-glucosaminidase, and beta-2-microglobulin

Subjects will be instructed to follow the standard urine collection procedure. Subjects will void their bladders just before each drug administration. To ensure a complete urine collection during each collection interval, subjects should completely empty their bladders at the end of each interval. The

urine collection time (start/end) and the total collected volume (or weight) during each collection interval must be recorded on CRF. Samples of each collection interval need to be well mixed, within 1 hour after each interval, and before sampling the aliquot for the renal safety lab assessments. Two 10 mL aliquots of each collection interval will be stored at -20°C for the measurement of the renal safety samples.

24-hour creatinine clearance (CLcr) on Day –1, 3, 5, 8, 11, 14, 16, 17, 18 and 19

CLcr [mL/min] = ((Creatinine urine [mg/dL]) x (V urine [mL/min])/Creatinine plasma [mg/dL]) x (1.73/BSA[m2])

BSA = Body Surface Area that can be obtained from nomograms which require height in cm and weight in kg

The amount of urine creatinine in each urine sample will be added to obtain a 24-hour cumulative urine creatinine, which will be used for calculation of CLcr along with serum creatinine determined on the same day.

Other

Bone turnover biomarkers

Urine NTX and urine deoxypyridinolines (DPD), serum bone specific alkaline phosphatase and serum osteocalcin (OC) will be assessed on Days –2, -1, 2, 3, 5, 8, 11, 14, 17, 18, 20, and 22 and at the follow up visit. Serum 1,25-dihydroxy Vitamin D, and parathyroid hormone (PTH) will be assessed on Days -2, -1, 5, 11, 17, 22 and at the Follow-up visit.

Coagulation

aPTT and INR will be assessed at screening and Day 20

Serology tests

HBsAg, anti-HCV antibody, HIV 1 and 2 antibodies will be tested at screening.

Endocrinology tests

ß-HCG, Follicle stimulating hormone (FSH), estradiol in females only at screening, Day -17 and Day -2 (a -2 day window is allowed at Day -2)

Thyroid function test:

TSH will be measured at screening.

Drug/Alcohol Screen:

A drug and alcohol screen will be performed at screening and Day -2.

9.5.3. Cardiac Monitoring*

Full 12-lead ECGs will be recorded. Subjects should be rested quietly in the supine position for at least 10 minutes before the measurement is started. 12-lead ECGs will be recorded at a paper speed of 25 mm/sec so that the different ECG intervals (RR, PR, QRS, QT, QTc) can be measured. The ECG will be recorded until 4 regular consecutive complexes are available for analysis. QT uncorrected, QTcB (corrected QT interval, calculated based on Bazett's method; primary) and QTcF (corrected QT interval, calculated based on Fridericia's method; secondary) will be calculated and presented.[15-18]

12-lead ECGs will be performed at screening and at T0, T2, T6 and T12 on Day –1 where T corresponds the dosing time on dosing days. The 12-lead ECGs will be performed at 0 (predose), 2, 6, 12, 24, and 48 hours post dose on Days 1 and 16, and at 2 hours on Days 3 to 15 and once at follow-up. The 12-lead ECGs will be measured in triplicates in 2 minutes apart at each predefined point. The average of the triplicate measurements at each time point on Day –1 will serve each subject's time-matched baseline value for the corresponding parameters on Days 1 to 20. ECGs will be evaluated by the Principal Investigator or the physician designee. All morning vital signs and 12-lead ECGs will be performed prior to breakfast.

Continuous Lead II ECG monitoring (telemetry) will be conducted from 30 minutes prior to till 8 hours post dosing on Days -1, 1, 6, 8 and 15. These data will not be recorded on the database and are for visual monitoring. Any abnormality detected by the device or the investigator will be printed out and retained as source data. Any clinically significant abnormalities will be recorded as adverse events.

* This section of the protocol has been revised. Please refer to the section of this document titled PROTOCOL AMENDMENTS (Amendment INT-4, 26 September 2007) for a detailed description of the specific changes.

9.5.4. Vital Signs

Systolic and diastolic blood pressure and pulse rate measurements will be assessed with a completely automated device consisting of an inflatable cuff and an oscillatory detection system. All values will be registered on a built-in digital recording system so that measurements are observer-independent. Manual blood pressure reading may be obtained in the event of instrument malfunction.

Systolic and diastolic blood pressure and heart rate measurements will be assessed following 5 minutes in the supine position and 3 minutes after standing (to assess orthostasis) using the contra-lateral arm to that used for blood sampling.

Vital signs (blood pressure and heart rate, supine) will be performed at screening and at T0, T2, T6 and T12 on Day –1 where T corresponds the dosing time on dosing days. Vital signs will also be performed at 0 (predose), 2, 6, 12, 24 and 48 hours post dose on Days 1 and 16, and at 2, 6 and 12 hours on Days 3 to 15 and once at the follow-up visit. The vital signs will be measured in triplicate 2 minutes apart at each predefined point. The average of the triplicate measurements at each time point on Day –1 will serve each subject's time-matched baseline value for the corresponding parameters on Days 1 to 20. All morning vital signs will be performed prior to breakfast.

Orthostatic vital signs will be measured on Day –2 and at 4 and 8 hours post dose or corresponding to dosing time on Days 1 to 20. Orthostatic vital signs will also be measured in triplicate.

Body temperature will be measured at screening in the morning, on Days -2, Days 1 to 20 and at the follow-up visit.

Height will be measured at the screening visit and will be utilized to calculate BMI and BSA. Weight will be measured at screening, on a calibrated scale in the morning, prior to meals and after voiding on Days -1 to 20 and at the Follow-up visit. Subjects will wear a hospital gown or light clothes, without shoes for each of the measurements. Scale calibration will occur weekly, and records stored in study files.

9.5.5. Physical Examination

Physical examinations will be performed at screening and on Days –2, 15, 20 and at the final follow-up visit. The physical examination will include a thorough whole body skin assessment. Any observed skin abnormalities should be recorded in the eCRFs. The study investigator, or authorized designee, will perform the physical examinations. If any clinically significant change is noted from Screening, it will be reported as an adverse event and will be followed up to resolution or reaching a stable end point.

The investigator will follow any clinically significant abnormalities persisting at the end of the study until resolution or until reaching a clinically stable endpoint.

9.5.6. Monitoring Blood Glucose Levels

During the washout period starting 16 days prior to dosing, subjects will be provided with glucometers and instructed to monitor their blood glucose levels at least once each morning (fasting sample) and post lunch. The results will be recorded on a diary card and reported to the study center on Day -9 for entry into the eCRF. The subject will be instructed to report any signs or symptoms of hypoglycemia, fasting blood glucose values > 15 mM (270 mg/dL), post lunch values > 22.2 mM (400 mg/dL) and blood glucose values ≤ 2.5 mM (45 mg/dL) to the study center. Subjects will be instructed to resume their previous oral anti-diabetic agent at any time during the washout period if confirmed fasting blood glucose levels rise above 15.0 mM (270 mg/dL) or postprandial glucose levels rise above 22.2 mM (400 mg/dL) following lunch. Instruction will also include reinforcement of signs and symptoms of hypoglycemia.

Investigational staff will monitor subjects for symptoms possibly indicative of hypoglycemia throughout the in-clinic residence, Days -3 to 20. Real time blood glucose concentrations will be determined prior to each standardized breakfast and 2 hours post each lunch using a glucose analyzer. Blood glucose will be immediately measured using a glucose analyzer should hypoglycemia symptoms appear and confirmed by conventional venous sampling. Attachment 7 provides the clinical signs, symptoms, grading scale, and treatment for hypoglycemia. Should hypoglycemia occur it should be entered in the eCRF adverse event page (see Attachment 7).

9.5.7. Monitoring for Skin Reactions

Subjects will be domiciled continuously from Days -3 through Day 20 and instructed to avoid direct exposure to sunlight or artificial sources of intense light for at least 96 hours post the last dose to minimize the risk of potential photosensitivity. In addition, subjects will be closely monitored for potential skin reactions with a thorough examination of the whole body skin for skin reactions (e.g. erythema, rash) by a physician or his/her designee at screening, on Days -1 through Day 20 at each dose level.

10. SUBJECT COMPLETION/WITHDRAWAL

10.1. Completion

A subject will be considered as having completed the study if he/she has completed all assessments up to and including all Follow-up assessments. However, data for all subjects receiving at least 1 dose of study drug and at least 1 pharmacodynamic assessment post randomization will be included in the pharmacodynamic analysis. Data for all subjects receiving a dose of JNJ-28431754 will be included in the pharmacokinetic analysis.

10.2. Discontinuation of Treatment

A subject should be discontinued from study treatment if

- the investigator believes that for safety reasons (e.g., adverse event) it is in the best interest of the subject to stop treatment
- any subject with a confirmed fasting blood glucose elevated above 15 mM (270 mg/dL) or a postprandial glucose level above 22.2 mM (400 mg/dL) following lunch will not be further administered study medication, and will be treated with appropriate hypoglycemic therapy and monitored until blood glucose levels have stabilized.

If a subject discontinues treatment before the end of the double-blind phase, further assessments and final Follow-up will be done unless the subject is withdrawn from the study (section 10.3).

10.3. Withdrawal From the Study

A subject will be withdrawn from the study for any of the following reasons:

1. lost to Follow-up
2. withdrawal of consent
3. safety reasons

No further dose escalation will occur if either more than 50% of the subjects experience moderate or severe adverse events or one subject experiences a serious adverse event of which a relationship to the trial medication can not be excluded.

When a subject withdraws before completing the study, the reason for withdrawal is to be documented on the CRF and in the source document. Every effort should be made to have the subject return to the study center and complete the assessments required for the Follow-up visit.

Study drug assigned to the withdrawn subject may not be assigned to another subject.

In case a subject is lost-to-Follow-up, every possible effort must be made by the study site personnel to contact the subject and determine the reason for discontinuation. The measures taken to follow up must be documented.

Subjects withdrawing from the main part of the study have the following options regarding pharmacogenomic research:

The DNA extracted from the subject's blood will be retained and used in accordance with the subject's DNA informed consent.

The subject may withdraw consent for pharmacogenomic research, in which case, the DNA sample will be destroyed and no further testing will take place. To initiate the sample destruction process, the investigator must notify the sponsor site contact to request sample destruction. The sponsor site contact will, in turn, contact the pharmacogenomics representative for sample destruction. Upon request, the investigator will receive written confirmation from the sponsor that the sample has been destroyed.

Withdrawal from Pharmacogenomic Research Only

- The subject may also withdraw consent for pharmacogenomic research only, while remaining in the clinical study. If a subject withdraws consent for pharmacogenomic research, any DNA extracted from the subject's blood will be destroyed. The sample destruction process will proceed as described above. After the clinical study is over, the sample will be made non-identifiable and cannot be found in order to be destroyed. If the sample has already undergone conversion to the non-identifiable format, the sponsor will notify the investigator in writing.

11. STATISTICAL METHODS

All data will be fully listed. Unless otherwise noted, all the measures indicated below will be summarized with descriptive statistics for each dose level and all placebo observations grouped together.

Subjects who need to restart their previous anti-diabetic medications during the treatment period will stop receiving study medication and will be closely monitored. These subjects will be withdrawn from the study; however, all safety, tolerability, PK and/or PD data collected from these subjects will be included in the final data analysis.

All statistical analyses will be considered exploratory and interpreted as such. No corrections will be made for multiple comparisons.

11.1. Sample Size Determination

It is estimated that a sample size of 20 (16 receiving active JNJ-28431754 and 4 receiving placebo) subjects with T2DM should be sufficient to detect a 15% reduction in 24-hour mean plasma glucose AUC with 80% power, assuming a one-sided test and a coefficient of variation of 18 percent[19].

11.2. Pharmacokinetics

Data for all subjects receiving a dose of active study drug will be included in the pharmacokinetic analysis. Pharmacokinetic parameters will be summarized and descriptive statistics (including means, median, standard deviations and coefficients of variation) will be generated for each dose. Dose proportionality will be assessed graphically for PK parameters. The graphical assessment of dose proportionality will be performed for both single dose PK parameters (AUC_{24hr} and C_{max}) as well as steady state AUCss and Cmax,ss after multiple daily dosing. At each dose level, AUC_{24hr} and C_{max} for Days 1 and 16 will also be compared. In addition, the effect of dose and time on other pharmacokinetic parameters will also be explored, as appropriate.

11.3. Pharmacodynamic Analyses

Pharmacodynamic analyses will be performed on all subjects receiving at least one dose of JNJ-28431754 or placebo and with at least 1 pharmacodynamic assessment. Summary statistics will be generated for all pharmacodynamic parameters.

The primary pharmacodynamic assessments will focus on

Change from baseline mean 24-hour plasma glucose concentration on Days 1 and 16

Change from baseline urine glucose excretion (UGE) that include (a) cumulative amount over each 24 hours on Days 1, 2, 8, 12, 16, 17, 18, 19 with Day-1 values as baseline and (b) UGE rate, calculated as the UGE amount in each fraction divided by hours of each collection interval on Days 1, 2, 8, 12, 16, 17, 18, 19.

Mixed effect ANOVA modeling appropriate for this design will be used to assess the treatment effects on 24-hour mean plasma glucose and the UGE parameters (amount and rate). The mixed effect linear model will include dose and days of measurements as the independent variables. The estimated least-squares means and 95% confidence intervals for the pair-wise difference for different doses will be obtained.

Mixed effect ANOVA modeling will be used to investigate effect of JNJ-28431754 on 24-hour urine glucose excretion and rate of glucose excretion. The change from baseline for 0-24 hour amount of glucose excretion will be calculated and analyzed by fitting a mixed effect linear model will include dose and days of measurements as the independent variables. The estimated least-squares means and appropriate confidence intervals for the pair-wise difference of the mean change from baseline in 0-24 hour urine glucose for different doses will be obtained. Also, rate of excretion of urine glucose will be similarly analyzed using mixed effect ANOVA model and the estimated least-squares means and appropriate confidence intervals for the pair-wise difference of the mean rate of excretion of urine glucose for different doses will be reported.

The secondary pharmacodynamic assessments are provided in Section 9.3.2.

These secondary endpoints will be analyzed based on appropriate statistical models that will be detailed in the Statistical Analysis Plan for this protocol.

11.4. Safety Analyses

Safety from screening (i.e., signing informed consent) through follow-up will be evaluated by examining incidence, severity, relationship to study medication, and type of adverse events; changes in clinical laboratory

results; physical examination; vital signs measurements; ECG. Data will be summarized using descriptive statistics. Descriptive analysis will be performed for creatinine clearance, fractional and cumulative urinary excretion of electrolytes, amino acids, albumin, osmolality and tubular enzymes.

Adverse Events

The original terms used in the CRFs by investigators to identify adverse events will be coded using the Medical Dictionary for Regulatory Activities (MedDRA). The percentage of subjects with specific treatment-emergent adverse events will be summarized for each treatment group.

Special attention will be given to those subjects who have discontinued treatment due to an adverse event or who experienced a severe or a serious adverse event.

Clinical Laboratory Tests

Laboratory data will be summarized by the type of laboratory test. Normal reference ranges and markedly abnormal results (specified in the Statistical Analysis Plan) will be used in the summary of laboratory data. Descriptive statistics will be calculated for each laboratory analyte at baseline and at each scheduled time point. Changes from baseline results will be presented in pre- versus posttreatment cross tabulations (with classes for below, within, and above normal ranges). A listing of subjects with any laboratory results outside the reference ranges will also be provided.

Cardiovascular Safety

The effects on cardiovascular variables will be evaluated by means of descriptive statistics and frequency tabulations. These tables will include shifts from baseline values (the predose ECG will be used as baseline) to allow detection of relevant changes in individuals.

The ECG variables that will be analyzed are heart rate, PR interval, QRS interval, QT interval, and QT interval corrected for heart rate (QTcB and QTcF). Pulse and systolic blood pressure and diastolic blood pressure (supine and standing) will also be analyzed.

QTc values for the ECGs recorded during the study periods will be tabulated for their absolute values and also tabulated relative to baseline measurements, as listed below, in order to detect individual QTc changes.

- The number of male subjects with
  - QTc <430 msec
  - 430 ≤QTc ≤450 msec
  - QTc >450 msec
- The number of female subjects with
  - QTc <450 msec
  - 450 ≤QTc ≤470 msec
  - QTc >470 msec
- The number of subjects with QTc >500 msec
- The number of subjects with an increase of the QTc interval with respect to baseline
  - <30 msec
  - 30 to 60 msec
  - >60 msec

All important abnormalities from the ECG readings, including changes in T-wave morphology and/or the occurrence of U-waves versus baseline recordings, will be reported.

Vital Signs and Physical Examination

Descriptive statistics will be provided to evaluate the changes at each scheduled time point.

11.5. Interim Analyses

No formal Interim Analysis is planned, however, a blinded data review will occur after each tested dose for the dose-escalation decision.

If requested by J&JPRD Clinical Team, an internal Data Review Committee (DRC), independent from the Clinical Team, may be established by the Sponsor to review unblinded data to aid decision-making during this study regarding the development of the compound. The DRC would ensure that confidentiality of any unblinded information is maintained until the study is terminated and the database is locked. Any consequent changes after an unplanned formal Interim Analysis will be specified in an amendment to the protocol.

The Sponsor's staff on the Clinical Study team responsible for coordinating the drug-development program may be partially unblinded after the completion of a dose level or cohort. In these instances, the integrity of the study will be maintained by providing unblinded summary outputs only to those Sponsor staff responsible for coordinating the drug-development program. Those involved in contact with study subjects or responsible for collecting and cleaning the clinical data will not have access to the unblinded summary outputs.

12. ADVERSE EVENT REPORTING

Timely, accurate, and complete reporting and analysis of safety information from clinical studies are crucial for the protection of subjects, investigators, and the sponsor, and are mandated by regulatory agencies worldwide. The sponsor has established Standard Operating Procedures (SOPs) in conformity with regulatory requirements worldwide to ensure appropriate reporting of safety information; all clinical studies conducted by the sponsor or its affiliates will be conducted in accordance with those procedures.

12.1. Definitions

12.1.1. Adverse Event Definitions and Classifications

- **Adverse Event**
  An adverse event is any untoward medical occurrence in a clinical study subject administered a pharmaceutical product. An adverse event does not necessarily have a causal relationship with the treatment. An adverse event can therefore be any unfavorable and unintended sign (including an abnormal finding), symptom, or disease temporally associated with the use of a medicinal (investigational) product, whether or not related to the medicinal (investigational) product. (Definition per International Conference on Harmonisation [ICH])

  This includes any occurrence that is new in onset or aggravated in severity or frequency from the baseline condition, or abnormal results of diagnostic procedures, including laboratory test abnormalities.

  Note: The sponsor collects adverse events starting with the signing of the informed consent.

- **Serious Adverse Event**
  A serious adverse event as defined by ICH is any untoward medical occurrence that at any dose meets any of the following conditions:
  - results in death

- is life-threatening
  (The subject was at risk of death at the time of the event. It does not refer to an event that hypothetically might have caused death if it were more severe.)
- requires inpatient hospitalization or prolongation of existing hospitalization
- results in persistent or significant disability/incapacity, or
- is a congenital anomaly/birth defect

Note: Medical and scientific judgment should be exercised in deciding whether expedited reporting is also appropriate in situations other than those listed above. For example, important medical events may not be immediately life threatening or result in death or hospitalization but may jeopardize the subject or may require intervention to prevent one of the outcomes listed in the definition above. Any adverse event is considered a serious adverse event if it is associated with clinical signs or symptoms judged by the investigator to have a significant clinical impact.

- **Unlisted (Unexpected) Adverse Event**
  An unlisted adverse event, the nature or severity of which is not consistent with the applicable product information. For an investigational product, the expectedness of an adverse event will be determined by whether or not it is listed in the Investigator's Brochure.
- **Associated With the Use of the Drug**
  An adverse event is considered associated with the use of the drug if the attribution is possible, probable, or very likely by the definitions listed in Section 12.1.2.

12.1.2. Attribution Definitions

- **Not related**
  An adverse event that is not related to the use of the drug.
- **Doubtful**
  An adverse event for which an alternative explanation is more likely, e.g., concomitant drug(s), concomitant disease(s), or the relationship in time suggests that a causal relationship is unlikely.
- **Possible**
  An adverse event that might be due to the use of the drug. An alternative explanation, e.g., concomitant drug(s), concomitant disease(s), is inconclusive. The relationship in time is reasonable; therefore, the causal relationship cannot be excluded.
- **Probable**
  An adverse event that might be due to the use of the drug. The relationship in time is suggestive (e.g., confirmed by dechallenge). An alternative explanation is less likely, e.g., concomitant drug(s), concomitant disease(s).

- **Very likely**
  An adverse event that is listed as a possible adverse reaction and cannot be reasonably explained by an alternative explanation, e.g., concomitant drug(s), concomitant disease(s). The relationship in time is very suggestive (e.g., it is confirmed by dechallenge and rechallenge).

12.2. Procedures

12.2.1. All Adverse Events

All adverse events will be reported from the time a signed and dated informed consent form is obtained until completion of the last study-related procedure. Those meeting the definition of serious adverse events must be reported using the Serious Adverse Event Form, including serious adverse events spontaneously reported to the investigator within 30 days after the subject has completed the study (including poststudy follow up). The sponsor will evaluate any safety information that is spontaneously reported by an investigator beyond the time frame specified in the protocol.

All events that meet the definition of a serious adverse event will be reported as serious adverse events, regardless of whether they are protocol-specific assessments.

All adverse events, regardless of seriousness, severity, or presumed relationship to study therapy, must be recorded using medical terminology in the source document and the CRF. Whenever possible, diagnoses should be given when signs and symptoms are due to a common etiology (e.g., cough, runny nose, sneezing, sore throat, and head congestion should be reported as "upper respiratory infection"). Investigators must record in the CRF their opinion concerning the relationship of the adverse event to study therapy. All measures required for adverse event management must be recorded in the source document and reported according to sponsor instructions.

The sponsor assumes responsibility for appropriate reporting of adverse events to the regulatory authorities. The sponsor will also report to the investigator all serious adverse events that are unlisted and associated with the use of the drug. The investigator (or sponsor where required) must report these events to the appropriate Independent Ethics Committee/Institutional Review Board (IEC/IRB) that approved the protocol unless otherwise required and documented by the IEC/IRB.

12.2.2. Serious Adverse Events

All serious adverse events occurring during clinical studies must be reported to the appropriate sponsor contact person by investigational staff within 24 hours of their knowledge of the event.

Information regarding serious adverse events will be transmitted to the sponsor using the Serious Adverse Event Form, which must be signed by a member of the investigational staff. The initial report of a serious adverse event may be made by facsimile (fax) or telephone. It is preferable that serious adverse events be reported via fax. Subsequent to a telephone report of a serious adverse event, a Serious Adverse Event Form must be completed by the investigational staff and transmitted to the sponsor within 1 working day.

All serious adverse events that have not resolved by the end of the study, or that have not resolved upon discontinuation of the subject's participation in the study, must be followed until any of the following occurs:

– the event resolves
– the event stabilizes
– the event returns to baseline, if a baseline value is available
– the event can be attributed to agents other than the study drug or to factors unrelated to study conduct
– when it becomes unlikely that any additional information can be obtained (subject or health care practitioner refusal to provide additional information, lost to follow-up after demonstration of due diligence with follow-up efforts)

The cause of death of a subject in a clinical study, whether or not the event is expected or associated with the investigational agent, is considered a serious adverse event. Any event requiring hospitalization (or prolongation of hospitalization) that occurs during the course of a subject's participation in a clinical study must be reported as a serious adverse event, except hospitalizations for:

- social reasons in absence of an adverse event
- surgery or procedure planned before entry into the study (must be documented in the CRF)

12.2.3. Pregnancies

Subject pregnancy must be reported by the investigational staff within 1 working day of their knowledge of the event using the pregnancy notification form. Any subject who becomes pregnant during the study must be promptly withdrawn from the study.

Because the study drug may have an effect on sperm, pregnancies in partners of male subjects included in the study will be reported by the investigational staff within 1 working day of their knowledge of the event using the pregnancy notification form.

Follow-up information regarding the outcome of the pregnancy and any postnatal sequelae in the infant will be required.

12.3. Contacting Sponsor Regarding Safety

The names of the individuals (and corresponding telephone numbers) who should be contacted regarding safety issues or questions regarding the study are listed on the Contact Information page(s), which will be provided as a separate document.

13. STUDY DRUG INFORMATION

13.1. Physical Description of Study Drug(s)

JNJ–28431754 is a white powder with very low aqueous solubility. JNJ–28431754 and its matching placebo will be dispensed using the provided oral dispenser and should be stored between 2° and 8°C and protected from white light. All study medication should be stored in a secure area under restricted access.

Active compound:	JNJ-28431754
Dosage form:	Liquid, suspension of JNJ-28431754 in 0.5% hypromellose
Strength:	5 and 50 mg/mL
Placebo:	0.5% hypromellose

Detailed instructions for the study drug dispensing and the storage conditions will be provided to the clinical study site.

13.2. Packaging

The study drug will be packaged according to current good manufacturing practices and local regulations. The JNJ–28431754 and its matching placebo

will be provided as bulk supplies (i.e., not packaged by individual subject numbers) in conventional packaging (i.e., not child resistant packaging) containing appropriate volumes.

13.3. Labeling

Study drug labels will contain information to meet the applicable regulatory requirements. The site will also be provided with dispensing labels to be applied at the time of dose preparation.

13.4. Preparation and Handling

The study medication should be stored at 2°C to 8°C and protected from white light. Detailed instructions for the study drug administration will be provided along with the clinical supply shipment.

13.5. Drug Accountability

The clinical investigator is responsible for ensuring that all study drug received at the site is inventoried and accounted for throughout the study. The dispensing of study drug to the subject, and the return of study drug from the subject (if applicable), must be documented on the drug accountability form. Subjects or their legally acceptable representative must be instructed to return all original containers, whether empty or containing study drug. Study drug returned by study subjects will be stored and disposed of according to the sponsor's instructions. Contents of the study drug containers must not be combined.

Study drug must be handled strictly in accordance with the protocol and the container label and will be stored in a limited access area or in a locked cabinet under appropriate environmental conditions. Unused study drug, and study drug returned by the subject (if applicable), must be available for verification by the sponsor's site monitor during on-site monitoring visits. The return to the sponsor of unused study drug, or used returned study drug for destruction, will be documented on the Drug Return Form.

Study drug should be dispensed under the supervision of the investigator, a qualified member of the investigational staff, or by a hospital/clinic pharmacist. Study drug will be supplied only to subjects participating in the study. Returned study drug must not be dispensed again, even to the same subject. Study drug may not be relabeled or reassigned for use by other

subjects. The investigator agrees neither to dispense the study drug from, nor store it at, any site other than the study sites agreed upon with the sponsor.

14. STUDY-SPECIFIC MATERIALS

The investigator will be provided with the following supplies:

- Oral solution dispenser
- Dispensing labels
- Randomization list (pharmacist only)
- Randomization code break envelopes
- Electronic CRFs (eCRF) and infrastructure
- PK sample labels

15. ETHICAL ASPECTS

15.1. Study-Specific Design Considerations

This is the first study with JNJ-28431754 in subjects with T2DM. JNJ-28431754 is an investigational drug that is currently being developed for the treatment of T2DM (see Section 1, Introduction).

Each subject must give written consent according to local requirements after the nature of the study has been fully explained. The consent form must be signed before performance of any study-related procedure. Subjects will be informed that their participation is voluntary, that they will derive no direct medical benefit from participating and that they may withdraw consent to participate at any time. Subjects will be fully informed of any new information as it becomes available throughout the course of the study as this may affect their decision to continue participation.

After initial screening, eligible subjects will discontinue their previous anti-diabetic medications starting 16 days prior to dosing on Day 1 and through to Day 22. Blood glucose levels are expected to rise modestly in many subjects, but fasting and post lunch blood glucose levels will be monitored at least daily throughout this study, and fasting and postprandial blood glucose levels will not be allowed to exceed approximately 270 mg/dL (15 mM) and 400 mg/dL (22.2 Mm), respectively. It will be possible to rapidly resume anti-diabetic therapy, if needed. While chronically elevated (for years) and/or uncontrolled hyperglycemia in T2DM patients can lead to serious diabetic complications, short-term discontinuation of oral anti-diabetic medications (for a maximum of approximately 8 weeks in this study) with modest blood

glucose elevations and with daily medical monitoring is not expected to result in clinically meaningful short- or long-term consequences for the study subjects.

The available data from the single ascending dose study with JNJ-28431754 in healthy male subjects has provided adequate safety, tolerability, pharmacokinetic and pharmacodynamic information to justify cautious and well controlled administration of single and multiple ascending doses in Type 2 diabetic subjects. The study design for this first administration of repeated doses in T2DM subjects takes a careful approach involving escalation of the 14-day repeated dosing only after review of preliminary safety and tolerability data from a single dose administration at each dose level. Subject safety will be closely monitored particularly in relation to adverse effects observed in preclinical testing and in the first-in-human study, especially body fluid and electrolyte balance, skin reaction and GI tolerability. In addition, preliminary data from a 3-month GLP toxicology study in rats and dogs will be available after the initiation of the present study. If necessary, modifications in doses and study procedures to the present study will be made in response to new toxicity data from the 3-month rat toxicology study.

Type 2 diabetes affects both men and women, and thus evaluating the safety, tolerability and effects of JNJ-28431754 in women is desirable for further developing this new agent. In the present study, it is planned to include post-menopausal or surgically sterile women. Based on the safety, tolerability and exposure data from the ongoing FIH study in healthy male subjects, it is justified to include women in this study in the planned dose range.

Subjects in this study may be taking other medications, especially anti-dislipidemic and hypertension drugs, which poses a potential risk of drug-drug interactions with JNJ-28431754. Based on preclinical data, the drug has a low potential for CYP related DDIs. To minimize the risk, medications that have a higher likelihood of interacting with JNJ-28431754 (based on information on JNJ-28431754 to date and the mechanism of action) have been excluded as co-medications during the study. These include diuretics, beta-blockers (which can mask symptoms of hypoglycemia) and CYP 3A4 inhibitors and inducers as JNJ-28431754 is at least partially metabolized by CYP3A4 (Attachment 8). Since the potential for, and magnitude of,

interactions in vivo via CYP3A4 is not clear at this time, a cautious approach to exclude these co-medications is warranted. The potential for other drug-drug interactions does exist, and this will be closely monitored for during the study.

The total volume of blood drawn for laboratory evaluations throughout this study is approximately 482 mL for each subject and 518 mL for each subject participating in the potential daily twice-daily cohort. An additional 10 mL of blood will be collected once from subjects who consent to genetic testing. This volume is considered to be acceptable in an 8-week trial. Subjects will be advised not to donate blood for at least 90 days after completion of the study.

Subjects safety will be closely monitored by clinical and laboratory assessments. Particular attention will be paid to the potential human organ toxicity based on animal toxicology studies and the ongoing FIH study, ie, body fluid and electrolyte balance, blood glucose levels and GI tolerability.

Subjects will be closely monitored for potentially drug-induced hypoglycemia, by frequent assessment of blood glucose levels and careful monitoring for signs and symptoms of hypoglycemia. A bedside glucose measurement using a glucose analyzer will be performed at scheduled time points and on an as needed basis when subjects develop symptoms suggestive of hypoglycemia. Grading and Treatment of Hypoglycemic Events are included in Attachment 7.

In the in vivo phototoxicity study in rats, JNJ-28431754 showed mild phototoxicity to skin, but not to eyes. Subjects will be instructed to avoid direct sunlight, or any artificial intense light, especially UV light, up to 96 hours (or at least 5 estimated half lives of the drug) post the last drug administration, or if sunlight exposure cannot be avoided, to use protective clothing and broad-spectrum (UVA/UVB) sunscreens and sun block lipstick that have a skin protection factor of at least 15.

As with all safety, tolerability, PK and PD studies, there are risk/discomforts associated with various study procedures. The insertion of the catheter and the venipuncture may cause brief pain, light bleeding and possibly a minor infection. However, the discomforts and the potential risks associated with venipuncture are generally minimum and transient.

15.2. Regulatory Ethics Compliance

15.2.1. Investigator Responsibilities

The investigator is responsible for ensuring that the clinical study is performed in accordance with the protocol, current ICH guidelines on Good Clinical Practice (GCP), and applicable regulatory requirements.

GCP is an international ethical and scientific quality standard for designing, conducting, recording, and reporting studies that involve the participation of human subjects. Compliance with this standard provides public assurance that the rights, safety, and well being of study subjects are protected, consistent with the principles that originated in the Declaration of Helsinki, and that the clinical study data are credible.

15.2.2. Independent Ethics Committee or Institutional Review Board (IEC/IRB)

Before the start of the study, the investigator (or sponsor where required) will provide the IEC/IRB with current and complete copies of the following documents:

- final protocol and, if applicable, amendments
- sponsor-approved informed consent form (and any other written materials to be provided to the subjects)
- Investigator's Brochure (or equivalent information) and amendments
- sponsor-approved subject recruiting materials
- information on compensation for study-related injuries or payment to subjects for participation in the study, if applicable
- investigator's curriculum vitae or equivalent information (unless not required, as documented by IEC/IRB)
- information regarding funding, name of the sponsor, institutional affiliations, other potential conflicts of interest, and incentives for subjects
- any other documents that the IEC/IRB requests to fulfill its obligation

This study will be undertaken only after IEC/IRB has given full approval of the final protocol, amendments (if any), the informed consent form, applicable recruiting materials, and subject compensation programs, and the sponsor has received a copy of this approval. This approval letter must be dated and must clearly identify the documents being approved.

IEC/IRB approval for the pharmacogenomic research component of the clinical study and for the pharmacogenomic informed consent form must be

obtained. IEC/IRB approval can be obtained for the protocol independent of approval for pharmacogenomic research.

During the study the investigator (or sponsor where required) will send the following documents to the IEC/IRB for their review and approval, where appropriate:

- protocol amendments
- revision(s) to informed consent form and any other written materials to be provided to subjects
- if applicable, new or revised subject recruiting materials approved by the sponsor
- revisions to compensation for study-related injuries or payment to subjects for participation in the study, if applicable
- Investigator's Brochure amendments or new edition(s)
- summaries of the status of the study (at least annually or at intervals stipulated in guidelines of the IEC/IRB)
- reports of adverse events that are serious, unlisted, and associated with the investigational drug
- new information that may adversely affect the safety of the subjects or the conduct of the study
- deviations from or changes to the protocol to eliminate immediate hazards to the subjects
- report of deaths of subjects under the investigator's care
- notification if a new investigator is responsible for the study at the site
- any other requirements of the IEC/IRB

For protocol amendments that increase subject risk, the amendment and applicable informed consent form revisions must be submitted promptly to the IEC/IRB for review and approval before implementation of the change(s).

At least once a year, the IEC/IRB will be asked to review and reapprove this clinical study. This request should be documented in writing.

At the end of the study, the investigator (or sponsor where required) will notify the IEC/IRB about the study completion.

15.2.3. Informed Consent

Each subject must give written consent according to local requirements after the nature of the study has been fully explained. The consent form must be signed before performance of any study-related activity. The consent form that is used must be approved by both the sponsor and by the reviewing IEC/IRB. The informed consent should be in accordance with principles that originated in the Declaration of Helsinki, current ICH and GCP guidelines, applicable regulatory requirements, and sponsor policy.

Before entry into the study, the investigator or an authorized member of the investigational staff must explain to potential subjects the aims, methods, reasonably anticipated benefits, and potential hazards of the study, and any discomfort it may entail. Subjects will be informed that their participation is voluntary and that they may withdraw consent to participate at any time. They will be informed that choosing not to participate will not affect the care the subject will receive for the treatment of his/her disease. Subjects will be told that alternative treatments are available if they refuse to take part and that such refusal will not prejudice future treatment. Finally, they will be told that the investigator will maintain a subject identification register for the purposes of long-term follow-up if needed and that their records may be accessed by health authorities and authorized sponsor staff without violating the confidentiality of the subject, to the extent permitted by the applicable law(s) or regulations. By signing the informed consent form the subject is authorizing such access, and agrees to be recontacted after study completion, by health authorities and authorized sponsor staff, for the purpose of obtaining consent for additional safety evaluations if needed.

The subject will be given sufficient time to read the informed consent form and the opportunity to ask questions. After this explanation and before entry into the study, consent should be appropriately recorded by means of the subject's dated signature. After having obtained the consent, a copy of the informed consent form must be given to the subject.

Subjects will also be asked to consent to participate in a pharmacogenomic research component of the study (where local regulations permit). After informed consent for the clinical study is appropriately obtained, the subject will be asked to sign and personally date a separate pharmacogenomic informed consent form indicating agreement to participate in optional

pharmacogenomic research. A copy of the signed pharmacogenomic informed consent form will be given to the subject.

If the subject or legally acceptable representative is unable to read or write, an impartial witness should be present for the entire informed consent process (which includes reading and explaining all written information) and should personally date and sign the informed consent form after the oral consent of the subject or legally acceptable representative is obtained.

15.2.4. Privacy of Personal Data

The collection and processing of personal data from subjects enrolled in this study will be limited to those data that are necessary to investigate the efficacy, safety, quality, and utility of the investigational product(s) used in this study.

These data must be collected and processed with adequate precautions to ensure confidentiality and compliance with applicable data privacy protection laws and regulations.

The sponsor ensures that the personal data will be

- processed fairly and lawfully
- collected for specified, explicit, and legitimate purposes and not further processed in a way incompatible with these purposes
- adequate, relevant, and not excessive in relation to said purposes
- accurate and, where necessary, kept current

Explicit consent for the processing of personal data will be obtained from the participating subject (or his/her legally acceptable representative) before collection of data. Such consent should also address the transfer of the data to other entities and to other countries.

The subject has the right to request through the investigator access to his/her personal data and the right to request rectification of any data that are not correct or complete. Reasonable steps should be taken to respond to such a request, taking into consideration the nature of the request, the conditions of the study, and the applicable laws and regulations.

Appropriate technical and organizational measures to protect the personal data against unauthorized disclosures or access, accidental or unlawful

destruction, or accidental loss or alteration must be put in place. Sponsor personnel whose responsibilities require access to personal data agree to keep the identity of study subjects confidential.

For those subjects who gave consent to store DNA samples for future research (Part 2), samples and corresponding relevant clinical data will undergo a procedure to make the samples and data non-identifiable, which involves removal of personal identifiers. Samples will be stored indefinitely until exhausted. Future genetic research will be restricted to that which is related to the drug or the indications for which the drug is developed. For subjects submitting a written request for their data generated on identifiable samples (Part 1), the sponsor will provide the raw data but cannot undertake to make decisions regarding the significance of any findings resulting from this pharmacogenomic research, and cannot, therefore, undertake to provide any genetic counselling. Genotypic data generated on non-identifiable samples (Part 2) cannot be returned to individual subjects.

16. ADMINISTRATIVE REQUIREMENTS

16.1. Protocol Modifications

Neither the investigator nor the sponsor will modify this protocol without a formal amendment. All protocol amendments must be issued by the sponsor, and signed and dated by the investigator. Protocol amendments must not be implemented without prior IEC/IRB approval, or when the relevant competent authority has raised any grounds for non-acceptance, except when necessary to eliminate immediate hazards to the subjects, in which case the amendment must be promptly submitted to the IEC/IRB and relevant competent authority. When the change(s) involves only logistic or administrative aspects of the study, the IRB (and IEC where required) only needs to be notified.

In situations requiring a departure from the protocol, the investigator or other physician in attendance will contact the appropriate sponsor representative by fax or telephone (see Contact Information pages provided separately). If possible, this contact will be made before implementing any departure from the protocol. In all cases, contact with the sponsor must be made as soon as possible in order to discuss the situation and agree on an appropriate course of action. The data recorded in the CRF and source document will reflect any

departure from the protocol, and the source documents will describe this departure and the circumstances requiring it.

16.2. Regulatory Documentation

16.2.1. Regulatory Approval/Notification

This protocol and any amendment(s) must be submitted to the appropriate regulatory authorities in each respective country, if applicable. A study may not be initiated until all local regulatory requirements are met.

16.2.2. Required Prestudy Documentation

The following documents must be provided to the sponsor before shipment of study drug to the investigational site:

- protocol and amendment(s), if any, signed and dated by the investigator
- a copy of the dated and signed written IEC/IRB approval of the protocol, amendments, informed consent form, any recruiting materials, and if applicable, subject compensation programs. This approval must clearly identify the specific protocol by title and number and must be signed by the chairman or authorized designee.
- name and address of the IEC/IRB including a current list of the IEC/IRB members and their function, with a statement that it is organized and operates according to GCP and the applicable laws and regulations. If accompanied by a letter of explanation from the IEC/IRB, a general statement may be substituted for this list. If an investigator or a member of the investigational staff is a member of the IEC/IRB, documentation must be obtained to state that this person did not participate in the deliberations or in the vote/opinion of the study.
- regulatory authority approval or notification, if applicable
- signed and dated statement of investigator (e.g., Form FDA 1572), if applicable
- documentation of investigator qualifications (e.g., curriculum vitae)
- completed investigator financial disclosure form from the investigator
- signed and dated clinical trial agreement, which includes the financial agreement
- any other documentation required by local regulations

The following documents must be provided to the sponsor before enrollment of the first subject:

- completed investigator financial disclosure forms from all subinvestigators

- documentation of subinvestigator qualifications (e.g., curriculum vitae)
- photocopy of the site signature log, describing delegation of roles and responsibilities at the start of the study
- name and address of any local laboratory conducting tests for the study, and a dated copy of current laboratory normal ranges for these tests
- local laboratory documentation demonstrating competence and test reliability (e.g., accreditation/license), if applicable.

16.3. Subject Identification Register and Subject Screening Log

The investigator agrees to complete a subject identification register to permit easy identification of each subject during and after the study. This document will be reviewed by the sponsor site contact for completeness.

The subject identification register will be treated as confidential and will be filed by the investigator in the trial center file. To ensure subject confidentiality, no copy will be made. All reports and communications relating to the study will identify subjects by initials and assigned number only.

The investigator must also complete a subject-screening log, which reports on all subjects who were seen to determine eligibility for inclusion in the study.

16.4. Case Report Form Completion

CRFs are provided for each subject in printed or electronic format.

Electronic Data Capture (EDC) will be used for this study. The majority of the study data will be transcribed by study personnel from the source documents onto an electronic CRF and transmitted in a secure manner to the sponsor. The electronic file will be considered as the CRF.

All data relating to the study must be recorded in CRFs prepared by the sponsor. Data must be entered into CRFs in English. The CRFs are to be completed at the time of the subject's visit, with the exception of results of tests performed outside the investigator's office, so that they always reflect the latest observations on the subjects participating in the study.

Every effort should be made to ensure that all subjective measurements (e.g., pain scale information or other questionnaires) to be recorded on the CRF

are completed by the same individual who made the initial baseline determinations. The investigator must verify that all data entries in the CRFs are accurate and correct.

All CRF entries, corrections, and alterations must be made by the investigator or other authorized study-site personnel.

16.5. Data Quality Assurance

Steps to be taken to ensure the accuracy and reliability of data include the selection of qualified investigators and appropriate study centers, review of protocol procedures with the investigator and associated personnel before the study, periodic monitoring visits by the sponsor, and direct transmission of clinical laboratory data from a central laboratory into the sponsor's data base. Written instructions will be provided for collection, preparation, and shipment of blood, plasma, and urine samples. CRF completion guidelines will be provided and reviewed with study personnel before the start of the study. The sponsor will review CRFs for accuracy and completeness during on-site monitoring visits and after their return to the sponsor; any discrepancies will be resolved with the investigator or designee, as appropriate. The data will be entered into the clinical study database and verified for accuracy.

16.6. Record Retention

In compliance with the ICH/GCP guidelines, the investigator/institution will maintain all CRFs and all source documents that support the data collected from each subject, as well as all study documents as specified in ICH/GCP Section 8, Essential Documents for the Conduct of a Clinical Trial, and all study documents as specified by the applicable regulatory requirement(s). The investigator/institution will take measures to prevent accidental or premature destruction of these documents.

Essential documents must be retained until at least 2 years after the last approval of a marketing application in an ICH region and until there are no pending or contemplated marketing applications in an ICH region or until at least 2 years have elapsed since the formal discontinuation of clinical development of the investigational product. These documents will be retained for a longer period if required by the applicable regulatory requirements or by an agreement with the sponsor. It is the responsibility of

the sponsor to inform the investigator/institution as to when these documents no longer need to be retained.

If the responsible investigator retires, relocates, or for other reasons withdraws from the responsibility of keeping the study records, custody must be transferred to a person who will accept the responsibility. The sponsor must be notified in writing of the name and address of the new custodian. Under no circumstance shall the investigator relocate or dispose of any study documents before having obtained written approval from the sponsor.

For CRFs completed on NCR paper, one copy is to be retained in the archives of the sponsor from the country in which the study is performed. A second copy must be archived by the investigator.

If it becomes necessary for the sponsor or the appropriate regulatory authority to review any documentation relating to this study, the investigator must permit access to such reports.

16.7. Monitoring

The sponsor will perform on-site monitoring visits as frequently as necessary. The monitor will record dates of the visits in a study center visit log that will be kept at the site. The first post-initiation visit will usually be made as soon as possible after enrollment has begun. At these visits, the monitor will compare the data entered into the CRFs with the hospital or clinic records (source documents). The nature and location of all source documents will be identified to ensure that all sources of original data required to complete the CRF are known to the sponsor and investigational staff and are accessible for verification by the sponsor site contact. If electronic records are maintained at the investigational site, the method of verification must be discussed with the investigational staff. At a minimum, source documentation must be available to substantiate: subject identification, eligibility, and participation; proper informed consent procedures; dates of visits; adherence to protocol procedures; records of safety and efficacy parameters; adequate reporting and follow-up of adverse events; administration of concomitant medication; drug receipt/dispensing/return records; study drug administration information; and date of subject completion, discontinuation from treatment, or withdrawal from the study, and the reason if appropriate. Specific items required as source documents will be reviewed with the investigator before the study.

If data are recorded directly into the CRF, at a minimum there should be an entry in the medical record that each of the assessments was done, and by whom and the date it was done. It is recommended that the author of an entry in the source documents be identifiable.

Direct access to source documentation (medical records) must be allowed for the purpose of verifying that the data recorded in the CRF are consistent with the original source data. Findings from this review of CRFs and source documents will be discussed with the investigational staff. The sponsor expects that, during monitoring visits, the relevant investigational staff will be available, the source documentation will be available, and a suitable environment will be provided for review of study-related documents. The monitor will meet with the investigator on a regular basis during the study to provide feedback on the study conduct.

If corrections to a CRF are needed after removal of the original CRF copy from the investigational site, a DCF will be used.

16.8. Study Completion/Termination

16.8.1. Study Completion

The study is considered completed with the last visit of the last subject undergoing the study. The final data from the investigational site will be sent to the sponsor (or designee) no more than 24 hours following completion of the final subject visit at that site.

16.8.2. Study Termination

The sponsor reserves the right to close the investigational site or terminate the study at any time. Investigational sites will be closed upon study completion. An investigational site is considered closed when all required documents and study supplies have been collected and a site closure visit has been performed.

The investigator may initiate site closure at any time, provided there is reasonable cause and sufficient notice is given in advance of the intended termination.

Reasons for the early closure of an investigational site by the sponsor or investigator, or termination of a study by the sponsor, may include but are not limited to:

- failure of the investigator to comply with the protocol, the sponsor's procedures, or GCP guidelines
- safety concerns
- sufficient data suggesting lack of efficacy
- inadequate recruitment of subjects by the investigator

16.9. On-Site Audits

Representatives of the sponsor's clinical quality assurance department may visit the site to conduct an audit of the study in compliance with regulatory guidelines and company policy. These audits will require access to all study records, including source documents, for inspection and comparison with the CRFs. Subject privacy must, however, be respected.

Similar auditing procedures may also be conducted by agents of any regulatory body reviewing the results of this study in support of a regulatory submission. The investigator should immediately notify the sponsor if they have been contacted by a regulatory agency concerning an upcoming inspection.

16.10. Use of Information and Publication

All information, including but not limited to information regarding JNJ-28431754 or the sponsor's operations (e.g., patent application, formulas, manufacturing processes, basic scientific data, prior clinical data, formulation information) supplied by the sponsor to the investigator and not previously published, and any data including pharmacogenomic research data generated as a result of this study, are considered confidential and remains the sole property of the sponsor. The investigator agrees to maintain this information in confidence and use this information only to accomplish this study, and will not use it for other purposes without the sponsor's prior written consent.

The investigator understands that the information developed in the clinical study will be used by the sponsor in connection with the continued development of JNJ-28431754, and thus may be disclosed as required to other clinical investigators or regulatory agencies. To permit the information derived from the clinical studies to be used, the investigator is obligated to provide the sponsor with all data obtained in the study.

The results of the study will be reported in a Clinical Study Report generated by the sponsor and will contain all data from all investigational sites. Results of any pharmacogenomic analyses performed after the Clinical Study Report has been issued will be reported in a separate report and will not require a revision of the Clinical Study Report. Study subject identifiers will not be used in publication of pharmacogenomic results. Any work created in connection with performance of the study and contained in the data that can benefit from copyright protection (except any publication by the investigator as provided for below) shall be the property of the sponsor as author and owner of copyright in such work.

The sponsor shall have the right to publish such data and information without approval from the investigator. If an investigator wishes to publish information from the study, a copy of the manuscript must be provided to the sponsor for review at least 60 days before submission for publication or presentation. Expedited reviews will be arranged for abstracts, poster presentations, or other materials. If requested by the sponsor in writing, the investigator will withhold such publication for up to an additional 60 days to allow for filing of a patent application. In the event that issues arise regarding scientific integrity or regulatory compliance, the sponsor will review these issues with the investigator. The sponsor will not mandate modifications to scientific content and does not have the right to suppress information. The investigator will recognize the integrity of a multicenter study by not publishing data derived from the individual site until the combined results from the completed study have been published in full, within 12 months after conclusion, abandonment, or termination of the study at all sites, or the sponsor confirms there will be no multicenter study publication. Authorship of publications resulting from this study will be based on generally accepted criteria for major medical journals.

17. REFERENCES

1. Leahy J et al b-cell dysfunction induced by chronic yperglycemia: current ideas on mechanism of impaired glucose-insulin insulin secretion. Diabetes Care (1992); 15:442-455.

2. Rossetti L et al. Glucose toxicity. Diabetes Care (1990); 13:610-630.

3. Gaede P, Vedel P, Larsen N, et al. Multifactorial intervention and cardiovascular disease in patients with type 2 diabetes. N. Eng. J. Med (2003), 348:383-393.

4. The Diabetes Control and Complications Trial Research Group: The relationship of glycemic exposure (HbA1c) to the risk of development and progression of retinopathy in the Diabetes Control and Complications Trial. Diabetes (1995); 44:968-993

5. The Diabetes Control and Complications Trial Research Group: Effect of intensive diabetes therapy on the development and progression of diabetic nephropathy in the Diabetes Control and Complications Trial. Kidney Int. (1995); 47:1703-1720

6. The Diabetes Control and Complications Trial Research Group: The effect of intensive diabetes therapy on the development and progression of neuropathy in the Diabetes Control and Complications Trial. Ann Intern Med (1995); 122:561-568

7. UK Prospective Diabetes Study (UKPDS) Group. Intensive blood-glucose countol with sulphonylrueas or insulin compared with conventional treatment and risk of complications in patients with Type 2 diabetes (UKPDS 33) Lancet; (1998) 352:837-853

8. Stratton IM et al Association of glycemia with macrovascular and microvascular domplications of Type 2 diabetes (UKPDS 35) BMJ; 2000 321(7258):405-412

9. Wright EM. Renal Na+-glucose cotransporters. Am J Physiol Renal Physiol 2001; 280:F10-18

10. Ehrenkranz et al. Phlorizin: a review Diabetes Metab Res Rew. 2005; 21:31-38

11. JNJ-28431754 Investigator's Brochure, April 2007, Johnson & Johnson Pharmaceutical Research and Development, L.L.C.

12. Flint A. et al. Reproducibility, power and validity of visual analogue scales in assessment of appetite sensations in single test meal studies. International Journal of Obesity. 2000; 24:38-48

13. Polidori D. SIARaA: A Robust Algebraic Method for Determining Insulin Sensitivity and Glucose Absorption Rates from Oral Glucose Tolerance Tests or Mixed Meals. Internal Report Johnson & Johnson Pharmaceutical Research and Development. December 12, 2006.

14. FDA Guidance for Industry and Reviewers: Estimating the safe starting dose in clinical trials for therapeutics in adult healthy volunteers (July 2005).

15. Lesley A et al. Assessing kidney function-measured and estimated glomerular filtration rate. N Engl J Med 2006; 354;2473-83.

16. Bazett HC. An analysis of the time-relationship of electrocardiograms. Heart 1919; 70:353-370.

17. Hodges M, Salerno D, Erlien D. Bazett's QT correction reviewed: evidence that a linear QT correction for heart rate is better. J Am Coll Cardiol 1983;1:694.

18. Sagie A, Larson MG, Goldberg RJ, Bengston JR, Levy D. An improved method for adjusting the QT interval for heart rate (the Framingham Heart Study). Am J Cardiol 1992;70:797-801.

19. EMEA (Human Medicines Evaluation Unit) Committee for Proprietary Medicinal Products (CPMP). The assessment of the potential for QT interval prolongation by non-cardiovascular medicinal products. 1996; 986.

20. Bo Ahrén, Eric Simonsson, et.al. Inhibition of Dipeptidyl Peptidase IV Improves Metabolic Control Over a 4- Week Study Period in Type 2 Diabetes, Diabetes Care, Vol. 25, No. 5, May 2002, pp 869-875.

21. Evidence-based nutrition principles and recommendations for the treatment and prevention of diabetes and related complications, Diabetes Care, 25:202-212.

ATTACHMENTS

Attachment 1:
Pharmacokinetic Sample Collection and Handling

Materials and Labeling

Blood will be collected in glass or polypropylene tubes containing the appropriate K3-EDTA anticoagulant. Resulting plasma samples must be stored in polypropylene storage tubes with polypropylene or polyethylene caps. No tubes with separation gel should be used.

Urine samples will be collected in polyethylene containers with polypropylene or polyethylene caps and stored in polypropylene tubes with polypropylene or polyethylene caps.

All tubes and containers will be labeled with preprinted labels. The preprinted information will include the study number, Case Report Form identification number (CRF i.d. #), treatment period, scheduled sampling day and time as stipulated in the flow chart, and the analyte name. No other information will be written on the labels.

Labels should be applied to the sample tubes as follows:

- Apply labels to the sample tubes so that they do not overlap and obscure any information. If possible expose an area between the 2 ends of the label to allow viewing of the contents of the tube.
- Do not alter the orientation of the label on the sample tube.
- Apply labels to all tubes in the same manner.

Preparation of Plasma Pharmacokinetic Samples

- Collect 3 mL of blood into the appropriate EDTA anticoagulant tube at each time point and place in ice until centrifuged.
- Record the exact date and time of sampling in the CRF.
- Gently invert the tubes 5 to 6 times to afford mixing, before processing.
- Centrifuge blood samples within 2 hours of collection in a clinical centrifuge (refrigerated) at 1000 g (about 2500–3000rpm) for 10 minutes to yield approximately 2 mL of plasma from each 4 mL whole blood sample.
- Plasma will be harvested by pipetting two approximately equal aliquots (“A” and “B”) into two separate pre-labeled screw-cap polypropylene tubes. The caps will be securely tightened and the samples will be frozen and stored upright immediately at -20 °C or below.

- The time between blood collection and freezing the plasma will not exceed 2 hours.
- Ship specimens according to the instructions provided. Aliquot A shipped to bioanalytical site. Aliquot B stored at clinical site until the end of of each cohort, and then transferred to PCPK/MET group at J&JPRD Raritan, NJ, USA for potential metabolite identification.
- Repeat or unscheduled samples may be taken if required for safety reasons.
- If a cannula is used for blood sample collection, a small amount of blood will be discarded each time a sample is taken via the cannula.
- Questions regarding handling the plasma pharmacokinetic specimens should be addressed to the contact person for the sponsor.

Preparation of Urine Pharmacokinetic Samples

- Collect total urine in the appropriate urine collection container for the time periods listed.
- Thoroughly mix the total urine collected during that time period.
- Measure and record the volume of the total urine output for that time period in the CRF or laboratory requisition form.
- Transfer two 10 mL aliquots (or as near to 10 mL as possible if the urine volume for the collection period is low) from each pooled collection, “A” and “B” samples, into two appropriately labeled urine storage tubes.
- Store the urine samples in an upright position, at –18°C or lower until transfer. Ship specimens according to the instructions provided.
- Ship specimens according to the instructions provided. Aliquots A shipped to J&J PRD Spring House, PA USA, aliquot B stored at clinical site until the end of study, and then transferred to PCPK/MET group in Raritan, NJ, USA for potential metabolite identification.

Questions regarding handling the urine pharmacokinetic samples should be addressed to the contact person for the sponsor.

Attachment 2:
Labeling Instructions for Pharmacokinetic Samples

STRUCTURE OF THE LABEL:
1. Each label has 2 identical parts. Please complete all of the requested information (current date, time of sample, etc.), on both label parts.
2. The backing of the label and the label itself are perforated.

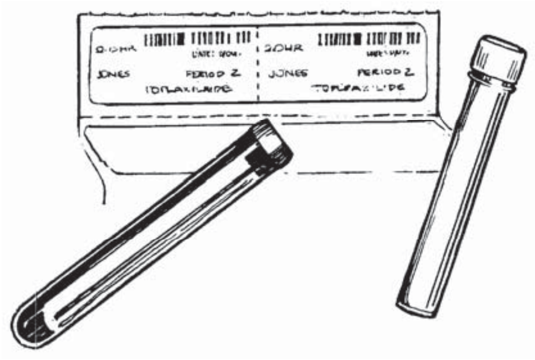

STEP 1: After the Vacutainer® has been filled, remove part 1 of the label and completely attach it to the Vacutainer®, lengthwise. Centrifuge the Vacutainer®.

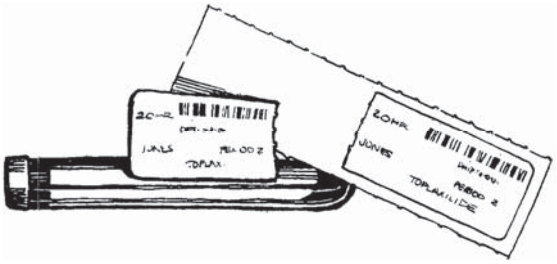

STEP 2: Remove part 2 of the label and attach it to the empty plasma collecting tube. The label must be attached to the tube lengthwise. You are now able to match the alphanumeric code, subject identification, and time point on the plasma collecting tube with the corresponding information on the Vacutainer®. After the tubes are matched, the plasma may be transferred from the Vacutainer® to the plasma collecting tube.

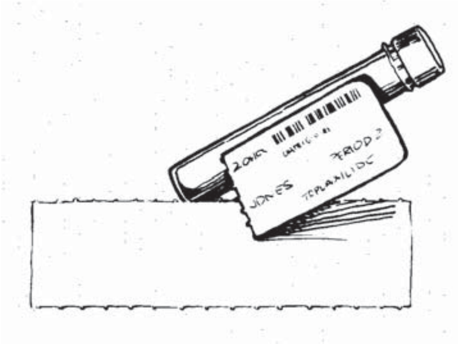

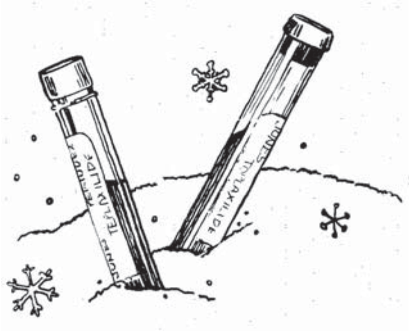

STEP 3: Freeze the sample in an upright position. Pack and ship the samples as instructed in the protocol.

Attachment 3:
Shipment of Pharmacokinetic Samples

All pharmacokinetic samples will be sent in multiple shipments as agreed upon with bioanalytical facility. An inventory list must be included with each shipment. The sponsor provides logs can be used as an inventory list. The inventory list must note each specimen drawn for each subject, and note any missing specimens.

The Study Investigator must follow the instructions below:

- For all shipments, Marken or World Courier will be used.
- The sponsor contact will be notified by FAX, that a shipment of samples is imminent. This notification will be made before the shipping date.
- Notify Johnson & Johnson Pharmaceutical Research and Development and Marken at least 24 hours in advance of the planned shipment. Provide World Courier with the appropriate account number to be used, if applicable.
- Double-bag the frozen samples for each subject in bags that can withstand dry ice conditions (e.g. cryogenic bags), and label with Subject i.d. #.
- Pack samples in divided grid boxes and secured with tapes
- Pack the frozen samples in sufficient quantity of dry ice in appropriate containers, to maintain a frozen state for at least 3 days.
- Avoid direct contact between sample bags and dry ice by separating them with a dry ice resistant material (e.g., newspaper).
- For all biological samples, follow the IATA regulations for shipment.
- Ensure that the total package weight does not exceed 40 pounds.
- Label the package with the sponsor name and study number.
- Include a return address (which includes the investigator’s name) on the outside of each shipping container.
- Comply with all courier regulations for the shipment of biological specimens (include all paperwork).
- Retain all documents indicating date, time and signature/s of person/people making the shipment, in the study files.

Attachment 3 (continued)
Shipment of Pharmacokinetic Samples

As soon as shipment day and air bill number are available, the site will call and fax Ms. Heather Hoffman, J&JPRD (contact information provided below). The call or fax must specify the study number, number of packages shipped, the number of pharmacokinetic samples, and the time of shipment pick-up.

PK plasma and PK urine aliquots "A" must be sent to:

Heather Hoffman
J&J PRD
Welsh and McKean Roads
Spring House PA 19477-0776
Tel: 215-540-4926
Fax: 215-540-4603

PK plasma and urine aliquots "B" must be sent to:

Dr. H.K. Lim
Johnson & Johnson PRD
1001 North Rt. #202
OCD Bldg., K 007
Raritan, NJ 08869
Tel: 908-218-6355
Fax: 908-541-0422
Email: hlim5@prdus.jnj.com

NOTE: If there are changes regarding the courier or location to which samples are shipped during the course of the clinical study, written notification will be provided to the Investigator and will not require a protocol amendment.

Attachment 4:
Pharmacogenomic Sample Collection and Shipment Procedure

Pharmacogenomic Sample Supplies and Labeling

The Study Investigator will use appropriately labeled 10 ml blood collection tubes containing potassium or sodium EDTA. Specimen labels shall be pre-printed with the following information: i) the specimen type (e.g., “pharmacogenomic sample”), ii) the trial number, and iii) Case Report Form identification number (CRF i.d.). No other information will be written on the labels. The Pharmacogenomics Department does not have the logistics to provide labels.

Preparation of Pharmacogenomic Samples

Pharmacogenomic samples should be prepared as follows:

– Invert the tube 10 to 15 times immediately after collection, to prevent coagulation.
– DO NOT centrifuge the sample.
– Blood samples collected and shipped within 24 hours and can be shipped at ambient temperature (see sample shipment below).
– When there is a delay of more than 24 hours between collection and shipment, samples should be stored at 4ºC at the investigational site for at most 3 calendar days, and shipped in ambient or cooled condition (but not on dry ice). Samples are not to be frozen.

Pharmacogenomic Sample Shipment

– All pharmacogenomic samples will be sent to the Pharmacogenomics Department/Raritan, NJ.
– For international shipments, World Courier (telephone number: +32 2 712 5060) will be used. For domestic shipments, a reliable domestic courier, such as Federal Express, will be used.
– Notify courier, at least 24 hours in advance of the planned shipment. Provide the courier with the appropriate account number to be used, if applicable.
– On the day of shipment, fax the Sample Accountability Form (SAF) (available from the Pharmacogenomics Department) to the Pharmacogenomics Department in order to notify that a shipment of samples is imminent. The SAF must contain the courier name and tracking number.
– The SAF must be included in the shipping container.
– DO NOT package the samples in dry ice.
– Label the shipping container with all courier regulations for the shipment of biological specimens (include all paperwork).

- Include a return address (which includes the investigator's name) on the outside of each shipping container.
- Retain all documents indicating date, time and signature/s of person/people making the shipment, in the study files.
- Ship blood samples via courier, to the Pharmacogenomics Department

Ship pharmacogenomic samples to:

Dr. Stephan Francke
Johnson and Johnson Pharmaceutical Research & Development
Department of Pharmacogenomics
1000 Route 202
Raritan, NJ 08869
Phone 908 218 6596
FAX: 908 429 0695
sfranck1@prdus.jnj.com

NOTE: If there are changes regarding the courier or location to which samples are shipped during the course of the clinical study, written notification will be provided to the Investigator and will not require a protocol amendment.

Attachment 5:
Pharmacodynamic Sample Collection and Shipment Procedure

1. Materials and Labeling

Blood for pharmacodynamic assessments (2 mL for C-peptide, glucose and insulin) must be collected in the appropriate collection tubes. Resulting plasma samples must be stored in polypropylene storage tubes with polypropylene or polyethylene caps.

All tubes and containers will be labeled with preprinted labels. The preprinted information will include the study number, Case Report Form identification number (CRF i.d. #), treatment period, scheduled sampling day and time as stipulated in the flow chart, and the analyte name. No other information will be written on the labels.

- Pharmacodynamic samples for the assessment of glucose, insulin, and C-peptide must be labeled with a label from the Optical Mark (OMR) form.

Labels should be applied to the sample tubes as follows:

– Apply labels to the sample tubes so that they do not overlap and obscure any information. If possible, expose an area between the 2 ends of the label to allow viewing of the contents of the tube.

– Do not alter the orientation of the label on the sample tube.

– Apply labels to all tubes in the same manner.

2. Preparation of Plasma Pharmacodynamic Samples for Glucose, Insulin, and C-Peptide

– Collect 2 mL of blood into a 2.0-mL Vacutainer ® lithium heparin tube at each time point for the assessment of glucose, insulin, and C-peptide (see 3 a, b, and c.). If a cannula is used for blood sample collection, a small amount of blood will be discarded each time a sample is taken via the cannula.

– Record the exact date and time of sampling in the CRF.

– Gently invert the tubes 5 to 6 times to afford mixing, before processing.

– Centrifuge the tubes for 10 minutes at 1500 x g at Room temperature.

– Ship specimens to the laboratory according to Attachment 6. The samples will be analyzed on an ongoing base.

– Questions regarding handling the pharmacodynamic specimens should be addressed to the Sponsor contact person.

a. Determination of Glucose in human plasma samples by Hitachi Modular/Roche

Matrix:	Plasma
Anticoagulant:	Lithium heparin
Preservatives:	None
Method:	hexokinase/glucose-6-P-dh

Sample collection: To prepare plasma samples, whole blood is directly drawn into a 2.0 mL Vacutainer ® lithium heparin tube. Centrifuge at 1500 x g for 10 minutes at room temperature. Samples will be analyzed on an ongoing base.

b. Determination of Insulin in human plasma samples by Hitachi Modular/Roche

Matrix: Plasma
Anticoagulant: Lithium heparin
Preservatives: None
Method: ECLIA
Sample collection: To prepare plasma samples, whole blood is directly drawn into a 2.0 mL Vacutainer® lithium heparin tube. Centrifuge at 1500 x g for 10 minutes at room temperature. Samples will be analyzed on a ongoing base

c. Determination of C-peptide in human plasma samples by Hitachi Modular/Roche

Matrix: Plasma
Anticoagulant: Lithium heparin
Preservatives: None
Method: ECLIA
Sample collection: To prepare plasma samples, whole blood is directly drawn into a 2.0 mL Vacutainer ® lithium heparin tube. Centrifuge at 1500 x g for 10 minutes at room temperature. Samples will be analyzed on an ongoing base

3. Preparation of Plasma Pharmacodynamic Samples for <u>GLP-1</u>

– Collect 3 mL of blood into the appropriate collection tube at each time point for GLP- (see 3.a.) If a cannula is used for blood sample collection, a small amount of blood will be discarded each time a sample is taken via the cannula.
– Record the exact date and time of sampling in the CRF.
– Gently invert the tubes 5 to 6 times to afford mixing, before processing.
– Centrifuge the tubes for 10 minutes at 1500 x g at 4°C
– Ship specimens to the laboratory according to instructions below.
– Questions regarding handling the pharmacodynamic specimens should be addressed to the Sponsor contact person.

a. Determination of GLP-1 in human plasma samples by ELISA

Matrix: Plasma
Anticoagulant: EDTA
Preservatives: DPP IV inhibitor
Sample collection: To prepare plasma samples, 3 ml whole blood is directly drawn into an ice-cooled 3.0 ml Vacutainer ® P700 V1.0 K_2 EDTA plasma tube containing 5.4 mg DPP4 inhibitor (BD; Cat# 366473). Invert tube to mix and store tubes in ice bath. Centrifuge immediately at 1000 x g for 10 minutes in refrigerated centrifuge or place tubes on ice and centrifuge within one hour. Store at –70°C. Samples must be shipped on dry ice.

Attachment 6:
Shipment of Pharmacodynamic Samples

All Glucose, Insulin, and C-peptide pharmacodynamic samples, urine glucose and urine electrolyte pharmacodynamic samples and bone specific alkaline phosphatase, serum osteocalcin, urine deoxypyridinolines and urine NTX-1 will be sent to the Medizinische Laboratorien Marienhof, in multiple shipments as agreed upon with J&JPRD. An inventory list must be included with each shipment. The inventory list must note each specimen drawn for each subject, and note any missing specimens.

Have the samples double-bagged in trays that can withstand dry ice conditions, and label with Subject's CRF i.d. #

The study coordinator must notify the Laboratory of the planned shipment.

Samples must be sent to:

Dr. Rudi Reinards
Medizinische Laboratorien Marienhof
Wallstr. 10
41061 Mönchengladbach
Germany
Tel. 49 2161 8194 416/410
email: RReinards@mlm-marienhof.de

Labeling of GLP-1 samples –

Please follow labeling instructions provided in Attachment 2. Watson labels will be provided.

Shipment of GLP-1 samples –

Please follow shipping instructions provided in Attachment 3.

As soon as shipment day and air bill number are available, the site will call and fax Ms. Cianna Cooper, J&JPRD (contact information provided below). The call or fax must specify the study number, number of packages shipped, the number of GLP-1 samples, and the time of shipment pick-up.

GLP-1 plasma samples must be sent to:

Cianna Cooper
Johnson & Johnson PRD
1000 Route 202 S
OMP Research, B-364
Raritan, NJ 08869
Tel: 908-704-5852
Fax: 908-218-0973
Email: ccooper5@prdus.jnj.com

NOTE: If there are changes regarding the courier or location to which samples are shipped during the course of the clinical study, written notification will be provided to the Investigator and will not require a protocol amendment.

Attachment 7:
Grading and Treatment of Hypoglycemic Events

Definition

Hypoglycemia is defined as one the following:

Characteristic symptoms of hypoglycemia (e.g., chills, sweats, shakes, tachycardia, withdrawn attitude) with no blood glucose check. Clinical picture must include prompt resolution with food intake, subcutaneous glucagons, or intravenous glucose.

Characteristic symptoms of hypoglycemia with blood glucose check showing glucose 59 mg/dL (3.2 mM) or less. Symptoms associated with a blood glucose of 60 mg./dL (3.3 mM) or greater cannot be reported as hypoglycemia.

Any glucose measurement 49 mg/dL (2.7 mM) or less, with or without symptoms.

Severity:

Every hypoglycemic event must be characterized with respect to its severity. In order to characterize the event as severe, all of the following criteria must be met:

- The subject was unable to treat himself or herself.
- The subject exhibited at least one of the following neurological symptoms:

Memory loss
Confusion
Uncontrollable behavior
Irrational behavior
Unusual difficulty in awakening
Suspected seizure
Seizure
Loss of consciousness

- Also, one of the following:

If blood glucose was measured and was 49 mg/dL or less or,
If the blood glucose was not measured, the clinical manifestations were revered by oral carbohydrates, subcutaneous glucagons, or intravenous glucose
Events that do not meet all 3 criteria for sever hypoglycemia are characterized as mild to moderate.

Monitoring and treatments

All subjects will be closed monitored. A bedside glucose measurement using a Glucometer will be performed at scheduled time points and on an as needed basis when subjects develop symptoms suggestive of hypoglycemia. For any subject whose blood glucose level is < 50 mg/dL, bedside glucose will be measured every 15 to 30 minutes until the blood glucose concentration is > 50 mg/dL.

Orange juice should be given to a subject with glucose < 50 mg/dL and with symptoms of hypoglycemia. Orange juice will automatically be given to any subject with a glucometer reading < 40mg/dL

Attachment 8:

Standard Meals

Standardized total daily calories based on daily energy expenditure on Days -3 to 20 (in-patient setting):

- 2300 kcal

BMI-Adjustment

BMI 19 - <25	approx. 2100-2400 kcal
BMI 25 - <30	approx. 2401-2700 kcal
BMI 30 - <35	approx. 2701-2900 kcal
BMI > =35	approx. 2901-3200 kcal

The standard meals used for this study will be in accordance with the recommendations of the American Diabetes Association (American Diabetes Association: Evidence-based nutrition principles and recommendations for the treatment and prevention of diabetes and related complications,[21] which foresee 50-60% of calorie intake to come from carbohydrates, 15-20% from protein and 25-35% from fat.

− Breakfast (or dinner for BID dosing) within 10 minutes after dosing
Approximately 30% of total calorie intake
− Lunch at 4.5 hours after dosing
Approximately 35% of total calorie intake
− Dinner at 10.5 hours after dosing
Approximately 35% of total calorie intake

The three meals served on Days -1, 1 and 16 will be of the exact same composition on all days for all subjects.

Subjects should finish each meal completely within 30 minutes. Subjects should be encouraged to complete all their standardized meals.

The menu of the standard meals will be prepared by a certified dietician at the study site according to the meal components as defined above and finalized with a joint agreement between the PI and the sponsor.

Attachment 9:
Medications with Potential for CYP3A4 Inhibition and/or Induction Properties

Medications with Potential for CYP3A4 Inhibition*

3A4,5,7
HIV Antivirals:
delaviridine
indinavir
nelfinavir
ritonavir
amiodarone
aprepitant
NOT azithromycin
chloramphenicol
cimetidine
ciprofloxacin
clarithromycin
diethyl-dithiocarbamate
diltiazem
erythromycin
fluconazole
fluvoxamine
gestodene
grapefruit juice
itraconazole
ketoconazole
mifepristone
nefazodone
norfloxacin
norfluoxetine
mibefradil
star fruit
verapamil

*Does not include azithromycin

Medications with Potential for CYP3A4 Induction

3A,4,5,7
HIV Antivirals:
efavirenz
nevirapine
barbiturates
carbamazepine
glucocorticoids
modafinil
phenobarbital
phenytoin
rifampin
St. John's wort
troglitazone
oxcarbazepine
pioglitazone
rifabutin

Attachment 10:
Concomitant Medications

Anti-Hypertension Drugs

For subjects with controlled hypertension enrolled in this study, monotherapy with an Angiotensin Converting Enzyme Inhibitor (ACEI), or an Angiotensin-II Receptor Antagonist (ATII Antagonist), or a Calcium-Channel Blocker (CCB).

Diuretics and Beta-blockers are not allowed.

The following combination drug regimens are also allowed: ATII Ant + CCB, ACEI + CCB. For all anti-hypertensive drug regimens, the dose and frequency must be stable and unchanged for at least 3 months preceding study start. Specific allowed agents are listed below. Other agents may be allowable if agreed between the Principal Investigator and the Sponsor during screening.

Allowed ACE Inhibitors

Benazepril, Captopril, Enalapril, Fosinopril, Lisinopril, Moexipril, Perindopril, Quinapril, Ramipril, Trandolapril

Allowed ATIIR Antagonists

Candisartan, Eprosartan, Telmisartan, Valsartan

Allowed CCBs

Amlodipine, Felodipine, Isradipine, Nicardipine, Nifedipine, Nisolodipine

Combined ACE Inhibitor / CCB Formulations

Lotrel (benazepril / amlodipine); Lexxel (enalapril / felodipine); Ta

Allowed Dyslipidemic Agents

For subjects with common dyslipidemias who have been on a stable regimen for at least 3 months prior to screening of a statin, or a fibrate, or a statin and a fibrate, the drugs listed below can be continued during the study. Subjects who have received bile-acid binding resins (e.g., cholestyramine, colestipol, colesevelam), or nicotinic acid within 3 months of the study may not be enrolled.

HMG-CoA Reductase Inhibitors (“Statins”)

Atorvastatin (Lipitor), Lovastatin (Mevacor), Pravastatin (Pravachol), Rosuvastatin (Crestor), Simvastatin (Zocor)

Fibrates

Bezafibrate, Ciprofibrate, Clofibrate, Fenofibrate, Gemfibrozil

Cholesterol Transporter Inhibitors

Ezetimibe, Ezetimibe/Simvastatin combination (Vytorin)

OTHERS

Low dose acetylsalicylic acid (≤100 mg/day)

Attachment 11:
Visual Analog Scale Questionnaire for Appetite and Satiety

Instructions:

- Record the exact date and time in the CRF.
- Subjects should not be allowed to view scores from previous assessments.

How hungry do you feel?

I am not hungry at all	________________________	I have never been hungrier

How satisfied do you feel?

I am completely empty	________________________	I cannot eat another bite

How full do you feel?

Not at all full	________________________	Totally full

How much do you think you can eat?

Nothing at all	________________________	A lot

Attachment 12:
Patient Reported Outcomes Questionnaire

INSTRUCTIONS: Below is a list of symptoms. If you had the symptom during the last 24 hours, please check YES. If you did have the symptom, please check the box that tells us how much the symptom DISTRESSED or BOTHERED you.

**Check *all* the symptoms you have had during the last 24 hours**	**Yes (x)**	**→ → IF YES: How much did it DISTRESS or BOTHER you?**				
		**Not at all**	**A little Bit**	**Some-what**	**Quite a Bit**	**Very Much**
1. Frequent urination during the daytime hours						
2. An uncomfortable urge to urinate						
3. Waking up at night because you had to urinate						
4. Waking up at night for reasons other than need to urinate						

Note: The data collected will be analyzed and reported separately from the Clinical Study Report. The information reported on this questionnaire will not be considered to be adverse events.

LAST PAGE

Johnson & Johnson Pharmaceutical Research & Development*

Clinical Protocol

A DOUBLE-BLIND, RANDOMIZED, PLACEBO-CONTROLLED STUDY TO EVALUATE THE SAFETY, TOLERABILITY, PHARMACOKINETICS AND PHARMACODYNAMICS OF SINGLE AND MULTIPLE ASCENDING ORAL DOSES OF JNJ-28431754 IN TYPE 2 DIABETES MELLITUS PATIENTS

Protocol 28431754NAP1002; Phase I

JNJ-28431754

Eudract No. 2007-000711-28

Amendment DEU-5

* Johnson & Johnson Pharmaceutical Research & Development (J&JPRD) is a global organization that operates through different legal entities in various countries. Therefore, the legal entity acting as the sponsor for studies of Johnson & Johnson Pharmaceutical Research & Development may vary, such as, but not limited to Johnson & Johnson Pharmaceutical Research & Development L.L.C. or Janssen-Cilag International N.V. The term "sponsor" is used throughout the protocol to represent these various legal entities; the sponsor is identified on the Contact Information page that accompanies the protocol.

This study will be conducted under Food & Drug Administration IND regulations (CFR Part 312).

**Issue/Report Date:**	27 SEPTEMBER 2007
**Prepared by:**	Johnson & Johnson Pharmaceutical Research & Development, L.L.C.<br>Division of Janssen-Cilag Ltd.
**Department:**	Drug Development
**Document No.:**	EDMS-PSDB-6956965:6.0

LOCAL AMENDMENT TO EDMS-PSDB-6657018:2.0

Confidentiality Statement

The information in this document contains trade secrets and commercial information that are privileged or confidential and may not be disclosed unless such disclosure is required by applicable law or regulations. In any event, persons to whom the information is disclosed must be informed that the information is *privileged* or *confidential* and may not be further disclosed by them. These restrictions on disclosure will apply equally to *all* future information supplied to you that is indicated as *privileged* or *confidential*.

INVESTIGATOR AGREEMENT

I have read this protocol and agree that it contains all necessary details for carrying out this study. I will conduct the study as outlined herein and will complete the study within the time designated.

I will provide copies of the protocol and all pertinent information to all individuals responsible to me who assist in the conduct of this study. I will discuss this material with them to ensure that they are fully informed regarding the study drug and the conduct of the study.

Coordinating Investigator (where required):

Name (typed or printed): ____________________

Institution and Address: ____________________

Signature: ____________________ Date: ____________________
(Day Month Year)

Principal (Site) Investigator:

Name (typed or printed): ____________________

Institution and Address: ____________________

Signature: ____________________ Date: ____________________
(Day Month Year)

Sponsor's Responsible Medical Officer:

Name (typed or printed): Sue Sh[illegible]

Institution: Johnson & Johnson Pharmaceutical Research & Development, L.L.C. Division of Janssen-Cilag Ltd.

Signature: [signature] Date: 27 Sep 2007
(Day Month Year)

Note: If the address or telephone number of the investigator changes during the course of the study, written notification will be provided by the investigator to the sponsor, and a protocol amendment will not be required.

TABLE OF CONTENTS

PROTOCOL AMENDMENTS 6

SYNOPSIS 16

TIME AND EVENTS SCHEDULE 23

ABBREVIATIONS 26

1. INTRODUCTION 29
1.1. Background 30
1.1.1. Pre-clinical Data 30
1.2. Overall Rationale for the Study 49

2. OBJECTIVES 49

3. OVERVIEW OF STUDY DESIGN 50
3.1. Study Design 50
3.2. Study Design Rationale 53
3.2.1. Rationale for DNA Collection 58
3.3. Subject Safety and Stopping Criteria 59

4. STUDY POPULATION 60
4.1. General Considerations 60
4.2. Inclusion Criteria 60
4.3. Exclusion Criteria 61
4.4. Prohibitions and Restrictions 64

5. RANDOMIZATION AND BLINDING 65
5.1. Overview 65
5.2. Procedures 66

6. DOSAGE AND ADMINISTRATION 67

7. COMPLIANCE 68

8. CONCOMITANT THERAPY 68

9. STUDY EVALUATIONS 69
9.1. Study Procedures 69
9.1.1. Overview 69
9.1.2. Screening Phase 71
9.1.3. Predosing Phase 72
9.1.4. Double-Blind Dosing Phase 75
9.1.5. Outpatient Phase 78
9.1.6. Posttreatment Phase (Follow-Up) 79
9.2. Pharmacokinetic Evaluations 80
9.2.1. Sample Collection and Handling 80
9.2.1.1. Plasma: 80
9.2.1.2. Urine 81
9.2.2. Analytical Procedures 81
9.2.3. Pharmacokinetic Parameters 82
9.2.3.1. Plasma 82
9.2.3.2. Urine 83
9.3. Pharmacodynamic Evaluations 84

TABLE OF CONTENTS (CONTINUED)

9.3.1. Primary 84
9.3.2. Secondary: 84
9.4. Pharmacogenomics 86
9.4.1. Analyses Related to the Trial (Part A) 86
9.4.2. DNA Storage for Future Analyses (Part B) 87
9.5. Safety Evaluations 87
9.5.1. Adverse Events 87
9.5.2. Clinical Laboratory 88
9.5.3. Cardiac Monitoring 92
9.5.4. Vital Signs 93
9.5.5. Physical Examination 94
9.5.6. Monitoring Blood Glucose Levels 94
9.5.7. Monitoring for skin reactions 95

10. SUBJECT COMPLETION/WITHDRAWAL 95
10.1. Completion 95
10.2. Discontinuation of Treatment 95
10.3. Withdrawal From the Study 95

11. STATISTICAL METHODS 97
11.1. Sample Size Determination 97
11.2. Pharmacokinetics 97
11.3. Pharmacodynamic Analyses 97
11.4. Safety Analyses 98
11.5. Interim Analyses 100

12. ADVERSE EVENT REPORTING 101
12.1. Definitions 101
12.1.1. Adverse Event Definitions and Classifications 101
12.1.2. Attribution Definitions 102
12.2. Procedures 103
12.2.1. All Adverse Events 103
12.2.2. Serious Adverse Events 104
12.2.3. Pregnancies 105
12.3. Contacting Sponsor Regarding Safety 105

13. STUDY DRUG INFORMATION 105
13.1. Physical Description of Study Drug(s) 105
13.2. Packaging 105
13.3. Labeling 106
13.4. Preparation and Handling 106
13.5. Drug Accountability 106

14. STUDY-SPECIFIC MATERIALS 107

15. ETHICAL ASPECTS 107
15.1. Study-Specific Design Considerations 107
15.2. Regulatory Ethics Compliance 110
15.2.1. Investigator Responsibilities 110
15.2.2. Independent Ethics Committee or Institutional Review Board (IEC/IRB) 110
15.2.3. Informed Consent 112
15.2.4. Privacy of Personal Data 113

TABLE OF CONTENTS (CONTINUED)

16. ADMINISTRATIVE REQUIREMENTS 114
16.1. Protocol Modifications 114
16.2. Regulatory Documentation 115
16.2.1. Regulatory Approval/Notification 115
16.2.2. Required Prestudy Documentation 115
16.3. Subject Identification Register and Subject Screening Log 116
16.4. Case Report Form Completion 116
16.5. Data Quality Assurance 117
16.6. Record Retention 117
16.7. Monitoring 118
16.8. Study Completion/Termination 119
16.8.1. Study Completion 119
16.8.2. Study Termination 119
16.9. On-Site Audits 120
16.10. Use of Information and Publication 120

17. REFERENCES 122

ATTACHMENTS 124
Attachment 1: Pharmacokinetic Sample Collection and Handling 125
Attachment 2: Labeling Instructions for Pharmacokinetic Samples 127
Attachment 3: Shipment of Pharmacokinetic Samples 128
Attachment 4: Pharmacogenomic Sample Collection and Shipment Procedure 130
Attachment 5: Pharmacodynamic Sample Collection and Shipment Procedure 132
Attachment 6: Shipment of Pharmacodynamic Samples 134
Attachment 7: Grading and Treatment of Hypoglycemic Events 135
Attachment 8: Standard Meals 137
Attachment 9: Medications with Potential for CYP3A4 Inhibition and/or Induction Properties 138
Attachment 10: Concomitant Medications 139
Attachment 11: Visual Analog Scale Questionnaire for Appetite and Satiety 141
Attachment 12: Patient Reported Outcomes Questionnaire 142

LAST PAGE 142

PROTOCOL AMENDMENTS

Amendment DEU-5 (27 September 2007)
This amendment is considered to be **substantial** based on the criteria set forth in Article 10(a) of Directive 2001/20EC of the European Parliament and the Council of the European Union.

Applicable Section(s)	Text Changes (new text in **bold**; deleted text in ~~strikeout~~)	Description of Change / Rationale for Change
Synopsis; Time and Events Schedule; 9.1.3 Predosing; 9.1.4 Double-blind; 9.5.3 Cardiac Monitoring	Continuous Lead II ECG monitoring (telemetry) will be conducted from 30 minutes prior to dosing until ~~6~~ **8** hours post dosing on Days -1, 1, **6,** 8 and 15.	Plasma drug concentration data from early cohorts (30 and 100mg dose) indicates that peak plasma concentration is reached at 0.5 to 6 hours post dose (Tmax). Telemetry monitoring will be extended from 6 to 8 hours to cover the range of Tmax. In addition, telemetry monitoring on Day 6 will be added to provide additional cardiac safety assessment at steady state drug exposure.
Synopsis; 3.1 Study Design	An **additional** ~~fifth~~ cohort will assess the safety, tolerability and effects of multiple doses of JNJ-28431754 on male and female Asian subjects	Additional cohorts of subjects may be enrolled to assess safety, tolerability and pharmacodynamic effects at different dose levels.
Synopsis; Time and Events Schedule	Urine samples for analysis of JNJ-28431754 will be collected at ~~predose~~ 0-2, 2-4.5 (prior to lunch), 4.5-7, 7-10.5 (prior to dinner), 10.5-13, 13-24, 24-48, 48-72 and 72-96 hours post dose	Clarification that predose PK urine samples are not required for Day 16.
Synopsis	Orthostatic vital signs will be measured on Day –2 **and Day 1** to 20 at 4 and 8 hours post dose.	Change made to maintain consistency with the body of the protocol.
Synopsis	Body temperature will be measured at Screening, on the morning of Day –2 **and Day 1** to 20 and at the follow-up visit.	Change made to maintain consistency with the body of the protocol.
4.4 Prohibitions and Restrictions	Weight maintaining diet must be adhered to from 2 weeks prior to admission **(Day –3** ~~2~~**)**	Clarification that admission occurs on Day -3

SYNOPSIS (CONTINUED)

Amendment DEU-4 (30 August 2007)
This amendment is considered to be **non-substantial** based on the criteria set forth in Article 10(a) of Directive 2001/20EC of the European Parliament and the Council of the European Union, in that it does not significantly impact the safety or physical/mental integrity of subjects, nor the conduct, management, or scientific value of the study.

Applicable Section(s)	Text Changes (new text in **bold**; deleted text in ~~strikeout~~)	Description of Change / Rationale for Change
Synopsis; Time and Events Schedule; Abbreviations; 9.1.2 Screening; 9.1.3 Predosing; 9.1.4 Double-blind; 9.1.5 Outpatient; 9.1.6 Post Treatment; 9.5.2 Clinical Laboratory	**Serum 1,25-dihydroxy Vitamin D, and parathyroid hormone (PTH) will be assessed at screening and on Days -2, 5, 11, 17, 22 and at the Follow-up visit.**	Based on animal toxicology data, the study drug might affect bone metabolism and electrolyte balance in humans. In addition to the bone markers already defined in the protocol, these 2 additional blood tests will allow further evaluation of any potential effects on bone metabolism and electrolyte balance. Approximately 0.5 ml serum is needed for these 2 tests, and will be obtained as part of the clinical chemistry serum samples. Therefore, this change will not require additional blood volume.
9.1.4 Double-Blind dosing Phase; 9.5.6 Monitoring Blood Glucose Levels	Real time blood glucose concentrations will be determined prior to each standardized breakfast and 2 hours post each lunch using a glucose analyzer. **On Days -1, 1 and 16 the measurements will be performed prior to breakfast and 2 hours post each meal.**	Clarification of glucose measurement time points.

Amendment DEU-3 (20 Jul 2007)
This amendment is considered to be **substantial** based on the criteria set forth in Article 10(a) of Directive 2001/20EC of the European Parliament and the Council of the European Union

Applicable Section(s)	Text Changes (new text in **bold**; deleted text in ~~strikeout~~)	Description of Change / Rationale for Change
Synopsis; 3.1 Study Design; 5.2 Procedures	At least **10** ~~15~~ subjects are required to complete each treatment period prior to a decision to escalate to the next higher dose level. If	The original statement requiring 15 subjects in a cohort to complete, prior to a dose escalation decision, was based on a randomization block size of 20. With that block size, and a 16:4 ratio of treated:placebo subjects, there would

SYNOPSIS (CONTINUED)

	fewer than **10** ~~15~~ subjects complete any cohort, a separate Data Review Committee (DRC) may be convened to review unblinded data and to provide a recommendation regarding dose escalation.	be at least 1 placebo subject included in 16 or more completed subjects in a given cohort so that unblinding would not be necessary for a dose-escalation decision. However, because more than one study center will participate in this study, and not all subjects in a given cohort may be studied at the same center, a block size of 20 might allow one center to study a small number of subjects that did not include placebo subjects. Thus, reducing the randomization block size from 20 to 5, will allow each center to enroll at least 5 subjects that will include at least 1 placebo subject. As a result, reducing the block size to 5 will minimize a potential center effect for clinical safety assessments in each cohort, ie, this will avoid the possibility that for a given cohort, a small number of subjects that includes no placebo subjects are enrolled in a given site. With this change, dose escalation decisions must be based on a minimum of 8 completing subjects on treatment and 2 completing subjects on placebo.

SYNOPSIS (CONTINUED)

Synopsis; 3.1 Study Design; 3.2 Study Design Rationale	The maximum predicted total plasma JNJ-28431754 exposure (AUC_{0-24h} at steady-state) will not exceed the NOAEL established in the 2-week GLP toxicology study in rats (AUC_{0-24h}:~ 80 µg.h/mL; ~~Cmax: ~ 6 µg/mL~~), **based on the incidence of hyperostosis (minimal to mild changes in distal end of femur & proximal end tibia) at 150 mg/kg,** without an amendment to this protocol.	The original statement in this protocol regarding the drug exposure limit (specifying both Cmax and AUC at steady-state) was technically imprecise. In the first Phase I single dose study in healthy subjects (Study 28431754 NAP1001), the average Cmax value at the 600 mg dose was 5.9 ug/mL (slightly below the average Cmax at the NOAEL in the 14-day rat toxicology study). Due to normal, inter-subject PK variances around the mean, some subjects in that first study had Cmax values slightly higher than 6 ug/mL. In the current multiple-dose study, it is expected that some subjects receiving a 600 mg dose, the planned maximum dose, will also have steady-state Cmax values that slightly exceed 6 ug/mL, due to normal inter-subject PK variability. It should be noted that based on the PK data from the first single dose study (Study 28431754 NAP1001), the projected mean Cmax value at steady-state is not expected to be meaningfully different from the mean Cmax value following single dose administration.<br><br>Therefore, in this amended protocol, only the mean AUC value (~ 80 ug.h/mL) at the NOAEL of the 14-day rat toxicology study will be specified as the exposure limit. That NOAEL in the 14-day rat toxicology study was based on the incidence of hyperostosis (minimal to mild changes in distal end of femur & proximal end tibia) at the dose of 150 mg/kg/day. Although it is not clear that the hyperostosis observed in the 2-week rat toxicology study is Cmax or AUC related, Cmax values in some subjects that slightly exceed the NOAEL Cmax value during a 14-day short treatment period in the current study are not considered a clinical safety concern. In addition, biomarkers for bone turnover are being closely monitored in all cohorts of this study.
Synopsis; 3.1 Study Design; 3.2 Study Design Rationale	Although subjects of Asian ethnicity have not been explicitly enlisted for the	Adding one cohort of Asian subjects in this study allows for the exploration of drug effect (safety and efficacy) in Asian subjects. Results

SYNOPSIS (CONTINUED)

	current protocol, based on the likely study demographics, relatively few Asian subjects are likely to be enrolled in each cohort. Since the incidence of T2DM is rapidly increasing in Asia, this new drug may be developed for use in Asian countries. To support potentially larger and longer clinical trials in Asian diabetics, an additional cohort will be studied that is comprised exclusively of Asian diabetics to allow exploring safety and efficacy in this population. Based on preclinical data related to JNJ-28431754 metabolism and clearance mechanisms, the PK and metabolism profiles of JNJ-28431754 in Asian subjects are not expected to be markedly different from that in Caucasians or other ethnic groups.<br>The dose selected for this additional Asian cohort will be a dose that has been already evaluated in a prior cohort as a safe and potentially efficacious dose.	from this cohort will support further clinical development in this population. The dose level chosen for this Asian cohort will have been previously tested in an earlier cohort and considered to be well tolerated.
Synopsis, 9.1.3 Predosing Phase; 9.1.4 Double-Blind Dosing Phase; 9.3.2 Secondary	Administration of visual analogue scale (VAS) questionnaires to assess appetite and satiety at 5 minutes prior to breakfast, 30, 60, 90, 120, 150, 180, 210 and 240 minutes after **starting** breakfast, 5 minutes prior to lunch and immediately after lunch	Clarification that the post breakfast time points are to be measured from the start of breakfast.
9.1.4 Double-Blind dosing Phase; 9.5.6 Monitoring Blood Glucose Levels	Real time blood glucose concentrations will be determined prior to each standardized ~~meal~~ **breakfast** and 2 hours post each lunch using a glucose analyzer.	Clarification that glucose concentrations will be measured prior to breakfast and after lunch on Days -3 to 20.

SYNOPSIS (CONTINUED)

Attachment 8	BMI-Adjustment<br>BMI 19 - <25 approx. 2100-2400 kcal<br>BMI 25 - <30 approx. 240**1**-2700 kcal<br>BMI 30 - <35 approx. 270**1**-2900 kcal<br>BMI > =35 approx. 290**1**-3200 kcal	BMI and kcal figures adjusted to eliminate overlap between levels
Synopsis; 3.1 Study Design	Twice-daily dosing may also be evaluated in one or more of the cohorts or ~~one~~ additional cohort**s** of up to 20 subjects may be added.	Text changed to allow for the addition of more than one cohort and to maintain consistency within the protocol.
4.3 Exclusion criteria	Clinically significant abnormal values for hematology, coagulation, fasting clinical chemistry or urinalysis (elevated HbA1c, fasting blood glucose values or mild abnormality in lipid profiles are allowed for diabetic subjects) at screening or **Day -2** (Note: Subjects who have fasting triglyceride levels greater than 5.1 mmol/L are excluded)	Text changed to clarify the laboratory assessments will be performed on Day -2 not upon admission to the unit.
Synopsis; 9.3.2 Secondary Pharmacodynamic Evaluations	Plasma glucagon-like peptide-1 (GLP-1) levels (active and/or total on Days -1, 1 and ~~17~~ **16** at -0.5 predose and 0.5, 1, 1.5 and 2.0 postdose	Text changed to clarify an inconsistency.
4.3 Exclusion Criteria 13	Clinically significant abnormality in physical examination, vital signs, or 12-lead electrocardiogram (ECG) at screening or ~~admission~~ **Day -2**.	Text changed to clarify the ECG is performed on Day -1 not upon admission to the unit.
9.1.4 Double-blind Dosing Phase	~~"except item 1"~~	Text changed to clarify the inconsistencies regarding urine collection.
Synopsis; 9.3.2 Secondary Pharmacodynamic Evaluations	Renal threshold (Rt), which is defined as the plasma glucose concentration at which maximum urinary glucose reabsorption is achieved and above which glucose is excreted in the urine, will be determined on **Days –1, 1 and 16**.	Predefined time when the Rt will be determined has been added.
9.5.2 Clinical Laboratory	**GFR calculated using the MDRD equation: $GFR = 175 \times (\text{standardized Scr})^{-1.154} \times (\text{age})^{-0.203} \times 0.742$ (if the subject is female) or × 1.212 (if the subject is black). GFR is expressed in milliliters per minute per 1.73 m^2 and race is either black or not.**	The MDRD GFR equation revised to include the component for females
9.5.4 Vital Signs	**Orthostatic vital signs will also be measured in triplicate.**	Text added to clarify procedure.

SYNOPSIS (CONTINUED)

Attachment 8; 3.1 Study Design	**The standard meals used for this study will be in accordance with the recommendations of the American Diabetes Association (American Diabetes Association: Evidence-based nutrition principles and recommendations for the treatment and prevention of diabetes and related complications which foresee 50-60% of calorie intake to come from carbohydrates, 15-20% from protein and 25-35% from fat.**<br><br>– **Breakfast (or dinner for BID dosing) within 10 minutes after dosing Approximately 30% of total calorie intake**<br>– **Lunch at 4.5 hours after dosing Approximately 35% of total calorie intake**<br>– **Dinner at 10.5 hours after dosing Approximately 35% of total calorie intake**	Text added to clarify meal composition and BMI adjustment.
9.1.3 Predosing Phase	Urinary drug abuse, serum pregnancy and breath alcohol test **(-2 day window allowed)**	The window will allow the drug screen, pregnancy test and alcohol test to be performed earlier ensuring the results are available prior to Day 1 dosing.
9.1.3 Predosing Phase	~~One aliquot will be stored at 70 C as a backup.~~	The site is to follow local laboratory requirements
9.2.1.2 Urine	Day 16:<br>Urine samples for analysis of JNJ-28431754 and its metabolites concentration will be collected at ~~predose~~,	Text deleted to clarify that a predose urine PK sample is not required on Day 16
9.5.2 Clinical Laboratory	**Drug/Alcohol Screen:**<br>**A drug and alcohol screen will be performed at screening and Day -2.**	This text was missing from Section 9.5.2
9.5.2 Clinical Laboratory	**Serum glucose** replaced plasma glucose	Correction to clinical laboratory panel
9.5.2 Clinical Laboratory	24-hour creatinine clearance (CLcr) on Day –1, 3, 5, 8, 11, 14, **16**, 17, 18 and **19**	Text changed to clarify creatinine clearance calculation days
9.5.2 Clinical Laboratory	HbA1c **(screening only)**	Text added to clarify HbA1c is required at screening only
References	**Reference No. 13 will be changed to " Polidori D. SIARaA: A Robust Algebraic Method for Determining Insulin Sensitivity and Glucose Absorption Rates from Oral Glucose Tolerance Tests or Mixed Meals. Internal Report. Johnson**	Corrections made to reference list

SYNOPSIS (CONTINUED)

	**& Johnson Pharmaceutical Research and Development. December 12, 2006**<br><br>Reference No. 14 will be the previous No. 13<br><br>**Reference No. 15 will be changed to "Lesley A et al. Assessing kidney function - measured and estimated glomerular filtration rate. N Engl J Med 2006; 354:2473-83"**<br><br>References No 16-19 will be the previous 15-18 (for ECG references)<br><br>Reference No 20 will the previous No 19.<br>**Reference No. 21"Evidence-based nutrition principles and recommendations for the treatment and prevention of diabetes and related complications Diabetes Care 2002; 25:202-212"**	
Attachment 1	samples will be frozen and stored upright immediately at ~~-18~~ **-20** °C or below.	PK sample storage conditions clarified
Attachment 3	**Marken** or World Courier	Additional courier added
9.1.1 Overview; 15.1 Study Specific Design Considerations	**475 mL/511 mL**	Blood volumes corrected
9.1.3 Predosing Phase; 9.5.6 Monitoring Blood Glucose Levels	The subject will be instructed to report any signs or symptoms of hypoglycemia, fasting blood glucose values > 13.3 mM (240 mg/dL), post lunch values > 19.4 mM (350 mg/dL) and blood glucose values < **2.8 mM (50 mg/dL**) to the study center.	Text changed to maintain consistency with Attachment 7.
9.5.2 Clinical Laboratory	**Chloride** added and ~~total protein~~ deleted from renal safety assessments.	Text changed to maintain consistency.
Synopsis; 9.1.3 Predosing Phase; 9.1.4 Double-Blind Dosing Phase; 9.5.2 Clinical Laboratory	~~osmolarity~~ **osmolality**	Text corrected
Synopsis; 9.2.1.1 PK Evaluations	Reference to Day 3 deleted	This time point is the same as 48 hour postdose
9.1.3 Predosing Phase	Frequency of urination over each 24 hours ~~except Days -1, 1 and 16 when fractionated urine samples will be collected~~	Text changed to maintain consistency.
9.5.3 Cardiac monitoring; 9.5.4 Vital signs	**except at screening and the Follow-up visit**	Text added to maintain consistency with synopsis

SYNOPSIS (CONTINUED)

Amendment DEU-2 (2 Jul 2007)
This amendment is considered to be **substantial** based on the criteria set forth in Article 10(a) of Directive 2001/20EC of the European Parliament and the Council of the European Union.

Applicable Section(s)	Text Changes (new text in **bold**; deleted text in ~~strikeout~~)	Description of Change / Rationale for Change
Synopsis; 4.2 Inclusion Criteria No. 4	At screening: body mass index (BMI = weight in kg/height in m^2) of 20 to 39.9 kg/m^2; HbA1c of ≥ 7% and ≤ **8.5%** ~~10%~~	BfArM request
4.3 Exclusion Criteria No. 3	History of clinically significant diabetic complications, including: retinopathy **(fundus photography within 3 months of screening required)**, nephropathy **(GFR < 70 ml/min/1.73 m^2 based on the MDRD equation, or macro-albuminuria >200 mg/L)**, neuropathy, gastroparesis, or ketoacidosis	BfArM request
Synopsis; 3.1 Study Design; 3.2 Study Design Rationale; 3.3 Stopping Criteria; 4.3 Exclusion Criteria; 9.1.1 Overview; 9.1.3 Predosing Phase; 9.1.5 Outpatient Phase; 9.5.6 Monitoring Blood Glucose Levels; 10.2 Discontinuation from Treatment; 15.1 Ethical Aspects	Subjects will resume their pre-study oral anti-diabetic medication regimen upon completion of the out-patient visit on Day 22 or potentially at any earlier time during the study if their confirmed fasting blood glucose levels rise above **13.3 mM (240** ~~270~~ **mg/dL)** or **confirmed** postprandial glucose levels rise above **19.4 mM (350** ~~400~~ **mg/dL)** following lunch.	BfArM request

SYNOPSIS (CONTINUED)

Original Protocol issued 12 April 2007
Amendments are listed beginning with the most recent amendment.

Amendment DEU-1 (7 June 2007)

This amendment is considered to be **non-substantial** based on the criteria set forth in Article 10(a) of Directive 2001/20EC of the European Parliament and the Council of the European Union, in that it does not significantly impact the safety or physical/mental integrity of subjects, nor the conduct, management, or scientific value of the study

Applicable Section(s)	Text Changes (new text in **bold**; deleted text in ~~strikeout~~)	Description of Change / Rationale for Change
4.3 Exclusion criteria	Clinically significant abnormal values for hematology, coagulation, fasting clinical chemistry or urinalysis **(hemoglobin <8.4 mmol/L in males and <6.8 mmol/L in females; creatinine ≥ 110 μmol/L for males and ≥ 101 μmol/L for females; alanine aminotransferase (ALAT) and aspartate amino transferase (ASAT) ≥ 3 times upper normal limit;** elevated HbA1c, fasting blood glucose values or mild abnormality in lipid profiles are allowed for diabetic subjects) at screening or admission (Note: Subjects who have fasting triglyceride levels greater than 5.1 mmol/L are excluded)	Additional parameters added at the request of the Ethics Committee.

SYNOPSIS (CONTINUED)

A DOUBLE-BLIND, RANDOMIZED, PLACEBO-CONTROLLED STUDY TO EVALUATE THE SAFETY, TOLERABILITY, PHARMACOKINETICS AND PHARMACODYNAMICS OF SINGLE AND MULTIPLE ASCENDING ORAL DOSES OF JNJ-28431754 IN TYPE 2 DIABETES MELLITUS SUBJECTS

SYNOPSIS

JNJ-28431754 is a potent, selective, and reversible inhibitor of the renal sodium glucose co-transporter (SGLT2). It is being investigated for the treatment of Type 2 diabetes mellitus (T2DM).

OBJECTIVES:

To evaluate the safety, tolerability, pharmacokinetics (PK) and pharmacodynamics (PD) of JNJ-28431754 after single and multiple ascending oral doses of JNJ-28431754 in subjects with Type 2 diabetes mellitus (T2DM).

OVERVIEW OF STUDY DESIGN:*

This is a randomized, double-blind, placebo-controlled, single and multiple (14 days) ascending dose, parallel group study. Five cohorts of subjects with T2DM (20 subjects per cohort) will be studied. One dose level will be evaluated in each cohort.

Sixteen (16) subjects will be randomized to JNJ-28431754 and four (4) to matching placebo within each cohort. Doses planned for evaluation are 30, 100, 300 and 600 mg per day. An additional cohort of Asian subjects will also be evaluated at a dose level, which was previously tested in a prior cohort and considered to be well tolerated. Following initial screening (conducted within 35 days prior to Day -3 of the study), eligible subjects will be instructed to discontinue their previous antidiabetic medications for 16 days prior to dosing on Day 1. Blood glucose levels will be monitored daily during the 16-day washout period to ensure their blood glucose levels remain within a well-tolerated range. Eligible subjects will be admitted to the Clinical Research Unit (CRU) on Day – 3. On Days –2 and -1, all subjects will receive placebo once daily in a single-blind fashion (subjects blinded) and undergo baseline safety and PD assessments. Eligible subjects will then be randomized to a double-blind treatment with JNJ-28431754 or placebo. A single dose of study medication will be administered on Day 1, followed by periodic safety, PK and PD assessments for 48 hours. Daily dosing will resume on Day 3 and continue through Day 16. Each daily dose will be administered at approximately the same time each day (between 8 AM and 9 AM) followed by a standardized breakfast within 10 minutes after dosing. Standardized lunch and dinner will be provided at 4.5 and 10.5 hours post dose, respectively. Subjects will be discharged from the CRU on Day 20 (96 hours after last dosing) and return to the CRU for safety assessments and PK sample collections on the mornings of Days 21 to 22. Subjects will also return within 7-10 days following the Day 22 outpatient visit for a final Follow-up visit.

Subjects will resume their pre-study oral anti-diabetic medication regimen upon completion of the out-patient visit on Day 22 or potentially at any earlier time during the study if their confirmed fasting blood glucose levels rise above 13.3 mM (240 mg/dL) or confirmed postprandial glucose levels rise above 19.4 mM (350 mg/dL) following the lunch. Subjects who need to restart anti-diabetic medications during the study will stop receiving study medication but continue with study related safety assessments and PK samples (relative to predose time point only) for at least 3 days after their final dose. The Principal Investigator will determine the dose at which to reinitiate the subjects' prior anti-diabetic medications and any subsequent dose adjustments.

* This section of the protocol has been revised. Please refer to the section of this document titled PROTOCOL AMENDMENTS (Amendment DEU-5, 27 September 2007) for a detailed description of the specific changes.

SYNOPSIS (CONTINUED)

The selected dose level for each subsequent cohort defined in this protocol may be modified if indicated based on evaluation of the safety, tolerability, pharmacokinetics or pharmacodynamics of prior cohorts. Modifications of any next dose can be a de-escalation, a reduced escalation step, or an increased escalation step. Twice-daily dosing may also be evaluated in one or more of the cohorts or additional cohorts of up to 20 subjects may be added. For any dose chosen for a possible twice-daily dosing regimen, the maximum predicted total plasma JNJ-28431754 exposure (AUC_{0-24} hr at steady-state) will not exceed the NOAEL established in the 2-week GLP toxicology study in rats (AUC_{0-24h}: ~ 80 µg.h/mL) based on the incidence of hyperostosis (minimal to mild changes in distal end of femur & proximal end tibia) at 150 mg/kg, without an amendment to this protocol. Pharmacokinetic and pharmacodynamic sampling time points may be adjusted to accommodate a twice-daily dosing regimen, but the total blood volume per subject specified in this protocol will not be exceeded. For all cohorts, JNJ-28431754 dose levels that are predicted to produce steady state JNJ-28431754 exposures above the NOAEL in the 2-week GLP toxicology study in rats (AUC_{0-24h}: ~ 80 µg.h/mL) will not be exceeded without an amendment to this protocol.

Planned JNJ-28431754 Dose Levels

Cohort[1]	N				
1	16+4[2]	30 mg or placebo			
2	16+4[2]		100 mg or placebo[3]		
3	16+4[2]			300 mg or placebo[3]	
4	16+4[2]				600 mg or placebo[3]
5	16+4[2]				30, 100 or 300 mg or placebo[3]

[1] Initiation of dosing for each subsequent cohort at a higher dose level will be at least 17 days following the initiation of dosing in the prior cohort.

[2] Sixteen on JNJ-28431754; four on placebo

[3] The actual dose (mg) level selected for each cohort may be modified based on evaluation of preliminary safety, pharmacokinetic and pharmacodynamic data from previous cohorts.

An additional cohort will assess the safety, tolerability and effects of multiple doses of JNJ-28431754 on male and female Asian subjects at a dose level which was previously tested in an earlier cohort and considered to be well tolerated

Successive cohorts at a higher dose level will be started with at least a 17-day interval relative to the start of dosing in the preceding cohort, after review of adverse events, vital signs, electrocardiograms (ECGs) and laboratory tests of the preceding cohort collected up to and including Day 17 for preliminary safety data and up to Day 11 for PK data. Each subsequent escalated dose level shall be performed if, in the judgement of the Principal Investigator and the Sponsor's Safety Physician, the results of the safety analysis of the preceding dose level are satisfactory. At least 10 subjects are required to complete each treatment period prior to a decision to escalate to the next higher dose level. If fewer than 10 subjects complete any dose level, a separate Data Review Committee (DRC) may be convened to review unblinded data and to provide a recommendation regarding dose escalation.

STUDY POPULATION:

Five cohorts of 20 (total 100) male and post-menopausal or surgically sterilized female Type 2 diabetic subjects, age 25 to 65 years, body mass index (BMI) between 20.0 and 39.9 kg/m^2 inclusive, meeting all protocol inclusion/exclusion criteria will be enrolled in the study. Within each cohort at least 3 subjects will be female. No subject will participate in more than one cohort.

Female subjects must be of non-childbearing potential, i.e., either post-menopausal, or pre-menopausal with documented surgical sterilization. Post-menopausal is defined as either no menses for at least 18 months prior to study start, or as no menses for 6 to 18 months prior to the start of this study and plasma FSH ≥ 40 mlU/mL and

SYNOPSIS (CONTINUED)

estradiol ≤ 20 pg/mL in 3 separate measurements (at Screening, on Day -17, and on Day -2 (-2 day window allowed).

All subjects will have HbA1c levels at Screening within the range of 7.0 to 8.5% and will also have fasting blood glucose at Day –2 within the range: ≥7.8 mM and ≤13.3 mM (140-240 mg/dl). In addition, during the 16 day washout period prior to Day 1, subjects will monitor their blood glucose levels daily and no subject will continue in the study if, during the washout period, fasting blood glucose levels are confirmed to rise above 13.3 mM (240 mg/dL) or postprandial glucose levels are confirmed to rise above 19.4 mM (350 mg/dL) following lunch.

Subjects must have been diagnosed with Type 2 diabetes at least 12 months prior to study start and will have been stably managed for at least 3 months prior to the Screening visit on metformin, or a sulfonylurea, or a meglitinide (e.g., repaglininide or nateglinide), or a DPP-IV inhibitor (e.g., sitagliptin or vildagliptin), or on an alpha-glucosidase inhibitor (e.g., Acarbose). Subjects will not have used any of the following anti-diabetic medications within 3 months of study start: exenatide, thiazolidinediones (e.g., rosiglitazone, or pioglitazone), or insulin.

Subjects with hypertension will be allowed in this study if their blood pressure has been successfully treated on a stable anti-hypertensive drug regimen for at least 3 months prior to study start with allowed anti-hypertensive medications (See Attachment 10), and there is no expectation that their anti-hypertensive medication regimen would change during the study. Subjects with dyslipidemias may be allowed in this study if they have been on a stable regimen of commonly prescribed dyslipidemic agents (e.g., statins, fibrates: See Attachment 10) for at least 3 months prior to this study, and their dyslipidemic drug regimen is not expected to change during this study. No subjects requiring concomitant medications for the continued treatment of obesity will be enrolled. The Principal Investigator and the Sponsor will review the inclusion of subjects on other medications (including non-prescription, herbal remedies or other over-the-counter agents) on a case-by-case basis. No alcohol consumption will be allowed from 72 hours prior to dosing until completion of the follow-up visit.

Eligible subjects for this study will return for an outpatient visit around Day -17, to be counseled by a registered dietician to follow a weight maintaining diet for approximately 2 weeks prior to admission and throughout the entire study except Days –3 to 20 when subjects will receive standardized meals (See Attachment 8). (The weight maintaining diet will follow standard recommendations of the American Diabetes Association, comprising ~55-60% carbohydrate, 10-20% protein, and ~30% fat, with total daily caloric intake individually adjusted for BMI). In addition, subjects will receive instruction in signs and symptoms of hypoglycemia, use of a portable home fingerstick blood glucose analyzer and reporting of results. During the washout phase and during the post-treatment phase (between discharge and prior to the Follow-up visit), subjects will record their fasting blood glucose levels and the postprandial blood glucose levels after lunch on a diary card which will be provided to them and report the results to the clinic weekly, or at any time values exceed prespecified limits. This information will be collected by the clinic and recorded in the eCRF.

DOSAGE AND ADMINISTRATION:

Compound:	JNJ-28431754
Strength:	5 and 50 mg/mL
Dosage form:	liquid, suspension of JNJ-28431754 in 0.5% hypromellose
Placebo:	0.5% hypromellose

PHARMACOKINETIC EVALUATIONS:

Day 1:

Plasma samples for measurement of JNJ-28431754 concentration will be collected at predose, 0.5, 1, 1.5, 2, 2.5, 3, 4, 6, 8, 10, 12, 24 and 48 hours postdose (total 14 samples).

Sample collection times for later cohorts may be modified as preliminary pharmacokinetic data becomes available during the study from initial cohorts. If a twice-daily dosing regimen is evaluated, 6 additional PK plasma samples may be collected at 10.5 (prior to dinner), 13, 14, 16, 19 and 22 hours after the morning dose. However, for all dose levels or dosing regimens, the total blood volume collected from each subject for all analyses (PK, PD, safety) will not exceed 550 mL over 8 weeks in this study.

Urine samples for measurement of JNJ-28431754 concentrations will be collected at predose, 0-2, 2-4.5 (prior to lunch), 4.5-7, 7-10.5 (prior to dinner), 10.5-13, 13-24 and 24-48 hours post dose.

Days 3 to 15:

SYNOPSIS (CONTINUED)

Blood samples will be collected at pre-dose in the morning on Days 5, 7, 9, 11, 13 and 15 to obtain trough JNJ-28431754 concentrations (total 6 samples)

Day 16:

Plasma samples for measurement of JNJ-28431754 concentration will be collected at predose, 0.5, 1, 1.5, 2, 2.5, 3, 4, 6, 8, 10, 12, 24, 48, 72, 96, 120 and 144 hours postdose (total 18 samples). If a twice-daily dosing regimen is evaluated, 6 additional PK plasma samples may be collected at 10.5 (prior to dinner), 13, 14, 16, 19 and 22 hours post the morning dose.

Sample collection times for later cohorts may be modified as preliminary pharmacokinetic data becomes available during the study from initial cohorts, but the total blood volume collected from each subject for all analyses (PK, PD, safety) will not exceed 550 mL over 8 weeks in this study.

Plasma and urine samples will be stored for potential identification/analysis of JNJ-28431754 metabolites or concomitant medication pharmacokinetic analysis and will be reported separately.

Urine samples for analysis of JNJ-28431754 will be collected at 0-2, 2-4.5 (prior to lunch), 4.5-7, 7-10.5 (prior to dinner), 10.5-13, 13-24, 24-48, 48-72 and 72-96 hours post dose.

Volumes will be recorded and aliquots taken after mixing for storage at -20°C and subsequent assay. Aliquots will also be taken for assay of glucose, electrolytes and creatinine (See below)

PHARMACODYNAMIC EVALUATIONS:

Primary:

1. Change from baseline for mean 24 hour plasma glucose concentration on Days 1 and 16, which is defined as the area under the plasma glucose concentration time curve over 0-24 hours, divided by 24 hours (Glucose AUC 0-24/24h). Glucose AUC 0-24/24h determined on Day –1 will be used as the baseline values

2. Change from baseline urine glucose excretion (UGE)

    a. Cumulative amount (grams of glucose) over each 24 hours on Days 1, 2, 8, 12, 16, 17, 18, 19; cumulative amount over 24 hours on Day –1 will be used as the baseline value

    b. UGE rate (grams of glucose per hour), defined as the UGE amount in each urine collection fraction, divided by the hour duration of each collection interval on Days 1, 2, 8, 12, 16, 17, 18, 19

Secondary:

1. Renal threshold (Rt), which is defined as the plasma glucose concentration at which maximum urinary glucose reabsorption is achieved and above which glucose is excreted in the urine will be determined on Days -1, 1 and 16. The Rt is calculated as a function of time for each subject based on measured plasma glucose, urinary glucose excretion, and glomerular filtration rate (GFR) (See Section 9.3.2)
2. Change from baseline mean 24 hour insulin concentration on Days 1 and 16, which is defined as the area under the plasma insulin time concentration time curve at 0-24 hours divided by 24 hours (Insulin AUC 0-24/24h). Insulin AUC 0-24/24h determined on Day –1 will be used as the baseline value
3. Changes from baseline morning fasting plasma glucose (FPG) and insulin (FPI) on Days 1 and 17. FPG and FPI values determined on Day –1 will be used as the baseline values. FPG and FPI on Days –1, 1 and 16 will be calculated by averaging predose values at –30, -15 and 0 minutes. On Days 2 to 15 and 17 to 20, one FPG and FPI will be determined within 30 minutes prior to dosing on dosing days or prior to breakfast on non-dosing days.
4. Change from baseline plasma glucose and insulin excursions: after each breakfast, defined as the difference between the maximum glucose and insulin values observed during the 4-h post meal period minus the mean of the premeal measurements at –30, -15 and 0 min on Days 1 and 16. Glucose and insulin excursions on Day –1 will be used as the baseline value.
5. Glucose AUC 0-2 on Days 1, and 16; Glucose AUC 0-2 on Days –1 will be used as the baseline value.

SYNOPSIS (CONTINUED)

6. Insulin sensitivity (See Section 9.3.2)
7. Beta-cell function (See Section 9.3.2)
8. Plasma glucagon-like peptide-1 (GLP-1) levels (active and/or total on Days -1, 1 and 16 at -0.5 predose and 0.5, 1, 1.5 and 2.0 postdose
9. Assessment of Visual Analogue Scale (VAS) questionnaires to assess appetite and satiety at 5 minutes prior to breakfast, 30, 60, 90, 120, 150, 180, 210 and 240 minutes after starting breakfast, 5 minutes prior to lunch and immediately after lunch on Days –2, 3, 7, 11 and Day 15. The VAS questionnaire schedule may be modified if a twice-daily dosing regimen is tested.
10. Morning fasting body weight will be assessed at Screening and on Days -1 through 20 and at the Follow-up visit using a calibrated scale.

Blood samples for plasma glucose, insulin and C-peptide will be collected on Day –1 at T-0.5, T-0.25, T0, T0.5, T1, T1.25, T1.5, T2.0, T2.5, T3.0, T4.5 (prior to lunch), T5.0, T5.5, T6.0, T6.5, T7.0, T8.0, T9.0, T10.5 (prior to dinner), T11, T12.0, T12.5, T13.0, T14.0 T16.0, T19.0, T22.0, and T24 hours where T0 corresponds to the dosing time on dosing days, and on Day 1 and 16 at -0.5, -0.25, 0, 0.5, 1, 1.25, 1.5, 2.0, 2.5, 3.0, 4.5 (prior to lunch), 5.0, 5.5, 6.0, 6.5, 7.0, 8.0, 9.0, 10.5 (prior to dinner), 11, 12.0, 12.5, 13.0, 14.0 16.0, 19.0, 22.0, and 24 hours postdose.

Morning fasting plasma glucose, insulin and C-peptide concentrations will be obtained daily on Days 2 to 15 and 17 to 20 within 30 minutes prior to dosing on dosing days or prior to breakfast on non-dosing days.

Blood samples for glucagon-like peptide-1 (GLP-1; active and total) measurement will be collected on Days -1, 1 and 16 at -0.5 predose and 0.5, 1, 1.5 and 2.0 hours following the morning dose.

Collection times for blood samples in later cohorts may be modified as preliminary PD data become available during the study from initial cohorts, but the total blood volume collected from each subject for all analyses (PK, PD, safety) will not exceed 550 mL over 8 weeks in this study.

All urine will be collected, within specified intervals: on Day –1 at T0–T2, T2–T4.5 (prior to lunch), T4.5–T7, T7-10.5 (prior to dinner), T10.5-13, and T13-24 hours, where T0 corresponds to the dosing time on dosing days, on Day 1 at 0-2, 2-4.5 (prior to lunch), 4.5-7, 7-10.5 (prior to dinner), 10.5-13, 13-24 and 24-48 hours postdose; on Day 16 at 0-2, 2-4.5 (prior to lunch), 4.5-7, 7-10.5 (prior to dinner), 10.5-13, 13-24, 24-48, 48-72 and 72-96 hours after the last dose; and on Days 3 to 15 at 0-24 hours.

PHARMACOGENOMICS:

A pharmacogenomic blood sample (10 mL) will be collected from subjects who give separate written informed consent for this component of the study. This will allow for pharmacogenomic research, as necessary. Participation in this pharmacogenomic research is optional.

SAFETY EVALUATIONS*:

Physical examinations including thorough whole body skin examination will be performed at Screening and on Days –2, 15, 20 and at the follow up visit.

Vital signs (resting blood pressure and heart rate) and 12-lead ECGs will be performed at Screening, and at approximately T0, T2, T6 and T12 hours on Day –1 where T corresponds the dosing time on dosing days. The vital signs and 12-lead ECGs will be performed at 0 (predose), 2, 6, 12, 24, 48, 72 and 96 hours post dose on Days 1 and 16, and at 2 hours postdose on Days 3 to 15 and once at the follow-up visit. At each specified times except at Screening and the follow-up visit, the vital signs and 12-lead ECG measurementwill be performed in triplicate, approximately 2 minutes apart. . The average of the triplicate measurements at each time point on Day –1 will serve each subject's time-matched baseline value for comparison to the corresponding parameters measured on Days 1 to 20. All morning vital signs and 12-lead ECGs will be performed prior to dosing on study drug administration days and prior to breakfast on non-dosing days.

* This section of the protocol has been revised. Please refer to the section of this document titled PROTOCOL AMENDMENTS (Amendment DEU-5, 27 September 2007) for a detailed description of the specific changes.

SYNOPSIS (CONTINUED)

Continuous Lead II ECG monitoring (telemetry) will be conducted from 30 minutes prior to dosing until 8 hours post dosing on Days -1, 1, 6, 8 and 15. These telemetry ECG data will not be recorded on the database and are for visual monitoring. Any abnormality detected by the device or the investigator will be printed out and retained as source data. Any clinically significant abnormalities will be recorded as adverse events.

Orthostatic vital signs will be measured on Day –2 and Day 1 to 20 at 4 and 8 hours post dose.

Body temperature will be measured at Screening, on the morning of Day –2 and Day 1 to 20 and at the follow-up visit.

Height and weight will be measured at the Screening visit and will be utilized to calculate BMI and BSA. Weight will be measured at Screening, on Days –1 to 20 and at the follow-up visit on a calibrated scale. Weekly calibration records will be stored in study files.

Adverse events will be recorded during the entire study and will be specifically queried on each dosing day at predose, 3, 6 and 12 hours post dose, once on Days 2, 20, 21, 22 and at the follow up visit.

Fasting samples for hematology, clinical chemistry and urinalysis dipstick will be collected at Screening, on Days -2, 2, 3, 5, 8, 11, 14, 17, 18, 20, and 22 and at the follow up visit. Samples for measurement of TSH to assess thyroid function will be collected at Screening, and for coagulation including International normalized ratio (INR) and activated partial thromboplastin time (APTT) will be collected at Screening and Day 20.

For each urine collection interval, total urine volume will be recorded, and, samples will be taken for the following analyses:

-glucose

-creatinine

-electrolytes: sodium, chloride, potassium, phosphate, calcium and magnesium

-pH, specific gravity and osmolality

-albumin

-N-acetyl-beta-glucosaminidase(NAG), and beta-2-microglobulin

All fluid intake, including water and all other beverages consumed at dosing and during meals, urine volume and frequency of urination over each 24-hour period will be recorded.

Urine levels of collagen cross-linked N-telopeptides (NTX) and urine deoxypyridinolines (DPD), serum bone specific alkaline phosphatase and serum osteocalcin (OC) will be assessed on Days –2, -1, 2, 3, 5, 8, 11, 14, 17, 18, 20, and 22 and at the follow up visit. Serum 1,25 dihydroxy Vitamin D and parathyroid hormone (PTH) will be assessed at screening and on Days -2, 5, 11, 17, 22 and at the Follow-up visit.

The investigational staff will monitor the subjects for symptoms of hypoglycemia throughout the in-clinic residency. Should symptoms possibly indicative of hypoglycemia appear (e.g. altered mental status, dizziness/lightheadedness, diaphoresis, tachycardia), blood glucose will be immediately measured using a glucose analyzer and confirmed by a conventional venous blood sample glucose determination. Subjects will also measure their fasting (morning) and postprandial (post lunch) blood glucose levels daily throughout the study (during the prestudy washout, inpatient and follow-up phases).

Subjects will be domiciled continuously from Days -3 through Day 20 and instructed to avoid direct exposure to sunlight or artificial sources of intense light for at least 96 hours post the last dose to minimize the risk of potential photosensitivity. In addition, subjects will be closely monitored for potential skin reactions with a thorough examination of the whole body skin by a physician or his/her designee at Screening, on Days -1 through Day 20 at each dose level.

PATIENT REPORTED OUTCOMES EVALUATION:

A short questionnaire related to urination frequency will be administered at approximately the same time each morning on Days -2, 4, 7 and 14 to each subject (Attachment 12).

SYNOPSIS (CONTINUED)

STATISTICAL METHODS:

PK: Pharmacokinetic analyses will be performed for all subjects receiving at least 1 dose of JNJ-28431754. Results will be summarized and descriptive statistics will be generated for each dose level.

PD: Mixed effect ANOVA modeling will be used to assess the treatment effects on 24-hour mean plasma glucose and urine glucose excretion (amount and rate). The estimated least-squares means and appropriate 95% confidence intervals for the difference of the mean pharmacodynamic parameters will be obtained for pharmacodynamic evaluations.

Safety: All data will be fully listed. Unless otherwise noted, all the measures will be summarized with descriptive statistics for each dose level and all placebo observations grouped together. All statistical analyses will be considered exploratory and interpreted as such. No corrections will be made for multiple comparisons.

It is estimated that a sample size of 20 (16 receiving active JNJ-28431754 and 4 receiving placebo) subjects with T2DM should be sufficient to detect a 15% reduction in 24-hour mean AUC with 80% power, assuming a one-sided test and a coefficient of variation of 18 percent.

TIME AND EVENTS SCHEDULE*

	Screen[a]	D-17[b]	D-2	D-1	Days 1 & 16																														D2	D3	D4	D5	D6-7	D8	D9-10	D11	D12-13	D14	D15	D17	D18	D19	D20	D21	D22	Follow-up[c]
Time					-0.5	-0.25	0	0.25	0.5	1	1.25	1.5	2	2.5	3	4	4.5	5	5.5	6	6.5	7.0	8	9	10	10.5	11	12	12.5	13	14	16	19	22																		
Resident in clinic[d]			x---																																														---x			
Informed consents	x																																																			
Diet instruction		x																																																		
Medical history	x																																																			
Physical exam	x		x																																										x				x			x
Height	x																																																			
Weight[e]	x			x	x																														x	x	x	x	x	x	x	x	x	x	x	x	x	x	x			x
Temperature	x		x		x																														x	x	x	x	x	x	x	x	x	x	x	x	x	x	x			x
Serology[f]	x																																																			
Pregnancy[g]	x	x	x																																																	
Drug-alcohol screen[h]	x		x																																																	
Vital signs[i]	x			x			x						x							x								x							x	x	x	x	x	x	x	x	x	x	x	x	x	x	x			x
Orthostatic VS[j]			x													x							x												x	x	x	x	x	x	x	x	x	x	x	x	x	x	x			

* This section of the protocol has been revised. Please refer to the section of this document titled PROTOCOL AMENDMENTS (Amendment DEU-5, 27 September 2007) for a detailed description of the specific changes.

TIME AND EVENTS SCHEDULE (CONTINUED)

	Screen[a]	D-17 washout	D-2	D-1	Days 1 & 16																														D2	D3	D4	D5	D6-7	D8	D9-10	D11	D12-13	D14	D15	D17	D18	D19	D20	D21	D22	Follow-up
Time					-0.5	-0.25	0	0.25	0.5	1	1.25	1.5	2	2.5	3	4	4.5	5	5.5	6	6.5	7.0	8	9	10	10.5	11	12	12.5	13	14	16	19	22																		
12-Lead ECG[k]	x			x			x						x							x								x							x	x	x	x	x	x	x	x	x	x	x	x	x	x	x			x
Oral dose[l]			x	x			x																													x	x	x	x	x	x	x	x	x	x							
PK sample collection[m]						x			x	x		x	x	x	x	x				x			x		x			x							x	x		x	x		x	x	x		x	x	x	x	x	x	x	
Urine collection[n]				x	x	x	x	x	x	x	x	x	x	x	x	x	x	x	x	x	x	x	x	x	x	x	x	x	x	x	x	x	x	x	x	x	x	x	x	x	x	x	x	x	x	x	x	x	x		x	
PD sample collection[o]				x	x	x	x		x	x	x	x	x	x	x		x	x	x	x	x	x	x	x		x	x	x	x	x	x	x	x	x	x	x	x	x	x	x	x	x	x	x	x	x	x	x	x	x	x	
Safety labs[p]	x		x	x																															x	x		x		x		x		x		x	x		x		x	x
PGx sample[q]							x																																													
Visual analogue scale[r]			x																																	x			x			x			x							
Fluid input-output[s]			x	x	x																														x	x	x	x	x	x	x	x	x	x	x	x	x	x	x			
Skin assessment[t]	x			x	x																														x	x	x	x	x	x	x	x	x	x	x	x	x	x	x			x
Telemetry[u]				x	x	x	x	x	x	x	x	x	x	x	x	x	x	x	x	x	x	x	x																x	x					x							
Hypoglycemia assessments[v]																																																				
AE/conmed recording[w]																																																				
PRO questionnaire			x																																		x		x					x								

a Within 35 days prior to Day -3
b Existing anti-diabetic medication use discontinued on Day -16; subjects begin weight maintaining diet as instructed by the dietician and receive instruction in signs and symptoms of hypoglycemia, use of glucose analyzer and reporting of results on Day-17
c 7-10 days following the Day 22 visit
d Subjects will be admitted to the clinic on Day -3 but, no assessments will be performed
e Determine weight, BSA, and BMI at Screening. Fasting body weight on Days -1 through 20 and at the follow-up visit
f HBsAg, HIV and HCV
g (1) Post-menopausal is defined as no menses for at least 18 months prior to study start or no menses for 6 to 18 months prior to the start of this study, and plasma FSH must be ≥ 40 mIU/mL and estradiol ≤ 20 pg/mL in 3 separate measurements which will occur at Screening, Day -17, and Day -2 (a -2 day window is allowed for Day -2); (2) Pre-menopausal surgically sterilized subjects must have a negative β-HCG pregnancy test at Screening and at Day –2
h Urine drug screening, alcohol breath test
i Vital signs (blood pressure and heart rate) will be performed at Screening, and at T0, T2, T6 and T12 on Day –1 where T corresponds to the dosing time on dosing days. The vital signs will also be performed at 0 (predose), 2, 6, 12, 24, 48, 72 and 96 hours post dose on Days 1 and 16, and at 2, 6 and 12 hours on Days 3 to 15 and once at the follow-up visit.
j Orthostatic vital signs will be measured on Day –2 and Days 1 to 20 at 4 and 8 hours post dose or corresponding to dosing time.
k 12-lead ECGs will be performed at Screening, and at T0, T2, T6 and T12 on Day –1 where T corresponds the dosing time on dosing days. The 12-lead ECGs will also be performed at 0 (predose), 2, 6, 12, 24, 48, 72 and 96 hours post dose on Days 1 and 16, and at 2 hours on Days 3 to 15 and once at the follow-up visit. The 12-lead ECGs will be measured in triplicate 2 minutes apart at each predefined point. The average of the triplicate measurements at each time point on Day –1 will serve each subject's time-matched baseline value for the corresponding parameters on Days 1 to 20. All morning vital signs and 12-lead ECGs will be performed prior to dosing on study drug administration days and prior to breakfast on non-dosing days.
l Subjects are randomized on Day 1, then receive study medication after at least a 10 hour fast; on Day 1; study medication is resumed on Days 3-16; all subjects receive a placebo dose on Days -2 and -1; meal times must be recorded on the CRF
m Day 1: Plasma samples for measurement of JNJ-28431754 concentration will be collected at predose, 0.5, 1, 1.5, 2, 2.5, 3, 4, 6, 8, 10, 12, 24 and 48 hours postdose (total 14 samples). Days 3 to 15: Blood samples will be collected at pre-dose in the morning on Days 3, 5, 7, 9, 11, 13 and 15 to obtain trough JNJ-28431754 concentrations (total 7 samples) Day 16: Plasma samples for measurement of JNJ-28431754 concentration will be collected at predose, 0.5, 1, 1.5, 2, 2.5, 3, 4, 6, 8, 10, 12, 24, 48, 72, 96, 120 and 144 hours postdose (total 18 samples).
n PK Day 1: Urine samples for analysis of JNJ-28431754 will be collected at predose, 0-2, 2-4.5, 4.5-7, 7-10.5, 10.5-13, 13-24 and 24-48 hours post dose. Day 16: Urine samples for analysis of JNJ-28431754 will be collected at 0-2, 2-4.5, 4.5-7, 7-10.5, 10.5-13, 13-24, 24-48, 48-72 and 72-96 hours post dose.
PD:All urine will be collected, within specified intervals: on Day –1 at T0–T2, T2–T4.5, T4.5–T7, T7-10.5, T10.5-13, and T13-24, where T0 corresponds to the dosing time on dosing days. All urine will also be collected within specified intervals: 0-2, 2-4.5, 4.5-7, 7-10.5, 10.5-13, 13-24 and 24-48 hours postdose on Days 1 and 16, 48-72 and 72-96 on Day 16 and 0-24 hours on Days 3 to 15.
o Blood samples for plasma glucose, insulin and C-peptide will be collected on Day –1 at T-0.5, T-0.25, T0, T0.5, T1, T1.25, T1.5, T2.0, T2.5, T3.0, T4.5 (prior to lunch), T5.0, T5.5, T6.0, T6.5, T7.0, T8.0, T9.0, T10.5 (prior to dinner), T11, T12.0, T12.5, T13.0, T14.0 T16.0, T19.0, T22.0, and T24 hours where T0 corresponds to the dosing time on dosing days, and on Day 1 and 16 at -0.5, -0.25, 0, 0.5, 1, 1.25, 1.5, 2.0, 2.5, 3.0, 4.5 (prior to lunch), 5.0, 5.5, 6.0, 6.5, 7.0, 8.0, 9.0, 10.5 (prior to dinner), 11, 12.0, 12.5, 13.0, 14.0 16.0, 19.0, 22.0, and 24 hours postdose. Morning fasting plasma glucose, insulin and C-peptide concentrations will be obtained on Days 3 to 22. Blood samples for GLP-1 active and total will be collected on Days -1, 1 and 16 at -0.5 predose and 0.5, 1, 1.5 and 2.0 postdose
p Fasting samples for hematology, clinical chemistry and urinalysis dipstick will be collected at Screening, on Days -2, 2, 3, 5, 8, 11, 14, 17, 18, 20, and 22 and at the follow up visit. A fasting sample for serum creatinine clearance on Day -1 will be obtained. A sample for TSH is collected at screening. Samples for INR and aPTT will be collected at Screening and Day 20. Urine NTX and urine deoxypyridinolines (DPD), bone specific alkaline phosphatase and serum osteocalcin (OC) will be assessed on Days –2, -1, 2, 3, 5, 8, 11, 14, 17, 18, 20, and 22 and at the follow up visit. Serum 1,25 dihydroxy Vitamin D and parathyroid hormone (PTH) will be assessed at screening and on Days -2, 5, 11, 17, 22 and at the Follow-up visit.
q A 10 mL blood sample (Day 1 only) will be collected only from subjects who give informed consent for the pharmacogenomic part of the study
r Administration of visual analogue scale (VAS) questionnaires to assess appetite and satiety at 5 minutes prior to breakfast, 30, 60, 90, 120, 150, 180, 210 and 240 minutes after breakfast, 5 minutes prior to lunch and immediately after lunch on Days –2, 3, 7, 11 and Day 15.
s All fluid intake (water and all other beverages), urine volume and frequency of urination over each 24-hour period will be recorded in the eCRFs
t subjects will be closely monitored for potential skin reactions with a thorough examination of the whole body skin for skin reactions (e.g. erythema, rash) by a physician or his/her designee at Screening, on Days -1 through Day 20 at each dose level.
u Continuous Lead II ECG monitoring (telemetry) will be conducted from 30 minutes prior to dosing until 8 hours post dosing on Day -1, 1, 6, 8 and 15.
v Investigational staff will monitor subjects for symptoms possibly indicative of hypoglycemia throughout the in-residence period. Blood glucose will be immediately measured using a glucose analyzer and confirmed by a conventional venous blood sample, should symptoms possibly indicative of hypoglycemia appear.
w Adverse events will be recorded during the entire study and will be specifically queried on each dosing day at predose, 3, 6 and 12 hours post dose, once on Days 2, 20, 21, 22 and at the follow up visit.

ABBREVIATIONS

ADME	absorption, distribution, metabolism, excretion
Ae	amount excreted into the urine
Ae (% dose)	% of the administered dose excreted in urine as unchanged drug
AE(s)	adverse event(s)
ALT	alanine aminotransferase
ANOVA	analysis of variance
anti - HCV	serum antibody against Hepatitis C virus
AST	aspartate aminotransferase
AUC	area under the curve
AUC_{∞}	area under the curve from zero to infinite time
AUC_{t1-t2}	area under the curve from time 1 to time 2
BID	twice daily
BMI	Body Mass Index
BSA	Body Surface Area
BUN	blood urea nitrogen
bw	body weight
°C	degree Celsius
CI	confidence interval
CL/F	total clearance of drug after extravascular administration, corrected for absolute bioavailability
CL_R	renal clearance
C_{max}	maximum observed plasma concentration
CPK	creatine phosphokinase
CR	creatinine
CRF	case report form
CRU	Clinical Research Unit
CYP	Cytochrome P450 oxidase
DNA	deoxyribonucleic acid
DRC	Data Review Committee
DRF	dose range-finding
ECG	electrocardiogram
eCRF	electronic case report form
EDC	electronic data capture
FDA	Food Drug Administration
FIH	first in human
FOBT	fecal occult blood test
FPG	fasting plasma glucose
FSH	follicle stimulating hormone
GCP	Good Clinical Practice
GGT	gamma-glutamyl transpeptidase
GLP	Good Laboratory Practice
GLP-1	glucagon like peptide-1
GLUT1	glucose transporter 1
GMP	Good Manufacturing Practice

ABBREVIATIONS (CONTINUED)

h	hour
HbA1c	hemoglobin A1c
HBsAg	hepatitis B surface antigen
hCG	human chorionic gonadotrophin
HED	human equivalent dose
HIV	Human Immunodeficiency Virus
HPLC	high-performance liquid chromatography
i.p.	intraperitoneal
i.v.	intravenous
IC50	inhibiting concentration at 50%
ICH	International Conference on Harmonization
IEC	Independent Ethics Committee
IRB	Institutional Review Board
J&JPRD	Johnson & Johnson Pharmaceutical Research & Development
kg	kilogram
LC-MS	liquid chromatography coupled to mass spectrometry detection
LC-MS/MS	liquid chromatography coupled to tandem mass spectrometry detection
LOAEL	lowest observed adverse effect level
λ_ζ	elimination rate constant
MedDRA	Medical Dictionary for Regulatory Activities
μg	microgram
mg	milligram
mL	milliliter
mRNA	messenger ribonucleic acid
MRSD	maximum recommended starting dose
MS	mass spectrometry
n	number (size of a sub-sample)
N	number (total sample size)
NAG	N-acetyl-ß-D-glucosaminidase
ng	nanogram
NOAEL	No Observed Adverse Effect Level
OC	osteocalcin
OGTT	oral glucose tolerance test
PD	pharmacodynamic
PG	pharmacogenomic
PI	Principal Investigator
PK	pharmacokinetic
PTH	parathyroid hormone
QTc	heart-rate corrected QT time interval in ECG
QTcB	corrected QT interval based on Bazett’s method
QTcF	corrected QT interval based on Fridericia’s method
RBC	red blood cell
SAD	single ascending dose

ABBREVIATIONS (CONTINUED)

SGLT	sodium glucose co-transporter
SOP(s)	standard operating procedure(s)
$t_{½}$	terminal half life
T2DM	Type 2 Diabetes Mellitus
t_{max}	time to attain maximum observed plasma concentration
USP	United States Pharmacopeia
UV	ultra violet
UVA	ultra violet A light
VAS	visual analog scale
Vd/F	apparent volume of distribution following oral administration
vs.	versus
WBC	white blood cell
WHO	World Health Organization
ZDF	Zucker diabetic fatty

1. INTRODUCTION

JNJ-28431754 is a potent, selective and reversible inhibitor of the renal sodium glucose co-transporter (SGLT2). It is being investigated for the treatment of Type 2 diabetes mellitus (T2DM).

T2DM is a metabolic disorder characterized by 2 primary defects: decreased insulin secretion by the pancreas, and resistance to insulin action in peripheral tissues (muscle, liver and adipose), which results in impaired glucose uptake and hyperglycemia. Chronic hyperglycemia leads to progressive impairment of insulin secretion and further insulin resistance of peripheral tissues (so-called glucose toxicity[1, 2]), which further worsens control of blood glucose. In addition, chronic hyperglycemia is a major risk factor for diabetic complications, including heart disease[3], retinopathy[4], nephropathy[5] and neuropathy[6]. Aggressive glycemic control decreases microvascular[7] and perhaps macrovascular complications.[8]

Although existing antidiabetic therapies can be effective, it can still be difficult to maintain optimal glycemic control in many diabetics. High rates of morbidity and mortality persist in the diabetic population. Thus, new antidiabetic agents that provide improved efficacy, safety and tolerability and that can be used in combination with existing therapies will be valuable additions for the treatment of Type 2 diabetes.

There are at least four isoforms of the SGLTs: SGLT1, SGLT2, SGLT3, and SGLT4. In healthy humans, the renal glomerula filter approximately 180 g of glucose from plasma each day. Most all of the filtered glucose is reabsorbed in the convoluted proximal tubule by SGLT2, a low-affinity, high-capacity co-transporter with a Na^+-glucose transport ratio of 1:1. Only a small proportion of the filtered glucose is absorbed in the straight proximal tubule by SGLT1. Both SGLT1 and SGLT2 are located in the apical plasma membranes of the tubular epithelium and derive their energy from the inward sodium gradient created by the Na^+/K^+ ATPase pumps located on the basolateral membrane. Once reabsorbed across the apical membrane, the elevated cytosolic glucose is then transported across the basolateral membrane, into the interstitial space by facilitated glucose transporters (GLUT1 and GLUT2).[9, 10]

In normal mice, rats and dogs and several rodent models of diabetes, JNJ-28431754 promotes urinary glucose excretion. Treatment with JNJ-

28431754 in rodent models of diabetes led to improved glucose tolerance and reduced fasting and postprandial serum glucose concentrations and HbA1c levels. In the two-week rat and dog toxicology studies, JNJ-28431754 increased urinary glucose secretion, and at higher exposures, decreased serum glucose concentrations. However, there were no significant changes in electrolyte balance and renal function[11]

This will be the first study of JNJ–28431754 in diabetic subjects. This study will evaluate the safety, tolerability, pharmacokinetics and pharmacodynamics of multiple oral ascending doses of JNJ-28431754 in male and postmenopausal or surgically sterilized female T2DM subjects. Pre-clinical and also clinical data (from the first single oral dose clinical study of JNJ-28431754) that support this multiple ascending dose study are summarized in Section 1.1. of this protocol. More detailed information regarding JNJ–28431754 can be found in the JNJ–28431754 Investigator's Brochure.[11]

The term Sponsor used throughout this document is referred to the entities listed in the Contact Information page(s), provided as a separate document.

1.1. Background

1.1.1. Pre-clinical Data

Pharmacology[11]

JNJ-28431754 is a potent, selective SGLT2 inhibitor in cultured Chinese Hamster Ovary CHOK1 cells expressing either human SGLT1 or SGLT2. In these cells, JNJ-28431754 inhibits Na^+-dependent C-α-methylglucoside uptake with an IC_{50} of 4.1 nM (1.8 ng/mL) and 664 nM (295 ng/mL) against SGLT2 versus SGLT1, respectively. A similar IC_{50} of 2.0 nM (0.9 ng/mL) was obtained for murine SGLT2. JNJ-28431754 had no significant inhibitory activity against the rat skeletal muscle GLUT1 (IC_{50} > 10000 nM).

Single oral doses of JNJ-28431754 administered prior to an oral glucose challenge produced a dose-dependent increase in urinary glucose excretion and urine volume in normal mice, diabetic rats and non-diabetic obese dogs. These pharmacological activities were related to drug exposure (AUC_{0-24h}) of JNJ-28431754 in plasma. Chronic treatment for 4 weeks with JNJ-28431754 resulted in a dose-dependent lowering of fed and fasted blood glucose levels as well as HbA1c in diabetic mice and rats. Moreover, chronic

treatment with JNJ-28431754 improved beta-cell function as reflected in a dose-dependent increase in plasma insulin levels in diabetic mice.

In addition, following chronic administration with JNJ-28431754 in ob/ob or diet-induced obese mice for 4 weeks, or in Sprague-Dawley rats for 2 weeks, JNJ-28431754 caused dose dependent reduction in body weight gain in the ob/ob mice and Sprague Dawley rats witthout significant increase in food intake, and caused body weight loss in diet-induced obese mice.

Safety Pharmacology and Toxicology[11]

Receptor Binding/ Selectivity

JNJ-28431754 was tested for in vitro inhibition of binding of relevant ligands to 50 recombinant human receptors. At 10 μM (free drug concentration of about 4.4 μg/mL; total drug concentration of about 220 μg/mL), JNJ-28431754 inhibited ligand binding to the NE transporter by 34% and inhibited ligand binding to 5-HT2A receptors by 37%. Binding to all other receptors was inhibited by less than 20%.

Safety Pharmacology

Nonclinical cardiovascular and pulmonary safety pharmacology studies suggested that JNJ-28431754 has a low potential for inducing adverse cardiovascular or pulmonary effects. JNJ-28431754 did not reduce IKr current in hERG-transfected HEK293 cells at the highest tested plasma concentration of 3 μM (free drug concentration of about 1.3 μg/mL; total drug concentration of about 65 μg/mL). In Langendorff-perfused rabbit hearts, JNJ-28431754 had no notable effect on electrophysiological parameters at the highest tested plasma concentration of 1 μM (free drug concentration of about 0.4 μg/mL; total concentration of about 20 μg/mL). In anesthetized guinea pigs, JNJ-28431754 had no drug-related cardiovascular effects detected at cumulative intravenous doses up to 9.86 mg/kg (a plasma drug concentration of approximately 13 μg/mL). In conscious dogs, oral doses up to 400 mg/kg (mean plasma concentration at 24 h post-dose of approximately 55 μg/mL) of JNJ-28431754 were not associated with notable cardiovascular and pulmonary effects. JNJ-28431754 did not elicit notable neurobehavioral effects in male rats at single doses up to 1000 mg/kg.

Toxicology

In single dose studies in mice and rats over an oral dose range of 250 to 2000 mg/kg, or over an i.p. dose range of 62.5 to 500 mg/kg, JNJ-28431754 was well tolerated in mice. The maximum non-lethal doses of JNJ-28431754 in mice were 2000 mg/kg and 500 mg/kg after a single oral and i.p dose, respectively. One of five female rats died after oral dosing (2,000 mg/kg), and 1/5 and 3/5 male rats died after receiving i.p. doses of 250 and 500 mg/kg, respectively. General gastrointestinal findings of abnormal feces were noted in both mice and rats after both routes of administration. The maximum non-lethal doses of JNJ-28431754 in rats were 1000 mg/kg and 125 mg/kg after a single oral and i.p dose, respectively.

In a 5-day dose finding (dose range: 10, 50, 250 and 500 mg/kg; N=5/sex/group) study in rats, one female rat at 500 mg/kg dose was terminated in moribund condition on Day 4; the cause of moribund condition for this animal was undetermined. There was a slight increase in stress associated peracute gastric erosions seen histologically in rats dosed at ≥ 50 mg/kg/day.

In 14-day GLP toxicology studies in rats (dose range: 3, 20 and 150 mg/kg; N=10/sex/group) slight increases in serum BUN, ALT, and AST were noted in rats dosed at ≥ 20 mg/kg/day. These changes were only slightly higher than controls, and had no histopathologic correlate; they are not considered toxicologically relevant. Microscopically, minimal to mild, acute, superficial gastric erosions in the glandular portion of the stomach were noted in all dose groups including controls: 1/10 female control rat, 2/10 female rats at 3 mg/kg and in both male and female rats at 20 mg/kg (3/10 and 2/10) and 150 mg/kg (4/10 and 4/10), respectively. Although this appears to be indicative of a dose-response, subsequent investigative toxicology studies have demonstrated that these are peracute responses, attributed to the stress of the combination of overnight fast and the bleeding procedure on the morning of necropsy. The increased incidence with dose in this study is thought to be related to the added effect of the additional stress associated with the compound such as the low blood glucose levels observed at the high dose in the study. These acute changes to the gastric mucosa are not considered primary, direct effects of the compound. The no observed adverse effect levels (NOAEL) for this study were determined to be 20 mg/kg for males and females based on the incidence of hyperostosis

(minimal to mild changes in distal end of femur & proximal end tibia) at 150 mg/kg. All other changes noted in the study were not considered to be toxicologically significant. The C_{max} and AUC_{0-24} values at the NOAEL were 4,690 ng/mL and 68,644 ng.h/mL in males and 5,795 ng/mL and 80,355 ng.h/mL in females, respectively. The lowest observed adverse effect level (LOAEL) associated with the observed hyperostosis were 32,850 ng/mL and 511,759 ng.h/mL in males and 45,775 ng/mL and 748463 ng.h/mL in females, respectively.

In a 5-day dog toxicology study (dose range:25, 100, 400 and 800 mg/kg; one dog of each gender at each dose level), a female dog at 800 mg/kg dose was euthanized on Day 5. Key toxicity findings from the dead animal include marked hypoglycemia (0.26x of control serum glucose), dehydration, high urine ketone, and lung findings suggestive of possible aspiration of emesis. No significant toxic effect was seen in dogs treated for 2 weeks with oral doses up to the NOAEL of 400 mg/kg (C_{max}: 40,200/76,800 ng/mL, M/F; AUC_{0-24h}: 539,359/1,067,058 ng.h/mL, M/F).

In the 2-week GLP toxicity study in dogs (dose range:4, 40 and 400 mg/kg, N=3/sex/group), minimal signs of toxicity were noted. The NOAEL for this study was 400 mg/kg/day. The corresponding C_{max} and AUC values at the NOAEL were 40,200 ng/mL and 539,359 ng.h/mL in males, and 76,800 ng/mL and 1,067,058 ng,h/mL in females.

In repeat dose studies up to 2 weeks in both rats (dose range 10-500 mg/kg/day) and dogs (dose range 4-800 mg/kg/day), pharmacological effects of JNJ-28431754 were evident in the clinical chemistry and urinalysis data. Elevated urinary glucose excretion along with increased urine volume and minimal to moderate decreases in serum glucose levels were observed. There were no meaningful changes in serum and urine electrolytes except that urinary excretion of calcium was increased up to 10 fold in rats and 4 fold in dogs.

In most dogs receiving 200 mg/kg/day in an ongoing 3 month GLP toxicology study, excessive diarrhea/loose stools and emesis were observed during the second week of the study. In this same group (n = 10; 5 dogs/sex), evidence of blood in stools was observed in 3/5 males. In general the animals of this group were depressed, inactive and some not eating and some appeared dehydrated. Upon lowering the dose to 100 mg/kg/day, the dogs

regained their appetites and, no longer show blood in stools, but the other fecal changes and emesis continue to occur. Occasionally mucoid feces and emesis have been observed in mid dose dogs (30 mg/kg/day, n = 6; 3/sex). These signs are probably related to the watery stools that were observed in an earlier study in dogs treated orally for 5 days with JNJ-2841754 at doses greater than or equal to 100 mg/kg/day.

Phototoxicity

In vitro cytotoxicity and phototoxicity of JNJ-28431754 were assessed by neutral red uptake assays in cultures of normal Balb/c 3T3 mouse fibroblasts following exposure to ultraviolet A light (UVA). Results indicated that JNJ-28431754 was phototoxic in vitro. A confirmatory in-vivo phototoxicity study was subsequently conducted in Sprague-Dawley rats following a single oral dose administration of JNJ-28431754 at 5, 50 and 500 mg/kg dose levels. Results from this study indicated that JNJ-28431754 was not phototoxic at the lowest dose, but was phototoxic to the skin (mild erythema) of female rat (in 1 of 5 female rats) at the mid dose, and to the skin of both males (mild erythema and edema in 1 of 5 male rats) and females (mild erythema and mild to moderate edema in 3 of 5 female rats) at the highest dose. These skin reactions occurred one day after the study drug administration and a UVA and UVB radiation (UVR) exposure. JNJ-28431754 was not associated with eye phototoxicity at any dose level tested in this study based on clinical, ophthamological and histopathological assessments.

Carcinogenicity, Mutagenicity

Genotoxicity studies completed thus far on JNJ 28431754 include the Ames test (negative in 5 strains tested: Salmonella strains: TA98, TA100, TA1535, TA1537; E. Coli strain: WP2uvrA), the in vitro mouse lymphoma assay (negative in the absence of S9 microsomes but positive in the presence of S9 microsome fraction from induced rat livers), the in vivo, single dose rat micronucleus test (negative) and the in vivo, single dose COMET study in rats (negative in rat liver). Mean plasma concentrations of JNJ-28431754 measured at 2 and 6 hours post dose were in the range of 125-136 mg/mL, which is approximately 25 fold higher than the Cmax observed at the maximum dose of 800 mg (Cmax 5.2 mg/mL) administered in the first in human study. From the panel of studies conducted, the in vivo COMET

study is currently considered a good indicator of in vivo genotoxic potential and is the most appropriate test to determine the relevance of the positive in vitro findings in the mouse lymphoma test. The negative outcome of the COMET test proves that JNJ-28431754 even after metabolic activation in vivo is not DNA-reactive to rat hepatocytes. Based on the negative findings in the COMET assay, and all other negative results in the in vitro and in vivo tests, the overall genotoxic potential of this compound is considered minimal towards humans.

Other toxicity studies

In an invitro bovine corneal opacity-permeability (BCOP) assay, JNJ-28431754 as a 20 % (w/w) suspension induced no increase in corneal opacity and a small increase in permeability. From this assay, a score of 3.2 ± 0.4 was calculated, which classified the RWJ-416457 formulation as a borderline non to mild eye irritant.

In a study to evaluate the skin sensitization potential of JNJJ-28431754 in mice at concentrations of 2.5, 15, 30 and 100 g, JNJ-28431754 did not cause redness or swelling of the ear of the mice, and radioactivity measurements in mice treated with JNJ 28431754 were not different from control animals. Thus, the stimulation index (SI) was less than 3.0 indicating the test article is not a contact sensitizer under the conditions of the study.

Based on available data from JNJ-28431754 safety and toxicity studies, potential dose-limiting human intolerability or toxicities may include, but are not limited to, osmotic diuresis due to increased urinary glucose excretion, changes in serum or urine electrolytes, GI intolerability, hypoglycemia, changes in bone formation and or in bone resorption, abnormalities in hepatic and renal functions, and phototoxicity.

Pharmacokinetic Profile[11]

Following single oral dose administration of JNJ-28431754 in mice, rats, dogs and monkeys, mean peak concentrations (t_{max}) were reached at 0.5 to 5.5 hours post-dose. The mean $t_{1/2}$ ranged from 3 to 8 hours with absolute oral bioavailability ranging from approximately 34% to 137%.

In the 2-week rat toxicology studies, mean C_{max} and $AUC_{0\text{-}24h}$ values increased more than proportionately with increased doses after single dose administration. For doses in a ratio of 1:7:50, the C_{max} and $AUC_{0\text{-}inf}$ values increased in a ratio of about 1:8:80 and 1:10:135, respectively. However,

following multiple dose administrations, the C_{max} and AUC_{0-24h} values increased dose proportionately. Mean t_{max} and $t_{1/2}$ values ranged approximately from 2 to 9 hours and 7 to 11 hours, respectively. At the high dose (150 mg/kg), C_{max} and AUC values decreased about 2 fold after 2-weeks of dosing compared to the first dose.

Following 2-week dosing of JNJ-28431754 in dogs, increases in C_{max} and AUC_{0-24h} values were less than dose proportional, likely due to emesis or absorption saturation. The drug exposure (AUC_{0-24h}) was up to 2.5 fold higher on Day 14 after multiple dosing, than the initial single dosing on Day 1, suggesting possible drug accumulation at steady state. Mean t_{max} and $t_{1/2}$ values ranged from 1 to 3 hours and 9 to 14 hours, respectively. C_{max} and AUC values were generally higher in females than in males in both rats and dogs. The difference in Cmax and AUC values rangedabout 10 to 90%. JNJ-28431754 has a plasma protein binding of 98% in all species tested, and the protein binding was concentration independent. The predominant plasma protein responsible for binding was albumin.

In vitro, unchanged JNJ-28431754 accounted for approximately 50% of drug-derived components in hepatocytes of rat, dog, and human. The major metabolic pathway of JNJ-28431754 in both rat and dog species was oxidation to a carboxy metabolite, while the major metabolite in human hepatocytes was from glucuronidation of the hydroxyl functionality on 2-hydroxymethyl-tetrahydro-pyran-3, 4, 5-triol moiety (detected for a total of 42%). All the metabolites identified in the human hepatocytes were also found in rat and/or dog hepatocytes. JNJ 28431754 was moderately metabolized in rats and dogs in vivo. The metabolic profile was qualitatively similar but quantitatively different between these two species. In rats and dogs, unchanged drug was the major drug-related material in systemic circulation. Mono oxygenation (aromatic hydroxylation) and alcohol oxidation were the predominant biotransformation pathways in the rat, while O-glucuronidation and mono-oxygenation (aromatic hydroxylation) pathways appeared to be more pronounced in the dog

In human hepatocyte cultures, JNJ-28431754 did not cause induction of mRNA or enzyme activity of CYPs 3A4, 2C9, 2C19 and 1A2 at concentrations up to 10 μM (4.4 μg/mL). The ability of JNJ-28431754 to inhibit major human cytochrome P450 (CYP) enzymes was evaluated in

vitro using human hepatic microsomes pooled from adult males and females. JNJ-28431754 showed a weak inhibition of CYP3A4 (testosterone) and CYP2C9 (tolbutamide) with IC50 values of 27 μM (12 μg/mL) and 80 μM (35.6 μg/mL), respectively. The calculated IC50 values for other CYP isoforms were >100 μM. JNJ-28431754 did not exhibit any potential for mechanism-based inactivation of the screened CYPs 1A2, 2C19, 2C9, 2D6, and 3A4. Overall, the drug has a low potential for involvement in CYP-based drug-drug interactions.

JNJ-28431754 is both a substrate and a weak inhibitor of p-glycoprotein (encoded by the MDR1 gene) and the multi-drug resistance associated proteins (MRP2) with IC50 of 19.3 μM (8.5 μg/mL) and 21.5 μM (9.5 μg/mL), respectively.

Following oral administration of 14C-JNJ-28431754 in male rats, a greater percentage of radioactivity was excreted in feces than urine at 25 hours after administration.

Clinical Data

Phase I Clinical Trials of JNJ-28431754

<u>A First-in-Human Study to Evaluate Safety, Tolerability, Pharmacokinetics and Pharmacodynamics of Single Escalating Oral Doses of JNJ-28431754 in Healthy Adult Male Subjects</u> (Protocol 28431754NAP1001)

The first Phase I study (Protocol 28431754NAP1001) to evaluate the safety, tolerability, pharmacokinetics (PK) and pharmacodynamics (PD) of single escalating oral doses of JNJ-28431754 is ongoing at a single study center in the Netherlands.

Part 1 of this study is a double-blind, randomized, placebo-controlled, sequential parallel groups, ascending single oral dose study in healthy normal male subjects. In each group (for each evaluated dose level), 6 subjects are randomized to JNJ-28431754 and 2 subjects to placebo. Subjects are admitted to the Clinical Research Unit (CRU) on Day –2 for baseline safety and PD assessments. On Day 1, after an overnight fast of ~10 hours, subjects receive study medication at ~8 AM, and have a standardized breakfast, lunch, and dinner at 0.5h, 4.5h, and 10.5h, respectively, post-dose. Subjects are domiciled in the CRU for frequent safety, tolerability, PK, and

PD assessments at specified time points until Day 5, when they may be discharged. Subjects return to the CRU for the final safety follow-up visit at 10-14 days post-dose. Assessments in Part 1 include:

Safety and tolerability

Adverse events, physical examination including whole body skin exam, vital signs, daily fluid intake and urine volume, telemetry and 12-lead ECGs, laboratory tests [routine hematology, clinical chemistry, urinalysis (including electrolytes, osmolality, protein, amino acids, tubular enzymes), and fecal occult blood tests (FOBTs)].

Pharmacokinetics

Blood and urine sampling for determination of plasma and urine JNJ-28431754 concentrations and potential metabolite metabolite profiling and/or metabolite quantification.

Pharmacodynamics

Cumulative urinary glucose excretion (UGE, in grams) over each 24-hour period (UGE/24h), UGE rate (grams/hr) over each timed urine collection interval, and 24-hour plasma glucose and insulin profiles.

In Part 2 of this study, the effect of co-administering JNJ-28431754 together with a standard high-fat breakfast, compared to administration in the fasting condition, on the PK and PD of JNJ-28431754 will be evaluated in 8 healthy male subjects. Part 2 is a balanced, 2-way crossover design with a washout period of at least 14 days between doses. Safety and tolerability will also be assessed as in Part 1.

As of March 21, 2007, 7 cohorts (total of 55 healthy men) have been dosed in Part 1 of this study at doses of 10, 30, 100, 200, 400, 600 and 800 mg of JNJ-28431754 or placebo (in the 800 mg cohort, 7 subjects have been dosed). A summary of safety, tolerability, PK and PD based on blinded, preliminary data from the 7 completed cohorts of Part 1 are included below.

Safety and Tolerability

Single oral doses of JNJ-28431754 at 10, 30, 100, 200, 400, 600 and 800 mg as a liquid suspension (5 or 50 mg/mL in 0.5% hypromellose vehicle) or matching placebo (0.5% hypromellose) have been generally well-tolerated.

To date, there have been no deaths, serious adverse events, or discontinuations due to adverse events.

Adverse Events

In the 7 cohorts of Part 1 of this study, 22 (40 %) of 55 subjects experienced at least 1 treatment-emergent adverse event (See blinded listing, Table 1). The majority of adverse events were either mild (43 [96%] of 45) or moderate (2 [4%] of 45) in severity. The most frequent adverse events were fatigue and headache (each 4 [9%] of 45), followed by postural dizziness (3 [7%] of 45). Twenty-four (24) of 45 (53%) adverse events were considered possibly related to the study drug. The remaining adverse events were considered either not related (14 [31%] of 45), or of doubtful relationship to study drug (7 [16%] of 45). No hypoglycemia was observed at any dose level. There was no apparent relationship between dose level and the type, severity or incidence of adverse events.

In Cohort 1 (10 mg or placebo), Subject 1005 reported moderate chest discomfort at 1-hour post dose, which lasted about 17 minutes, but without changes in vital signs or ECG parameters. This subject also developed mild, localized cutaneous erythema on the anterior surface of both knees 5 days after dosing, which had resolved by the follow-up visit. Subject 1002 developed transient postural hypotension accompanied with dizziness, which lasted about 2 minutes, at 2 hours post dose [Supine systolic blood pressure: 115 mmHg, Standing systolic blood pressure: 92 mmHg, Systolic blood pressure (Standing-Supine): -23 mmHg; Supine diastolic pressure: 55 mmHg, Standing diastolic blood pressure: 33 mmHg, Diastolic blood pressure (Standing-Supine): -22 mmHg; Supine pulse rate: 64 bpm, Standing pulse rate: 75 bpm, Pulse rate (Standing-Supine): 11 bpm].

In Cohort 2 (30 mg or placebo), Subject 1016 reported persistent whole body dry skin, mild in severity, on Day 3 post dose. The dry skin resolved at the follow up visit.

In Cohort 3 (100 mg or placebo), Subject 1020 was observed to have a mild, multi focal macular rash on the dorsal surface of both feet beginning at about 3 days post dose. The rash was accompanied by mild pruritis. The rash and pruritis resolved on the following day.

In Cohort 4 (200 mg or placebo), Subject 1028 complained of a burning skin sensation over his whole body approximately 30 minutes post dose. The sensation lasted about 1 hour. There was no skin abnormality observed. Subject 1029 reported fatigue about 2 hours post dose, which resolved 3.5 hours post dose. There were no clinically meaningful changes in any of his clinical chemistry lab test results, including plasma glucose and serum electrolytes on Day 1.

In Cohort 5 (400 mg or placebo), Subject 1033 reported fatigue that started 1-hour post dose and lasted 21 hours. The subject also reported abdominal fullness on Day 2, which resolved by his follow-up visit. The subject excreted 62 and 39 g of glucose in the urine over 0-24 and 24-48 hours post-dose, respectively. There were no significant changes in his urine volume, urine sodium excretion and serum electrolytes post dose compared to his baseline values. Subject 1036 experienced mild myalgia beginning at 48 hours post dose that resolved during his follow-up visit. The subject had normal CPK and LFT values post dose. His UGE/24 values were 72 and 33 g on Days 1 and 2, respectively. The subject's urine volume and urine sodium excretion were not significantly changed post dose compared with his baseline values. There were slight decreases in his serum sodium (131 mmol/L) and calcium (2.08 mmol/L) levels on Day 1 compared to his baseline Day -1 values (serum sodium: 138 mmol/L; serum calcium: 2.18 mmol/L). His serum sodium and calcium values returned to within the normal range on Day 2. Subject 1040 experienced a warm feeling and moderate headache about 6 hours post dose, which lasted about 7 hours. There were no clinically significant abnormalities in his vital signs and ECGs. The subject excreted 86 and 55 g of glucose in the urine over 0-24 hour and 24-48 hours, respectively. There were no significant changes in his urine volume, urine sodium excretion and serum electrolytes post dose compared with the baseline values.

In Cohort 6 (600 mg or placebo), Subject 1043 developed postural hypotension (Note: This adverse event was reported by the investigator as postural dizziness) 4-hours post dose [Supine systolic blood pressure: 118 mmHg, Standing systolic blood pressure: 84 mmHg, Systolic blood pressure (Standing-Supine): -34 mmHg; Supine diastolic pressure: 63 mmHg, Standing diastolic blood pressure: 53 mmHg, Diastolic blood pressure (Standing-Supine): -10 mmHg; Supine pulse rate: 75 bpm, Standing

pulse rate: 105 bpm, Pulse rate (Standing-Supine): 30 bpm]. The postural hypotension was accompanied by a transient dizziness, which lasted 4 minutes. The subject excreted about 90 and 63 grams of glucose in the urine over the 0-24 and 24-48 hour urine collection intervals. There were no increases in his urine volume and sodium excretion post dose compared with the baseline values. His serum electrolytes on Days 1 and 2 were comparable to his baseline Day –1 values. Two subjects reported adverse skin reactions. Subject 1044 experienced a 4-5 cm diameter area of cutaneous erythema on his neck 3 days post dose. The erythema was accompanied by mild pruritis and resolved during the follow up period. Subject 1045 developed a few acne-like pustules on his back and extremities on Day 2. The pustules resolved on Day 5. Three subjects experienced mild adverse gastrointestinal reactions. Subject 1044 reported abdominal cramps beginning at about 2 hours post dose, which lasted about 2 hours. Subjects 1043 and 1045 reported loose stool at about 5 and 2 hours post dose, lasting about 2 days and 5.5 hours, respectively.

In Cohort 7 (800 mg or placebo), all events were mild in intensity. Subject 1052 experienced whole body dry skin, mild in severity, on Day 2 post dose. The dry skin lasted about one day. Subject 1056 developed mild diarrhea (loose stool 5 times) beginning at about 1 hour post dose, which lasted about 12 hours. The diarrhea was not accompanied by any other symptom.

Table 1: Blinded Incidence of Treatment Emergent Adverse Events by System Organ Class and Preferred Term
(Study JNJ28431754-NAP1001-Part 1: Preliminary Safety Analysis Set)

**Body System or Organ Class**<br>Preferred Term	10mg or placebo (N=8) n (%)	30mg or Placebo (N=8) n (%)	100mg or placebo (N=8) n (%)	200mg or placebo (N=8) n (%)	400mg or placebo (N=8) n (%)	600mg or placebo (N=8) n (%)	800mg or placebo (N=7) n (%)
**Total Number of Subjects With Adverse Events**	4 (50)	1 (13)	2 (25)	2 (25)	4 (50)	6 (75)	3 (43)
**Gastrointestinal disorders**	1 (13)	0	0	0	2 (25)	2 (25)	1 (14)
Abdominal distension	0	0	0	0	2 (25)	0	0
Abdominal pain	1 (13)	0	0	0	0	1 (13)	0
Constipation	0	0	0	0	1 (13)	0	0
Diarrhoea	0	0	0	0	0	1 (13)	1 (14)
**General disorders and administration site conditions**	3 (38)	0	0	1 (13)	3 (38)	2 (25)	0
Catheter site related reaction	1 (13)	0	0	0	1 (13)	0	0
Chest discomfort	1 (13)	0	0	0	0	0	0
Fatigue	0	0	0	1 (13)	2 (25)	1 (13)	0
Feeling hot	1 (13)	0	0	0	1 (13)	1 (13)	0
**Infections and infestations**	1 (13)	0	1 (13)	0	1 (13)	1 (13)	0
Nasopharyngitis	1 (13)	0	1 (13)	0	1 (13)	0	0
Rash pustular	0	0	0	0	0	1 (13)	0
**Musculoskeletal and connective tissue disorders**	0	0	0	0	1 (13)	1 (13)	0
Back pain	0	0	0	0	0	1 (13)	0
Myalgia	0	0	0	0	1 (13)	0	0
**Nervous system disorders**	2 (25)	0	1 (13)	1 (13)	1 (13)	2 (25)	1 (14)
Dizziness	1 (13)	0	1 (13)	0	0	0	0
Dizziness postural	1 (13)	0	0	0	0	2 (25)	0

Note: Percentages calculated with the number of subjects in each group as denominator.
Coding Dictionary Version: MEDDRA V9.1

Table 1: Blinded Incidence of Treatment Emergent Adverse Events by System Organ Class and Preferred Term (Continued)
(Study JNJ28431754-NAP1001-Part 1: Preliminary Safety Analysis Set)

Body System or Organ Class<br>Preferred Term	10mg or placebo (N=8) n (%)	30mg or Placebo (N=8) n (%)	100mg or placebo (N=8) n (%)	200mg or placebo (N=8) n (%)	400mg or placebo (N=8) n (%)	600mg or placebo (N=8) n (%)	800mg or placebo (N=7) n (%)
**Nervous system disorders (continued)**							
Headache	0	0	1 (13)	1 (13)	1 (13)	0	1 (14)
Paraesthesia	1 (13)	0	0	0	0	0	0
**Respiratory, thoracic and mediastinal disorders**	0	0	0	0	0	1 (13)	1 (14)
Pharyngolaryngeal pain	0	0	0	0	0	1 (13)	1 (14)
**Skin and subcutaneous tissue disorders**	1 (13)	1 (13)	1 (13)	1 (13)	0	1 (13)	2 (29)
Dry skin	0	1 (13)	0	0	0	0	1 (14)
Erythema	1 (13)	0	0	0	0	1 (13)	0
Pruritus	0	0	0	0	0	1 (13)	0
Rash macular	0	0	1 (13)	0	0	0	0
Skin burning sensation	0	0	0	1 (13)	0	0	0
Skin irritation	0	0	0	0	0	0	1 (14)
**Vascular disorders**	1 (13)	0	0	0	0	0	0
Orthostatic hypotension	1 (13)	0	0	0	0	0	0

See footnotes on the first page of the table.

Clinical laboratory tests

No clinically significant treatment-emergent changes were observed in hematology, clinical chemistry and urinary analyses. Although values for some parameters were slightly outside the normal range at isolated time points for all treatments, these changes were transient and were not considered clinically significant. Except for dose-dependent increases in urinary excretion of glucose - reflecting the expected pharmacological effect of the study drug - there were no dose-dependent increases or decreases in serum electrolytes and osmolality, urinary excretion of electrolytes, creatinine, albumin and tubular enzymes. Daily Fecal Occult Blood Tests (FOBTs) were negative for all subjects.

Vital signs

There were no indications of any consistent treatment effect on supine or standing systolic and diastolic blood pressure or pulse rate following administration of the study drug. Two subjects developed mild, transient postural hypotension accompanied by dizziness: Subject 1002 at 2-hour post dose (10 mg) and Subject 1043 at 4-hour post dose (600 mg) as described in the adverse event section, above.

There were no indications of any consistent, or dose-dependent treatment effects on daily fluid intake and urinary volume, despite significant, dose-related increases in UGE over the 0-24 hr and 24-48 hr post-dose periods.

12-lead electrocardiograms

There were no consistent or dose-related changes in the 12-lead ECG parameters (heart rate, PR interval, QRS interval, QT interval, QTcB [Bazett's correction] or QTcF [Fridericia's correction] following administration of JNJ-28431754. There were no abnormalities noted during the post-dose telemetry monitoring period (0-6 hours post dose).

Pharmacokinetics

Preliminary plasma concentration time profiles and PK parameters for JNJ-28431754 following single oral doses of 10 to 800 mg are presented in Figure 1 and 2 and Table 2. JNJ-28431754 was rapidly absorbed into the systemic circulation with median t_{max} values in the range of 1.25 to 1.75 hours. Mean C_{max} and $AUC_{0-\infty}$ values increased with increasing dose up

to 600 mg. Normalization of individual C_{max} or $AUC_{0-\infty}$ values with the administered dose also demonstrated consistent systemic exposures up to 600 mg dose. Thereafter an increase in dose from 600 mg to 800 mg resulted in less than proportional increase in the AUC values. The Cmax values were comparable between 600 mg and 800 mg doses. These results suggest that the gastrointestinal absorption of JNJ-28431754 may have saturated at the 600-800 mg dose level.

The elimination of JNJ-2841754 appeared to be biphasic, with mean (SD) apparent terminal elimination half-lives (t1/2) ranging from 8.39 (4.20) to 13.6 (1.49) hours across the dose range. The mean (SD) clearance and apparent volume of distribution values of JNJ-2841754 across all doses ranged from 15.1 (2.62) to 22.4 (5.59) L/h, and from 240 (8.3) to 310 (37.4) L, respectively.

Figure 1: Mean plasma concentration-time profiles of JNJ-2841754 in healthy normal male subjects following a single oral dose of JNJ-2841754-linear scale (preliminary results, Protocol JNJ-2841754 NAP1001) (Note: only a 24-hour profile included for the 800 mgdose group)

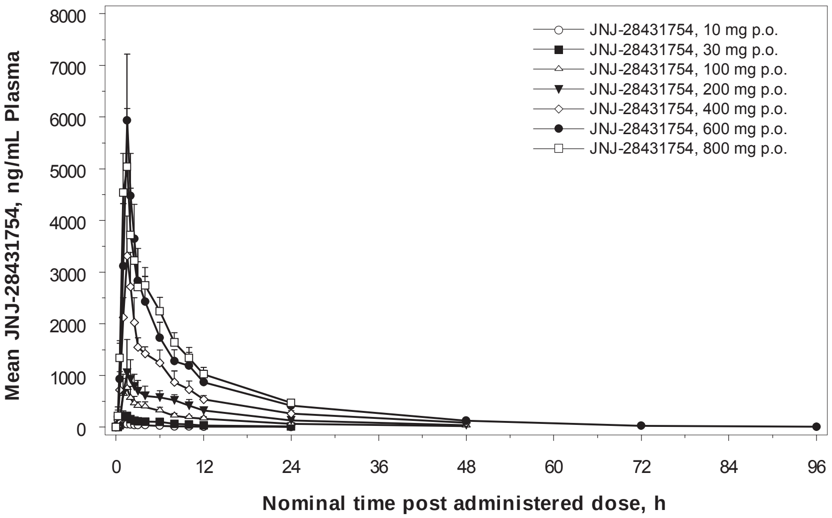

Figure 2: Mean plasma concentration-time profiles of JNJ-2841754 in healthy normal male subjects following a single oral dose of JNJ-2841754-logarithmic scale (preliminary results, Protocol JNJ-2841754 NAP1001) (Note: only a 24-hour profile included for the 800 mg dose group)

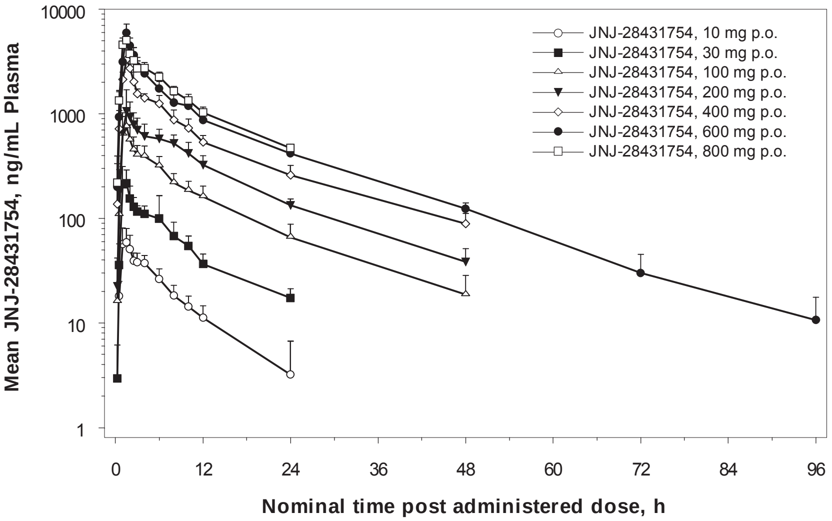

Table 2: Mean (SD) plasma JNJ-28431754 pharmacokinetic parameters in healthy normal male subjects following a single oral dose of JNJ-2841754 (Preliminary Results, Protocol 28431754-NAP-1001)

Dose	N	$AUC_{0\text{-}24\ h}$	AUC_{∞}	C_{max}	t_{max}[a]	$t_{1/2}$	CL/F	Vd/F
(mg)		ng.h/mL	ng.h/mL	ng/mL	h	h	L/h	L
10	6	408 (70.0)	471 (118)	67.5 (24.9)	1.50 (1.00-2.00)	8.39 (4.20)	22.4 (5.59)	245 (84.3)
30	6	1428 (188)	1685 (298)	254 (49.9)	1.25 (1.00-6.00)	9.52 (1.99)	18.2 (2.99)	245 (30.7)
100	6	5094 (1169)	6426 (1772)	755 (313)	1.50 (1.50-4.00)	11.3 (1.34)	16.4 (3.54)	263 (40.1)
200	6	9315 (707)	12052 (1065)	1164 (523)	1.75 (1.50-8.00)	11.7 (1.70)	16.7 (1.50)	280 (32.1)
400	6	19702 (2439)	25665 (3862)	3322 (759)	1.50 (1.00-1.50)	13.6 (1.49)	15.9 (2.38)	310 (37.4)
600	6	31771 (6500)	40639 (6204)	5935 (1288)	1.50 (1.50-1.50)	13.2 (3.21)	15.1 (2.62)	297 (129)
800	6	35643 (3.10)	41630 (4.10)	5210 (20.8)	1.50 (1.00-1.50)	8.66 (10.5)	19.2 (4.10)	240 (8.30)

[a]Data presented as Median (Min-Max)

Pharmacodynamics (PD)

Preliminary PD results summarized below are based on blinded data from Cohort 1 to Cohort 7 (10 mg to 800 mg JNJ-2841754). The PD parameters evaluated were the amount of urine glucose excretion (UGE), the rate of urine glucose excretion, plasma glucose concentrations, and plasma insulin concentrations.

UGE increased with higher doses, but in a less than dose-proportional (or plasma JNJ-2841754 AUC-proportional) manner. Also, the difference in the mean UGE over the first 24 hours post dose was minimal between the 400 and 800 mg doses, indicating that the UGE response in healthy male subjects may be maximal at or near the 400-800 mg dose level (Table 3; Note: As the mean UGE values in Table 3 include the placebo subjects, the reported UGE values are expected to be lower than that for the treated subjects).

Elevated urine glucose excretion occurred 2 to 7 hours after dosing at all dose levels. The maximal UGE rate was approximately 3.5 g/h at the 400 - 800 mg dose range. The mean total UGE values on Day 2 post dose (in 24-48-hr urine collection interval) became substantial over the dose range of 100 mg - 800 mg. At these dose levels, the UGE rates achieved were about 2-3 g/h over the first 13 hours post dose and about 0.5-2 g/h over 13 to 48 hours post dose.

Table 3: Mean (SD) Amount Urine Glucose Excreted (UGE) For All Subjects
(Preliminary results: Protocol JNJ-28431754-NAP-1001)

Dose	n		Urine Glucose Excreted (g)	
			0-24 hrs	24-48 hrs
10 mg/Placebo	8	Mean	6.16	0.532
		(SD)	(4.40)	(1.31)
30 mg/Placebo	8	Mean	13.1	0.648
		(SD)	(10.2)	(0.990)
100 mg/Placebo	8	Mean	32.7	9.20
		(SD)	(21.0_)	(6.49)
200 mg/Placebo	8	Mean	36.5	14.5
		(SD)	(23.7)	(11.7)
400 mg/Placebo	8	Mean	47.9	30.0
		(SD)	(31.5)	(21.4)
600 mg/Placebo	8	Mean	51.9	35.9
		(SD)	(33.6)	(24.5)
800 mg/Placebo	7	Mean	56.1	49.8
		(SD)	(27.3)	(26.5)

In Part 1 of this single ascending dose study, blood samples for assessment of 24-hour plasma glucose and insulin profiles were obtained at predefined time points over 24 hours post dose. Mean postprandial plasma glucose excursions appeared to decrease in a dose dependent manner after a single oral administration of JNJ-28431754, particularly during the morning meal.

Mean postprandial plasma insulin levels also decreased in a dose dependent manner after a single oral administration of JNJ-28431754.

No hypoglycemia was observed in any subject at all dose levels. The absence of hypoglycemia is consistent with the anticipated mechanism of pharmacological action for this drug, ie, plasma glucose is not expected to fall below the renal glucose threshold in healthy or diabetic subjects

1.2. Overall Rationale for the Study

This will be the first multiple dose study of JNJ-28431754, a potent, orally available, selective inhibitor of the renal sodium glucose co-transporter (SGLT2) in subjects with Type 2 diabetes mellitus (T2DM).

Results from the ascending dose study in healthy men indicated that single oral doses of JNJ-28431754 up to 800 mg have been generally well tolerated, as summarized in Section 1.1 of this protocol. There were no serious adverse events, no subjects were discontinued from the study, and there were no clinically significant changes or treatment-related trends in clinical laboratory values, physical examinations, vital signs, or ECG parameters. Those observations support further evaluation of JNJ-28431754 in T2DM subjects. Type 2 diabetes affects both men and women, and thus evaluating the safety, tolerability and effects of multiple JNJ-28431754 dosing in women as well as in men, is warranted to support further clinical development of this new agent. The data from this study will be used to design and guide dose selection for future studies of JNJ-28431754 in T2DM subjects involving treatment durations greater than 14 days.

2. OBJECTIVES

The overall objectives are to evaluate the safety, tolerability, pharmacokinetics (PK) and pharmacodynamics (PD) of JNJ-28431754 after single and multiple ascending oral doses of JNJ-28431754 in subjects with Type 2 Diabetes Mellitus (T2DM). It is hypothesized that daily oral administration of JNJ-28431754 for 2 weeks, at safe and well-tolerated dose levels, will lower mean plasma glucose concentrations ($AUC_{0\text{-}24hr\text{-}glucose}$) in diabetic patients to a clinically meaningful extent, compared to baseline and/or to placebo administration.

3. OVERVIEW OF STUDY DESIGN

Five (5) Cohorts, total of 100 subjects, 20 in each cohort, will participate in this study, with potential for an additional cohort of 20 subjects.

A blood sample will be collected on Day 1 from subjects consenting to the pharmacogenomic component of the study. This will allow for pharmacogenomic analysis, as necessary. Subject participation in pharmacogenomic research is optional and requires separate informed consent. Refusal to consent for this component does not exclude a subject from participation in the clinical study.

3.1. Study Design*

This is a randomized, double-blind, placebo-controlled, single and multiple (14 days) ascending dose, parallel group study. Five cohorts of subjects with T2DM (20 subjects per cohort) will be studied. One dose level will be evaluated in each cohort. The study will be conducted at multiple sites.

Sixteen (16) subjects will be randomized to JNJ-28431754 and to matching placebo within each cohort. Doses planned for evaluation are 30, 100, 300 and 600 mg/day. An additional cohort of Asian subjects will also be evaluated at a dose level, which was previously tested in a prior cohort and considered to be well tolerated. Following initial screening (conducted within 35 days prior to Day -3 of the study), eligible subjects will be instructed to withdraw from their previous antidiabetic medications for 16 days prior to dosing on Day 1. Blood glucose levels will be monitored daily during the 16-day washout period. Eligible subjects will be admitted to the Clinical Research Unit (CRU) on Day – 3. On Days –2 and -1, subjects will receive placebo once daily in a single-blind fashion (subjects blinded) and undergo baseline safety and PD assessments. At least fifty percent of subjects in each cohort should be targeted to have fasting blood glucose ≥170 mg/dL (9.45 mM) and a MDRD calculated GFR ≥ 80 (mL/min/1.73 m^2). Eligible subjects will then be randomized to a double-blind treatment with JNJ-28431754 or placebo. A single dose of study medication will be administered on Day 1, followed by periodic safety, PK and PD assessments. Daily dosing will resume on Day 3 and continue through Day 16 (14 days of

* This section of the protocol has been revised. Please refer to the section of this document titled PROTOCOL AMENDMENTS (Amendment DEU-5, 27 September 2007) for a detailed description of the specific changes.

dosing). Each daily dose will be administered at approximately 8 AM followed by a standardized breakfast within 10 minutes after dosing. Standardized lunch and dinner will be provided at 4.5 and 10.5 hours post dose, respectively. Subjects will be discharged from the CRU on Day 20 (96 hours post last dosing) and return to the CRU for safety assessments and PK sample collections on the mornings of Days 21 to 22. Subjects will also return within 7-10 days following the Day 22 outpatient visit for a final Follow-up visit.

Subjects must have been diagnosed with Type 2 diabetes at least 12 months prior to study start and will have been stably managed for at least 3 months prior to the screening visit on oral antidiabetic monotherapy (metformin, or a sulfonylurea, or a meglitinide (e.g., repaglininide or nateglinide), or a DPP-IV inhibitor (e.g., sitagliptin or vildagliptin), or alpha-glucosidase inhibitor (e.g., acarbose). Subjects will not have used any of the following anti-diabetic medications within 3 months of study start: exenatide, thiazolidinediones (e.g., rosiglitazone, or pioglitazone), or insulin.

Eligible subjects for this study will return for an outpatient visit around Day -17, to be counseled by a registered dietician to follow a standard weight maintaining diet for about 2 weeks prior to admission and throughout the entire study except on Days –3 to 20 when subjects will receive standardized meals (See Attachment 8) (The weight maintaining diet will follow standard recommendations of the American Diabetes Association, comprising ~50-60% carbohydrate, 15-20% protein, and ~25-35% fat, with total daily caloric intake individually adjusted for BMI). In addition, subjects will receive instruction in signs and symptoms of hypoglycemia, use of a portable home fingerstick blood glucose analyzer and reporting of results. During the 16-daywashout phase and during the post-treatment phase (between discharge and prior to the Follow-up visit), subjects will record their fasting blood glucose levels and the postprandial blood glucose levels after lunch on a diary card which will be provided to them and report the results to the clinic weekly, or at any time values exceed prespecified limits. This information will be collected by the clinic and recorded in the eCRF.

Subjects will resume their pre-study oral anti-diabetic medication regimen upon completion of the outpatient visit on Day 22 or potentially at any earlier time during the study if their confirmed fasting blood glucose levels

rise above 13.3 mM (240 mg/dL) or confirmed postprandial glucose levels rise above 19.4 mM (350 mg/dL) following lunch. Subjects who need to restart anti-diabetic medications during the study will stop receiving study medication but continue with study related safety procedures and daily PK samples (relative to predose time point only) for at least 3 days after the final dose. The Principal Investigator will determine the dose at which to reinitiate the subjects' medications and any subsequent dose adjustments.

The escalation of doses defined in this protocol may be modified if indicated based on evaluation of the safety, tolerability, pharmacokinetics or pharmacodynamics of prior cohorts. Modifications of any next dose can be a de-escalation, a reduced escalation step, or an increased escalation step. Twice-daily dosing may also be evaluated in one or more of the cohorts or additional cohorts of 20 subjects may be added. For any dose chosen for a possible twice-daily dosing regimen, the maximum predicted total plasma JNJ-28431754 exposure (AUC_{0-24} hr at steady-state) will not exceed the NOAEL established in the 2-week GLP toxicology study in rats (AUC_{0-24h}: ~ 80 μg.h/mL) based on the incidence of hyperostosis (minimal to mild changes in distal end of femur & proximal end tibia) at 150 mg/kg, without amendment to the protocol. Pharmacokinetic and pharmacodynamic sampling time points may be adjusted to accommodate a twice-daily dosing regimen but, the total blood volumes specified in the protocol will not be exceeded. For all cohorts, JNJ-28431754 dose levels that are predicted to produce steady state JNJ-28431754 exposures above the NOAEL in the 2-week GLP toxicology study in rats (AUC_{0-24h}: ~ 80 μg.h/mL) will not be exceeded without an amendment to this protocol.

Planned JNJ-28431754 Dose Levels					
Cohort[1]	N				
1	16+4[2]	30 mg or placebo			
2	16+4[2]		100 mg or placebo[3]		
3	16+4[2]			300 mg or placebo[3]	
4	16+4[2]				600 mg or placebo[3]
5	16+4[2]				30, 100 or 300 mg or placebo[3]

1 Initiation of dosing for each subsequent cohort at a higher dose level will be at least 17 days following the initiation of dosing in the prior cohort.

2 Sixteen on JNJ-28431754; four on placebo

3 The actual dose (mg) level selected for each cohort may be modified based on evaluation of preliminary safety, pharmacokinetic and pharmacodynamic data from previous cohorts.

An additional cohort will assess the safety, tolerability and effects of multiple doses of JNJ-28431754 on male and female Asian subjects at a dose level which was previously tested in an earlier cohort and considered to be well tolerated

Successive cohorts at a higher dose level will be started with at least a 17-day interval relative to the start of dosing in the preceding cohort, after review of adverse events, vital signs, electrocardiograms (ECGs) and laboratory tests of the preceding cohort collected up to and including Day 17 for preliminary safety data and Day 11 for PK data. Each subsequent escalated dose level shall be performed if, in the judgement of the Investigator and the J&JPRD Safety Physician, the results of the safety analysis of the preceding dose level are satisfactory. If a single dose at a given dose level is not well tolerated by individual subjects, the single dose may be repeated at a lower dose level, or the multiple dosing may start at a lower dose for those subjects or the entire cohort, in the judgement of the Investigator and the J&JPRD Safety Physician. At least 10 subjects are required to complete each treatment period prior to a decision to escalate to the next higher dose level. If fewer than 10 subjects complete any cohort, a separate Data Review Committee (DRC) may be convened to review unblinded data and to provide a recommendation regarding dose escalation.

3.2. Study Design Rationale

This is the first single and multiple dose study with JNJ-28431754 in Type 2 diabetic subjects.

This will be a double-blind study in order to avoid subjective bias in the assessment of safety/tolerability and pharmacological effects of the study drug. Placebo will be administered as a control in order to establish the frequency and/or magnitude of changes in clinical endpoints that may occur in the absence of active drug treatment.

Based on the mechanism of action of an SGLT-2 inhibitor, the effects of JNJ-28431754 on urinary glucose excretion and plasma glucose concentration are expected to be greater in hyperglycemic diabetic subjects as compared in healthy, euglycemic subjects. Enrolling diabetic subjects in this study allows early assessment of safety and efficacy of JNJ-28431754 in the intended target population under safe and well-controlled condition, and thus will guide subsequent clinical development.

Type 2 diabetes affects both men and women, and thus evaluating the safety, tolerability and effects of multiple JNJ-28431754 dosing in women as well as in men, is warranted to support further clinical development of this new agent. In the present multiple dose study, since reproductive toxicology studies have not yet been completed, only women of non-childbearing potential (post-menopausal, or surgically sterile) are to be enrolled initially. That restriction will limit the number of female Type 2 diabetics who would otherwise be eligible to participate in this study. In the 2-week GLP toxicology studies in both rats and dogs, systemic exposure of JNJ-28431754 was generally higher in females (up to 2 fold) than in males. Female subjects of non-child bearing potential will be enrolled in this study to allow exploring potential differences in safety and PK between males and females.

Although subjects of Asian ethnicity have not been explicitly enlisted for the current protocol, based on the likely study demographics, relatively few Asian subjects are likely to be enrolled in each cohort. Since the incidence of T2DM is rapidly increasing in Asia, this new drug may be developed for use in Asian countries. To support potentially larger and longer clinical trials in Asian diabetics, an additional cohort will be studied that is comprised exclusively of Asian diabetics to allow exploring safety and efficacy in this population. Based on preclinical data related to JNJ-28431754 metabolism and clearance mechanisms, the PK and metabolism profiles of JNJ-28431754 in Asian subjects are not expected to be markedly different from that in Caucasians or other ethnic groups.

The dose selected for this additional Asian cohort will be a dose that has been already evaluated in a prior cohort as a safe and potentially efficacious dose.

Since this is the first study of JNJ-28431754 in Type 2 diabetic subjects, subjects in each cohort will first receive a single dose of JNJ-28431754 followed by frequent and carefully monitored safety assessments for 2 days, prior to initiating the 14-day multiple dosing period on Day 3.

The planned dose range for this study is 30, 100, 300 and 600 mg/day of JNJ-28431754. The starting dose is based on safety, tolerability and PK and PD data from the ongoing SAD study (Protocol JNJ-28431754 NAP-1001) in healthy male subjects. In that study, with JNJ-28431754 doses of 10 mg up to 800 mg, the mean urine glucose excretion (UGE) over 24 hours post dose (UGE/24h) increased less than dose proportionally, from 6 g to 56 g (including placebo subjects). At all dose levels, the increased UGE was not associated with clinically significant abnormalities in fluid or electrolyte balance. No hypoglycemia was observed at any dose level. Systemic plasma drug exposure following single doses of JNJ-28431754 increased dose proportionally as doses increased from 10 to 600 mg ($AUC_{0\text{-}inf}$: 0.5 to 41 μg.h/mL at doses 10 to 600 mg). The terminal half-life ranged from 8 to 14 hours. Based on the observed single dose PK parameters, no major drug accumulation (less than 2 fold) is anticipated following repeated QD administration of JNJ-28431754. Thus, 30 mg is considered a safe starting dose for the present multiple dose study. The selected dose range of 30 to 600 mg is expected to cover a potentially efficacious dose range with respect to increases in UGE and decreases in plasma glucose concentration (sufficient for expecting HbA1c reduction of at least 0.8% in longer duration trials). The predicted mean exposure ($AUC_{0\text{-}24h}$ at steady state) at the maximum dose for this study will not exceed the mean plasma exposure at the NOAEL dose (20 mg/kg) in the 2-week GLP toxicology study in rats ($AUC_{0\text{-}24h}$: ~ 80 μg.h/mL), an exposure at which no dose limiting organ toxicity was observed (mildly higher AST, BUN, minimally lower serum calcium and mildly to moderately lower blood glucose).

Preliminary data from the single ascending oral dose study in healthy normal men (Protocol JNJ-28431754 NAP 1001) suggests that at dosages of 400 mg and higher, JNJ-28431754 may have a greater effect than expected to lower

prandial glucose excursions (potentially by slowing the rate of gastrointestinal glucose absorption). Therefore, a BID regimen, with dose levels of 400 mg, given with the morning and evening meals, might have superior glucose lowering efficacy as compared to a QD regimen. To characterize this effect in diabetic subjects, a BID dose regimen may be studied in the present multiple dose study, at the high end of the planned dose range, assuming the planned QD dose levels are well tolerated.

Superficial and small (~1 mm diameter) gastric erosions had been observed in some animals in a 2-week rat toxicology study. The first-in-human single ascending oral dose Phase I trial (JNJ-28431754 NAP-1001) therefore had fecal occult blood tests (FOBTs) performed daily on Days 1-4 post-dose. Subsequent investigative rat toxicology studies indicate that the histologically very acute gastric erosions noted in rats are attributable to acute stresses induced by certain study procedures conducted just prior to necropsy. Those rat erosions are therefore no longer considered as primary, direct effects of JNJ-2843175. On that basis, and also since in the single ascending oral dose study (JNJ-28431754 NAP-1001) all FOBTs at every dose level have been negative, FOBTs are not included in the present trial. Nonetheless, all T2DM subjects in this study will be closely monitored for signs and symptoms of adverse gastrointestinal effects. Preliminary results of a currently ongoing 3-month rat toxicology study with JNJ-28431754 (using modified pre-terminal necropsy procedures) are anticipated to become available during early stage of the present study. Any unexpected results from that 3-month rat toxicology study that are potentially related to adverse GI effects will be evaluated in relation to the clinical monitoring procedures in the present multiple-dose study.

At the highest planned dose of 600 mg/day in this multiple-dose study, mean plasma JNJ-28431754 exposures at steady-state are expected to approximate the JNJ-28431754 exposures attained at the highest tested, 800 mg dose (which was evaluated as being well-tolerated) in the single ascending oral dose study (JNJ-28431754 NAP-1001). Recently, in an ongoing 3-month dog toxicology study, during the first week at the highest tested dose of 200 mg/kg/day only, 3 of 5 male dogs in each gender had blood in stools. This adverse GI effect resolved upon dose reduction to 100 mg/kg/day. No abnormal microscopic or gross pathological findings were observed in the previous 2-week GLP toxicology study in dogs, even at daily doses up to

400 mg/kg/day. In the ongoing 3-month GLP toxicology study in dogs, the mean plasma JNJ-28431754 steady-state exposures at 100 mg/kg/day are conservatively expected to be in the range of approximately 264,000-445,000 ng.hr/mL and 32,000-40,000 ng/mL, for $AUC_{0\text{-}24}$ and $C_{max,}$ respectively. Compared to those plasma exposures in dogs, human plasma JNJ-28431754 exposures in the last cohort at the highest escalated dose of 600 mg/day in this study are expected to substantially lower, by about 6- to 11-fold (for $AUC_{0\text{-}24}$) and about 5- to 7-fold (for C_{max}). Nonetheless, at all dose levels in this study, subjects will be closely monitored for signs and symptoms of adverse GI effects. Preliminary results from the ongoing 3-month toxicology study in dogs are scheduled to become available during early stage of the present study, and any unexpected results from that 3-month dog toxicology study which are potentially related to adverse GI effects will also be evaluated in relation to the clinical monitoring procedures in the present multiple-dose study.

Diabetic subjects who participate in this study will temporarily discontinue their prior anti-diabetic medication(s) in order to clearly demonstrate the urine glucose excretion and blood glucose lowering effects of monotherapy with JNJ-28431754. In addition discontinuing their prior anti-diabetic medications will minimize potential drug-drug interactions that have not been investigated. A 16-day washout period preceding dosing is adequate to allow washout of the drug exposures and pharmacological effects of the piror anti-diabetic medication(s) of the eligible subjects. This washout period also allows subjects to establish modestly elevated and stabilized fasting blood glucose levels (7.8 to 13.3 mM or 140 to 240 mg/mL) such that effect of JNJ-28431754 on plasma glucose concentration can be adequately determined. Subjects will be closely monitored for their blood glucose levels throughout the study, including during the 16-day washout period. Any subject will be discontinued from the study and be treated by the investigator with appropriate hypoglycemic agents if his/her confirmed (by conventional venous sample) fasting blood glucose level is above 13.3 mM (240 mg/dL), or confirmed postprandial glucose level rises above 19.4 mM (350 mg/dL) following lunch. It will be possible to rapidly resume anti-diabetic therapy. While chronically elevated (for years) and/or uncontrolled hyperglycemia in T2DM patients can lead to serious diabetic complications, short-term discontinuation of oral anti-diabetic medications (for a maximum

of approximately 6 weeks in this study) with modest blood glucose elevations and with daily medical monitoring is not expected to result in clinically meaningful short- or long-term consequences for the study subjects.

Because SGLT-2 inhibitors cause the net loss of calories from the body in the form of glucose, these compounds also have potential to reduce body weight. However, the increase in urinary glucose excretion might lead to a compensatory increase in food intake. In this study, food intake cannot be directly measured, as subjects will receive standard meals to ensure reliable determination of drug effect on the 24-hour plasma glucose profile, the primary end point. Visual Analogue Scales (VAS) are commonly used to assess appetite and satiety sensations, which correlate well with food intake. In this study, the VAS will be assessed at baseline and post dose at predefined times using a validated study procedure.[12]

Based on available data from preclinical JNJ-28431754 toxicology studies in rats and dogs, potential adverse human effects may occur. These include, but are not limited to, osmotic diuresis due to increased urinary glucose excretion, changes in serum or urine electrolytes, GI intolerability, hypoglycemia, changes in bone formation and or in bone resorption, abnormalities in hepatic and renal functions, and phototoxicity. Subjects will be closely monitored during study using well-established clinical procedures and laboratory tests, including monitoring of daily fluid intake and urine volume, serum and urine electrolytes for monitoring body fluid and electrolyte imbalance, self-monitoring using a glucometer as well laboratory assessment of plasma glucose levels to monitor potential hypo-/hyperglycemia, serum and urine biomarkers for monitoring bone turnover and protection from sunlight exposure during the study.

3.2.1. Rationale for DNA Collection

It is recognized that genetic variation within the population can be an important contributory factor to inter-individual differences in drug distribution and response and can also serve as a marker for disease susceptibility and prognosis. An association between a genetic polymorphism and clinical outcome may help to explain inter-individual variability in that outcome and may help to identify population subgroups that respond differently to a drug. The overall goal of the pharmacogenomic

component is to allow for the identification of genetic factors that may influence pharmacokinetics, pharmacodynamics or safety/tolerability of JNJ-28431754. DNA samples will be collected in this clinical study in order to help address emerging clinical issues and to enable the development of safer, more effective, and ultimately individualized therapies in the future. Further details are provided in Section 9.4.

3.3. Subject Safety and Stopping Criteria

After each cohort, the decision to continue with the next higher dose will be made jointly by the Principal Investigator and the Sponsor after review of all available blinded clinical safety and tolerability data, and pharmacokinetic and pharmacodynamic data. Blinded interim safety and pharmacokinetic data will be provided to the J&JPRD Safety Physician or designee prior to the planned dose escalation meeting with the Principal Investigator. Dose escalation decisions will take into account the incidence, severity and duration of all adverse events reported in prior cohorts.

This study will enroll diabetic subjects who will discontinue temporarily their current oral anti-diabetic medications in order to investigate the blood glucose lowering effects of JNJ-28431754. A 16-day washout preceding dosing in this study is adequate to allow washout of the plasma concentrations and pharmacological effects of sulfonylurea, meglitinide, metformin and DPP-IV inhibitor treatments, and to establish relatively stable blood glucose levels in these subjects, prior to Day 1. During that 16-day washout period, subjects will be trained to measure their fasting and postprandial blood glucose levels after lunch daily using a standard glucometer, and report those values to the study center for evaluation by the Principal Investigator, to ensure that no subject develops excessively high fasting blood glucose levels above 13.3 mM (240 mg/dL) and high postprandial values after lunch above 19.4 mM (350 mg/dL) during the washout period. Eligible study subjects will maintain a daily record of their fasting and prandial blood glucose levels during the pre-study washout period and the post-treatment phase. This information will be collected by the clinic and recorded. On Day –2 of this study, the fasting blood glucose levels in each subject must be within a well-tolerated hyperglycemic range of 7.8 – 13.3 mM (140-240 mg/dL) to continue in the study. Subjects will continue to measure their fasting (morning) and postprandial (post lunch) blood glucose levels throughout the study (during inpatient and outpatient

phases), and any subject with a confirmed fasting blood glucose elevated above 13.3 mM (240 mg/dL) or confirmed postprandial glucose levels above 19.4 mM (350 mg/dL) following lunch will not be further administered study medication, and will be treated with appropriate hypoglycemic therapy and monitored until blood glucose levels have stabilized.

In addition, if > 50 percent of subjects in any cohort experience an adverse event classified as moderate or severe by the Principal Investigator or one subject experiences a serious adverse event where the relationship to the study drug can not be excluded by the Principal Investigator, further dose escalation will not occur.

The sponsor's Safety Physician may request an internal Data Review Committee (DRC) to review unblinded data to decide the next dose or whether to stop or continue the study. This committee, with membership independent of the principal investigator, the safety physician and other members of the study team, will indicate their decision based on decision rules agreed upon prior to unblinding. The confidentiality of any unblinded information will be maintained until the study is terminated and the database is locked.

4. STUDY POPULATION

4.1. General Considerations

The specific inclusion and exclusion criteria for enrolling subjects in this study are described in the following sections.

4.2. Inclusion Criteria

Subjects must satisfy the following criteria to be enrolled in the study:

1. T2DM subjects who have been diagnosed for at least 12 months prior to study screening
2. T2DM subjects who have been taking a stable dose regimen of **oral** antidiabetic **monotherapy** except exenatide and thiazolidinedione (TZD) for at least 3 months prior to study screening.
3. Males or postmenopausal/surgically sterile females, age 25 to 65 years inclusive

[Note: (1) Post-menopausal is defined as no menses for at least 18 months prior to study start or no menses for 6 to 18 months prior to the start of this study, and plasma FSH must be ≥ 40 mlU/mL and estradiol ≤ 20 pg/mL in 3 separate measurements which will occur at screening, Day -17, and Day -2

(a -2 day window is allowed at Day -2); (2) Pre-menopausal surgically sterilized subjects must have a negative β-HCG pregnancy test at screening and at Day –2.

4. At screening: body mass index (BMI = weight in kg/height in m^2) of 20 to 39.9 kg/m^2; HbA1c of $\geq$ 7% and $\leq$ 8.5%
5. On Day –2, fasting blood glucose (FBG) concentrations between 7.8 mM (140 mg/dL) and 13.3 mM (240 mg/dL)
6. Subjects who have not smoked more than 10 cigarettes per day in the last 6 months prior to screening.
7. Subjects may enter who have well-controlled hypertension, but they must be on a stable regimen of allowed anti-hypertensive medications (see Section 8, and Attachment 10) for at least 3 months prior to screening,
8. At screening and Day -2: a) systolic BP of $\geq$95 and $\leq$ 160 mm Hg, supine, b) diastolic BP $\geq$ 50 and $\leq$ 100 mm Hg, supine, c) HR $\geq$ 50 beats per minute, supine, d) pulse rate increase of $\leq$ 30 beats per minute and a decrease of systolic BP of $\leq$ 20 mm Hg when standing from a supine position,
9. Subjects may enter with dyslipidemias, but they must be on a stable regimen of allowed dyslipidemic medications (see Attachment 10) for at least 3 months prior to screening.
10. Competency in speaking and comprehending the languages where the study will be conducted.
11. Willing to adhere to the prohibitions and restrictions specified in this protocol (Section 4.4).
12. Subjects must have signed an informed consent document indicating that they understand the purpose and the procedures of the study and are willing to participate in the study.
13. To participate in the optional pharmacogenomic component of this study, subjects must have signed the informed consent form for pharmacogenomic research indicating willingness to participate in the pharmacogenomic component of the study (where local regulations permit). Refusal to consent for this component does not exclude a subject from participation in the clinical study.

4.3. Exclusion Criteria

Potential subjects who meet any of the following criteria will be excluded from participating in the study:

1. Subjects who have a history of Type 1, "brittle" diabetes or secondary forms of diabetes

2. Subjects who have a known history of repeated severe hypoglycemic episodes as defined in Attachment 7
3. History of clinically significant diabetic complications, including: retinopathy (fundus photography within 3 months of screening required), nephropathy (GFR < 70 ml/min/1.73 m^2 based on the MDRD equation (See Section 9.5.2, or macro-albuminuria >200 mg/L), neuropathy, gastroparesis, or ketoacidosis
4. History of, or currently active, significant illness including (but not limited to) cardiovascular disease (including cardiac arrhythmias, myocardial infarction, stroke, peripheral vascular disease), hematological disease, respiratory disease, hepatic or gastrointestinal disease, endocrine/metabolic disorders (excluding Type 2 diabetes), neurologic or psychiatric disease, malignant neoplasms (other than adequately treated cutaneous basal or squamous cell cancers), or any other illness that the Investigator considers should exclude the subject
5. History of having taken insulin, TZDs, exenatide, thiazide diuretics, or beta-blockers, within 3 months of the screening visit
6. History of having taken oral steroids within 3 months of the screening visit, or anticipates a need to take oral steroids during the course of the study
7. History of having chronically taken aspirin (greater than 100 mg/day), NSAIDs, anticoagulants or other drugs known to interfere with blood clotting within 3 months of study start, or anticipates a need to take any of these during the course of the study
8. History of recent major surgery (within 6 months)
9. History or currently active renal diseases, nephrolithiasis, upper or lower urinary tract infections or prostatitis
10. History, or family history, of bleeding or coagulation disorders
11. History of gastritis and GI ulcer and resultant complications, e.g. GI bleeding, perforation and obstruction.
12. History of eating disorder (e.g. anorexia, bulimia) or recent significant changes in body weight due to dieting or nutritional treatment
13. Clinically significant abnormality in physical examination, vital signs, or 12-lead electrocardiogram (ECG) at screening or Day -2.
14. Clinically significant abnormal values for hematology, coagulation, fasting clinical chemistry or urinalysis (hemoglobin <8.4 mmol/L in males and <6.8 mmol/L in females; creatinine ≥ 110 µmol/L for males and ≥ 101 µmol/L for females; alanine aminotransferase (ALAT) and aspartate amino transferase (ASAT) ≥ 3 times upper normal limit; elevated HbA1c, fasting blood glucose values or mild abnormality in lipid profiles are allowed for diabetic subjects) at screening or Day-2 (Note: Subjects who have fasting triglyceride levels greater than 5.1 mmol/L are excluded)

15. History of disorders that are potential causes of GI bleeding including, but not limited to:
    - GI mass lesions, obstruction, perforation and diverticula
    - Inflammation (e.g., oesophagitis, gastritis, ulcer at any site of GI tract, ulcerative colitis and Crohn's disease)
    - Vascular disorders (vascular ectasia at any site of GI tract, e.g., internal hemorrhoids, portal hypertensive gastropathy or colonopathy, varices at any site of GI tract, haemangioma, GI vascular malformations)
    - Chronic GI infections (e.g., worm infestations, tuberculous enterocolitis, amoebiasis
    - Surreptitious bleeding (e.g., recurrent or chronic hemoptysis, oropharyngeal bleeding, or epistaxis)
16. Currently active, clinically significant skin disorders
17. History of hypersensitivity to sunlight or artificial source of intense light, especially UV light
18. History of recent travel (within 6 months) to locations that may predispose to the acquisition of communicable illnesses (e.g., parasitic or water-borne illnesses in developing tropical regions)
19. Tested positive for serology: hepatitis B surface antigen (HBsAg), hepatitis C antibodies (anti-HCV) or human immunodeficiency virus (HIV) antibodies.
20. Recent history of alcohol or drug abuse within 6 months prior to screening
21. History of or currently positive for alcohol and/or drugs of abuse (including barbiturates, opiates, cocaine, cannabinoids, amphetamines and benzodiazepines) at screening and Day -2
22. Clinically significant acute illness within 14 days prior to study drug administration
23. History of drug and/or food allergies, including allergies or intolerance
24. Donation of 1 or more units (approximately 450 mL) of blood or acute loss of an equivalent amount of blood within 90 days prior to study drug administration
25. Participated in any research study within the past 4 weeks or received an experimental drug or used an experimental medical device within 90 days prior to study drug administration
26. Use of any prescription or over-the-counter medications (including herbal medications, homeopathic remedies, or vitamins or mineral supplements (other than once-daily multivitamins approved by the PI) within 14 days prior to study drug administration [not including allowed concomitant

medications or occasional use of paracetamol/acetaminophen (Section 8, Attachment 10)

27. Male subjects who are not sterile or not willing to abstain from sexual intercourse for the duration of the study (and until 90 days after the last dose of study medication), ensure that their partner practices a highly effective method of birth control such as implants, injectables, combined oral contraceptives, or hormonal IUDs (intrauterine devices) according to Note for Guidance on Non-Clinical Safety Studies for the Conduct of Human Trials for Pharmaceuticals (CPMP/ICH/286/95, modification).
28. Any condition that in the opinion of the Principal Investigator would complicate or compromise the study, or the well being of the subject

4.4. Prohibitions and Restrictions*

Potential subjects must be willing to adhere to the following prohibitions and restrictions during the course of the study to be eligible for participation.

1. Weight maintaining diet must be adhered to from 2 weeks prior to admission (Day –3) and throughout the study as instructed by a registered dietician designated by the Principal Investigator. Willing to consume entirely the 3 same standard meals provided on Days –1, 1, and 16.
2. Strenuous exercise (e.g., long distance running ≥5 km/day, weight lifting, or any physical activity to which the subject is not accustomed) is to be avoided throughout the study, from screening through the follow-up visit
3. Alcohol consumption must be avoided for at least 72 hours prior to study drug administration until the final Follow-up visit.
4. Methylxanthine containing products (e.g., coffee, cola, tea, chocolate) and quinine-containing products (e.g., tonic water) are not permitted from 48 hours prior to study drug administration until at least 48 hours following the last administration of study drug.
5. Grapefruit, grapefruit juice, cranberry juice, Seville oranges, St John's wort, phlorizin, charcoal broiled foods, and any medication with CYP3A4 inhibition or induction properties (to allow a sufficient wash out/recovery period, particularly if the medication is a CYP3A4 mechanism-based inhibitor such as some of the macrolide antibiotics [e.g. erythromycin]; see Attachment 9) must not be consumed from 14

* This section of the protocol has been revised. Please refer to the section of this document titled PROTOCOL AMENDMENTS (Amendment DEU-5, 27 September 2007) for a detailed description of the specific changes.

days prior to study drug administration, or while confined to the clinical study center and continuing to the Follow-up visit.

6. Subjects will be instructed to abstain from poppy seed consumption at least 72 hours prior to the screening visit and prior to admission to the Unit.
7. Subjects must be advised not to donate blood for at least 90 days after completion of the study.
8. There is no information about the effect of JNJ-28431754 on sperm or its production in the body; nor is there information about any possible effects on the development of the fetus. It is important that male subjects' partners should not become pregnant during the study. As a precaution, male subjects should use appropriate contraception (e.g. condoms) during the time interval between taking the first dose and 3 months after taking the last dose of study medication. Subjects will be advised that partners should also use highly effective contraceptives (i.e. those with less than a 1% failure rate per year) which include implants, injectables, combined oral contraceptives, hormonal IUDs, sexual abstinence or vasectomized partner) according to Note for Guidance (CPMP/ICH/286/95, modification) during this period. Subjects should inform the principal investigator or the study physician if their partner becomes pregnant during the study.
9. Subjects must be advised not to donate sperm during the time interval between taking the first dose and for at least 90 days after completion of the study.
10. While in the study center subjects will not be allowed additional sugar in beverages or on cereal, though limited use of other sweeteners is allowed.
11. Following administration of the first dose of the study medication, subjects will be advised to avoid direct exposure to sunlight or artificial sources of intense light, especially UV light, up to 96 hours post the last drug administration, or if sunlight exposure cannot be avoided, to use protective clothing and broad-spectrum (UVA/UVB) sunscreens and sun block lipsticks that have a skin protection factor (SPF) of at least 15.

5. RANDOMIZATION AND BLINDING

5.1. Overview

Randomization will be used to avoid bias in the assignment of subjects to treatment, to increase the likelihood that known and unknown subject attributes (e.g., demographic and baseline characteristics) are evenly balanced across treatment groups, and to enhance the validity of statistical comparisons across treatment groups. Blinded treatment will be used to

reduce potential bias during data collection and evaluation of clinical endpoints.

5.2. Procedures

Prior to the study drug administration on the morning of Day 1 subjects will be assigned subject numbers as described below.

Cohort 1	1001 through 1020
Cohort 2	1021 through 1040
Cohort 3	1041 through 1060
Cohort 4	1061 through 1080
Cohort 5 (if conducted)	1081 through 1100

Within each cohort (1 through 4) subjects will be randomly assigned to receive JNJ-28431754 (n=16) or placebo (n=4). It is preferable (but not required) that an approximately equal number of subjects in each cohort will be included from each site. At least 2 additional subjects will be admitted on Day –3 and will undergo all assessments leading up to dosing to ensure that at least 20 subjects are dosed in each treatment cohort. Subjects who withdraw will be replaced at the discretion of the sponsor as soon as study logistics allow. Withdrawn subjects for whom the blind was broken will not be replaced. If subjects need to be replaced after dosing for any reason the replacement will be numbered with the prefix 2, whereby subject 2001 replaces 1001, etc.

At least 10 subjects are required to complete each cohort for the dose-escalation decision based on the preliminary blinded data, ensuring that in each cohort at least 1 subject would have received placebo. If less than 10 subjects complete a cohort and both the principal investigator (PI) and the Sponsor still wish to consider dose escalation, or a safety concern arises for a given cohort, an internal Data Review Committee (internal DRC; comprised of Sponsor staff separate from the Clinical Study Team) may be asked to review the unblinded clinical data. Details of the internal DRC decision-making process will be outlined in the DRC charter.

The computer generated randomization schedule will be prepared by J&JPRD prior to the study using randomized permuted blocks. A subject

who withdraws from the study may be replaced at the discretion of the Sponsor.

Based on the randomization codes, the unblinded pharmacist or properly trained designee will prepare the study drug for the oral administration.

Only the pharmacist or properly trained designee will be unblinded to the randomization codes. With the exception of the above, the individual subject randomization schedule will not be revealed to study subjects, investigators and clinical staff, or the J&JPRD study team until all subjects have completed the double-blind phase of the study and the database has been finalized by J&JPRD. The investigator will receive a sealed envelope for each subject containing information on the study drug identification. These sealed envelopes will be kept together, in a limited access area that is accessible 24 hours per day. Under normal circumstances, the blind should not be broken. The blind should be broken only if specific emergency treatment would be dictated by knowing the treatment status of the subject, or upon written agreement with the J&J PRD safety physician or designee. In such cases, the investigator must contact the sponsor. If the investigator is unable to contact the sponsor, the investigator may in an emergency determine the identity of the treatment by opening the sealed envelope. The sponsor must be informed as soon as possible. The date, time and reason for the unblinding must be documented on the appropriate page of the case report form (CRF) and in the source document. At the completion of dosing for each cohort, the study team may unblind the data on a cohort-by-cohort basis in order to evaluate the complete information to aid in decision making regarding future dosing. Unblinding at the completion of each cohort is warranted since the subjects are no longer participating in the study and a fully informed decision can be made regarding subsequent study conduct.

All randomization codes, whether opened or sealed, will be collected at the end of the study.

A second set of sealed randomization envelopes will be kept at the sponsor site in a safe and limited access area.

6. DOSAGE AND ADMINISTRATION

Subjects will be admitted to the CRU on Day –3. Following an overnight fast for at least 10 hours subjects will be administered a placebo dose on Days –2

and –1 and JNJ-28431754 or placebo on Day 1 and Days 3 to 16. The study medication will be administered in the sitting position between 8:00 to 9:00 am. The exact dosing time must be recorded in the CRFs. Study drug will be administered as a single oral dose via an oral liquid dispenser directly into subject's mouth by the investigator or the designated study personnel. Subjects will drink 240 mL of water immediately after dosing. Within 10 minutes after the study drug administration, subjects will receive a standardized breakfast. Standardized lunch and dinner will be provided at 4.5 and 10.5 hours, respectively, post-dose. Each meal should be completely finished within 30 minutes. Subjects will be encouraged to complete their meals. Incompletion of a meal should be recorded in the eCRFs. The three meals served on Days -1, 1 and 16 will be of the exact same composition on all days for all subjects. Water will be allowed ad libitum throughout the study. All fluid intake (i.e., volume), including that taken during meal times, will be recorded in the CRFs from Day-2 through discharge from the CRU on Day 20. Subjects will receive standardized meals in the CRU starting Day –3 through Day 20. Detailed information on the standardized meals is provided in Attachment 8. Mealtime (start and end) in the CRU, starting from admission through Day 20, will be recorded in the CRF.

7. COMPLIANCE

The investigator or designated study personnel will maintain a log of all study drug dispensed and returned. Drug supplies will be inventoried and accounted throughout the trial. The investigator or designee will supervise administration of all study medication. The exact time of each drug administration for each subject will be recorded in the CRF.

8. CONCOMITANT THERAPY

The subjects are not allowed to take any prescription or non-prescription over-the-counter (including non-steroidal anti-inflammatory drugs) or herbal medications (including herbal tea), vitamins or mineral supplements or organic/nutritional supplements within 14 days prior to study drug administration and for the duration of the study. Exceptions to this will be, as specified in Attachments 8 and 9: 1) stable doses of once daily multivitamins if approved by the PI, 2) stable doses (at least 3 months) of anti-hypertensive agents, 3) drugs for treating dyslipidemia, 4) low dose aspirin (≤100 mg/day) and 5) occasional intake of paracetamol or acetaminophen (maximum 1 g every 6 hours; 4 g/day), which is permitted periodically throughout the study

if approved by the Principal Investigator. Need for excessive or continued use of paracetamol or acetaminophen could lead to exclusion from the trial.

If the administration of any concomitant therapy becomes necessary (including allowed concomitant therapy), it must be reported in the appropriate section of the CRF. The sponsor must be notified in advance (or as soon as possible thereafter) of any instances in which prohibited therapies are administered

9. STUDY EVALUATIONS

9.1. Study Procedures

Study procedures must be performed according to this protocol. In the case of multiple assessments at a given time point the sequence of events should be: adverse event questioning, 12-lead ECG, blood pressure and pulse rate, body temperature, blood sampling for PK, PD, and clinical laboratory testing. All procedures should be initiated early enough to ensure that the PK and PD blood samples are collected at the protocol-specified times.

9.1.1. Overview

The Time and Events Schedule included in the Synopsis summarizes the frequency and timing of safety, tolerability, PK, PD, or other measurements. Venous blood will be collected for all blood-based analysis. The exact dates and times of blood drawn and study drug administration must be recorded in the CRF. Attachments 1 through 6 provide further information regarding handling of biological samples.

Adverse events and concomitant medications must be recorded on a continuous basis, starting with signing of consent and ending with the last study procedure (Follow-up). During the treatment phase study staff will periodically ask the subjects “how are you feeling?” at the time points specified in the Time and Events Schedule. In the event subjects develop symptoms of hypoglycemia, blood glucose will be assessed with a glucose analyzer and the suggested treatments listed in Attachment 7 are to be implemented as considered appropriate by the investigator. If fasting blood glucose levels are confirmed to be above 13.3 mM (240 mg/dL) or confirmed postprandial glucose levels above 19.4 mM (350 mg/dL) after lunch, the subject will be discontinued from study medication and treated with rescue medication.

The total volume of blood drawn for laboratory evaluations throughout this study is approximately 475 mL for each subject and 511 mL for those subjects participating in twice-daily dosing cohorts. A 10 mL blood sample will be collected from subjects who consent to pharmacogenomic research; this will be taken on Day 1 following study drug administration. In the event of DNA extraction failure, a replacement pharmacogenomic blood sample may be requested from the subject. Signed informed consent will be required to obtain a replacement sample. All blood samples will be collected by individual venipuncture. An indwelling catheter (kept open with a low-rate infusion of 0.9% saline) may be allowed on intensive blood sampling days.

**Parameter**	Vol. Per Sample (mL)	Total # of Samples	Total Volume (mL)
Serology	2	1	2
Endocrinology	2	3	6
Clinical Biochemistry	4	13	52
-serum creatinine	1	1	1
Hematology	2	13	26
Coagulation	2	2	4
Bone markers	1	13	13
Pharmacokinetics	3	38	114
-twice daily cohort	3	12	36
Pharmacodynamics			
-glucose, insulin, C-peptide	2	101	202
-GLP-1 active and total	3	15	45
Pharmacogenomics	10	1	10
**Total Blood Volume**			**475**
**-**twice daily cohort			**511**

9.1.2. Screening Phase

Subjects will report to the CRU for eligibility screening within 35 days prior to Day -3. Before any study specific procedures are conducted the subjects must read, understand (as judged by the Principal Investigator or designee) and sign a written Informed Consent Form. Adverse events and concomitant medication recording will start at the signing of informed consent and continue until the last study related procedure.

The following will be performed:

- Informed consent process
- Complete separate informed consent form for the optional pharmacogenomic research component of the study.
- Review inclusion/exclusion criteria
- Obtain relevant medical history and demography
- Complete physical examination including a thorough whole body skin assessment by a physician
- Measure height and body weight to determine BMI and BSA
- Measure supine vital signs (after 5 minutes in a supine position) including blood pressure and pulse rate.
- Body temperature
- 12-lead ECG
- Clinical laboratory tests including hematology, clinical chemistry, coagulation and urinary analysis using both dipstick and microscopic assessments. [Note: (1) subjects must have an overnight fast of at least 8 hours, (2) GFR calculated using the MDRD equation defined in Section 9.5.2. for details of the listed tests]
- Collect blood samples for PTH and 1,25 dihydroxy Vitamin D
- Measure serum thyrotropin (TSH)
- Collect blood samples for HBsAg, anti-HCV antibody, HIV 1 and 2 antibodies
- Collect β-HCG pregnancy test for all females
- Collect FSH and estradiol blood samples in postmenopausal females who have had no menses for 6 to 18 months prior to study start
- Urinary drug abuse and breath alcohol test

- Record adverse events from signing of informed consent and concomitant medication usage

9.1.3. Predosing Phase*

Subjects who have successfully completed the screening assessments, who meet all inclusion criteria and do not demonstrate any exclusion criteria will be instructed to withdraw their existing anti-diabetic medications 16 days prior to dosing on Day 1, then asked to return to the CRU for admission on Day –3.

Day -17 to Day -3

Candidate subjects for this study will return for an outpatient visit on Day -17 for the following instruction.

- Discontinue use of existing anti-diabetic medication on Day -16
- Subjects will be counseled by a registered dietician designated by the investigators to follow a weight maintaining diet for 2 weeks prior to study admission. In addition, the study subjects will discuss about the status of the weight maintaining diet with the dietician by telephone on Day –9. The weight maintaining diet will follow standard recommendations of the American Diabetes Association, comprising ~55-60% carbohydrate, 10-20% protein, and ~30% fat, with total daily caloric intake individually adjusted for BMI.
- Subjects will be provided with glucose analyzers and instructed to monitor their blood glucose at least once each morning (fasting sample), and post lunch. The results will be recorded on a diary card and reported to the study center on Day -9 for entry into the eCRF. The subject will be instructed to report any signs or symptoms of hypoglycemia, fasting blood glucose values > 13.3 mM (240 mg/dL), post lunch values > 19.4 mM (350 mg/dL) and blood glucose values < 2.8 mM (50 mg/dL) to the study center. Subjects will be instructed to resume taking their previous oral anti-diabetic agent at any time during the washout period if confirmed fasting blood glucose levels rise above 13.3 mM (240 mg/dL) or confirmed postprandial glucose levels rise above 19.4 mM (350 mg/dL) following the lunch. Subjects will discontinue from the study after they safely resume their previous anti-diabetic medication at the discretion of the investigator.
- Collect FSH and estradiol blood samples in postmenopausal females who have had no menses for 6 to 18 months prior to study start

* This section of the protocol has been revised. Please refer to the section of this document titled PROTOCOL AMENDMENTS (Amendment DEU-5, 27 September 2007) for a detailed description of the specific changes.

Day –3 to Day –1.

Subjects will be admitted to the study center on Day –3. After admission, inclusion/exclusion criteria, prohibitions/restrictions and diary cards will be reviewed. Eligible subjects will remain in the clinic until discharge on Day 20.

Subjects must fast overnight for 10 hours prior to the anticipated time of placebo dosing on Days –2 and –1 (between 8:00 to 9:00 AM) in a single blind fashion (subjects will be blinded)

The exact times for each blood draw or assessment will be recorded in the CRF.

The following assessments will be performed on Day –2:

- Physical examination including a thorough whole body skin assessment by a physician
- Body temperature
- Orthostatic vital signs at 4 and 8 hours post dose
- Urinary drug abuse, serum pregnancy and breath alcohol test (-2 day window allowed)
- Fasting clinical laboratory profiles: hematology, urinalysis and biochemistry (see Section 9.5.2. for details)
- Collect FSH and estradiol blood samples in postmenopausal females who have had no menses for 6 to 18 months prior to study start (-2 day window allowed)
- Urine NTX and urine deoxypyridinolines (DPD), serum bone specific alkaline phosphatase and serum osteocalcin (OC)
- Collect blood samples for PTH and 1,25 dihydroxy Vitamin D
- Administration of visual analogue scale (VAS) questionnaires to assess appetite and satiety at 5 minutes prior to breakfast, 30, 60, 90, 120, 150, 180, 210 and 240 minutes after starting breakfast, 5 minutes prior to lunch and immediately after lunch on Day –2 (Each subject will complete his/her VAS assessment in a separate area and will not be allowed to talk to others or exchange messages with others. (Attachment X)
- A four-item patient reported outcomes questionnaire will be administered upon rising in the morning (Attachment 12). (Each subject will complete his/her outcome questionnaire in a separate area and will not be allowed to communicate or exchange messages with others.

- Record 24 hour fluid intake and urine output volume. The times and volumes of fluid intake or urine output volumes should be recorded in the eCRFs.
- Frequency of urination over each 24 hours should also be recorded
- Record adverse events and concomitant medication usage

Following completion of the predose assessments, a dose of placebo will be administered in the sitting position between 08:00 to 09:00 AM in a single blind fashion (subjects will be blinded), and the exact time of dosing will be recorded in the CRF. Subjects will receive standardized breakfast starting within 10 minutes postdose, lunch and dinner at 4.5, 10.5 hours, respectively, post morning study drug administration. Water will be allowed ad libitum throughout Days –2 to 20. Fluid intake including those taken during each meal, urine volume and frequency over each 24-hour period will be recorded in the eCRFs.

The following assessments will be performed on Day –1:

- Fasting serum creatinine
- Whole body skin assessment by a physician
- Vital signs (blood pressure and heart rate, supine) and 12-lead ECGs will be performed at T0, T2, T6 and T12 where T corresponds the dosing time on dosing days. Vital signs and 12-lead ECGs will be measured in triplicates in 2 minutes apart. Triplicate recordings, each 2 minutes apart, at each predefined point. The average of the triplicate measurements at each time point on Day –1 will serve each subject's time-matched baseline value for the corresponding parameters on Days 1 to 20. All morning vital signs and 12-lead ECGs will be performed prior to breakfast.
- Continuous Lead II ECG monitoring (telemetry) will be conducted from 30 minutes prior to till 8 hours post dosing.
- Urine samples will be collected on Day –1 at T0–T2, T2–T4.5, T4.5–T7, T7-10.5, T10.5-13, and T13-24, where T0 corresponds to the dosing time on dosing days. Urine volume of each collection interval will be recorded in the eCRFs. Two 10 ml aliquots of urine samples will be taken from from each collection interval. One will be used for back up. The other will be used for measurements of the following:
    - glucose
    - creatinine
    - electrolytes: sodium, potassium, chloride, phosphate, calcium and magnesium

  - pH, specific gravity and osmolality
  - albumin
  - N-acetyl-beta-glucosaminidase, and beta-2-microglobulin
- 24-hour plasma glucose, insulin and C-peptide levels will be collected at T-0.5, T –0.25, T0 (predose), T0.5, T1, T1.25, T1.5, T2.0, T2.5, T3.0, T4.5 (prior to lunch), T5.0, T5.5, T6.0, T6.5, T7.0, T8.0, T9.0, T10.5 (prior to dinner), T11, T12.0, T12.5, T13.0, T14.0 T16.0, T19.0, T22.0, and T24 hours where T0 corresponds the dosing time (hours) on each dosing days
- Blood samples for GLP-1 active and total will be collected on Day -1 predose at -0.5 and 0.5, 1, 1.5 and 2.0 postdose
- Urine NTX and urine deoxypyridinolines (DPD), serum bone specific alkaline phosphatase and serum osteocalcin (OC)
- Body weight will be assessed using a calibrated scale prior to breakfast
- Record 24 hour fluid intake and frequency of urination

9.1.4. Double-Blind Dosing Phase*

Days 1-22

Following completion of the Day –2 and –1 safety and PD baseline assessments, subjects will be randomized to receive JNJ-28431754 or placebo in a double-blind fashion between 08:00 to 09:00 AM on Day 1 and Days 3 through 16, and the exact time of dosing will be recorded in the CRF. No study drug will be administered on Day 2. Subjects will receive standardized breakfast within 10 minutes postdose, lunch and dinner at 4.5 and 10.5 hours, respectively, post morning study drug administration. Water will be allowed ad libitum. Fluid intake including those taken during each meal and urine volume and urine frequency over each 24-hour period will be recorded in the eCRFs.

The following assessments will be performed on Days 1-16. The exact times for each blood draw or assessment will be recorded in the CRF. Where assessments occur at the same time point, the blood sample for pharmacokinetic analysis must always be taken as close to the scheduled time as possible. For pharmacokinetic timepoints up to and including the 4-hour sample a window of ± 5 minutes is allowed and for postdose time

* This section of the protocol has been revised. Please refer to the section of this document titled PROTOCOL AMENDMENTS (Amendment DEU-5, 27 September 2007) for a detailed description of the specific changes.

points greater than the 8-hour sample a window of ±15 minutes is allowed. The order of multiple assessments within 1 protocol time point should also be the same throughout the study.

- Plasma samples for measurement of JNJ-28431754 and its metabolite concentrations will be collected for measurement of JNJ-28431754 concentration at predose, 0.5, 1, 1.5, 2, 2.5, 3, 4, 6, 8, 10, 12, 24 and 48 hours postdose on Days 1 and 16, and at pre-dose on Days 3, 5, 7, 9, 11, 13 and 15 and at 72, 96, 120 and 144 hours postdose on Day 16.
- Urine samples will be collected at predose, 0-2, 2-4.5, 4.5-7, 7-10.5, 10.5-13, 13-24 and 24-48 post dose on Day 1 and 0-2, 2-4.5, 4.5-7, 7-10.5, 10.5-13, 13-24, 24-48, 48-72 and 72-96 on Day 16. Three aliquots (10 mL each) will be taken from each urine collection fractions. One aliquot will be stored at -70˚ C for potential future determination of additional analytes. One will be used for measurement of JNJ-28431754. Another will be used to determine the following:
    - glucose
    - creatinine
    - electrolytes: sodium, chloride, potassium, phosphate, calcium and magnesium
    - pH, specific gravity and osmolality
    - albumin
    - N-acetyl-beta-glucosaminidase, and beta-2-microglobulin
- 0-24 hour urine samples will be collected on Days 3 to 15. For each urine collection, the above urinary assessments will be conducted. Urine volume collected in each time intervals will be recorded and aliquots taken after mixing for storage at -20°C and subsequent assay.
- 24-hour plasma glucose, insulin and C-peptide levels will be collected at – 0.5, -0.25, 0 (predose), 0.5, 1, 1.25, 1.5, 2.0, 2.5, 3.0, 4.5 (prior to lunch), 5.0, 5.5, 6.0, 6.5, 7.0, 8.0, 9.0, 10.5 (prior to dinner), 11, 12.0, 12.5, 13.0, 14.0 16.0, 19.0, 22.0, and 24 hours on Days 1 and 16
- Blood samples for GLP-1 active and total will be collected on Days 1 and 16 at -0.5 predose and 0.5, 1, 1.5 and 2.0 hours postdose
- Morning fasting plasma glucose, insulin and C-peptide on Days 3 to 22
- Administration of visual analogue scale (VAS) questionnaires to assess appetite and satiety at 5 minutes prior to breakfast, 30, 60, 90, 120, 150, 180, 210 and 240 minutes after starting breakfast, 5 minutes prior to lunch and immediately after lunch on Days 3, 7, 11 and Day 15. (Each subject will complete his/her VAS assessment in a separate area and will not be

allowed to talk to others or exchange messages with others. (Attachment 11)

- Morning fasting body weight will be assessed on Days 1 through 20 using a calibrated scale.
- Physical examinations will be performed on Days 15 and 20
- Body temperature will be measured in the morning at Days 1 to 20
- Whole body skin assessments on Days 1 to 20 by a physician
- Vital signs (blood pressure and heart rate, supine) and 12-lead ECGs will be performed at 0 (predose), 2, 6, 12, 24, 48, 72 and 96 hours post dose on Days 1 and 16; at 2, 6 and 12 hours on Days 3 to 15. Vital signs and 12-lead ECGs will be measured in triplicate 2 minutes apart. Triplicate recordings, each 2 minutes apart, at each predefined point. The average of the triplicate measurements at each time point on Day –1 will serve each subject's time-matched baseline value for the corresponding parameters on Days 1 to 20. All morning vital signs and 12-lead ECGs will be performed prior to dosing on study drug administration days and prior to breakfast on non-dosing days.
- Continuous Lead II ECG monitoring (telemetry) will be conducted from 30 minutes prior to till 8 hours post dosing on Day 1, 6, 8 and 15.
- Orthostatic vital signs (blood pressure and heart rate) will be measured after 3 minutes standing following the supine vital sign measurements at 4 and 8 hours post dose or corresponding to dosing time on Days 1 to 20.
- Adverse events will be recorded during the whole study and will be specifically queried on each dosing day at predose, at 3, 6 and 12 hours post dose, on the mornings of Days 2, 20, 21, 22 and at the follow up visit.
- Fasting samples for hematology, clinical chemistry and urinalysis will be collected on Days 2, 3, 5, 8, 11, 14, 17, 18, 20, and 22.
- A coagulation sample will be collected on Day 20.
- Urine NTX and urine deoxypyridinolines (DPD), serum bone specific alkaline phosphatase and serum osteocalcin (OC) will be assessed on Days 2, 3, 5, 8, 11, 14, 17, 18 and 20.
- Collect blood samples for PTH and 1,25 dihydroxy Vitamin D on Days 5, 11, and 17.
- A four-item patient reported outcomes questionnaire will be administered on Days 4, 7 and 14 to each subject upon rising in the morning (Attachment 12). (Each subject will complete his/her assessment in a separate area and will not be allowed to talk to others or exchange messages with others. (Attachment 12)
- All fluid intake, urine volume and frequency of urination over each 24 hour period will be recorded in the eCRFs throughout Days 1 to 19

- A 10 mL blood sample will be collected from subjects who gave separate informed consent for the pharmacogenomic component of the study, on Day 1.
- Concomitant medications will be recorded throughout the study.
- Real time blood glucose concentrations will be determined prior to each standardized breakfast and 2 hours post lunch using a glucose analyzer throughout Days 1 to 20. On Days -1, 1 and 16 the measurements will be performed prior to breakfast and 2 hours post each meal. Investigational staff will monitor subjects for symptoms possibly indicative of hypoglycemia throughout the in-clinic residence. Blood glucose will be immediately measured using a glucose analyzer should hypoglycemia symptoms appear and confirmed by conventional venous sampling. Attachment 7 provides the clinical signs, symptoms, grading scale, and treatment for hypoglycemia. Should hypoglycemia occur it should be entered in the eCRF adverse event page (see Attachment 7).

On Day 20 following completion of all postdose assessments, subjects may be discharged, unless there are clinically significant adverse events in which case the subject will be asked to remain in the clinic. When discharged, subjects will be instructed that they have to follow the restrictions as outlined in Section 4.4 (Prohibitions and Restrictions).

- Subjects will be given a contact number for the on-call physician at the clinic that can be called 24 hours a day 7 days a week in case an adverse event occurs. Subjects will be instructed to return to the clinic daily in the morning on Days 21 to 22. They will also return for a Follow-up visit 7 to 10 days post the Day 22 visit.

9.1.5. Outpatient Phase

Days 21 to 22

Assessments should be performed relative to the previous study drug administration time.

- Plasma samples for measurement of JNJ-28431754 and its metabolites at 120 (Day 21) and 144 hours (Day 22) post dose
- Morning fasting plasma glucose, insulin and C-peptide concentrations will be obtained on Days 21 and 22.
- Adverse events will be recorded during the whole study and will be specifically queried on the mornings of Days 21 and 22.
- Fasting samples for hematology, clinical chemistry and urinalysis will be collected on Day 22

- Urine NTX and urine deoxypyridinolines (DPD), serum bone specific alkaline phosphatase and serum osteocalcin (OC) will be assessed on Day 22.
- Collect blood samples for PTH and 1,25 dihydroxy Vitamin D on Day 22.
- Concomitant medications will be recorded throughout the study.

Subjects will be instructed to resume taking their previous oral anti-diabetic agent on Day 22, or at any time during the study if confirmed fasting blood glucose levels rise above 13.3 mM (240 mg/dL) or confirmed postprandial glucose levels rise above 19.4 mM (350 mg/dL) following lunch. Instruction will also include reinforcement of signs and symptoms of hypoglycemia. Subjects will be instructed to monitor their blood glucose via fingerstick using a glucose meter at least once each morning (fasting sample) and post lunch. The results will be recorded on a diary card and reported to the study center for entry into the eCRF. The subject will be instructed to report any signs or symptoms of hypoglycemia, fasting blood glucose values > 13.3 mM (240 mg/dL), post lunch values > 19.4 mM (350 mg/dL) and blood glucose values < 2.5 mM to the study center. For the safety of the study subjects, they will remain in the clinic for approximately 6 hours after re-starting their anti-diabetic medications.

9.1.6. Posttreatment Phase (Follow-Up)

Subjects will return to the clinical study center 7 to 10 days following the Day 22 visit for a Follow-up visit. The following assessments will be performed:

- Physical examination including thorough whole body skin assessment
- Vital signs (blood pressure and heart rate) and 12-lead ECG
- Body temperature
- Morning fasting body weight
- Adverse events will be recorded during the entire study and will be specifically queried during the follow-up visit
- Fasting samples for hematology, clinical chemistry and urinalysis dipstick
- Urine NTX and urine deoxypyridinolines (DPD), serum bone specific alkaline phosphatase and serum osteocalcin (OC) will be assessed.
- Collect blood samples for PTH and 1,25 dihydroxy Vitamin D

9.2. Pharmacokinetic Evaluations

9.2.1. Sample Collection and Handling

9.2.1.1. Plasma:

Day 1:

Venous blood samples (3 mL) for measurement of JNJ-28431754 and its metabolites concentration will be collected at predose, 0.5, 1, 1.5, 2, 2.5, 3, 4, 6, 8, 10, 12, 24 and 48 hours postdose (total 14 samples). If a twice-daily dosing regimen is administered, 6 additional PK plasma samples will be collected at 10.5 (prior to dinner), 13, 14, 16, 19 and 22 hours post the morning dose.

Days 3 to 15:

Venous blood samples (3 mL) will be collected at pre-dose in the morning on Days 5, 7, 9, 11, 13 and 15 to obtain trough JNJ-28431754 and its metabolites concentrations

Day 16:

Venous blood samples (3 mL) for measurement of JNJ-28431754 and its metabolites concentration will be collected at predose, 0.5, 1, 1.5, 2, 2.5, 3, 4, 6, 8, 10, 12, 24, 48, 72, 96, 120 and 144 hours postdose (total 18 samples). If a twice-daily dosing regimen is administered, 6 additional PK plasma samples will be collected at 10.5 (prior to dinner), 13, 14, 16, 19 and 22 hours post the morning dose.

Collection times may be modified as pharmacokinetic data become available during the study, but total blood volume collection for each subject for all analysis (PK, PD, safety) will not exceed 550 mL over 8 weeks in this study.

Plasma and urine samples will be stored following analysis for potential identification/analysis of further JNJ-28431754 metabolites or concomitant medication pharmacokinetic analysis and will be reported separately.

The exact dates and times of blood sampling must be recorded in the CRF. Blood samples will be collected from an intravenous cannula or by direct venipuncture if required or if the cannula is not functioning. If blood samples are collected via an indwelling cannula, the following procedure will be used. The saline infusion will be stopped and 2 mL of blood drawn into a syringe attached to a stopcock. The required volume of blood is drawn into

a second syringe according to instructions provided by the laboratory. The 2 mL of blood and saline in the first syringe will be reinjected and the slow saline infusion continued.

Refer to Attachments 1, 2 and 3 for further information regarding handling, shipment and labeling of biological samples.

9.2.1.2. Urine

Day 1:

Urine samples for analysis of JNJ-28431754 and its metabolites will be collected at predose, 0-2, 2-4.5 (prior to lunch), 4.5-7, 7-10.5 (prior to dinner), 10.5-13, 13-24 and 24-48 hours post dose.

Day 16:

Urine samples for analysis of JNJ-28431754 and its metabolites concentration will be collected at 0-2, 2-4.5 (prior to lunch), 4.5-7, 7-10.5 (prior to dinner), 10.5-13, 13-24, 24-48, 48-72 and 72-96 hours post dose.

Subjects will be instructed to follow the standard urine collection procedure. Subjects will void their bladders just before drug administration. To ensure a complete urine collection during each collection interval, subjects should completely empty their bladders at the end of each interval. The urine collection time (start/end) and the total collected volume (or weight) during each collection interval must be recorded on CRF. Samples of each collection interval need to be well mixed, within 1 hour after each interval, and before sampling the aliquot for the PK, PD, and Safety Lab analysis. Two 10 mL aliquots of each collection interval will be stored at -20°C for the measurement of JNJ-28431754 and its metabolites levels in the urine. An aliquot (5 mL) will be taken from the 24-hour urine collection and stored at -70˚ C for potential future determination of additional analytes. Aliquots will also be taken for assay of urine PD and safety samples (See Sections 9.3 and 9.5). Attachments 1-3 provide further information regarding sample handling, labeling, and shipment.

9.2.2. Analytical Procedures

Pharmacokinetic plasma and urine samples will be analyzed for concentrations of JNJ-28431754 using validated, selective and sensitive

liquid chromatography-mass spectrometry (LC-MS/MS) methods under the responsibility of the Bioanalytical Group at J&JPRD

After quantification of JNJ-28431754, the remaining plasma and urine samples will be stored at -20°C for future metabolite profiling and/or metabolite quantification as deemed necessary by the sponsor (to be reported separately from this study).

Samples collected from the placebo subjects will not be analyzed except 2 samples: one predose and the other around the expected t_{max}. This is to ensure from a safety perspective that no placebo subject could have been incorrectly dosed with JNJ-28431754.

9.2.3. Pharmacokinetic Parameters

9.2.3.1. Plasma

Based on the individual plasma concentration-time data, using the actual sampling times, the following pharmacokinetic parameters of JNJ-28431754 and its metabolites will be estimated:

Day 1

C_{max}	peak plasma concentration
t_{max}	time to reach the peak plasma concentration
CL/F	Apparent systemic clearance following oral administration, calculated as: D/AUC_{τ} ; for the parent compound, JNJ-28630368, only
AUC_{24hr}	area under the curve from 0 to 24 hours post dosing, calculated by trapezoidal summation
AUC_{∞}	AUC_t extrapolated to infinity, calculated as $AUC_t + C_{last}/\lambda_z$
λ_z	elimination rate constant, determined by linear regression of the terminal points of the ln-linear plasma concentration-time curve
$t_{½}$	terminal half-life, defined as $0.693/\lambda_z$

Day 3-15

C_{trough}trough plasma concentration prior to dosing or at the end of the dosing interval of any dose other than the first dose

Day 16

$C_{max, ss}$ maximum plasma concentration during a dosing interval at steady state

$AUC_{\tau,ss}$ area under the plasma concentration-time curve during a dosing interval (τ) at steady-state

CL/F Apparent systemic clearance following oral administration, calculated as: D/AUC_{τ} ; for the parent compound, JNJ-28630368, only

Vd_{ss}/F apparent volume of distribution at steady-state following oral administration, calculated as MRT*CL; for the parent compound, JNJ-28630368, only.

FI fluctuation index, i.e., percentage fluctuation (variation between peak and trough at steady-state), calculated as $100*[(C_{max, ss} - C_{min, ss})/C_{avg,ss}]$

Acc Ratio accumulation index, calculated by using the following equation: $Acc\,Ratio = \frac{AUC_{\tau,ss}}{AUC_{\tau,sd}}$

where $AUC_{\tau,ss}$ and $AUC_{\tau,sd}$ are the AUCs over the dosing interval (τ) at steady-state (ss) and following a single dose (sd), respectively.

$t_{½}$ terminal half-life, defined as $0.693/\lambda_z$, where λ_z is the elimination rate constant

Additional plasma PK parameters may be determined as appropriate.

9.2.3.2. Urine

Based on the urinary excretion data (Days 1 and 16), the following parameters will be calculated, as appropriate for JNJ–28431754

Aet	total urinary recovery of unchanged drug over interval 0-t hours
CLR	renal clearance calculated as the cumulative amount excreted in urine (Aet) divided by the AUCt
Ae (% dose)	percentage of the dose excreted in the urine unchanged calculated as (Ae/Dose)*100

Additional urine PK parameters may be determined as appropriate.

9.3. Pharmacodynamic Evaluations

9.3.1. Primary

1. Change from baseline mean 24-hour plasma glucose concentration on Days 1 and 16, which is defined as the area under the plasma glucose time concentration time curve at 0-24 hours divided by 24 hours (Glucose AUC 0-24/24h). Glucose AUC 0-24/24h determined on Day –1 will be used as the baseline values.
2. Change from baseline urine glucose excretion (UGE)
   a. Cumulative amount over each 24 hours on Days 1, 2, 8, 12, 16, 17, 18, 19; cumulative amount over 24 hours on Day –1 will be used a the baseline value.
   b. UGE rate, which is defined as the UGE amount in each fraction divided by hours of each collection interval on Days 1, 2, 8, 12, 16, 17, 18, 19

9.3.2. Secondary:

1. Renal threshold (R_T), which is defined as the plasma glucose level at which maximum urinary glucose reabsorption is achieved and above which glucose is excreted in the urine will be determined on Days -1, 1 and 16. The R_T is calculated as a function of time for each subject based on measured plasma glucose, urinary glucose excretion, and glomerular filtration rate (GFR)

   The equations used to calculate R_T between times t1 and t2 are

   $$GFR\int_{t_1}^{t_2} f\left(G, R_t\right)dt = UGE(t_1, t_2)\ ;\ (1)$$

   $$f(G, R_T) = \begin{cases} G - R_T & \text{if } G > R_T \\ 0 & \text{if } G < R_T \end{cases} \quad (2)$$

   Where G is the plasma glucose concentration, UGE (t1,t2) is the amount of glucose excreted in the urine between times t1 and t2.

During each time interval that UGE is collected, the integral is calculated using the trapezoidal method and the mean value of R_T over this interval is calculated

2. Change from baseline mean 24-hour insulin concentration on Days 1 and 16, which is defined as the area under the plasma insulin time concentration time curve at 0-24 hours divided by 24 hours (Insulin AUC 0-24/24h). Insulin AUC 0-24/24h determined on Day –1 will be used as the baseline values.
3. Changes from baseline morning fasting plasma glucose (FPG) and insulin (FPI) on Days 1 and 17. FPG and FPI values determined on Day –1 will be used as the baseline value. FPG and FPI on Days –1, 1 and 16 will be calculated by averaging predose values at –30, -15 and 0 minutes. On Days 2 to 15 and 17 to 20, one FPG and FPI will be determined within 30 minutes prior to dosing on dosing days or prior to breakfast on non-dosing days.
4. Change from baseline glucose and insulin excursions: after each breakfast, defined as the difference between the maximum glucose and insulin values observed during the 4-h post meal period minus the mean of the premeal measurements at –30, -15 and 0 min on Days 1 and 16. Glucose and insulin excursion on Day –1 will be used as the baseline value.
5. Glucose AUC 0-2 on Days 1, and 16; Glucose AUC 0-2 on Days –1 will be used as the baseline value.
6. Insulin sensitivity (IS) will be calculated based on plasma glucose and insulin concentration time curves post breakfast on Day –1, 1 and 16 using a robust algebraic method, namely $S_I AR_a A$.[13]
7. Beta-cell function

   Two measures of beta-cell function will be calculated from the glucose and insulin and C-peptide curves post breakfast on Days –1, 1 and 16.

   Insulinogenic index, a measure of early insulin release and is $= \dfrac{I(30) - I(0)}{G(30) - G(0)}$

   Insulin secretion rate (ISR), ISR will be calculated based on measured C-peptide and glucose concentrations using a modified method of Mari et al.[14]
8. Active and total glucagon-like peptide-1 (GLP-1) levels on Days -1, 1 and 16 at -0.5 predose and 0.5, 1, 1.5 and 2.0 postdose
9. Assessment of Visual Analogue Scale (VAS) questionnaires to assess appetite and satiety at 5 minutes prior to breakfast, 30, 60, 90, 120, 150, 180, 210 and 240 minutes after starting breakfast, 5 minutes prior to lunch and immediately after lunch on Days –2, 3, 7, 11 and

Day 15. The VAS questionnaire schedule may be modified if a twice-daily dosing regimen is tested. (Each subject will complete his/her VAS assessment in a separate area and will not be allowed to talk to others or exchange messages with others. (Attachment 11)

10. Morning fasting body weight will be assessed at Screening and on Days -1 through 20 and at the Follow-up visit using a calibrated scale.

9.4. Pharmacogenomics

A 10 mL blood sample will be collected from subjects who consent to the pharmacogenomic component of the study. Instructions on sample handling, labeling and shipment are provided in Attachment 4. There are two parts to the pharmacogenomic component of this study:

9.4.1. Analyses Related to the Trial (Part A)

This part of pharmacogenomic research allows for the analysis of genes that may be relevant to JNJ-28431754 or Type 2 diabetes mellitus. Candidate genes will only be genotyped, as necessary, if it is hypothesized that this may help resolve issues with the clinical data. The genes that are currently hypothesized to potentially be relevant to JNJ-28431754 or Type 2 diabetes mellitus are provided below. Genotyping of any of the candidate genes listed below would be performed on identifiable samples.

ADME related genes: *ABCB1, ABCB4, ABCC1, ABCC2, ADH gene family, AHR, ALDH gene family, ARNT, CYP1A1, CYP1A2, CYP1B1, CYP2A6, CYP2B6, CYP2C19, CYP2C8, CYP2C9, CYP2D6, CYP2E1, CYP3A4, CYP3A5, CYP4B1, EPHX1, EPHX2, FMO1, FMO2, FMO3, FMO4, GSTM1, GSTP2, GSTT1, MPO, NAT1, NAT2, NFE2L2, NR1I2, SULT1A1, SULT1A2, SULT2A1, TPMT, UGT1A1, UGT1A3, UGT1A4, UGT1A6, UGT1A7, UGT1A8, UGT1A9, UGT2B15, UGT2B4, UGT2B7, SLC22A1-5 (OCT gene family), SLC22A6-8 and SLC22A6-11 (OAT gene family), SLC21A3, SLC21A9, SLC21A11 and SLC21A12 (OATP gene family).*

Target related genes: *SGLT gene family (SLC5 family), GLUT gene family (SLC2 family).*

Diabetic related genes: ABCA1, ABCC8, ACE, ACP1, ACTN4, ADA, ADIPOQ, ADRB3, AKR1B1, ALB, ALMS1, ANGPTL4, APOA1, APOA4, APOB, APOC3, APOE, ARG1, ASIP, BBS4, BCHE, CAPN10, CART, CCK, CCKAR, CCL2, CD36, CD59, CIDEA, CPE, CTLA4, CYP19A1, DF,

DIANPH, DPP4, ENPP1, EPHX2, ESR1, FABP10, FABP2, FABP4, FABP5, FASN, FOXC2, FXN, GAD1, GAD2, GAL, GCG, GCGR, GCK, GCKR, GDF8, GH1, GH2, GHRL, GIP, GIPR, GNB3, GPD2, GPKOW, GYS1, HBA1, HFE, HK1, HK2, HK3, HLA-DQA1, HLA-DQB1, HMGA2, HNF4A, HSD11B1, HSD17B7, IAPP, IDE, IGF1, IGF1R, IKBKB, IL6, INS, INSR, IPF1, IRS1, IRS2, IRS4, ISL1, JPH3, KCNJ11, KCNJ9, LEP, LEPR, LIPC, LIPE, LMNA, LPA, LPAL2, LPL, LRPAP1, MAPK8, MAPK8IP1, MC3R, MC4R, MKKS, NEUROD1, NEUROG3, NFKB1, NOS3, NPY, NR0B2, NR3C1, NUCB2, PBX1, PCSK1, PDE3B, PGC, PGR, PLG, PLIN, POMC, PON1, PON2, PPARA, PPARD, PPARG, PPARGC1A, PPARGC1B, PPP1R1A, PPP1R3A, PRL, PTPN1, PTPRN, RBP4, REG1A, RETN, RPS6KB1, SCARB1, SERPINE1, SIM1, SLC2A1, SLC2A10, SLC2A2, SLC2A4, SORBS1, SPINK1, SREBF1, SST, TCF1, TCF2, TCF7L2, TGFB1, TH, TNF, TRAPPC2, TUB, TULP2, UCN, UCP1, UCP2, UCP3, VDR, WFS1, WRN, XBP1, and ZDHHC23.

9.4.2. DNA Storage for Future Analyses (Part B)

This part of the pharmacogenomic research allows for the storage of DNA samples for future genetic research related to JNJ-28431754 or the indications for which it is developed. Stored DNA samples and relevant clinical data will be held in a non-identifiable format, whereby the study subject identifier is replaced with a new number, thus limiting the possibility of linking genetic data to a subject's identity. Samples will be made non-identifiable after the Clinical Study Report has been issued.

Subjects will be given the option to participate in Part A, Part B, both parts, or neither part of the pharmacogenomic component of this study (where local regulations permit).

9.5. Safety Evaluations

9.5.1. Adverse Events

Spontaneously reported adverse events will be recorded for the entire duration of the study i.e., from the time a signed and dated informed consent form is obtained until completion of the last study-related procedure at the safety follow-up visit. Adverse events will also be specifically queried using non-directive questioning (e.g. study staff will ask the subjects "How are you feeling?") on Day -2 to 1 and 3 to 16 at predose and at 3, 6 and 12 hours post dose (placebo dose on Days -2 and -1), on the mornings of Days

2, 20, 21, 22 and at the follow-up visit. Specific details on adverse event reporting are provided in Section 12.

9.5.2. Clinical Laboratory

Clinical safety laboratory tests (hematology, clinical chemistry, urinary analysis using both dipstick and microscopic assessments) will be conducted at the fasting condition at screening, on Days -2, 2, 3, 5, 8, 11, 14, 17, 18, 20, and 22 and at the final follow-up visit 7 to 10 days post Day 22. Coagulation (INR and aPTT) will be assessed at screening and Day 20. TSH will be done at screening. The Principal Investigator or his/her designees must review all laboratory reports; document his reviews and record any clinically relevant changes occurring during the study in the adverse event section of the eCRF. The following tests will be performed:

Hematology Panel

Hemoglobin	Platelet count
Hematocrit	Percent reticulocytes
Red blood cell (RBC) count	HbA1c (screening only)
White blood cell (WBC) count with differential	

Clinical Chemistry Panel

Serum electrolytes: sodium, potassium, calcium, magnesium, chloride, inorganic phosphate, bicarbonates, uric acid	Aspartate aminotransferase (AST)
Serum creatinine, GFR*	Alanine aminotransferase (ALT)
Alkaline phosphatase	Gamma-glutamyltransferase (GGT)
Lactic dehydrogenase (LDH)	Total bilirubin
Albumin	serum glucose
Total protein	Total cholesterol
Blood urea nitrogen (BUN)	LDL- cholesterol
Creatine phosphokinase (CPK)	HDL- cholesterol
Serum osmolality	triglycerides
Anion gap**	

*Glomerular filtration rate (GFR) calculated using the MDRD equation defined as: GFR = 175 x (standardized Scr)$^{-1.154}$ x (age) $^{-0.203}$ x 0.742 (if the subject is female) x 1.212 (if the subject is black). GFR is expressed in milliliters per minute per 1.73 m^2 and race is either black or not.[15]

** The anion gap (AG) formula: AG = Na – (Cl + HCO^3)

Urinalysis

Dipstick	Sediment
specific gravity	RBC
pH	WBC
glucose	epithelial cells
protein	crystals
blood	casts
ketones	bacteria
bilirubin	
urobilinogen	
nitrite	
leukocyte esterase	

Renal safety assessments:

Urine samples will be collected at predose, 0-2, 2-4.5 (pre lunch), 4.5-7, 7-10.5 (pre dinner), 10.5-13 and 13-24 hours post dose on Day -1 at 0-2, 2-4.5 (pre lunch), 4.5-7, 7-10.5 (pre dinner), 10.5-13, 13-24 and 24-48 hours post dose on Day 1 and at 0-2, 2-4.5 (pre lunch), 4.5-7, 7-10.5 (pre dinner), 10.5-13, 13-24, 24-48, 48-72 and 72-96 hours post dose on Day 16. Urine samples will also be collected at 0-24 hour interval post each dose on Days 3 to 15. For each urine collection, the followings will be measured:

1. glucose
2. creatinine
3. electrolytes: sodium, potassium, phosphate, calcium, chloride and magnesium
4. pH, specific gravities and osmolality
5. albumin
6. N-acetyl-beta-glucosaminidase, and beta-2-microglobulin

Subjects will be instructed to follow the standard urine collection procedure. Subjects will void their bladders just before each drug administration. To ensure a complete urine collection during each collection interval, subjects should completely empty their bladders at the end of each interval. The

urine collection time (start/end) and the total collected volume (or weight) during each collection interval must be recorded on CRF. Samples of each collection interval need to be well mixed, within 1 hour after each interval, and before sampling the aliquot for the renal safety lab assessments. Two 10 mL aliquots of each collection interval will be stored at -20°C for the measurement of the renal safety samples.

24-hour creatinine clearance (CLcr) on Day –1, 3, 5, 8, 11, 14, 16, 17, 18 and 19

CLcr [mL/min] = ((Creatinine urine [mg/dL]) x (V urine [mL/min])/Creatinine plasma [mg/dL]) x (1.73/BSA[m2])

BSA = Body Surface Area that can be obtained from nomograms which require height in cm and weight in kg

The amount of urine creatinine in each urine samples will be added to obtain a 24-hour cumulative urine creatinine, which will be used for calculation of CLcr along with serum creatinine determined on the same day

Other

Bone turnover biomarkers

Urine NTX and urine deoxypyridinolines (DPD), serum bone specific alkaline phosphatase and serum osteocalcin (OC) will be assessed on Days –2, -1, 2, 3, 5, 8, 11, 14, 17, 18, 20, and 22 and at the Follow-up visit. PTH and 1,25 dihydroxy Vitamin D will be assessed at screening and on Days -2, 5, 11, 17, 22 and at the Follow-up visit.

Coagulation

aPTT and INR will be assessed at screening and Day 20

Serology tests

HBsAg, anti-HCV antibody, HIV 1 and 2 antibodies will be tested at screening.

Endocrinology tests

ß-HCG, Follicle stimulating hormone (FSH), estradiol in females only at screening, Day -17 and Day -2 (a -2 day window is allowed at Day -2)

Thyroid function test:

TSH will be measured at screening.

Drug/Alcohol Screen:

A drug and alcohol screen will be performed at screening and Day -2.

9.5.3. Cardiac Monitoring*

Full 12-lead ECGs will be recorded. Subjects should be rested quietly in the supine position for at least 10 minutes before the measurement is started. 12-lead ECGs will be recorded at a paper speed of 25 mm/sec so that the different ECG intervals (RR, PR, QRS, QT, QTc) can be measured. The ECG will be recorded until 4 regular consecutive complexes are available for analysis. QT uncorrected, QTcB (corrected QT interval, calculated based on Bazett's method; primary) and QTcF (corrected QT interval, calculated based on Fridericia's method; secondary) will be calculated and presented.[15-18]

12-lead ECGs will be performed at screening and at T0, T2, T6 and T12 on Day –1 where T corresponds the dosing time on dosing days. The 12-lead ECGs will be performed at 0 (predose), 2, 6, 12, 24, and 48 hours post dose on Days 1 and 16, and at 2 hours on Days 3 to 15 and once at follow-up. The 12-lead ECGs will be measured in triplicate 2 minutes apart at each predefined point except at screening and the Follow-up visit. The average of the triplicate measurements at each time point on Day –1 will serve each subject's time-matched baseline value for the corresponding parameters on Days 1 to 20. ECGs will be evaluated by the Principal Investigator or the physician designee. All morning vital signs and 12-lead ECGs will be performed prior to breakfast.

Continuous Lead II ECG monitoring (telemetry) will be conducted from 30 minutes prior to till 8 hours post dosing on Days -1, 1, 6, 8 and 15. These data will not be recorded on the database and are for visual monitoring. Any abnormality detected by the device or the investigator will be printed out and retained as source data. Any clinically significant abnormalities will be recorded as adverse events.

* This section of the protocol has been revised. Please refer to the section of this document titled PROTOCOL AMENDMENTS (Amendment DEU-5, 27 September 2007) for a detailed description of the specific changes.

9.5.4. Vital Signs

Systolic and diastolic blood pressure and pulse rate measurements will be assessed with a completely automated device consisting of an inflatable cuff and an oscillatory detection system. All values will be registered on a built-in digital recording system so that measurements are observer-independent. Manual blood pressure reading may be obtained in the event of instrument malfunction.

Systolic and diastolic blood pressure and heart rate measurements will be assessed following 5 minutes in the supine position and 3 minutes after standing (to assess orthostasis) using the contra-lateral arm to that used for blood sampling.

Vital signs (blood pressure and heart rate, supine) will be performed at screening and at T0, T2, T6 and T12 on Day –1 where T corresponds the dosing time on dosing days. Vital signs will also be performed at 0 (predose), 2, 6, 12, 24 and 48 hours post dose on Days 1 and 16, and at 2, 6 and 12 hours on Days 3 to 15 and once at the follow-up visit. The vital signs will be measured in triplicate 2 minutes apart at each predefined point except at screening and the Follow-up visit. The average of the triplicate measurements at each time point on Day –1 will serve each subject's time-matched baseline value for the corresponding parameters on Days 1 to 20. All morning vital signs will be performed prior to breakfast.

Orthostatic vital signs will be measured on Day –2 and at 4 and 8 hours post dose or corresponding to dosing time on Days 1 to 20. Orthostatic vital signs will also be measured in triplicate.

Body temperature will be measured at screening in the morning, on Days -2, Days 1 to 20 and at the follow-up visit.

Height will be measured at the screening visit and will be utilized to calculate BMI and BSA. Weight will be measured at screening, on a calibrated scale in the morning, prior to meals and after voiding on Days -1 to 20 and at the Follow-up visit. Subjects will wear a hospital gown or light clothes, without shoes for each of the measurements. Scale calibration will occur weekly, and records stored in study files.

9.5.5. Physical Examination

Physical examinations will be performed at screening and on Days –2, 15, 20 and at the final follow-up visit. The physical examination will include a thorough whole body skin assessment. Any observed skin abnormalities should be recorded in the eCRFs. The study investigator, or authorized designee, will perform the physical examinations. If any clinically significant change is noted from Screening, it will be reported as an adverse event and will be followed up to resolution or reaching a stable end point.

The investigator will follow any clinically significant abnormalities persisting at the end of the study until resolution or until reaching a clinically stable endpoint.

9.5.6. Monitoring Blood Glucose Levels

During the washout period starting 16 days prior to dosing, subjects will be provided with glucometers and instructed to monitor their blood glucose levels at least once each morning (fasting sample) and post lunch. The results will be recorded on a diary card and reported to the study center on Day -9 for entry into the eCRF. The subject will be instructed to report any signs or symptoms of hypoglycemia, fasting blood glucose values > 13.3 mM (240 mg/dL), post lunch values > 19.4 mM (350 mg/dL) and blood glucose values < 2.8 mM (50 mg/dL) to the study center. Subjects will be instructed to resume their previous oral anti-diabetic agent at any time during the washout period if confirmed fasting blood glucose levels rise above 13.3 mM (240 mg/dL) or postprandial glucose levels rise above 19.4 mM (350 mg/dL) following lunch. Instruction will also include reinforcement of signs and symptoms of hypoglycemia.

Investigational staff will monitor subjects for symptoms possibly indicative of hypoglycemia throughout the in-clinic residence, Days -3 to 20. Real time blood glucose concentrations will be determined prior to each standardized breakfast and 2 hours post each lunch using a glucose analyzer. On Days -1, 1 and 16 the measurements will be performed prior to breakfast and 2 hours post each meal. Blood glucose will be immediately measured using a glucose analyzer should hypoglycemia symptoms appear and confirmed by conventional venous sampling. Attachment 7 provides the clinical signs, symptoms, grading scale, and treatment for hypoglycemia. Should

hypoglycemia occur it should be entered in the eCRF adverse event page (see Attachment 7).

9.5.7. Monitoring for skin reactions

Subjects will be domiciled continuously from Days -3 through Day 20 and instructed to avoid direct exposure to sunlight or artificial sources of intense light for at least 96 hours post the last dose to minimize the risk of potential photosensitivity. In addition, subjects will be closely monitored for potential skin reactions with a thorough examination of the whole body skin for skin reactions (e.g. erythema, rash) by a physician or his/her designee at screening, on Days -1 through Day 20 at each dose level.

10. SUBJECT COMPLETION/WITHDRAWAL

10.1. Completion

A subject will be considered as having completed the study if he/she has completed all assessments up to and including all Follow-up assessments. However, data for all subjects receiving at least 1 dose of study drug and at least 1 pharmacodynamic assessment post randomization will be included in the pharmacodynamic analysis. Data for all subjects receiving a dose of JNJ-28431754 will be included in the pharmacokinetic analysis.

10.2. Discontinuation of Treatment

A subject should be discontinued from study treatment if

- the investigator believes that for safety reasons (e.g., adverse event) it is in the best interest of the subject to stop treatment
- any subject with a confirmed fasting blood glucose elevated above 13.3 mM (240 mg/dL) or a confirmed postprandial glucose level above 19.4 mM (350 mg/dL) following lunch will not be further administered study medication, and will be treated with appropriate hypoglycemic therapy and monitored until blood glucose levels have stabilized.

If a subject discontinues treatment before the end of the double-blind phase, further assessments and final Follow-up will be done unless the subject is withdrawn from the study (section 10.3).

10.3. Withdrawal From the Study

A subject will be withdrawn from the study for any of the following reasons:

1. lost to Follow-up
2. withdrawal of consent

3. safety reasons

No further dose escalation will occur if either more than 50% of the subjects experience moderate or severe adverse events or one subject experiences a serious adverse event of which a relationship to the trial medication can not be excluded.

When a subject withdraws before completing the study, the reason for withdrawal is to be documented on the CRF and in the source document. Every effort should be made to have the subject return to the study center and complete the assessments required for the Follow-up visit.

Study drug assigned to the withdrawn subject may not be assigned to another subject.

In case a subject is lost-to-Follow-up, every possible effort must be made by the study site personnel to contact the subject and determine the reason for discontinuation. The measures taken to follow up must be documented.

Subjects withdrawing from the main part of the study have the following options regarding pharmacogenomic research:

The DNA extracted from the subject's blood will be retained and used in accordance with the subject's DNA informed consent.

The subject may withdraw consent for pharmacogenomic research, in which case, the DNA sample will be destroyed and no further testing will take place. To initiate the sample destruction process, the investigator must notify the sponsor site contact to request sample destruction. The sponsor site contact will, in turn, contact the pharmacogenomics representative for sample destruction. Upon request, the investigator will receive written confirmation from the sponsor that the sample has been destroyed.

Withdrawal from Pharmacogenomic Research Only

- The subject may also withdraw consent for pharmacogenomic research only, while remaining in the clinical study. If a subject withdraws consent for pharmacogenomic research, any DNA extracted from the subject’s blood will be destroyed. The sample destruction process will proceed as described above. After the clinical study is over, the sample will be made non-identifiable and cannot be found in order to be destroyed. If the sample has already undergone conversion to the non-identifiable format, the sponsor will notify the investigator in writing.

11. STATISTICAL METHODS

All data will be fully listed. Unless otherwise noted, all the measures indicated below will be summarized with descriptive statistics for each dose level and all placebo observations grouped together.

Subjects who need to restart their previous anti-diabetic medications during the treatment period will stop receiving study medication and will be closely monitored. These subjects will be withdrawn from the study; however, all safety, tolerability, PK and/or PD data collected from these subjects will be included in the final data analysis.

All statistical analyses will be considered exploratory and interpreted as such. No corrections will be made for multiple comparisons.

11.1. Sample Size Determination

It is estimated that a sample size of 20 (16 receiving active JNJ-28431754 and 4 receiving placebo) subjects with T2DM should be sufficient to detect a 15% reduction in 24-hour mean plasma glucose AUC with 80% power, assuming a one-sided test and a coefficient of variation of 18 percent[19].

11.2. Pharmacokinetics

Data for all subjects receiving a dose of active study drug will be included in the pharmacokinetic analysis. Pharmacokinetic parameters will be summarized and descriptive statistics (including means, median, standard deviations and coefficients of variation) will be generated for each dose. Dose proportionality will be assessed graphically for PK parameters. The graphical assessment of dose proportionality will be performed for both single dose PK parameters (AUC_{24hr} and C_{max}) as well as steady state AUCss and Cmax,ss after multiple daily dosing. At each dose level, AUC_{24hr} and C_{max} for Days 1 and 16 will also be compared. In addition, the effect of dose and time on other pharmacokinetic parameters will also be explored, as appropriate.

11.3. Pharmacodynamic Analyses

Pharmacodynamic analyses will be performed on all subjects receiving at least one dose of JNJ-28431754 or placebo and with at least 1 pharmacodynamic assessment. Summary statistics will be generated for all pharmacodynamic parameters.

The primary pharmacodynamic assessments will focus on

Change from baseline mean 24-hour plasma glucose concentration on Days 1 and 16

Change from baseline urine glucose excretion (UGE) that include (a) cumulative amount over each 24 hours on Days 1, 2, 8, 12, 16, 17, 18, 19 with Day-1 values as baseline and (b) UGE rate, calculated as the UGE amount in each fraction divided by hours of each collection interval on Days 1, 2, 8, 12, 16, 17, 18, 19.

Mixed effect ANOVA modeling appropriate for this design will be used to assess the treatment effects on 24-hour mean plasma glucose and the UGE parameters (amount and rate). The mixed effect linear model will include dose and days of measurements as the independent variables. The estimated least-squares means and 95% confidence intervals for the pair-wise difference for different doses will be obtained.

Mixed effect ANOVA modeling will be used to investigate effect of JNJ-28431754 on 24-hour urine glucose excretion and rate of glucose excretion. The change from baseline for 0-24 hour amount of glucose excretion will be calculated and analyzed by fitting a mixed effect linear model will include dose and days of measurements as the independent variables. The estimated least-squares means and appropriate confidence intervals for the pair-wise difference of the mean change from baseline in 0-24 hour urine glucose for different doses will be obtained. Also, rate of excretion of urine glucose will be similarly analyzed using mixed effect ANOVA model and the estimated least-squares means and appropriate confidence intervals for the pair-wise difference of the mean rate of excretion of urine glucose for different doses will be reported.

The secondary pharmacodynamic assessments are provided in Section 9.3.2.

These secondary endpoints will be analyzed based on appropriate statistical models that will be detailed in the Statistical Analysis Plan for this protocol.

11.4. Safety Analyses

Safety from screening (i.e., signing informed consent) through follow-up will be evaluated by examining incidence, severity, relationship to study medication, and type of adverse events; changes in clinical laboratory

results; physical examination; vital signs measurements; ECG. Data will be summarized using descriptive statistics. Descriptive analysis will be performed for creatinine clearance, fractional and cumulative urinary excretion of electrolytes, amino acids, albumin, osmolality and tubular enzymes.

Adverse Events

The original terms used in the CRFs by investigators to identify adverse events will be coded using the Medical Dictionary for Regulatory Activities (MedDRA). The percentage of subjects with specific treatment-emergent adverse events will be summarized for each treatment group.

Special attention will be given to those subjects who have discontinued treatment due to an adverse event or who experienced a severe or a serious adverse event.

Clinical Laboratory Tests

Laboratory data will be summarized by the type of laboratory test. Normal reference ranges and markedly abnormal results (specified in the Statistical Analysis Plan) will be used in the summary of laboratory data. Descriptive statistics will be calculated for each laboratory analyte at baseline and at each scheduled time point. Changes from baseline results will be presented in pre- versus posttreatment cross tabulations (with classes for below, within, and above normal ranges). A listing of subjects with any laboratory results outside the reference ranges will also be provided.

Cardiovascular Safety

The effects on cardiovascular variables will be evaluated by means of descriptive statistics and frequency tabulations. These tables will include shifts from baseline values (the predose ECG will be used as baseline) to allow detection of relevant changes in individuals.

The ECG variables that will be analyzed are heart rate, PR interval, QRS interval, QT interval, and QT interval corrected for heart rate (QTcB and QTcF). Pulse and systolic blood pressure and diastolic blood pressure (supine and standing) will also be analyzed.

QTc values for the ECGs recorded during the study periods will be tabulated for their absolute values and also tabulated relative to baseline measurements, as listed below, in order to detect individual QTc changes.

- The number of male subjects with
  - QTc <430 msec
  - 430 ≤QTc ≤450 msec
  - QTc >450 msec
- The number of female subjects with
  - QTc <450 msec
  - 450 ≤QTc ≤470 msec
  - QTc >470 msec
- The number of subjects with QTc >500 msec
- The number of subjects with an increase of the QTc interval with respect to baseline
  - <30 msec
  - 30 to 60 msec
  - >60 msec

All important abnormalities from the ECG readings, including changes in T-wave morphology and/or the occurrence of U-waves versus baseline recordings, will be reported.

Vital Signs and Physical Examination

Descriptive statistics will be provided to evaluate the changes at each scheduled time point.

11.5. Interim Analyses

No formal Interim Analysis is planned, however, a blinded data review will occur after each tested dose for the dose-escalation decision.

If requested by J&JPRD Clinical Team, an internal Data Review Committee (DRC), independent from the Clinical Team, may be established by the Sponsor to review unblinded data to aid decision-making during this study regarding the development of the compound. The DRC would ensure that confidentiality of any unblinded information is maintained until the study is terminated and the database is locked. Any consequent changes after an unplanned formal Interim Analysis will be specified in an amendment to the protocol.

The Sponsor's staff on the Clinical Study team responsible for coordinating the drug-development program may be partially unblinded after the completion of a dose level or cohort. In these instances, the integrity of the study will be maintained by providing unblinded summary outputs only to those Sponsor staff responsible for coordinating the drug-development program. Those involved in contact with study subjects or responsible for collecting and cleaning the clinical data will not have access to the unblinded summary outputs.

12. ADVERSE EVENT REPORTING

Timely, accurate, and complete reporting and analysis of safety information from clinical studies are crucial for the protection of subjects, investigators, and the sponsor, and are mandated by regulatory agencies worldwide. The sponsor has established Standard Operating Procedures (SOPs) in conformity with regulatory requirements worldwide to ensure appropriate reporting of safety information; all clinical studies conducted by the sponsor or its affiliates will be conducted in accordance with those procedures.

12.1. Definitions

12.1.1. Adverse Event Definitions and Classifications

- **Adverse Event**
  An adverse event is any untoward medical occurrence in a clinical study subject administered a pharmaceutical product. An adverse event does not necessarily have a causal relationship with the treatment. An adverse event can therefore be any unfavorable and unintended sign (including an abnormal finding), symptom, or disease temporally associated with the use of a medicinal (investigational) product, whether or not related to the medicinal (investigational) product. (Definition per International Conference on Harmonisation [ICH])

  This includes any occurrence that is new in onset or aggravated in severity or frequency from the baseline condition, or abnormal results of diagnostic procedures, including laboratory test abnormalities.

  Note: The sponsor collects adverse events starting with the signing of the informed consent.

- **Serious Adverse Event**
  A serious adverse event as defined by ICH is any untoward medical occurrence that at any dose meets any of the following conditions:
  - results in death

- is life-threatening
  (The subject was at risk of death at the time of the event. It does not refer to an event that hypothetically might have caused death if it were more severe.)
- requires inpatient hospitalization or prolongation of existing hospitalization
- results in persistent or significant disability/incapacity, or
- is a congenital anomaly/birth defect

Note: Medical and scientific judgment should be exercised in deciding whether expedited reporting is also appropriate in situations other than those listed above. For example, important medical events may not be immediately life threatening or result in death or hospitalization but may jeopardize the subject or may require intervention to prevent one of the outcomes listed in the definition above. Any adverse event is considered a serious adverse event if it is associated with clinical signs or symptoms judged by the investigator to have a significant clinical impact.

- **Unlisted (Unexpected) Adverse Event**
  An unlisted adverse event, the nature or severity of which is not consistent with the applicable product information. For an investigational product, the expectedness of an adverse event will be determined by whether or not it is listed in the Investigator's Brochure.
- **Associated With the Use of the Drug**
  An adverse event is considered associated with the use of the drug if the attribution is possible, probable, or very likely by the definitions listed in Section 12.1.2.

12.1.2. Attribution Definitions

- **Not related**
  An adverse event that is not related to the use of the drug.
- **Doubtful**
  An adverse event for which an alternative explanation is more likely, e.g., concomitant drug(s), concomitant disease(s), or the relationship in time suggests that a causal relationship is unlikely.
- **Possible**
  An adverse event that might be due to the use of the drug. An alternative explanation, e.g., concomitant drug(s), concomitant disease(s), is inconclusive. The relationship in time is reasonable; therefore, the causal relationship cannot be excluded.
- **Probable**
  An adverse event that might be due to the use of the drug. The relationship in time is suggestive (e.g., confirmed by dechallenge). An alternative explanation is less likely, e.g., concomitant drug(s), concomitant disease(s).

- **Very likely**
  An adverse event that is listed as a possible adverse reaction and cannot be reasonably explained by an alternative explanation, e.g., concomitant drug(s), concomitant disease(s). The relationship in time is very suggestive (e.g., it is confirmed by dechallenge and rechallenge).

12.2. Procedures

12.2.1. All Adverse Events

All adverse events will be reported from the time a signed and dated informed consent form is obtained until completion of the last study-related procedure. Those meeting the definition of serious adverse events must be reported using the Serious Adverse Event Form, including serious adverse events spontaneously reported to the investigator within 30 days after the subject has completed the study (including poststudy follow up). The sponsor will evaluate any safety information that is spontaneously reported by an investigator beyond the time frame specified in the protocol.

All events that meet the definition of a serious adverse event will be reported as serious adverse events, regardless of whether they are protocol-specific assessments.

All adverse events, regardless of seriousness, severity, or presumed relationship to study therapy, must be recorded using medical terminology in the source document and the CRF. Whenever possible, diagnoses should be given when signs and symptoms are due to a common etiology (e.g., cough, runny nose, sneezing, sore throat, and head congestion should be reported as "upper respiratory infection"). Investigators must record in the CRF their opinion concerning the relationship of the adverse event to study therapy. All measures required for adverse event management must be recorded in the source document and reported according to sponsor instructions.

The sponsor assumes responsibility for appropriate reporting of adverse events to the regulatory authorities. The sponsor will also report to the investigator all serious adverse events that are unlisted and associated with the use of the drug. The investigator (or sponsor where required) must report these events to the appropriate Independent Ethics Committee/Institutional Review Board (IEC/IRB) that approved the protocol unless otherwise required and documented by the IEC/IRB.

12.2.2. Serious Adverse Events

All serious adverse events occurring during clinical studies must be reported to the appropriate sponsor contact person by investigational staff within 24 hours of their knowledge of the event.

Information regarding serious adverse events will be transmitted to the sponsor using the Serious Adverse Event Form, which must be signed by a member of the investigational staff. The initial report of a serious adverse event may be made by facsimile (fax) or telephone. It is preferable that serious adverse events be reported via fax. Subsequent to a telephone report of a serious adverse event, a Serious Adverse Event Form must be completed by the investigational staff and transmitted to the sponsor within 1 working day.

All serious adverse events that have not resolved by the end of the study, or that have not resolved upon discontinuation of the subject's participation in the study, must be followed until any of the following occurs:

- the event resolves
- the event stabilizes
- the event returns to baseline, if a baseline value is available
- the event can be attributed to agents other than the study drug or to factors unrelated to study conduct
- when it becomes unlikely that any additional information can be obtained (subject or health care practitioner refusal to provide additional information, lost to follow-up after demonstration of due diligence with follow-up efforts)

The cause of death of a subject in a clinical study, whether or not the event is expected or associated with the investigational agent, is considered a serious adverse event. Any event requiring hospitalization (or prolongation of hospitalization) that occurs during the course of a subject's participation in a clinical study must be reported as a serious adverse event, except hospitalizations for:

- social reasons in absence of an adverse event
- surgery or procedure planned before entry into the study (must be documented in the CRF)

12.2.3. Pregnancies

Subject pregnancy must be reported by the investigational staff within 1 working day of their knowledge of the event using the pregnancy notification form. Any subject who becomes pregnant during the study must be promptly withdrawn from the study.

Because the study drug may have an effect on sperm, pregnancies in partners of male subjects included in the study will be reported by the investigational staff within 1 working day of their knowledge of the event using the pregnancy notification form.

Follow-up information regarding the outcome of the pregnancy and any postnatal sequelae in the infant will be required.

12.3. Contacting Sponsor Regarding Safety

The names of the individuals (and corresponding telephone numbers) who should be contacted regarding safety issues or questions regarding the study are listed on the Contact Information page(s), which will be provided as a separate document.

13. STUDY DRUG INFORMATION

13.1. Physical Description of Study Drug(s)

JNJ–28431754 is a white powder with very low aqueous solubility. JNJ–28431754 and its matching placebo will be dispensed using the provided oral dispenser and should be stored between 2° and 8°C and protected from white light. All study medication should be stored in a secure area under restricted access.

Active compound:	JNJ-28431754
Dosage form:	Liquid, suspension of JNJ-28431754 in 0.5% hypromellose
Strength:	5 and 50 mg/mL
Placebo:	0.5% hypromellose

Detailed instructions for the study drug dispensing and the storage conditions will be provided to the clinical study site.

13.2. Packaging

The study drug will be packaged according to current good manufacturing practices and local regulations. The JNJ–28431754 and its matching placebo

will be provided as bulk supplies (i.e., not packaged by individual subject numbers) in conventional packaging (i.e., not child resistant packaging) containing appropriate volumes.

13.3. Labeling

Study drug labels will contain information to meet the applicable regulatory requirements. The site will also be provided with dispensing labels to be applied at the time of dose preparation.

13.4. Preparation and Handling

The study medication should be stored at 2°C to 8°C and protected from white light. Detailed instructions for the study drug administration will be provided along with the clinical supply shipment.

13.5. Drug Accountability

The clinical investigator is responsible for ensuring that all study drug received at the site is inventoried and accounted for throughout the study. The dispensing of study drug to the subject, and the return of study drug from the subject (if applicable), must be documented on the drug accountability form. Subjects or their legally acceptable representative must be instructed to return all original containers, whether empty or containing study drug. Study drug returned by study subjects will be stored and disposed of according to the sponsor's instructions. Contents of the study drug containers must not be combined.

Study drug must be handled strictly in accordance with the protocol and the container label and will be stored in a limited access area or in a locked cabinet under appropriate environmental conditions. Unused study drug, and study drug returned by the subject (if applicable), must be available for verification by the sponsor's site monitor during on-site monitoring visits. The return to the sponsor of unused study drug, or used returned study drug for destruction, will be documented on the Drug Return Form.

Study drug should be dispensed under the supervision of the investigator, a qualified member of the investigational staff, or by a hospital/clinic pharmacist. Study drug will be supplied only to subjects participating in the study. Returned study drug must not be dispensed again, even to the same subject. Study drug may not be relabeled or reassigned for use by other

subjects. The investigator agrees neither to dispense the study drug from, nor store it at, any site other than the study sites agreed upon with the sponsor.

14. STUDY-SPECIFIC MATERIALS

The investigator will be provided with the following supplies:

- Oral solution dispenser
- Dispensing labels
- Randomization list (pharmacist only)
- Randomization code break envelopes
- Electronic CRFs (eCRF) and infrastructure
- PK sample labels

15. ETHICAL ASPECTS

15.1. Study-Specific Design Considerations

This is the first study with JNJ-28431754 in subjects with T2DM. JNJ-28431754 is an investigational drug that is currently being developed for the treatment of T2DM (see Section 1, Introduction).

Each subject must give written consent according to local requirements after the nature of the study has been fully explained. The consent form must be signed before performance of any study-related procedure. Subjects will be informed that their participation is voluntary, that they will derive no direct medical benefit from participating and that they may withdraw consent to participate at any time. Subjects will be fully informed of any new information as it becomes available throughout the course of the study as this may affect their decision to continue participation.

After initial screening, eligible subjects will discontinue their previous anti-diabetic medications starting 16 days prior to dosing on Day 1 and through to Day 22. Blood glucose levels are expected to rise modestly in many subjects, but fasting and post lunch blood glucose levels will be monitored at least daily throughout this study, and fasting and postprandial blood glucose levels will not be allowed to exceed approximately 240 mg/dL (13.3 mM) and 350 mg/dL (19.4 mM), respectively. It will be possible to rapidly resume anti-diabetic therapy, if needed. While chronically elevated (for years) and/or uncontrolled hyperglycemia in T2DM patients can lead to serious diabetic complications, short-term discontinuation of oral anti-diabetic medications (for a maximum of approximately 8 weeks in this

study) with modest blood glucose elevations and with daily medical monitoring is not expected to result in clinically meaningful short- or long-term consequences for the study subjects.

The available data from the single ascending dose study with JNJ-28431754 in healthy male subjects has provided adequate safety, tolerability, pharmacokinetic and pharmacodynamic information to justify cautious and well controlled administration of single and multiple ascending doses in Type 2 diabetic subjects. The study design for this first administration of repeated doses in T2DM subjects takes a careful approach involving escalation of the 14-day repeated dosing only after review of preliminary safety and tolerability data from a single dose administration at each dose level. Subject safety will be closely monitored particularly in relation to adverse effects observed in preclinical testing and in the first-in-human study, especially body fluid and electrolyte balance, skin reaction and GI tolerability. In addition, preliminary data from a 3-month GLP toxicology study in rats and dogs will be available after the initiation of the present study. If necessary, modifications in doses and study procedures to the present study will be made in response to new toxicity data from the 3-month rat toxicology study.

Type 2 diabetes affects both men and women, and thus evaluating the safety, tolerability and effects of JNJ-28431754 in women is desirable for further developing this new agent. In the present study, it is planned to include post-menopausal or surgically sterile women. Based on the safety, tolerability and exposure data from the ongoing FIH study in healthy male subjects, it is justified to include women in this study in the planned dose range.

Subjects in this study may be taking other medications, especially anti-dislipidemic and hypertension drugs, which poses a potential risk of drug-drug interactions with JNJ-28431754. Based on preclinical data, the drug has a low potential for CYP related DDIs. To minimize the risk, medications that have a higher likelihood of interacting with JNJ-28431754 (based on information on JNJ-28431754 to date and the mechanism of action) have been excluded as co-medications during the study. These include diuretics, beta-blockers (which can mask symptoms of hypoglycemia) and CYP 3A4 inhibitors and inducers as JNJ-28431754 is at least partially metabolized by CYP3A4 (Attachment 8). Since the potential for, and magnitude of,

interactions in vivo via CYP3A4 is not clear at this time, a cautious approach to exclude these co-medications is warranted. The potential for other drug-drug interactions does exist, and this will be closely monitored for during the study.

The total volume of blood drawn for laboratory evaluations throughout this study is approximately 475 mL for each subject and 511 mL for each subject participating in the potential daily twice-daily cohort. An additional 10 mL of blood will be collected once from subjects who consent to genetic testing. This volume is considered to be acceptable in an 8-week trial. Subjects will be advised not to donate blood for at least 90 days after completion of the study.

Subjects safety will be closely monitored by clinical and laboratory assessments. Particular attention will be paid to the potential human organ toxicity based on animal toxicology studies and the ongoing FIH study, ie, body fluid and electrolyte balance, blood glucose levels and GI tolerability.

Subjects will be closely monitored for potentially drug-induced hypoglycemia, by frequent assessment of blood glucose levels and careful monitoring for signs and symptoms of hypoglycemia. A bedside glucose measurement using a glucose analyzer will be performed at scheduled time points and on an as needed basis when subjects develop symptoms suggestive of hypoglycemia. Grading and Treatment of Hypoglycemic Events are included in Attachment 7.

In the in vivo phototoxicity study in rats, JNJ-28431754 showed mild phototoxicity to skin, but not to eyes. Subjects will be instructed to avoid direct sunlight, or any artificial intense light, especially UV light, up to 96 hours (or at least 5 estimated half lives of the drug) post the last drug administration, or if sunlight exposure cannot be avoided, to use protective clothing and broad-spectrum (UVA/UVB) sunscreens and sun block lipstick that have a skin protection factor of at least 15.

As with all safety, tolerability, PK and PD studies, there are risk/discomforts associated with various study procedures. The insertion of the catheter and the venipuncture may cause brief pain, light bleeding and possibly a minor infection. However, the discomforts and the potential risks associated with venipuncture are generally minimum and transient.

15.2. Regulatory Ethics Compliance

15.2.1. Investigator Responsibilities

The investigator is responsible for ensuring that the clinical study is performed in accordance with the protocol, current ICH guidelines on Good Clinical Practice (GCP), and applicable regulatory requirements.

GCP is an international ethical and scientific quality standard for designing, conducting, recording, and reporting studies that involve the participation of human subjects. Compliance with this standard provides public assurance that the rights, safety, and well being of study subjects are protected, consistent with the principles that originated in the Declaration of Helsinki, and that the clinical study data are credible.

15.2.2. Independent Ethics Committee or Institutional Review Board (IEC/IRB)

Before the start of the study, the investigator (or sponsor where required) will provide the IEC/IRB with current and complete copies of the following documents:

- final protocol and, if applicable, amendments
- sponsor-approved informed consent form (and any other written materials to be provided to the subjects)
- Investigator's Brochure (or equivalent information) and amendments
- sponsor-approved subject recruiting materials
- information on compensation for study-related injuries or payment to subjects for participation in the study, if applicable
- investigator's curriculum vitae or equivalent information (unless not required, as documented by IEC/IRB)
- information regarding funding, name of the sponsor, institutional affiliations, other potential conflicts of interest, and incentives for subjects
- any other documents that the IEC/IRB requests to fulfill its obligation

This study will be undertaken only after IEC/IRB has given full approval of the final protocol, amendments (if any), the informed consent form, applicable recruiting materials, and subject compensation programs, and the sponsor has received a copy of this approval. This approval letter must be dated and must clearly identify the documents being approved.

IEC/IRB approval for the pharmacogenomic research component of the clinical study and for the pharmacogenomic informed consent form must be

obtained. IEC/IRB approval can be obtained for the protocol independent of approval for pharmacogenomic research.

During the study the investigator (or sponsor where required) will send the following documents to the IEC/IRB for their review and approval, where appropriate:

- protocol amendments
- revision(s) to informed consent form and any other written materials to be provided to subjects
- if applicable, new or revised subject recruiting materials approved by the sponsor
- revisions to compensation for study-related injuries or payment to subjects for participation in the study, if applicable
- Investigator's Brochure amendments or new edition(s)
- summaries of the status of the study (at least annually or at intervals stipulated in guidelines of the IEC/IRB)
- reports of adverse events that are serious, unlisted, and associated with the investigational drug
- new information that may adversely affect the safety of the subjects or the conduct of the study
- deviations from or changes to the protocol to eliminate immediate hazards to the subjects
- report of deaths of subjects under the investigator's care
- notification if a new investigator is responsible for the study at the site
- any other requirements of the IEC/IRB

For protocol amendments that increase subject risk, the amendment and applicable informed consent form revisions must be submitted promptly to the IEC/IRB for review and approval before implementation of the change(s).

At least once a year, the IEC/IRB will be asked to review and reapprove this clinical study. This request should be documented in writing.

At the end of the study, the investigator (or sponsor where required) will notify the IEC/IRB about the study completion.

15.2.3. Informed Consent

Each subject must give written consent according to local requirements after the nature of the study has been fully explained. The consent form must be signed before performance of any study-related activity. The consent form that is used must be approved by both the sponsor and by the reviewing IEC/IRB. The informed consent should be in accordance with principles that originated in the Declaration of Helsinki, current ICH and GCP guidelines, applicable regulatory requirements, and sponsor policy.

Before entry into the study, the investigator or an authorized member of the investigational staff must explain to potential subjects the aims, methods, reasonably anticipated benefits, and potential hazards of the study, and any discomfort it may entail. Subjects will be informed that their participation is voluntary and that they may withdraw consent to participate at any time. They will be informed that choosing not to participate will not affect the care the subject will receive for the treatment of his/her disease. Subjects will be told that alternative treatments are available if they refuse to take part and that such refusal will not prejudice future treatment. Finally, they will be told that the investigator will maintain a subject identification register for the purposes of long-term follow-up if needed and that their records may be accessed by health authorities and authorized sponsor staff without violating the confidentiality of the subject, to the extent permitted by the applicable law(s) or regulations. By signing the informed consent form the subject is authorizing such access, and agrees to be recontacted after study completion, by health authorities and authorized sponsor staff, for the purpose of obtaining consent for additional safety evaluations if needed.

The subject will be given sufficient time to read the informed consent form and the opportunity to ask questions. After this explanation and before entry into the study, consent should be appropriately recorded by means of the subject's dated signature. After having obtained the consent, a copy of the informed consent form must be given to the subject.

Subjects will also be asked to consent to participate in a pharmacogenomic research component of the study (where local regulations permit). After informed consent for the clinical study is appropriately obtained, the subject will be asked to sign and personally date a separate pharmacogenomic informed consent form indicating agreement to participate in optional

pharmacogenomic research. A copy of the signed pharmacogenomic informed consent form will be given to the subject.

If the subject or legally acceptable representative is unable to read or write, an impartial witness should be present for the entire informed consent process (which includes reading and explaining all written information) and should personally date and sign the informed consent form after the oral consent of the subject or legally acceptable representative is obtained.

15.2.4. Privacy of Personal Data

The collection and processing of personal data from subjects enrolled in this study will be limited to those data that are necessary to investigate the efficacy, safety, quality, and utility of the investigational product(s) used in this study.

These data must be collected and processed with adequate precautions to ensure confidentiality and compliance with applicable data privacy protection laws and regulations.

The sponsor ensures that the personal data will be

- processed fairly and lawfully
- collected for specified, explicit, and legitimate purposes and not further processed in a way incompatible with these purposes
- adequate, relevant, and not excessive in relation to said purposes
- accurate and, where necessary, kept current

Explicit consent for the processing of personal data will be obtained from the participating subject (or his/her legally acceptable representative) before collection of data. Such consent should also address the transfer of the data to other entities and to other countries.

The subject has the right to request through the investigator access to his/her personal data and the right to request rectification of any data that are not correct or complete. Reasonable steps should be taken to respond to such a request, taking into consideration the nature of the request, the conditions of the study, and the applicable laws and regulations.

Appropriate technical and organizational measures to protect the personal data against unauthorized disclosures or access, accidental or unlawful

destruction, or accidental loss or alteration must be put in place. Sponsor personnel whose responsibilities require access to personal data agree to keep the identity of study subjects confidential.

For those subjects who gave consent to store DNA samples for future research (Part 2), samples and corresponding relevant clinical data will undergo a procedure to make the samples and data non-identifiable, which involves removal of personal identifiers. Samples will be stored indefinitely until exhausted. Future genetic research will be restricted to that which is related to the drug or the indications for which the drug is developed. For subjects submitting a written request for their data generated on identifiable samples (Part 1), the sponsor will provide the raw data but cannot undertake to make decisions regarding the significance of any findings resulting from this pharmacogenomic research, and cannot, therefore, undertake to provide any genetic counselling. Genotypic data generated on non-identifiable samples (Part 2) cannot be returned to individual subjects.

16. ADMINISTRATIVE REQUIREMENTS

16.1. Protocol Modifications

Neither the investigator nor the sponsor will modify this protocol without a formal amendment. All protocol amendments must be issued by the sponsor, and signed and dated by the investigator. Protocol amendments must not be implemented without prior IEC/IRB approval, or when the relevant competent authority has raised any grounds for non-acceptance, except when necessary to eliminate immediate hazards to the subjects, in which case the amendment must be promptly submitted to the IEC/IRB and relevant competent authority. When the change(s) involves only logistic or administrative aspects of the study, the IRB (and IEC where required) only needs to be notified.

In situations requiring a departure from the protocol, the investigator or other physician in attendance will contact the appropriate sponsor representative by fax or telephone (see Contact Information pages provided separately). If possible, this contact will be made <u>before</u> implementing any departure from the protocol. In all cases, contact with the sponsor must be made as soon as possible in order to discuss the situation and agree on an appropriate course of action. The data recorded in the <u>CRF and source document</u> will reflect any

departure from the protocol, and the source documents will describe this departure and the circumstances requiring it.

16.2. Regulatory Documentation

16.2.1. Regulatory Approval/Notification

This protocol and any amendment(s) must be submitted to the appropriate regulatory authorities in each respective country, if applicable. A study may not be initiated until all local regulatory requirements are met.

16.2.2. Required Prestudy Documentation

The following documents must be provided to the sponsor before shipment of study drug to the investigational site:

- protocol and amendment(s), if any, signed and dated by the investigator
- a copy of the dated and signed written IEC/IRB approval of the protocol, amendments, informed consent form, any recruiting materials, and if applicable, subject compensation programs. This approval must clearly identify the specific protocol by title and number and must be signed by the chairman or authorized designee.
- name and address of the IEC/IRB including a current list of the IEC/IRB members and their function, with a statement that it is organized and operates according to GCP and the applicable laws and regulations. If accompanied by a letter of explanation from the IEC/IRB, a general statement may be substituted for this list. If an investigator or a member of the investigational staff is a member of the IEC/IRB, documentation must be obtained to state that this person did not participate in the deliberations or in the vote/opinion of the study.
- regulatory authority approval or notification, if applicable
- signed and dated statement of investigator (e.g., Form FDA 1572), if applicable
- documentation of investigator qualifications (e.g., curriculum vitae)
- completed investigator financial disclosure form from the investigator
- signed and dated clinical trial agreement, which includes the financial agreement
- any other documentation required by local regulations

The following documents must be provided to the sponsor before enrollment of the first subject:

- completed investigator financial disclosure forms from all subinvestigators

- documentation of subinvestigator qualifications (e.g., curriculum vitae)
- photocopy of the site signature log, describing delegation of roles and responsibilities at the start of the study
- name and address of any local laboratory conducting tests for the study, and a dated copy of current laboratory normal ranges for these tests
- local laboratory documentation demonstrating competence and test reliability (e.g., accreditation/license), if applicable.

16.3. Subject Identification Register and Subject Screening Log

The investigator agrees to complete a subject identification register to permit easy identification of each subject during and after the study. This document will be reviewed by the sponsor site contact for completeness.

The subject identification register will be treated as confidential and will be filed by the investigator in the trial center file. To ensure subject confidentiality, no copy will be made. All reports and communications relating to the study will identify subjects by initials and assigned number only.

The investigator must also complete a subject-screening log, which reports on all subjects who were seen to determine eligibility for inclusion in the study.

16.4. Case Report Form Completion

CRFs are provided for each subject in printed or electronic format.

Electronic Data Capture (EDC) will be used for this study. The majority of the study data will be transcribed by study personnel from the source documents onto an electronic CRF and transmitted in a secure manner to the sponsor. The electronic file will be considered as the CRF.

All data relating to the study must be recorded in CRFs prepared by the sponsor. Data must be entered into CRFs in English. The CRFs are to be completed at the time of the subject's visit, with the exception of results of tests performed outside the investigator's office, so that they always reflect the latest observations on the subjects participating in the study.

Every effort should be made to ensure that all subjective measurements (e.g., pain scale information or other questionnaires) to be recorded on the CRF

are completed by the same individual who made the initial baseline determinations. The investigator must verify that all data entries in the CRFs are accurate and correct.

All CRF entries, corrections, and alterations must be made by the investigator or other authorized study-site personnel.

16.5. Data Quality Assurance

Steps to be taken to ensure the accuracy and reliability of data include the selection of qualified investigators and appropriate study centers, review of protocol procedures with the investigator and associated personnel before the study, periodic monitoring visits by the sponsor, and direct transmission of clinical laboratory data from a central laboratory into the sponsor's data base. Written instructions will be provided for collection, preparation, and shipment of blood, plasma, and urine samples. CRF completion guidelines will be provided and reviewed with study personnel before the start of the study. The sponsor will review CRFs for accuracy and completeness during on-site monitoring visits and after their return to the sponsor; any discrepancies will be resolved with the investigator or designee, as appropriate. The data will be entered into the clinical study database and verified for accuracy.

16.6. Record Retention

In compliance with the ICH/GCP guidelines, the investigator/institution will maintain all CRFs and all source documents that support the data collected from each subject, as well as all study documents as specified in ICH/GCP Section 8, Essential Documents for the Conduct of a Clinical Trial, and all study documents as specified by the applicable regulatory requirement(s). The investigator/institution will take measures to prevent accidental or premature destruction of these documents.

Essential documents must be retained until at least 2 years after the last approval of a marketing application in an ICH region and until there are no pending or contemplated marketing applications in an ICH region or until at least 2 years have elapsed since the formal discontinuation of clinical development of the investigational product. These documents will be retained for a longer period if required by the applicable regulatory requirements or by an agreement with the sponsor. It is the responsibility of

the sponsor to inform the investigator/institution as to when these documents no longer need to be retained.

If the responsible investigator retires, relocates, or for other reasons withdraws from the responsibility of keeping the study records, custody must be transferred to a person who will accept the responsibility. The sponsor must be notified in writing of the name and address of the new custodian. Under no circumstance shall the investigator relocate or dispose of any study documents before having obtained written approval from the sponsor.

For CRFs completed on NCR paper, one copy is to be retained in the archives of the sponsor from the country in which the study is performed. A second copy must be archived by the investigator.

If it becomes necessary for the sponsor or the appropriate regulatory authority to review any documentation relating to this study, the investigator must permit access to such reports.

16.7. Monitoring

The sponsor will perform on-site monitoring visits as frequently as necessary. The monitor will record dates of the visits in a study center visit log that will be kept at the site. The first post-initiation visit will usually be made as soon as possible after enrollment has begun. At these visits, the monitor will compare the data entered into the CRFs with the hospital or clinic records (source documents). The nature and location of all source documents will be identified to ensure that all sources of original data required to complete the CRF are known to the sponsor and investigational staff and are accessible for verification by the sponsor site contact. If electronic records are maintained at the investigational site, the method of verification must be discussed with the investigational staff. At a minimum, source documentation must be available to substantiate: subject identification, eligibility, and participation; proper informed consent procedures; dates of visits; adherence to protocol procedures; records of safety and efficacy parameters; adequate reporting and follow-up of adverse events; administration of concomitant medication; drug receipt/dispensing/return records; study drug administration information; and date of subject completion, discontinuation from treatment, or withdrawal from the study, and the reason if appropriate. Specific items required as source documents will be reviewed with the investigator before the study.

If data are recorded directly into the CRF, at a minimum there should be an entry in the medical record that each of the assessments was done, and by whom and the date it was done. It is recommended that the author of an entry in the source documents be identifiable.

Direct access to source documentation (medical records) must be allowed for the purpose of verifying that the data recorded in the CRF are consistent with the original source data. Findings from this review of CRFs and source documents will be discussed with the investigational staff. The sponsor expects that, during monitoring visits, the relevant investigational staff will be available, the source documentation will be available, and a suitable environment will be provided for review of study-related documents. The monitor will meet with the investigator on a regular basis during the study to provide feedback on the study conduct.

If corrections to a CRF are needed after removal of the original CRF copy from the investigational site, a DCF will be used.

16.8. Study Completion/Termination

16.8.1. Study Completion

The study is considered completed with the last visit of the last subject undergoing the study. The final data from the investigational site will be sent to the sponsor (or designee) no more than 24 hours following completion of the final subject visit at that site.

16.8.2. Study Termination

The sponsor reserves the right to close the investigational site or terminate the study at any time. Investigational sites will be closed upon study completion. An investigational site is considered closed when all required documents and study supplies have been collected and a site closure visit has been performed.

The investigator may initiate site closure at any time, provided there is reasonable cause and sufficient notice is given in advance of the intended termination.

Reasons for the early closure of an investigational site by the sponsor or investigator, or termination of a study by the sponsor, may include but are not limited to:

- failure of the investigator to comply with the protocol, the sponsor's procedures, or GCP guidelines
- safety concerns
- sufficient data suggesting lack of efficacy
- inadequate recruitment of subjects by the investigator

16.9. On-Site Audits

Representatives of the sponsor's clinical quality assurance department may visit the site to conduct an audit of the study in compliance with regulatory guidelines and company policy. These audits will require access to all study records, including source documents, for inspection and comparison with the CRFs. Subject privacy must, however, be respected.

Similar auditing procedures may also be conducted by agents of any regulatory body reviewing the results of this study in support of a regulatory submission. The investigator should immediately notify the sponsor if they have been contacted by a regulatory agency concerning an upcoming inspection.

16.10. Use of Information and Publication

All information, including but not limited to information regarding JNJ-28431754 or the sponsor's operations (e.g., patent application, formulas, manufacturing processes, basic scientific data, prior clinical data, formulation information) supplied by the sponsor to the investigator and not previously published, and any data including pharmacogenomic research data generated as a result of this study, are considered confidential and remains the sole property of the sponsor. The investigator agrees to maintain this information in confidence and use this information only to accomplish this study, and will not use it for other purposes without the sponsor's prior written consent.

The investigator understands that the information developed in the clinical study will be used by the sponsor in connection with the continued development of JNJ-28431754, and thus may be disclosed as required to other clinical investigators or regulatory agencies. To permit the information derived from the clinical studies to be used, the investigator is obligated to provide the sponsor with all data obtained in the study.

The results of the study will be reported in a Clinical Study Report generated by the sponsor and will contain all data from all investigational sites. Results of any pharmacogenomic analyses performed after the Clinical Study Report has been issued will be reported in a separate report and will not require a revision of the Clinical Study Report. Study subject identifiers will not be used in publication of pharmacogenomic results. Any work created in connection with performance of the study and contained in the data that can benefit from copyright protection (except any publication by the investigator as provided for below) shall be the property of the sponsor as author and owner of copyright in such work.

The sponsor shall have the right to publish such data and information without approval from the investigator. If an investigator wishes to publish information from the study, a copy of the manuscript must be provided to the sponsor for review at least 60 days before submission for publication or presentation. Expedited reviews will be arranged for abstracts, poster presentations, or other materials. If requested by the sponsor in writing, the investigator will withhold such publication for up to an additional 60 days to allow for filing of a patent application. In the event that issues arise regarding scientific integrity or regulatory compliance, the sponsor will review these issues with the investigator. The sponsor will not mandate modifications to scientific content and does not have the right to suppress information. The investigator will recognize the integrity of a multicenter study by not publishing data derived from the individual site until the combined results from the completed study have been published in full, within 12 months after conclusion, abandonment, or termination of the study at all sites, or the sponsor confirms there will be no multicenter study publication. Authorship of publications resulting from this study will be based on generally accepted criteria for major medical journals.

17. REFERENCES

1. Leahy J et al b-cell dysfunction induced by chronic yperglycemia: current ideas on mechanism of impaired glucose-insulin insulin secretion. Diabetes Care (1992); 15:442-455.

2. Rossetti L et al. Glucose toxicity. Diabetes Care (1990); 13:610-630.

3. Gaede P, Vedel P, Larsen N, et al. Multifactorial intervention and cardiovascular disease in patients with type 2 diabetes. N. Eng. J. Med (2003), 348:383-393.

4. The Diabetes Control and Complications Trial Research Group: The relationship of glycemic exposure (HbA1c) to the risk of development and progression of retinopathy in the Diabetes Control and Complications Trial. Diabetes (1995); 44:968-993

5. The Diabetes Control and Complications Trial Research Group: Effect of intensive diabetes therapy on the development and progression of diabetic nephropathy in the Diabetes Control and Complications Trial. Kidney Int. (1995); 47:1703-1720

6. The Diabetes Control and Complications Trial Research Group: The effect of intensive diabetes therapy on the development and progression of neuropathy in the Diabetes Control and Complications Trial. Ann Intern Med (1995); 122:561-568

7. UK Prospective Diabetes Study (UKPDS) Group. Intensive blood-glucose countol with sulphonylrueas or insulin compared with conventional treatment and risk of complications in patients with Type 2 diabetes (UKPDS 33) Lancet; (1998) 352:837-853

8. Stratton IM et al Association of glycemia with macrovascular and microvascular domplications of Type 2 diabetes (UKPDS 35) BMJ; 2000 321(7258):405-412

9. Wright EM. Renal Na+-glucose cotransporters. Am J Physiol Renal Physiol 2001; 280:F10-18

10. Ehrenkranz et al. Phlorizin: a review Diabetes Metab Res Rew. 2005; 21:31-38

11. JNJ-28431754 Investigator's Brochure, April 2007, Johnson & Johnson Pharmaceutical Research and Development, L.L.C.

12. Flint A. et al. Reproducibility, power and validity of visual analogue scales in assessment of appetite sensations in single test meal studies. International Journal of Obesity. 2000; 24:38-48

13. Polidori D. SIARaA: A Robust Algebraic Method for Determining Insulin Sensitivity and Glucose Absorption Rates from Oral Glucose Tolerance Tests or Mixed Meals. Internal Report Johnson & Johnson Pharmaceutical Research and Development. December 12, 2006.

14. FDA Guidance for Industry and Reviewers: Estimating the safe starting dose in clinical trials for therapeutics in adult healthy volunteers (July 2005).

15. Lesley A et al. Assessing kidney function-measured and estimated glomerular filtration rate. N Engl J Med 2006; 354;2473-83.

16. Bazett HC. An analysis of the time-relationship of electrocardiograms. Heart 1919; 70:353-370.

17. Hodges M, Salerno D, Erlien D. Bazett's QT correction reviewed: evidence that a linear QT correction for heart rate is better. J Am Coll Cardiol 1983;1:694.

18. Sagie A, Larson MG, Goldberg RJ, Bengston JR, Levy D. An improved method for adjusting the QT interval for heart rate (the Framingham Heart Study). Am J Cardiol 1992;70:797-801.

19. EMEA (Human Medicines Evaluation Unit) Committee for Proprietary Medicinal Products (CPMP). The assessment of the potential for QT interval prolongation by non-cardiovascular medicinal products. 1996; 986.

20. Bo Ahrén, Eric Simonsson, et.al. Inhibition of Dipeptidyl Peptidase IV Improves Metabolic Control Over a 4- Week Study Period in Type 2 Diabetes, Diabetes Care, Vol. 25, No. 5, May 2002, pp 869-875.

21. Evidence-based nutrition principles and recommendations for the treatment and prevention of diabetes and related complications, Diabetes Care, 25:202-212.

ATTACHMENTS

Attachment 1:
Pharmacokinetic Sample Collection and Handling

Materials and Labeling

Blood will be collected in glass or polypropylene tubes containing the appropriate K3-EDTA anticoagulant. Resulting plasma samples must be stored in polypropylene storage tubes with polypropylene or polyethylene caps. No tubes with separation gel should be used.

Urine samples will be collected in polyethylene containers with polypropylene or polyethylene caps and stored in polypropylene tubes with polypropylene or polyethylene caps.

All tubes and containers will be labeled with preprinted labels. The preprinted information will include the study number, Case Report Form identification number (CRF i.d. #), treatment period, scheduled sampling day and time as stipulated in the flow chart, and the analyte name. No other information will be written on the labels.

Labels should be applied to the sample tubes as follows:

- Apply labels to the sample tubes so that they do not overlap and obscure any information. If possible expose an area between the 2 ends of the label to allow viewing of the contents of the tube.
- Do not alter the orientation of the label on the sample tube.
- Apply labels to all tubes in the same manner.

Preparation of Plasma Pharmacokinetic Samples

- Collect 3 mL of blood into the appropriate EDTA anticoagulant tube at each time point and place in ice until centrifuged.
- Record the exact date and time of sampling in the CRF.
- Gently invert the tubes 5 to 6 times to afford mixing, before processing.
- Centrifuge blood samples within 2 hours of collection in a clinical centrifuge (refrigerated) at 1000 g (about 2500–3000rpm) for 10 minutes to yield approximately 2 mL of plasma from each 4 mL whole blood sample.
- Plasma will be harvested by pipetting two approximately equal aliquots (“A” and “B”) into two separate pre-labeled screw-cap polypropylene tubes. The caps will be securely tightened and the samples will be frozen and stored upright immediately at -20 °C or below.

- The time between blood collection and freezing the plasma will not exceed 2 hours.
- Ship specimens according to the instructions provided. Aliquot A shipped to bioanalytical site. Aliquot B stored at clinical site until the end of of each cohort, and then transferred to PCPK/MET group at J&JPRD Raritan, NJ, USA for potential metabolite identification.
- Repeat or unscheduled samples may be taken if required for safety reasons.
- If a cannula is used for blood sample collection, a small amount of blood will be discarded each time a sample is taken via the cannula.
- Questions regarding handling the plasma pharmacokinetic specimens should be addressed to the contact person for the sponsor.

Preparation of Urine Pharmacokinetic Samples

- Collect total urine in the appropriate urine collection container for the time periods listed.
- Thoroughly mix the total urine collected during that time period.
- Measure and record the volume of the total urine output for that time period in the CRF or laboratory requisition form.
- Transfer two 10 mL aliquots (or as near to 10 mL as possible if the urine volume for the collection period is low) from each pooled collection, “A” and “B” samples, into two appropriately labeled urine storage tubes.
- Store the urine samples in an upright position, at –18°C or lower until transfer. Ship specimens according to the instructions provided.
- Ship specimens according to the instructions provided. Aliquots A shipped to J&J PRD Spring House, PA USA, aliquot B stored at clinical site until the end of study, and then transferred to PCPK/MET group in Raritan, NJ, USA for potential metabolite identification.

Questions regarding handling the urine pharmacokinetic samples should be addressed to the contact person for the sponsor.

Attachment 2:
Labeling Instructions for Pharmacokinetic Samples

STRUCTURE OF THE LABEL:
1. Each label has 2 identical parts. Please complete all of the requested information (current date, time of sample, etc.), on both label parts.
2. The backing of the label and the label itself are perforated.

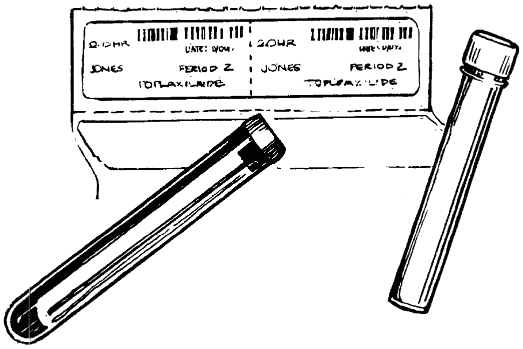

STEP 1: After the Vacutainer® has been filled, remove part 1 of the label and completely attach it to the Vacutainer®, lengthwise. Centrifuge the Vacutainer®.

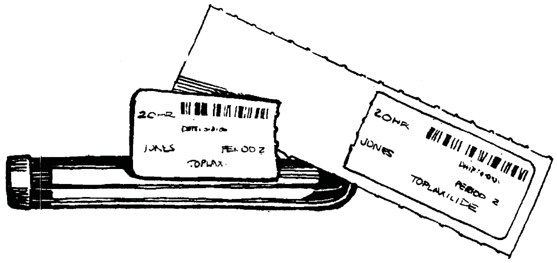

STEP 2: Remove part 2 of the label and attach it to the empty plasma collecting tube. The label must be attached to the tube lengthwise. You are now able to match the alphanumeric code, subject identification, and time point on the plasma collecting tube with the corresponding information on the Vacutainer®. After the tubes are matched, the plasma may be transferred from the Vacutainer® to the plasma collecting tube.

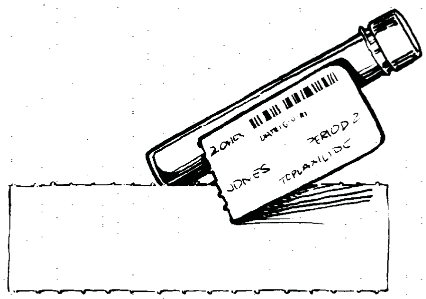

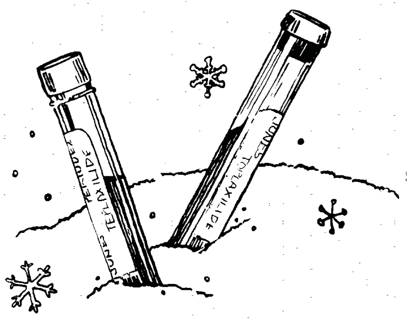

STEP 3: Freeze the sample in an upright position. Pack and ship the samples as instructed in the protocol.

Attachment 3:
Shipment of Pharmacokinetic Samples

All pharmacokinetic samples will be sent in multiple shipments as agreed upon with bioanalytical facility. An inventory list must be included with each shipment. The sponsor provides logs can be used as an inventory list. The inventory list must note each specimen drawn for each subject, and note any missing specimens.

The Study Investigator must follow the instructions below:

- For all shipments, Marken or World Courier will be used.
- The sponsor contact will be notified by FAX, that a shipment of samples is imminent. This notification will be made before the shipping date.
- Notify Johnson & Johnson Pharmaceutical Research and Development and World Courier at least 24 hours in advance of the planned shipment. Provide World Courier with the appropriate account number to be used, if applicable.
- Double-bag the frozen samples for each subject in bags that can withstand dry ice conditions (e.g. cryogenic bags), and label with Subject i.d. #.
- Pack samples in divided grid boxes and secured with tapes
- Pack the frozen samples in sufficient quantity of dry ice in appropriate containers, to maintain a frozen state for at least 3 days.
- Avoid direct contact between sample bags and dry ice by separating them with a dry ice resistant material (e.g., newspaper).
- For all biological samples, follow the IATA regulations for shipment.
- Ensure that the total package weight does not exceed 40 pounds.
- Label the package with the sponsor name and study number.
- Include a return address (which includes the investigator’s name) on the outside of each shipping container.
- Comply with all courier regulations for the shipment of biological specimens (include all paperwork).
- Retain all documents indicating date, time and signature/s of person/people making the shipment, in the study files.

Attachment 3 (continued)
Shipment of Pharmacokinetic Samples

As soon as shipment day and air bill number are available, the site will call and fax Ms. Heather Hoffman, J&JPRD (contact information provided below). The call or fax must specify the study number, number of packages shipped, the number of pharmacokinetic samples, and the time of shipment pick-up.

PK plasma and PK urine aliquots “A” must be sent to:

Heather Hoffman
J&J PRD
Welsh and McKean Roads
Spring House PA 19477-0776
Tel: 215-540-4926
Fax: 215-540-4603

PK plasma and urine aliquots “B” must be sent to:

Dr. H.K. Lim
Johnson & Johnson PRD
1001 North Rt. #202
OCD Bldg., K 007
Raritan, NJ 08869
Tel: 908-218-6355
Fax: 908-541-0422
Email: hlim5@prdus.jnj.com

NOTE: If there are changes regarding the courier or location to which samples are shipped during the course of the clinical study, written notification will be provided to the Investigator and will not require a protocol amendment.

Attachment 4:
Pharmacogenomic Sample Collection and Shipment Procedure

Pharmacogenomic Sample Supplies and Labeling

The Study Investigator will use appropriately labeled 10 ml blood collection tubes containing potassium or sodium EDTA. Specimen labels shall be pre-printed with the following information: i) the specimen type (e.g., "pharmacogenomic sample"), ii) the trial number, and iii) Case Report Form identification number (CRF i.d.). No other information will be written on the labels. The Pharmacogenomics Department does not have the logistics to provide labels.

Preparation of Pharmacogenomic Samples

Pharmacogenomic samples should be prepared as follows:

– Invert the tube 10 to 15 times immediately after collection, to prevent coagulation.
– DO NOT centrifuge the sample.
– Blood samples collected and shipped within 24 hours and can be shipped at ambient temperature (see sample shipment below).
– When there is a delay of more than 24 hours between collection and shipment, samples should be stored at 4ºC at the investigational site for at most 3 calendar days, and shipped in ambient or cooled condition (but not on dry ice). Samples are not to be frozen.

Pharmacogenomic Sample Shipment

– All pharmacogenomic samples will be sent to the Pharmacogenomics Department/Raritan, NJ.
– For international shipments, World Courier (telephone number: +32 2 712 5060) will be used. For domestic shipments, a reliable domestic courier, such as Federal Express, will be used.
– Notify courier, at least 24 hours in advance of the planned shipment. Provide the courier with the appropriate account number to be used, if applicable.
– On the day of shipment, fax the Sample Accountability Form (SAF) (available from the Pharmacogenomics Department) to the Pharmacogenomics Department in order to notify that a shipment of samples is imminent. The SAF must contain the courier name and tracking number.
– The SAF must be included in the shipping container.
– DO NOT package the samples in dry ice.
– Label the shipping container with all courier regulations for the shipment of biological specimens (include all paperwork).

- Include a return address (which includes the investigator's name) on the outside of each shipping container.
- Retain all documents indicating date, time and signature/s of person/people making the shipment, in the study files.
- Ship blood samples via courier, to the Pharmacogenomics Department

Ship pharmacogenomic samples to:

Dr. Stephan Francke
Johnson and Johnson Pharmaceutical Research & Development
Department of Pharmacogenomics
1000 Route 202
Raritan, NJ 08869
Phone 908 218 6596
FAX: 908 429 0695
sfranck1@prdus.jnj.com

NOTE: If there are changes regarding the courier or location to which samples are shipped during the course of the clinical study, written notification will be provided to the Investigator and will not require a protocol amendment.

Attachment 5:
Pharmacodynamic Sample Collection and Shipment Procedure

1. Materials and Labeling

Blood for pharmacodynamic assessments (2 mL for C-peptide, glucose and insulin) must be collected in the appropriate collection tubes. Resulting plasma samples must be stored in polypropylene storage tubes with polypropylene or polyethylene caps.

All tubes and containers will be labeled with preprinted labels. The preprinted information will include the study number, Case Report Form identification number (CRF i.d. #), treatment period, scheduled sampling day and time as stipulated in the flow chart, and the analyte name. No other information will be written on the labels.

- Pharmacodynamic samples for the assessment of glucose, insulin, and C-peptide must be labeled with a label from the Optical Mark (OMR) form.

Labels should be applied to the sample tubes as follows:

- Apply labels to the sample tubes so that they do not overlap and obscure any information. If possible, expose an area between the 2 ends of the label to allow viewing of the contents of the tube.
- Do not alter the orientation of the label on the sample tube.
- Apply labels to all tubes in the same manner.

2. Preparation of Plasma Pharmacodynamic Samples for Glucose, Insulin, and C-Peptide

- Collect 2 mL of blood into a 2.0-mL Vacutainer ® lithium heparin tube at each time point for the assessment of glucose, insulin, and C-peptide (see 3 a, b, and c.). If a cannula is used for blood sample collection, a small amount of blood will be discarded each time a sample is taken via the cannula.
- Record the exact date and time of sampling in the CRF.
- Gently invert the tubes 5 to 6 times to afford mixing, before processing.
- Centrifuge the tubes for 10 minutes at 1500 x g at Room temperature.
- Ship specimens to the laboratory according to Attachment 6. The samples will be analyzed on an ongoing base.
- Questions regarding handling the pharmacodynamic specimens should be addressed to the Sponsor contact person.

a. Determination of Glucose in human plasma samples by Hitachi Modular/Roche

Matrix: Plasma
Anticoagulant: Lithium heparin
Preservatives: None
Method: hexokinase/glucose-6-P-dh

Sample collection: To prepare plasma samples, whole blood is directly drawn into a 2.0 mL Vacutainer ® lithium heparin tube. Centrifuge at 1500 x g for 10 minutes at room temperature. Samples will be analyzed on an ongoing base.

b. Determination of Insulin in human plasma samples by Hitachi Modular/Roche

Matrix: Plasma
Anticoagulant: Lithium heparin
Preservatives: None
Method: ECLIA
Sample collection: To prepare plasma samples, whole blood is directly drawn into a 2.0 mL Vacutainer® lithium heparin tube. Centrifuge at 1500 x g for 10 minutes at room temperature. Samples will be analyzed on a ongoing base

c. Determination of C-peptide in human plasma samples by Hitachi Modular/Roche

Matrix: Plasma
Anticoagulant: Lithium heparin
Preservatives: None
Method: ECLIA
Sample collection: To prepare plasma samples, whole blood is directly drawn into a 2.0 mL Vacutainer ® lithium heparin tube. Centrifuge at 1500 x g for 10 minutes at room temperature. Samples will be analyzed on an ongoing base

3. Preparation of Plasma Pharmacodynamic Samples for GLP-1

- Collect 3 mL of blood into the appropriate collection tube at each time point for GLP- (see 3.a.) If a cannula is used for blood sample collection, a small amount of blood will be discarded each time a sample is taken via the cannula.
- Record the exact date and time of sampling in the CRF.
- Gently invert the tubes 5 to 6 times to afford mixing, before processing.
- Centrifuge the tubes for 10 minutes at 1500 x g at 4°C
- Ship specimens to the laboratory according to instructions below.
- Questions regarding handling the pharmacodynamic specimens should be addressed to the Sponsor contact person.

a. Determination of GLP-1 in human plasma samples by ELISA

Matrix: Plasma
Anticoagulant: EDTA
Preservatives: DPP IV inhibitor
Sample collection: To prepare plasma samples, 3 ml whole blood is directly drawn into an ice-cooled 3.0 ml Vacutainer ® P700 V1.0 K_2 EDTA plasma tube containing 5.4 mg DPP4 inhibitor (BD; Cat# 366473). Invert tube to mix and store tubes in ice bath. Centrifuge immediately at 1000 x g for 10 minutes in refrigerated centrifuge or place tubes on ice and centrifuge within one hour. Store at –70°C. Samples must be shipped on dry ice.

Attachment 6:
Shipment of Pharmacodynamic Samples

All Glucose, Insulin, and C-peptide pharmacodynamic samples, urine glucose and urine electrolyte pharmacodynamic samples and bone specific alkaline phosphatase, serum osteocalcin, urine deoxypyridinolines and urine NTX-1 will be sent to the Medizinische Laboratorien Marienhof, in multiple shipments as agreed upon with J&JPRD. An inventory list must be included with each shipment. The inventory list must note each specimen drawn for each subject, and note any missing specimens.

Have the samples double-bagged in trays that can withstand dry ice conditions, and label with Subject's CRF i.d. #

The study coordinator must notify the Laboratory of the planned shipment.

Samples must be sent to:

Dr. Rudi Reinards
Medizinische Laboratorien Marienhof
Wallstr. 10
41061 Mönchengladbach
Germany
Tel. 49 2161 8194 416/410
email: RReinards@mlm-marienhof.de

Labeling of GLP-1 samples –

Please follow labeling instructions provided in Attachment 2. Watson labels will be provided.

Shipment of GLP-1 samples –

Please follow shipping instructions provided in Attachment 3.

As soon as shipment day and air bill number are available, the site will call and fax Ms. Cianna Cooper, J&JPRD (contact information provided below). The call or fax must specify the study number, number of packages shipped, the number of GLP-1 samples, and the time of shipment pick-up.

GLP-1 plasma samples must be sent to:

Cianna Cooper
Johnson & Johnson PRD
1000 Route 202 S
OMP Research, B-364
Raritan, NJ 08869
Tel: 908-704-5852
Fax: 908-218-0973
Email: ccooper5@prdus.jnj.com

NOTE: If there are changes regarding the courier or location to which samples are shipped during the course of the clinical study, written notification will be provided to the Investigator and will not require a protocol amendment.

Attachment 7:
Grading and Treatment of Hypoglycemic Events

Definition

Hypoglycemia is defined as one the following:

Characteristic symptoms of hypoglycemia (e.g., chills, sweats, shakes, tachycardia, withdrawn attitude) with no blood glucose check. Clinical picture must include prompt resolution with food intake, subcutaneous glucagons, or intravenous glucose.

Characteristic symptoms of hypoglycemia with blood glucose check showing glucose 59 mg/dL (3.2 mM) or less. Symptoms associated with a blood glucose of 60 mg./dL (3.3 mM) or greater cannot be reported as hypoglycemia.

Any glucose measurement 49 mg/dL (2.7 mM) or less, with or without symptoms.

Severity:

Every hypoglycemic event must be characterized with respect to its severity. In order to characterize the event as severe, all of the following criteria must be met:

- The subject was unable to treat himself or herself.
- The subject exhibited at least one of the following neurological symptoms:

Memory loss
Confusion
Uncontrollable behavior
Irrational behavior
Unusual difficulty in awakening
Suspected seizure
Seizure
Loss of consciousness

- Also, one of the following:

If blood glucose was measured and was 49 mg/dL or less or,
If the blood glucose was not measured, the clinical manifestations were revered by oral carbohydrates, subcutaneous glucagons, or intravenous glucose
Events that do not meet all 3 criteria for sever hypoglycemia are characterized as mild to moderate.

Monitoring and treatments

All subjects will be closed monitored. A bedside glucose measurement using a Glucometer will be performed at scheduled time points and on an as needed basis when subjects develop symptoms suggestive of hypoglycemia. For any subject whose blood glucose level is < 50 mg/dL, bedside glucose will be measured every 15 to 30 minutes until the blood glucose concentration is > 50 mg/dL.

Orange juice should be given to a subject with glucose < 50 mg/dL and with symptoms of hypoglycemia. Orange juice will automatically be given to any subject with a glucometer reading < 40mg/dL

Attachment 8:

Standard Meals

Standardized total daily calories based on daily energy expenditure on Days -3 to 20 (in-patient setting):

- 2300 kcal

BMI-Adjustment

BMI 19 - <25	approx. 2100-2400 kcal
BMI 25 - <30	approx. 2401-2700 kcal
BMI 30 - <35	approx. 2701-2900 kcal
BMI > =35	approx. 2901-3200 kcal

The standard meals used for this study will be in accordance with the recommendations of the American Diabetes Association (American Diabetes Association: Evidence-based nutrition principles and recommendations for the treatment and prevention of diabetes and related complications,[21] which foresee 50-60% of calorie intake to come from carbohydrates, 15-20% from protein and 25-35% from fat.

− Breakfast (or dinner for BID dosing) within 10 minutes after dosing
Approximately 30% of total calorie intake
− Lunch at 4.5 hours after dosing
Approximately 35% of total calorie intake
− Dinner at 10.5 hours after dosing
Approximately 35% of total calorie intake

The three meals served on Days -1, 1 and 16 will be of the exact same composition on all days for all subjects.

Subjects should finish each meal completely within 30 minutes. Subjects should be encouraged to finish off all their standardized meals.

The menu of the standard meals will be prepared by a certified dietician at the study site according to the meal components as defined above and finalized with a joint agreement between the PI and the sponsor.

Attachment 9:
Medications with Potential for CYP3A4 Inhibition and/or Induction Properties

Medications with Potential for CYP3A4 Inhibition*

3A4,5,7
HIV Antivirals:
delaviridine
indinavir
nelfinavir
ritonavir
amiodarone
aprepitant
NOT azithromycin
chloramphenicol
cimetidine
ciprofloxacin
clarithromycin
diethyl-dithiocarbamate
diltiazem
erythromycin
fluconazole
fluvoxamine
gestodene
grapefruit juice
itraconazole
ketoconazole
mifepristone
nefazodone
norfloxacin
norfluoxetine
mibefradil
star fruit
verapamil

*Does not include azithromycin

Medications with Potential for CYP3A4 Induction

3A,4,5,7
HIV Antivirals:
efavirenz
nevirapine
barbiturates
carbamazepine
glucocorticoids
modafinil
phenobarbital
phenytoin
rifampin
St. John's wort
troglitazone
oxcarbazepine
pioglitazone
rifabutin

Attachment 10:
Concomitant Medications

Anti-Hypertension Drugs

For subjects with controlled hypertension enrolled in this study, monotherapy with an Angiotensin Converting Enzyme Inhibitor (ACEI), or an Angiotensin-II Receptor Antagonist (ATII Antagonist), or a Calcium-Channel Blocker (CCB).

Diuretics and Beta-blockers are not allowed.

The following combination drug regimens are also allowed: ATII Ant + CCB, ACEI + CCB. For all anti-hypertensive drug regimens, the dose and frequency must be stable and unchanged for at least 3 months preceding study start. Specific allowed agents are listed below. Other agents may be allowable if agreed between the Principal Investigator and the Sponsor during screening.

Allowed ACE Inhibitors

Benazepril, Captopril, Enalapril, Fosinopril, Lisinopril, Moexipril, Perindopril, Quinapril, Ramipril, Trandolapril

Allowed ATIIR Antagonists

Candisartan, Eprosartan, Telmisartan, Valsartan

Allowed CCBs

Amlodipine, Felodipine, Isradipine, Nicardipine, Nifedipine, Nisolodipine

Combined ACE Inhibitor / CCB Formulations

Lotrel (benazepril / amlodipine); Lexxel (enalapril / felodipine); Ta

Allowed Dyslipidemic Agents

For subjects with common dyslipidemias who have been on a stable regimen for at least 3 months prior to screening of a statin, or a fibrate, or a statin and a fibrate, the drugs listed below can be continued during the study. Subjects who have received bile-acid binding resins (e.g., cholestyramine, colestipol, colesevelam), or nicotinic acid within 3 months of the study may not be enrolled.

HMG-CoA Reductase Inhibitors ("Statins")

Atorvastatin (Lipitor), Lovastatin (Mevacor), Pravastatin (Pravachol), Rosuvastatin (Crestor), Simvastatin (Zocor)

Fibrates

Bezafibrate, Ciprofibrate, Clofibrate, Fenofibrate, Gemfibrozil

Cholesterol Transporter Inhibitors

Ezetimibe, Ezetimibe/Simvastatin combination (Vytorin)

OTHERS

Low dose acetylsalicylic acid (≤100 mg/day)

Attachment 11:
Visual Analog Scale Questionnaire for Appetite and Satiety

Instructions:

- Record the exact date and time in the CRF.
- Subjects should not be allowed to view scores from previous assessments.

How hungry do you feel?

I am not hungry at all	______________________	I have never been hungrier

How satisfied do you feel?

I am completely empty	______________________	I cannot eat another bite

How full do you feel?

Not at all full	______________________	Totally full

How much do you think you can eat?

Nothing at all	______________________	A lot

Attachment 12:
Patient Reported Outcomes Questionnaire

INSTRUCTIONS: Below is a list of symptoms. If you had the symptom during the last 24 hours, please check YES. If you did have the symptom, please check the box that tells us how much the symptom DISTRESSED or BOTHERED you.

**Check *all* the symptoms you have had during the last 24 hours**	**Yes (x)**	**→ → IF YES: How much did it DISTRESS or BOTHER you?**				
		**Not at all**	**A little Bit**	**Some-what**	**Quite a Bit**	**Very Much**
1. Frequent urination during the daytime hours						
2. An uncomfortable urge to urinate						
3. Waking up at night because you had to urinate						
4. Waking up at night for reasons other than need to urinate						

Note: The data collected will be analyzed and reported separately from the Clinical Study Report. The information reported on this questionnaire will not be considered to be adverse events.

LAST PAGE
