# Supplementary material for: Correction: Pharmacodynamic Effects of Canagliflozin, a Sodium Glucose Co-Transporter 2 Inhibitor, from a Randomized Study in Patients with Type 2 Diabetes
Source: PLoS One. 2014 Sep 30;9(9):e110069. doi: 10.1371/journal.pone.0110069 (PMC4182631; doi:10.1371/journal.pone.0110069)
Supplement: File S2 — Republished, corrected article PDF [file pone.0110069.s002.pdf]

OPEN ACCESS Freely available online

PLOS ONE

Pharmacodynamic Effects of Canagliflozin, a Sodium Glucose Co-Transporter 2 Inhibitor, from a Randomized Study in Patients with Type 2 Diabetes

Sue Sha[1]*, Damayanthi Devineni[1], Atalanta Ghosh[1], David Polidori[2], Marcus Hompesch[3], Sabine Arnolds[4], Linda Morrow[3], Heike Spitzer[4], Keith Demarest[1], Paul Rothenberg[1]

1 Janssen Research & Development, LLC, Raritan, NJ, United States of America, **2** Janssen Research & Development, LLC, San Diego, CA, United States of America, **3** Profil Institute for Clinical Research, Chula Vista, CA, United States of America, **4** Profil Institut für Stoffwechselforschung GmbH, Neuss, Germany

Abstract

Introduction: This randomized, double-blind, placebo-controlled, single and multiple ascending-dose study evaluated the pharmacodynamic effects and safety/tolerability of canagliflozin, a sodium glucose co-transporter 2 inhibitor, in patients with type 2 diabetes.

Methods: Patients (N = 116) discontinued their antihyperglycemic medications 2 weeks before randomization. Patients received canagliflozin 30, 100, 200, or 400 mg once daily or 300 mg twice daily, or placebo at 2 study centers in the United States and Germany, or canagliflozin 30 mg once daily or placebo at 1 study center in Korea, while maintaining an isocaloric diet for 2 weeks. On Days –1, 1, and 16, urinary glucose excretion (UGE), plasma glucose (PG), fasting PG (FPG), and insulin were measured. The renal threshold for glucose (RT_G) was calculated from UGE, PG, and estimated glomerular filtration rate. Safety was evaluated based on adverse event (AE) reports, vital signs, electrocardiograms, clinical laboratory tests, and physical examinations.

Results: Canagliflozin increased UGE dose-dependently (~80–120 g/day with canagliflozin ≥100 mg), with increases maintained over the 14-day dosing period with each dose. Canagliflozin dose-dependently decreased RT_G, with maximal reductions to ~4–5 mM (72–90 mg/dL). Canagliflozin also reduced FPG and 24-hour mean PG; glucose reductions were seen on Day 1 and maintained over 2 weeks. Plasma insulin reductions with canagliflozin were consistent with observed PG reductions. Canagliflozin also reduced body weight. AEs were transient, mild to moderate in intensity, and balanced across groups; 1 canagliflozin-treated female reported an episode of vaginal candidiasis. Canagliflozin did not cause hypoglycemia, consistent with the RT_G values remaining above the hypoglycemia threshold. At Day 16, there were no clinically meaningful changes in urine volume, urine electrolyte excretion, renal function, or routine laboratory test values.

Conclusions: Canagliflozin increased UGE and decreased RT_G, leading to reductions in PG, insulin, and body weight, and was generally well tolerated in patients with type 2 diabetes.

Trial Registration: ClinicalTrials.gov NCT00963768

Citation: Sha S, Devineni D, Ghosh A, Polidori D, Hompesch M, et al. (2014) Pharmacodynamic Effects of Canagliflozin, a Sodium Glucose Co-Transporter 2 Inhibitor, from a Randomized Study in Patients with Type 2 Diabetes. PLoS ONE 9(8): e105638. doi:10.1371/journal.pone.0105638

Editor: Noel Christopher Barengo, University of Tolima, Colombia

Received March 28, 2014; **Accepted** July 21, 2014; **Published** August 28, 2014

Copyright: © 2014 Sha et al. This is an open-access article distributed under the terms of the Creative Commons Attribution License, which permits unrestricted use, distribution, and reproduction in any medium, provided the original author and source are credited.

Data Availability: The authors confirm that all data underlying the findings are fully available without restriction. All relevant data are within the paper and its Supporting Information files.

Funding: This study was sponsored by Janssen Research & Development, LLC. Editorial support was provided by Cherie Koch, PhD, of MedErgy, and was funded by Janssen Global Services, LLC. Canagliflozin has been developed by Janssen Research & Development, LLC, in collaboration with Mitsubishi Tanabe Pharma Corporation. The funding organization was involved in the design, data collection and analysis, and preparation of the manuscript for publication. The funder also provided support in the form of salaries for authors Sue Sha, Damayanthi Devineni, Atalanta Ghosh, David Polidori, Keith Demarest and Paul Rothenberg.

Competing Interests: The authors have the following interests. This study was sponsored by Janssen Research & Development, LLC. Editorial support was provided by Cherie Koch, PhD, of MedErgy, and was funded by Janssen Global Services, LLC. Canagliflozin has been developed by Janssen Research & Development, LLC, in collaboration with Mitsubishi Tanabe Pharma Corporation. The funding organization was involved in the design, data collection and analysis, and preparation of the manuscript for publication. Sue Sha, Damayanthi Devineni, Atalanta Ghosh, David Polidori, Keith Demarest and Paul Rothenberg are employed by Janssen Research & Development. Marcus Hompesch and Linda Morrow are employed by Profil Institute for Clinical Research and Sabine Arnolds and Heike Spitzer by Profil Institut für Stoffwechselforschung GmbH. There are no further patents, products in development or marketed products to declare. This does not alter the authors' adherence to all the PLOS ONE policies on sharing data and materials, as detailed online in the guide for authors.

* Email: ssha@its.jnj.com

Introduction

The kidney plays an important role in glucose homeostasis, in large part through reabsorption of filtered glucose at the proximal tubule [1]. The majority of renal glucose reabsorption is mediated by the sodium glucose co-transporter 2 (SGLT2), which is a high-capacity, low-affinity glucose co-transporter expressed in the S1 segment of the proximal tubule [2,3]. SGLT1, a low-capacity,

high-affinity glucose co-transporter expressed in the S2 and S3 segments of the proximal tubule, is also involved in renal glucose reabsorption, but to a lesser extent than SGLT2 [2,3]. The SGLT1 and SGLT2 transporters are able to reabsorb virtually all filtered glucose until the filtered load exceeds the capacity of the transporters; the plasma glucose (PG) concentration at which this occurs is designated as the renal threshold for glucose (RT_G) [2,4,5]. Reducing renal glucose reabsorption via SGLT2 inhibition is a new approach to treating patients with type 2 diabetes [6].

Canagliflozin is an SGLT2 inhibitor approved in the United States, the European Union, and other countries for the treatment of adults with type 2 diabetes mellitus [7–16]. Canagliflozin lowers PG by lowering RT_G and reducing renal glucose reabsorption, leading to increased urinary glucose excretion (UGE) [17–19]. The increased UGE with SGLT2 inhibition is associated with a mild osmotic diuresis and a loss of calories leading to body weight reduction.

In Phase 3 studies, canagliflozin 100 and 300 mg improved glycemic control and reduced body weight, and were generally well tolerated in patients with type 2 diabetes on a variety of background antihyperglycemic therapies [7,8,10–12,15,16]. The purpose of the current study was to evaluate the pharmacodynamic effects and safety/tolerability of single and multiple ascending oral doses of canagliflozin in patients with type 2 diabetes mellitus. The effects of a range of doses of canagliflozin on UGE, RT_G, PG, insulin, and body weight were assessed.

Methods

Patients and Study Design

This randomized, double-blind, placebo-controlled, single and multiple ascending-dose, Phase 1 study was conducted at 3 study centers in the United States, Germany, and South Korea (ClinicalTrials.gov, NCT00963768; available at: http://www.clinicaltrials.gov/ct2/show/NCT00963768) from June 4, 2007 to December 27, 2007. The current study was registered after enrollment of patients had begun because the registration of Phase 1 trials was not required at the time the study began. The authors confirm that ongoing and related trials for this drug are registered, with the exception of some pilot studies. The protocol for this trial and supporting CONSORT checklist are available as supporting information; see Protocol S1 and Checklist S1. The study consisted of a washout period (approximately 2 weeks to discontinue from their previous antihyperglycemic agents [AHAs]), and a single-day treatment followed by a washout day and then 14 consecutive days of treatment.

Eligible patients were men and postmenopausal or surgically sterile women aged 25 to 65 years with a body mass index (BMI) of 20 to 40 kg/m^2 and a diagnosis of type 2 diabetes mellitus for $\geq$12 months before screening. Patients were on a stable regimen of oral AHA (excluding exenatide and thiazolidinediones) as monotherapy (German study center) or as monotherapy or dual therapy (US and Korean study centers) for $\geq$3 months prior to screening. Patients at the US and Korean study centers had A1C of 7.0% to 10.0% at screening and fasting plasma glucose (FPG) levels between 7.8 and 15.0 mM (140–270 mg/dL) on Day −2. Patients at the German study center had A1C of 7.0% to 8.5% at screening and FPG levels between 7.8 and 13.3 mM (140–240 mg/dL) on Day −2.

Exclusion criteria included a history of type 1, brittle diabetes or secondary forms of diabetes; repeated severe hypoglycemia episodes; diabetic complications including retinopathy, nephropathy, neuropathy, gastroparesis, or ketoacidosis; a history of, or currently active, clinically significant illness (eg, cardiovascular, hematologic, respiratory, hepatic, or gastrointestinal disease; endocrine or metabolic disorders; neurologic or psychiatric disease; or malignant neoplasms). For the German study center, patients with estimated glomerular filtration rate (eGFR) $<$ 70 mL/min/1.73 m^2 based on the Modification of Diet in Renal Disease equation [20] or macroalbuminuria $>$0.2 g/L were excluded; there were no eGFR exclusion criteria for the study centers in the United States or Korea.

Ethics statement

This study was conducted in accordance with the ethical principles that have their origin in the Declaration of Helsinki and are consistent with Good Clinical Practice and applicable regulatory requirements. Approval was obtained from institutional review boards and independent ethics committees for participating centers (ie, Ärztekammer Nordrhein Ethik-Kommission, Düsseldorf, Germany; COAST IRB, LLC, Lake Forest, CA, USA; Institutional Review Board of Severance Hospital, Seoul, South Korea). All patients provided informed, written consent prior to participation.

Randomization and Study Treatments

Following an initial screening, eligible patients discontinued their previous AHAs for 16 consecutive days prior to receiving the first dose of study medication on Day 1. At an outpatient visit on approximately Day −17, patients were counseled by a registered dietician to follow a standard diet in accordance with the

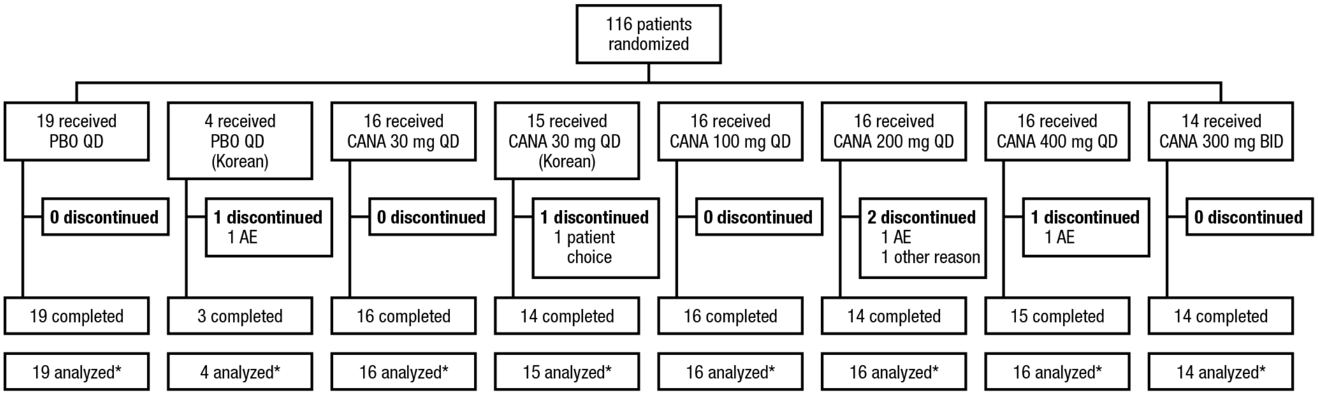

Figure 1. Study flow diagram. PBO, placebo; QD, once daily; CANA, canagliflozin; BID, twice daily. *Safety analysis set.
doi:10.1371/journal.pone.0105638.g001

Table 1. Baseline Demographic and Metabolic Characteristics.*

	PBO	PBO	CANA	CANA	CANA	CANA	CANA	CANA
		(Korean)	30 mg QD	30 mg QD (Korean)	100 mg QD	200 mg QD	400 mg QD	300 mg BID
	(n = 19)†	(n = 4)	(n = 16)	(n = 15)	(n = 16)	(n = 16)	(n = 16)	(n = 14)
Gender, n (%)								
Male	16 (84.2)	3 (75.0)	8 (50.0)	8 (53.3)	8 (50.0)	14 (87.5)	13 (81.3)	11 (78.6)
Female	3 (15.8)	1 (25.0)	8 (50.0)	7 (46.7)	8 (50.0)	2 (12.5)	3 (18.8)	3 (21.4)
Age, y	52.4±8.4	52.0±10.9	53.1±6.8	54.1±5.7	56.4±4.0	53.3±9.1	51.9±9.0	52.9±8.3
Race, n (%)‡								
White	10 (52.6)	0	5 (31.3)	0	16 (100)	11 (68.8)	6 (37.5)	9 (64.3)
Black	4 (21.1)	0	4 (25.0)	0	0	1 (6.3)	2 (12.5)	0
Asian	0	4 (100)	2 (12.5)	15 (100)	0	0	0	0
Hispanic	5 (26.3)	0	5 (31.3)	0	0	4 (25.0)	8 (50.0)	5 (35.7)
Weight, kg	94.2±21.0	75.5±20.1	88.9±17.8	66.2±11.4	87.8±14.6	92.2±11.0	92.7±15.2	94.8±23.2
BMI, kg/m^2	30.2±5.2	26.9±4.6	31.8±5.7	25.8±2.7	30.8±5.1	30.2±3.7	31.5±3.3	30.9±5.8
A1C, %	8.3±0.8	8.4±1.1	7.9±1.0	8.1±1.1	7.7±0.5	8.0±0.8	8.3±1.1	8.1±0.7
eGFR, mL/min/1.73 m^2	98.8±20.7	77.5±18.8	96.9±23.2	77.8±14.3	102.4±23.3	92.0±18.9	104.1±21.7	103.6±24.5
FPG, mM§	11.2±2.4	10.7±1.1	11.1±2.1	9.9±1.7	10.3±1.7	11.2±2.4	11.6±3.1	10.3±2.0
24-h mean PG, mM§	12.4±2.8	14.5±3.6	12.4±1.9	13.2±1.9	11.8±2.3	12.3±2.9	13.4±3.9	11.4±2.9

PBO, placebo; CANA, canagliflozin; QD, once daily; BID, twice daily; BMI, body mass index; eGFR, estimated glomerular filtration rate; FPG, fasting plasma glucose; PG, plasma glucose; SD, standard deviation.
*Data are mean ± SD unless otherwise indicated.
†Mean value for the PBO group was pooled from all dose groups.
‡Percentages may not total 100.0% due to rounding.
§Baseline values for FPG and 24-h mean PG are values on Day −1.
doi:10.1371/journal.pone.0105638.t001

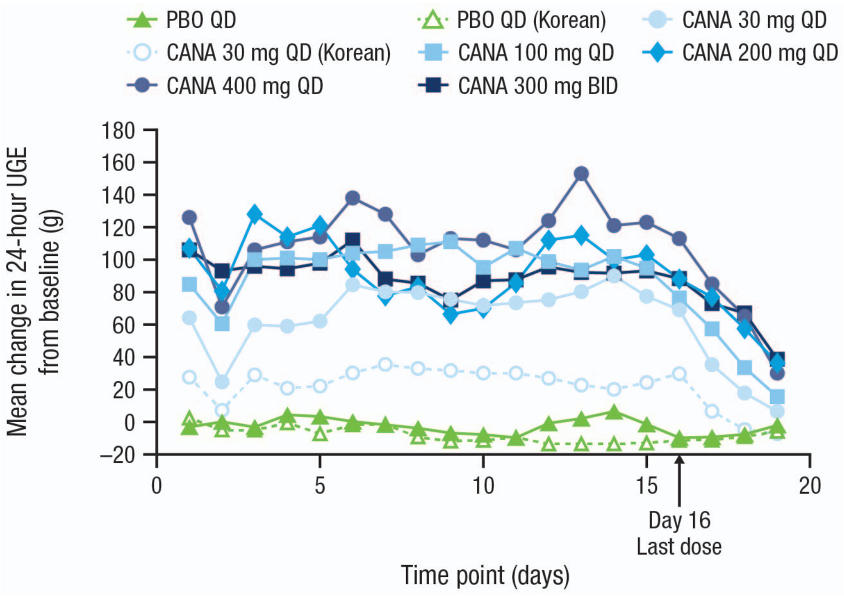

Figure 2. Change from baseline in 24-hour UGE. UGE, urinary glucose excretion; PBO, placebo; CANA, canagliflozin; QD, once daily; BID, twice daily.
doi:10.1371/journal.pone.0105638.g002

recommendation of the American Diabetes Association (ADA); blood glucose levels were monitored daily during the washout period. Patients who met enrollment criteria were assigned to cohorts according to randomization numbers assigned by the sponsor and randomized (4:1 within each cohort) to receive canagliflozin as liquid suspension (1 of 5 doses in an ascending, sequential order) or placebo. The unblinded pharmacist prepared the study drug for oral administration via an amber oral syringe covered with aluminum foil to maintain blinding.

Patients were admitted to the clinical research unit on Day –3 and were domiciled there through Day 20 (4 days after the final drug dose). Following an overnight fast, patients were given placebo in a single-blind fashion QD on Day −2 and Day −1, and underwent baseline safety and pharmacodynamic assessments on Day −1 (pretreatment baseline). Patients received double-blind treatment with canagliflozin (30, 100, 200, or 400 mg QD, or 300 mg twice daily [BID] in the United States and Germany and 30 mg QD in Korea) or placebo (at all 3 study centers) on Day 1 and on Days 3 to 16. The first dose was given between 8:00 and 9:00 AM with 240 mL of water. Within 10 minutes of dosing, patients received a standardized breakfast; standardized lunch and dinner were provided 4.5 and 10.5 hours postdose, respectively. The evening dose (for canagliflozin 300 mg BID) or matched placebo was administered between 6:00 and 7:00 PM. Standard meals in accordance with ADA recommendations consisted of 50% to 60% of calorie intake from carbohydrates, 15% to 20% from protein, and 25% to 35% from fat. Standardized total daily calories were based on estimated daily energy expenditure during the inpatient period in the clinical research unit (mean daily intake was approximately 2,300 kcal) and were adjusted based on BMI. For each patient, the 3 meals served on Days −1, 1, and 16 were of identical composition.

Patients were discharged from the study center on Day 20 and returned for safety assessments on the mornings of Days 21 and 22 and during a follow-up visit 7 to 10 days after the outpatient visit on Day 22.

Clinical Evaluations

Sample Collection and Bioanalyses. Blood samples for pharmacodynamic assessments (PG and insulin) were collected on Days −1, 1, and 16 at −0.5, −0.25, 0, 0.5, 1, 1.25, 1.5, 2, 2.5, 3, 4.5 (prior to lunch), 5, 5.5, 6, 6.5, 7, 8, 9, 10.5 (prior to dinner), 11, 12, 12.5, 13, 14, 16, 19, 22, and 24 hours. Morning FPG was obtained on Day 2 and within 30 minutes prior to dosing on Days 3 to 15 and prior to breakfast on Days 17 to 20.

Urine samples for the assessment of UGE were collected at intervals of 0 to 2, 2 to 4.5, 4.5 to 7, 7 to 10.5, 10.5 to 13, and 13 to 24 hours on Days −1, 1, and 16. On all other days, urine was collected over the interval of 0 to 24 hours.

Plasma glucose and insulin and urine glucose analyses were performed by MLM Medizinische Laboratorien, Marienhof, Germany. Plasma and urine glucose were analyzed by the hexokinase/glucose-6-phosphate dehydrogenase method using Gluco-quant Glucose/HK (Roche Diagnostics GmbH, Mannheim, Germany). Plasma insulin was analyzed by the electrochemiluminescence immunoassay (ECLIA) method (Roche Diagnostics Ltd., Rotkreuz, Switzerland).

As a result of inappropriate bioanalytical data manipulation by a single chemist, the pharmacokinetics data for canagliflozin from the current study were not considered reliable and are not included in this manuscript. Pharmacokinetics data with canagliflozin in patients with T2DM have been reported previously [21].

Pharmacodynamic Assessments

On Days 1 and 16, changes from baseline (Day −1) were determined for FPG and 24-hour mean PG. Changes from baseline in 24-hour cumulative UGE were assessed on Days 1, 2, 8, 12, 16, 17, 18, and 19.

RT_G on Days −1, 1, and 16 was calculated from measured PG, UGE, and eGFR as previously described [17–19,22]. RT_G was calculated over each of the time intervals where UGE was collected; the 24-hour mean RT_G and mean RT_G over the 0- to 13-hour and 13- to 24-hour intervals were calculated from the values obtained over each of the subintervals. Because patients not treated with canagliflozin often had only minimal amounts of

Table 2. Change in 24-hour UGE and 24-hour Mean RT_G.*

	PBO	PBO (Korean)	CANA 30 mg QD	CANA 30 mg QD (Korean)	CANA 100 mg QD	CANA 200 mg QD	CANA 400 mg QD	CANA 300 mg BID
	(n = 18)	(n = 4)	(n = 16)	(n = 15)	(n = 16)	(n = 16)	(n = 16)	(n = 13)
**UGE, g**								
24-hour UGE Day −1	24.5±31.9	17.4±11.6	10.9±11.9	17.5±14.5	11.5±11.5	22.2±32.9	43.7±67.9	12.5±16.4
LS mean change Day −1 to Day 1	−3.1	2.4	64.2	27.6	78.1	106.6	126.2	104.1
Difference vs PBO (95% CI)			67.3 (51.9, 82.7)†	25.3 (−0.3, 50.8)	81.2 (65.8, 96.6)†	109.7 (94.3, 125.1)†	129.3 (113.8, 144.6)†	107.2 (91.2, 123.1)†
LS mean change Day −1 to Day 16	−10.7	−11.3	67.2	29.7	76.6	88.0	113.1	85.3
Difference vs PBO (95% CI)			77.9 (58.7, 97.1) †	41.1 (5.1, 77.0)‡	87.3 (68.1, 106.5)†	98.7 (78.8, 118.6)†	123.8 (104.3, 143.3)†	96.0 (76.1, 115.9)†
**RT_G, mM**								
24-hour RT_G Day −1	13.2±1.7	14.9±2.6	14.4±1.5	14.1±1.0	13.0±1.6	13.5±1.6	14.0±1.8	13.3±1.8
24-hour RT_G Day 16	13.2±1.6	14.4±2.2	8.5±1.3	7.6±1.4	5.6±1.4	4.9±1.5	4.0±1.2	4.8±1.3
Change Day −1 to Day 16	−0.1±0.6	−1.2±0.6	−5.8±1.0†	−6.5±1.3†	−7.5±1.3†	−8.6±2.1†	−9.8±1.3†	−8.7±2.3†

UGE, urinary glucose excretion; RT_G, renal threshold for glucose; PBO, placebo; CANA, canagliflozin; QD, once daily; BID, twice daily; SD, standard deviation; LS, least squares; CI, confidence interval.
*Data are mean ± SD unless otherwise indicated.
$^{\dagger}P<0.0001$ versus PBO.
$^{\ddagger}P<0.05$ versus PBO.
doi:10.1371/journal.pone.0105638.t002

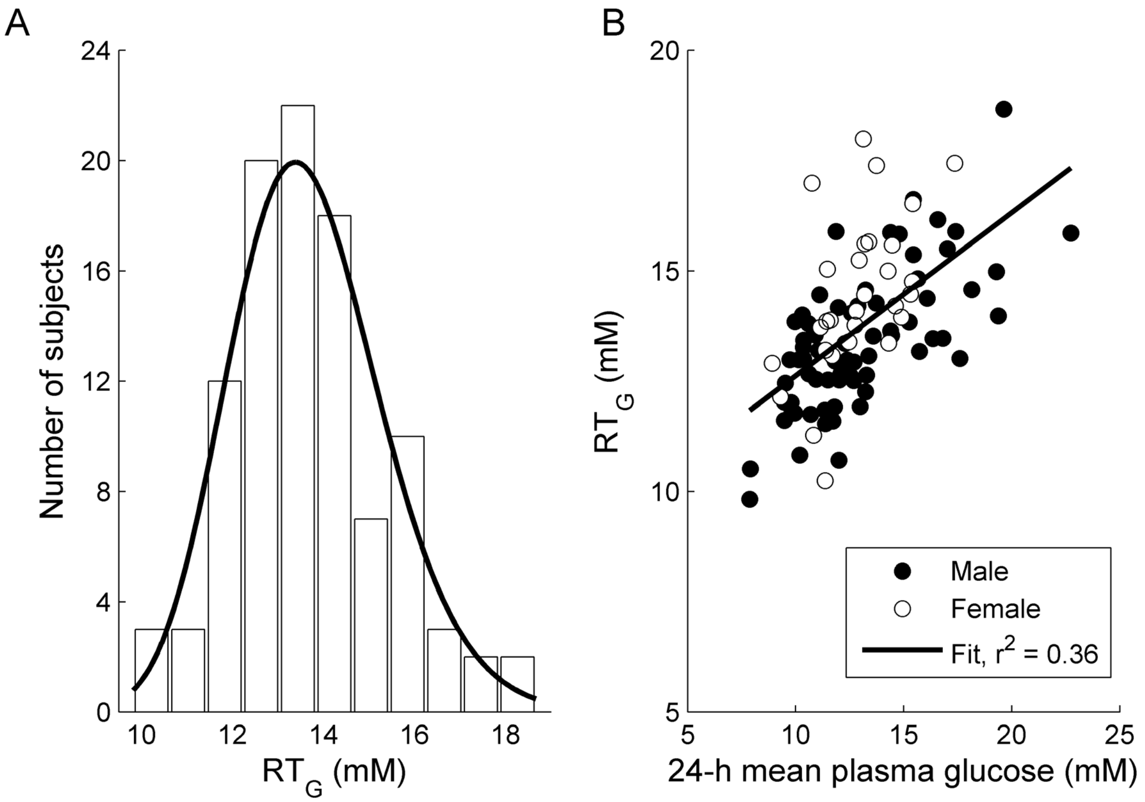

Figure 3. Relationship between baseline 24-hour mean RT_G and 24-hour mean PG. (A) Distribution of 24-hour mean RT_G values at baseline and (B) correlation between RT_G and 24-hour mean PG prior to canagliflozin treatment. RT_G, renal threshold for glucose; PG, plasma glucose.
doi:10.1371/journal.pone.0105638.g003

UGE during some of the individual collection intervals, RT_G values at the baseline visit and for placebo-treated patients were calculated using the full 24-hour urine collections and glucose profiles.

Safety Assessments

Safety was assessed from the time patients provided informed consent until the completion of the last study-related procedure at the final follow-up visit. Evaluations were based on the type, incidence, and severity of treatment-emergent adverse events (TEAEs) reported throughout the study, and on changes in vital sign measurements (ie, blood pressure and pulse rate), physical examinations, 12-lead electrocardiograms (ECGs), and clinical laboratory test results (ie, hematology, clinical chemistry, and urinalysis). With the exception of TEAEs, which patients reported

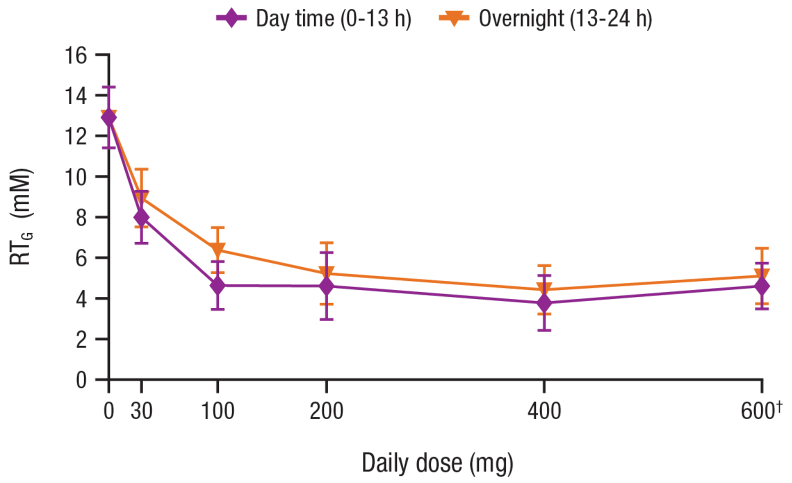

Figure 4. Change in RT_G by total daily dose of canagliflozin (Day 16). * RT_G, renal threshold for glucose; BID, twice daily. *Data are from Western patients only. †Canagliflozin 300 mg BID.
doi:10.1371/journal.pone.0105638.g004

Table 3. Change in PG Concentration.*

	PBO	PBO	CANA	CANA	CANA	CANA	CANA	CANA
		(Korean)	30 mg QD	30 mg QD (Korean)	100 mg QD	200 mg QD	400 mg QD	300 mg BID
FPG Day −1, mM	11.7±2.4	11.4±2.4	11.0±1.7	10.6±1.6	11.4±2.0	11.5±2.8	11.5±3.0	11.0±2.5
ΔFPG Day −1 to Day 16	−1.4±2.0	-3.4±1.4	−1.2±1.2	-2.9±1.3	−3.0±1.3†	−3.6±2.9‡	−3.3±2.7‡	−3.7±1.9‡
24-h mean PG Day −1, mM	12.4±2.8	14.5±3.6	12.4±1.9	13.2±1.9	11.8±2.3	12.3±2.9	13.4±3.9	11.4±2.9
Δ24-h mean PG Day −1 to Day 16	−1.0±1.4	-3.5±0.9	−1.2±1.0	-3.3±1.0	−2.6±1.4‡	−3.2±2.4§	−3.6±2.0§	−3.0±1.7§

PG, plasma glucose; PBO, placebo; CANA, canagliflozin; QD, once daily; BID, twice daily; FPG, fasting plasma glucose; SD, standard deviation.
*Data are mean ± SD.
†P<0.05 versus PBO.
‡P<0.01 versus PBO.
§P<0.001 versus PBO.
doi:10.1371/journal.pone.0105638.t003

voluntarily, standardized safety evaluations were conducted at designated times during the study.

Statistical Analyses

For sample size determination, it was estimated that 20 patients at each dose level (16 receiving canagliflozin and 4 receiving placebo) would be sufficient to detect a 15% reduction in mean 24-hour PG with 80% power, assuming a 1-sided *t*-test ($\alpha = 0.05$) and a coefficient of variation of 18%. Pharmacodynamic analyses were performed in all patients who received ≥1 dose of study drug and had ≥1 pharmacodynamic assessment. A mixed effect analysis of variance (ANOVA) model including dose and days of measurements as independent variables was used to analyze 24-hour mean PG and 24-hour UGE. The estimated least-squares (LS) means and 95% confidence intervals (CIs) for pair-wise comparisons of each canagliflozin dose to placebo were determined. All randomized patients were included for analyses in the safety population.

Results

Patient Disposition and Baseline Characteristics

A total of 116 patients were randomized to receive canagliflozin (n = 93) or placebo (n = 23). The flow of patients in the trial is provided in **Figure 1**. Baseline demographic and metabolic characteristics were generally comparable across cohorts (**Table 1**), with the exception of lower baseline body weight, BMI, and eGFR among patients in the Korean cohort.

Pharmacodynamic Effects

Urinary Glucose Excretion and RT_G. Canagliflozin treatment produced dose-dependent increases in UGE on both Day 1 and Day 16, compared with small decreases seen with placebo (**Table 2**). Increases in UGE from baseline were approximately 60 g/day in patients treated with the 30 mg dose and ranged from approximately 80 to 120 g/day in patients treated with doses ≥ 100 mg. The increase in UGE seen in the Korean patients who received canagliflozin 30 mg was approximately 30 g/day, which was smaller than the increase observed in the Western patients treated with the 30 mg dose. The effect of canagliflozin on 24-hour UGE observed on Day 1 was sustained over 2 weeks of treatment (**Figure 2**). UGE returned toward baseline after the last dose of canagliflozin.

The baseline values of 24-hour mean RT_G observed in this study in patients with type 2 diabetes mellitus ranged from 9.8 to 18.7 mM (177 to 336 mg/dL), with a mean (standard deviation) of 13.7 (1.7) mM (246 [30] mg/dL), and appeared to be log normally distributed (**Figure 3A**). The baseline RT_G values were generally higher in patients with higher baseline 24-hour mean PG levels (**Figure 3B**). Treatment with canagliflozin lowered RT_G in a dose-dependent manner (**Table 2**), with a maximal reduction of mean RT_G to approximately 4 to 5 mM (~72–90 mg/dL; **Figure 4**). For canagliflozin doses of ≥200 mg, near-maximal suppression of RT_G was sustained throughout the 24-hour dosing period. The 100 mg dose of canagliflozin provided near-maximal reduction in RT_G for the first 13 hours after dosing, with a modest waning of the effect in the overnight period (mean $RT_{G:13\text{–}24\ h}$ = 6.4 mM [124.5 mg/dL]) for the 100 mg dose on Day 16. The 30 mg dose of canagliflozin provided submaximal reductions in RT_G during both the daytime and overnight periods (**Figure 4**). Note that even though UGE was lower in the Korean patients compared with the Western patients treated with the 30 mg dose, a modestly greater suppression in mean RT_G was observed in the Korean patients compared with the Western patients (**Table 2**).

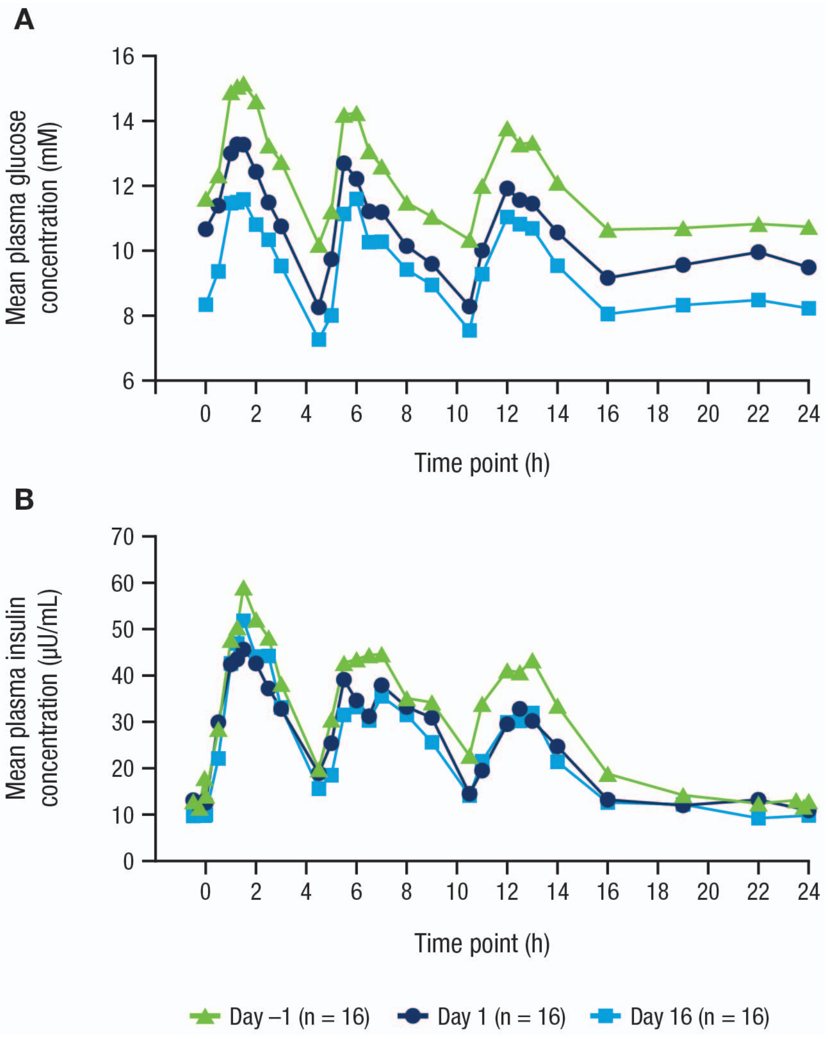

Figure 5. Mean (A) PG and (B) plasma insulin levels before (Day –1) and after a single dose (Day 1) and after multiple doses (Day 16) of canagliflozin 100 mg QD. PG, plasma glucose; QD, once daily.
doi:10.1371/journal.pone.0105638.g005

PG and Insulin. Canagliflozin treatment was associated with dose-dependent reductions in fasting and 24-hour mean PG. Canagliflozin doses ≥100 mg QD lowered fasting and postprandial PG levels and provided significantly greater reductions from baseline to Day 16 in mean fasting and 24-hour mean PG compared with placebo (**Table 3**). A representative 24-hour PG profile is shown in **Figure 5A** for canagliflozin 100 mg; similar results were seen with higher canagliflozin doses. Mean PG concentrations were reduced after the first dose of canagliflozin on Day 1, and the reductions in mean PG persisted over the entire 2-week treatment period following multiple dosing. Plasma insulin concentrations were also reduced with canagliflozin treatment (representative insulin profile for canagliflozin 100 mg shown in **Figure 5B**), consistent with the reduced PG concentrations.

Body Weight. A progressive, dose-dependent reduction in body weight from baseline (Day –1) to Day 16 was observed over the 2 weeks of treatment with canagliflozin (**Figure 6**). Mean body weight reductions were about 1 to 1.5 kg greater with canagliflozin doses ≥100 mg than with placebo after 2 weeks of treatment. Change in body weight with canagliflozin 30 mg was comparable to that observed with placebo.

Safety and Tolerability

Treatment with canagliflozin was generally well tolerated; among Western patients, 17 of 19 (89%) patients in the placebo group and 63 of 78 (81%) patients treated with canagliflozin reported TEAEs. Among Korean patients, 4 of 4 (100%) patients in the placebo group and 12 of 15 (80%) patients in the canagliflozin 30 mg group reported TEAEs. The majority of TEAEs were mild to moderate in intensity and were considered by the investigators to be not related, doubtfully related, or possibly related to study drug. There was no apparent correlation between the canagliflozin dose and the type, severity, duration, or incidence of TEAEs reported. There were no deaths reported. No serious TEAEs were reported in the Western cohort; 2 serious TEAEs were reported in the Korean cohort (1 AE of gallstone without cholecystitis in the placebo group, which led to study discontinuation, and 1 AE of diarrhea reported 9 days after the last study drug dose in the canagliflozin 30 mg group). Two patients in the Western cohort discontinued from the study due to TEAEs; a patient in the canagliflozin 200 mg group discontinued due to transient ventricular arrhythmia considered by the investigator to be doubtfully related to study drug, and a patient in the

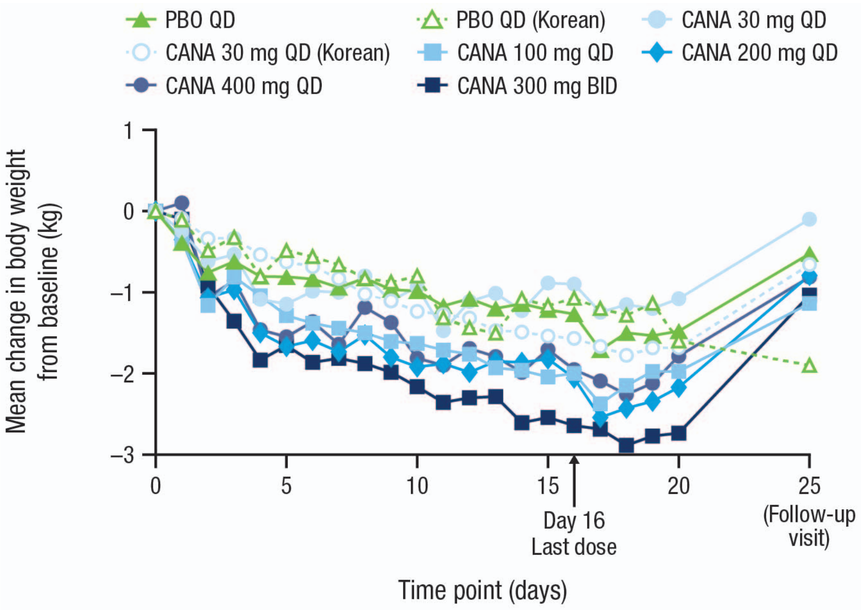

Figure 6. Mean change in body weight with daily canagliflozin treatment. PBO, placebo; CANA, canagliflozin; QD, once daily; BID, twice daily.
doi:10.1371/journal.pone.0105638.g006

canagliflozin 400 mg group discontinued due to persistent hyperglycemia.

The most frequently reported TEAEs were ECG electrode application site irritation and skin-related AEs (eg, erythema, lichenification, pruritus, psoriasis, and skin irritation). The skin-related TEAEs were not dose-related and generally resolved from within a few hours to a few days. There was 1 TEAE of vaginal candidiasis reported by a female patient in the canagliflozin 30 mg group of the Western cohort; this AE responded to butoconazole treatment and resolved in approximately 1 week. No hypoglycemia was reported during the study treatment period.

At baseline (Day –1), mean 24-hour urine volume was, on average, approximately 3 to 4 L in all treatment groups in the Western cohort; mean 24-hour urine volume was slightly lower among Korean patients (~2.7–3 L). Transient increases in 24-hour urine volume occurred with canagliflozin doses ≥200 mg on Day 1, ranging from approximately 200 to 700 mL; these increases declined toward baseline levels over the 2-week dosing period (**Figure 7**). Mean systolic blood pressure in the Western cohort decreased by 5 to 11 mmHg from baseline after 2 weeks of treatment with canagliflozin, compared with a 3 mmHg decrease in the placebo group (**Table 4**). In the Korean cohort, mean systolic blood pressure decreased by 2.5 mmHg with canagliflozin 30 mg compared with an increase of 1.0 mmHg with placebo. There were no observed increases in pulse rate and no incidences of orthostatic hypotension across cohorts. There were no clinically meaningful changes in mean 24-hour urinary albumin excretion, 24-hour creatinine clearance, or 24-hour urinary excretion of electrolytes (sodium, chloride, potassium, calcium, magnesium, or phosphate; **Table 4**). At Day 16, canagliflozin doses ≥100 mg were associated with a 20% mean reduction from baseline in serum uric acid. Overall, there was no apparent relationship between the frequency of any laboratory abnormality and the dose of canagliflozin in the study.

Discussion

In this study of patients with type 2 diabetes mellitus, canagliflozin doses from 30 to 400 mg QD increased UGE and lowered RT_G, FPG, and 24-hour mean PG levels. Increases in 24-hour total UGE were dose-dependent following single-dose administration of canagliflozin and were maintained after multiple dosing over the 14-day treatment period, with mean increases of approximately 80 to 120 g/day with canagliflozin doses ≥100 mg. An apparent saturation of the 24-hour UGE response was observed with canagliflozin doses >200 mg/day. Changes in 24-hour mean RT_G from baseline following single- and multiple-dose administration were also dose-dependent, with maximal reduction to approximately 4 to 5 mM (72–90 mg/dL), suggesting a low risk for treatment-induced hypoglycemia in patients treated with canagliflozin. Canagliflozin acted rapidly to decrease RT_G and increase UGE, with similar effects observed on Day 1 and Day 16. The daily UGE was ~50% lower with canagliflozin 30 mg in the Korean cohort versus the Western cohort. This difference appears to be explained by the lower GFR and plasma glucose concentrations (and hence, lower filtered glucose load) in the Korean patients compared with the Western patients rather than being due to reduced pharmacodynamic activity of canagliflozin in the Korean patients. This is supported by noting that the mean RT_G values, which provide a measure of renal glucose reabsorptive capacity that accounts for differences in GFR and plasma glucose concentrations, were suppressed by 6.5 mM in the Korean patients, which is numerically greater than the 5.8 mM suppression observed in the Western patients treated with canagliflozin 30 mg. Statistically and clinically significant dose-dependent reductions from baseline in 24-hour mean PG were observed with canagliflozin doses ≥100 mg compared with placebo. PG concentrations were also rapidly reduced with canagliflozin treatment, with reductions in PG observed within hours of treatment on Day 1 and further reductions in PG observed on Day 16 compared with Day 1. Plasma insulin concentrations were also reduced after canagliflozin treatment.

Table 4. Summary of Changes in Safety Parameters.

	PBO	PBO (Korean)	CANA 30 mg QD	CANA 30 mg QD (Korean)	CANA 100 mg QD	CANA 200 mg QD	CANA 400 mg QD	CANA 300 mg BID
	(n = 19)	(n = 4)	(n = 16)	(n = 15)	(n = 16)	(n = 16)	(n = 16)	(n = 13)
**Vital signs**								
Supine SBP Day −1, mmHg*	129.8±11.9	127.3±3.3	125.6±17.7	118.9±10.8	130.8±10.4	120.3±5.1	122.2±14.7	125.3±14.9
Change Day −1 to Day 16*	–3.1±9.6	1.0±9.9	−10.9±15.5	–2.5±6.8	–4.7±7.3	−11.5±7.3	–9.4±7.2	–9.8±7.6
Supine DBP Day −1, mmHg*	79.9±8.3	78.1±2.6	74.6±7.8	73.9±6.1	78.7±7.1	72.7±5.3	71.7±6.5	74.4±6.8
Change Day −1 to Day 16*	–0.4±6.3	1.2±9.7	–3.9±6.8	0.1±5.2	0.2±6.6	–4.5±6.1	–3.4±5.1	–2.9±4.5
Supine pulse rate Day −1, bpm*	70.9±7.3	69.3±24.1	71.5±13.7	72.3±7.3	73.2±7.9	68.0±7.4	71.2±6.0	68.1±7.0
Change Day −1 to Day 16*	–3.1±9.2	–7.3±2.2	–7.1±10.4	–9.2±9.3	–9.7±5.6	–5.1±6.1	–4.9±6.0	–5.5±3.9
**24-hour cumulative urine electrolytes, albumin excretion, and creatinine clearance**								
Calcium Day –1, mmol*	4.3±2.8	3.5±1.1	3.3±1.7	4.2±2.3	4.3±2.4	3.8±1.9	4.1±1.7	3.3±1.9
% change Day −1 to Day 16[†]	8.9±14.9	–39.8±8.2	–2.0±8.9	–5.5±9.1	–7.8±6.3	1.9±14.1	2.3±6.9	–11.7±10.5
Chloride Day –1, mmol*	202.5±56.4	105.2±48.7	227.4±79.8	115.7±44.5	238.1±59.3	216.3±61.4	204.3±48.0	227.6±44.6
% change Day −1 to Day 16[†]	–11.3±6.3	–9.9±15.6	–17.5±5.9	20.3±12.8	–7.4±5.7	–9.6±5.2	35.8±10.5	–19.5±8.2
Magnesium Day –1, mmol*	5.6±1.8	3.7±0.9	4.2±1.2	3.4±1.0	5.3±1.7	5.0±1.3	5.9±2.2	4.5±1.2
% change Day −1 to Day 16[†]	–6.9±6.6	–13.7±12.3	21.2±14.1	11.3±7.8	6.2±5.4	13.1±5.5	16.0±7.0	3.7±8.8
Phosphate Day –1, mmol*	32.6±7.7	16.2±6.8	31.0±7.9	13.9±4.7	32.8±11.5	31.2±7.2	34.3±5.8	30.2±7.6
% change Day −1 to Day 16[†]	–11.7±6.3	–17.1±14.4	–0.5±6.4	21.0±9.4	2.5±7.0	7.1±6.6	9.6±3.7	–3.1±6.9
Potassium Day –1, mmol*	68.7±23.5	43.8±18.4	69.0±14.9	40.5±11.1	74.0±20.2	68.7±14.2	73.9±19.7	68.0±19.0
% change Day −1 to Day 16[†]	–4.5±7.2	14.8±23.7	–9.0±6.0	31.9±9.6	–10.1±5.3	12.5±8.9	18.7±5.2	–3.0±8.5
Sodium Day –1, mmol*	218.2±56.4	111.5±33.6	234.0±69.5	118.8±45.9	266.2±60.6	225.0±51.4	231.3±34.7	233.0±50.6
% change Day −1 to Day 16[†]	–10.6±5.7	–12.0±5.4	–16.5±6.2	14.1±9.8	–15.0±4.9	–0.1±6.0	26.3±8.2	–22.9±6.8
Albumin Day –1, mg*	31.4±43.7	61.5±84.1	93.8±305.3	13.9±9.9	51.3±131.0	10.9±4.5	69.6±121.4	16.3±14.3
% change Day −1 to Day 16[†]	–2.0±11.4	–46.2±22.4	23.0±40.1	–2.1±9.7	34.1±45.1	–1.1±11.0	–9.3±8.1	–2.9±7.6
Creatinine clearance Day –1, mL/min/1.73 m^2*	118.6±30.8	59.7±24.9	105.1±24.7	51.8±14.2	135.4±55.8	106.9±23.8	122.4±29.1	104.6±24.1
% change Day −1 to Day 16[†]	–12.9±7.9	–35.0±6.2	–10.7±4.7	47.4±13.0	–14.7±4.7	–7.4±5.2	–9.3±4.7	–14.7±9.0

PBO, placebo; CANA, canagliflozin; QD, once daily; BID, twice daily; SBP, systolic blood pressure; DBP, diastolic blood pressure; bpm, beats per minute; SD, standard deviation; SE, standard error.
*Data are mean ± SD.
[†]Data are mean ± SE.
doi:10.1371/journal.pone.0105638.t004

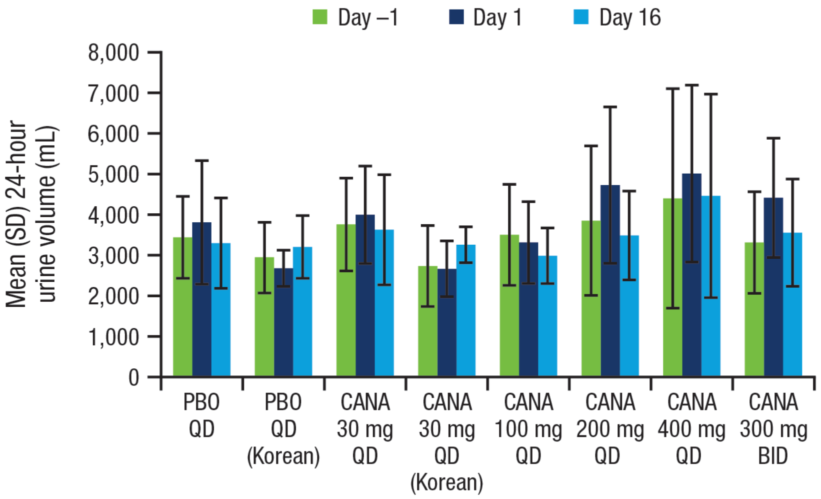

Figure 7. Mean 24-hour urine volume at baseline (Day –1) and after a single dose (Day 1) and after multiple doses (Day 16). SD, standard deviation; PBO, placebo; CANA, canagliflozin; QD, once daily; BID, twice daily.
doi:10.1371/journal.pone.0105638.g007

In patients with type 2 diabetes mellitus, mean baseline 24-hour RT_G was 13.6 mM (248 mg/dL), which is higher than the commonly cited range of 10 to 11 mM (180–200 mg/dL) for nondiabetic individuals [23,24]. The high baseline values of RT_G in patients in this study are similar to those reported in previous studies in patients with type 2 diabetes [17,21] and are consistent with reports of increased renal glucose reabsorption in patients with diabetes relative to nondiabetic individuals [25–27]. Increased expression of glucose transporters in the renal proximal tubules has been reported in human studies and animal models of diabetes [28–30], and this may contribute to the increase in RT_G associated with diabetes. An increase in the renal glucose resorptive capacity in patients with diabetes may contribute to sustained hyperglycemia in these patients [31].

In the current study, canagliflozin doses ≥100 mg were associated with body weight reductions that were about 1 to 1.5 kg greater than those observed with placebo. Both decreases in fluid volume and caloric loss via UGE are likely to contribute to the weight loss observed with canagliflozin over this time period. In longer-term Phase 3 studies of canagliflozin, progressive weight loss was observed through approximately 26 weeks, followed by a plateau with stable body weight reduction of approximately 2% to 4% [7,8,10–13,15]. Canagliflozin treatment over the 2-week treatment period was also associated with reductions in blood pressure. The blood pressure-lowering observed with canagliflozin in this study is consistent with findings from Phase 3 studies of longer duration [7,8,10–13,15,16] and may be related, at least in part, to an osmotic diuresis associated with canagliflozin.

Canagliflozin was generally well tolerated, with no clinically notable imbalances among treatments in the incidence or type of AEs reported or clinically relevant adverse changes in laboratory or ECG safety parameters. One female patient treated with canagliflozin reported a TEAE of vaginal candidiasis that resolved with standard topical therapy. This is consistent with findings from the canagliflozin Phase 3 development program showing an increase in incidence of genital mycotic infections with canagliflozin relative to placebo [32]. No hypoglycemia episodes were reported in this study, consistent with the observation that the maximum mean reduction in 24-hour RT_G with canagliflozin treatment was to approximately 5 mM (90 mg/dL), which is above the typical threshold for hypoglycemia (3.9 mM [70 mg/dL]). There were no persistent changes from baseline in daily 24-hour total urine volumes and urinary excretion of measured electrolytes over the 2 weeks of treatment, despite significantly elevated 24-hour UGE. Clinically significant adverse effects on measures of renal function (ie, urinary albumin excretion and creatinine clearance) were not observed.

A limitation of this study is that pharmacokinetic data for canagliflozin are not available for presentation in conjunction with the pharmacodynamic and safety assessments.

In summary, results of this study showed that treatment with canagliflozin at doses ≥100 mg QD for 2 weeks was associated with increased UGE and a decreased RT_G. Treatment with canagliflozin ≥100 mg QD resulted in clinically meaningful and statistically significant reductions from baseline in mean FPG and 24-hour mean PG in patients with type 2 diabetes. These findings likely account for the improvements in glycemic control and reductions in body weight observed with canagliflozin treatment in longer-term Phase 3 clinical studies [7,8,10–13,15,16].

Supporting Information

Checklist S1.
(DOC)

Protocol S1.
(PDF)

Acknowledgments

The authors thank all investigators, study teams, and patients for participating in this study. The authors acknowledge Stephan Voswinkel, of MLM Medical Labs, for his contributions to the safety and pharmacodynamics laboratory analyses. This study was previously presented, in part, in abstract form at the 70th Scientific Sessions of the American Diabetes Association, Orlando, Florida, June 25–29, 2010; and at the 46th Annual Meeting of the European Association for the Study of Diabetes, Stockholm, Sweden, September 20–24, 2010.

Author Contributions

Conceived and designed the experiments: SS DD AG MH SA KD PR. Performed the experiments: SS MH SA LM HS. Analyzed the data: DD AG DP. Contributed to the writing of the manuscript: SS DP PR. Other: SS DD AG DP MH SA LM HS KD PR.

References

1. Wright EM, Hirayama BA, Loo DF (2007) Active sugar transport in health and disease. J Intern Med 261: 32–43.
2. Gerich JE (2010) Role of the kidney in normal glucose homeostasis and in the hyperglycaemia of diabetes mellitus: therapeutic implications. Diabet Med 27: 136–142.
3. Wright EM (2001) Renal Na(+)-glucose cotransporters. Am J Physiol Renal Physiol 280: F10–F18.
4. Abdul-Ghani MA, Norton L, Defronzo RA (2011) Role of sodium-glucose cotransporter 2 (SGLT 2) inhibitors in the treatment of type 2 diabetes. Endocr Rev 32: 515–531.
5. Bays H (2009) From victim to ally: the kidney as an emerging target for the treatment of diabetes mellitus. Curr Med Res Opin 25: 671–681.
6. Chao EC, Henry RR (2010) SGLT2 inhibition–a novel strategy for diabetes treatment. Nat Rev Drug Discov 9: 551–559.
7. Bode B, Stenlöf K, Sullivan D, Fung A, Usiskin K (2013) Efficacy and safety of canagliflozin treatment in older subjects with type 2 diabetes mellitus: a randomized trial. Hosp Pract 41: 72–84.
8. Cefalu WT, Leiter LA, Yoon K-H, Arias P, Niskanen L, et al. (2013) Efficacy and safety of canagliflozin versus glimepiride in patients with type 2 diabetes inadequately controlled with metformin (CANTATA-SU): 52 week results from a randomised, double-blind, phase 3 non-inferiority trial. Lancet 382: 941–950.
9. Rosenstock J, Aggarwal N, Polidori D, Zhao Y, Arbit D, et al. (2012) Dose-ranging effects of canagliflozin, a sodium-glucose cotransporter 2 inhibitor, as add-on to metformin in subjects with type 2 diabetes. Diabetes Care 35: 1232–1238.
10. Schernthaner G, Gross JL, Rosenstock J, Guarisco M, Fu M, et al. (2013) Canagliflozin compared with sitagliptin for patients with type 2 diabetes who do not have adequate glycemic control with metformin plus sulfonylurea: a 52-week, randomized trial. Diabetes Care 36: 2508–2515.
11. Stenlöf K, Cefalu WT, Kim K-A, Alba M, Usiskin K, et al. (2013) Efficacy and safety of canagliflozin monotherapy in subjects with type 2 diabetes mellitus inadequately controlled with diet and exercise. Diabetes Obes Metab 15: 372–382.
12. Yale JF, Bakris G, Cariou B, Yue D, David-Neto E, et al. (2013) Efficacy and safety of canagliflozin in subjects with type 2 diabetes and chronic kidney disease. Diabetes Obes Metab 15: 463–473.
13. Lavalle-González FJ, Januszewicz A, Davidson J, Tong C, Qiu R, et al. (2013) Efficacy and safety of canagliflozin compared with placebo and sitagliptin in patients with type 2 diabetes on background metformin monotherapy: a randomised trial. Diabetologia 56: 2582–2592.
14. INVOKANA (2013) (canagliflozin) tablets, for oral use [package insert]. Titusville, NJ: Janssen Pharmaceuticals.
15. Wilding JP, Charpentier G, Hollander P, González-Gálvez G, Mathieu C, et al. (2013) Efficacy and safety of canagliflozin in patients with type 2 diabetes mellitus inadequately controlled with metformin and sulphonylurea: a randomised trial. Int J Clin Pract 67: 1267–1282.
16. Forst T, Guthrie R, Goldenberg R, Yee J, Vijapurkar U, et al. (2014) Efficacy and safety of canagliflozin over 52 weeks in patients with type 2 diabetes on background metformin and pioglitazone. Diabetes Obes Metab 16: 467–477.
17. Devineni D, Morrow L, Hompesch M, Skee D, Vandebosch A, et al. (2012) Canagliflozin improves glycemic control over 28 days in subjects with type 2 diabetes not optimally controlled on insulin. Diabetes Obes Metab 14: 539–545.
18. Polidori D, Sha S, Ghosh A, Plum-Mörschel L, Heise T, et al. (2013) Validation of a novel method for determining the renal threshold for glucose excretion in untreated and canagliflozin-treated subjects with type 2 diabetes mellitus. J Clin Endocrinol Metab 98: E867–E871.
19. Sha S, Devineni D, Ghosh A, Polidori D, Chien S, et al. (2011) Canagliflozin, a novel inhibitor of sodium glucose co-transporter 2, dose dependently reduces calculated renal threshold for glucose excretion and increases urinary glucose excretion in healthy subjects. Diabetes Obes Metab 13: 669–672.
20. Levey AS, Bosch JP, Lewis JB, Greene T, Rogers N, et al. (1999) A more accurate method to estimate glomerular filtration rate from serum creatinine: a new prediction equation. Modification of Diet in Renal Disease Study Group. Ann Intern Med 130: 461–470.
21. Devineni D, Curtin CR, Polidori D, Gutierrez MJ, Murphy J, et al. (2013) Pharmacokinetics and pharmacodynamics of canagliflozin, a sodium glucose co-transporter 2 inhibitor, in subjects with type 2 diabetes mellitus. J Clin Pharmacol 53: 601–610.
22. Magni L, Raimondo DM, Bossi L, Man CD, De Nicolao G, et al. (2007) Model predictive control of type 1 diabetes: an in silico trial. J Diabetes Sci Technol 1: 804–812.
23. Ganong WF (2005) Formation and excretion of urine. Renal function and micturition. In: Foltin J, Lebowitz H, Brown RY, editors. Review of Medical Physiology. 22 ed. New York, NY: Lange Medical Books/McGraw-Hill. 699–728.
24. Rave K, Nosek L, Posner J, Heise T, Roggen K, et al. (2006) Renal glucose excretion as a function of blood glucose concentration in subjects with type 2 diabetes–results of a hyperglycaemic glucose clamp study. Nephrol Dial Transplant 21: 2166–2171.
25. Mogensen CE (1971) Maximum tubular reabsorption capacity for glucose and renal hemodynamics during rapid hypertonic glucose infusion in normal and diabetic subjects. Scand J Clin Lab Invest 28: 101–109.
26. Farber SJ, Berger EY, Earle DP (1951) Effect of diabetes and insulin of the maximum capacity of the renal tubules to reabsorb glucose. J Clin Invest 30: 125–129.
27. Defronzo RA, Hompesch M, Kasichayanula S, Liu X, Hong Y, et al. (2013) Characterization of renal glucose reabsorption in response to dapagliflozin in healthy subjects and subjects with type 2 diabetes. Diabetes Care 36: 3169–3176.
28. Freitas HS, Anhe GF, Melo KF, Okamoto MM, Oliveira-Souza M, et al. (2008) Na(+) -glucose transporter-2 messenger ribonucleic acid expression in kidney of diabetic rats correlates with glycemic levels: involvement of hepatocyte nuclear factor-1alpha expression and activity. Endocrinology 149: 717–724.
29. Rahmoune H, Thompson PW, Ward JM, Smith CD, Hong G, et al. (2005) Glucose transporters in human renal proximal tubular cells isolated from the urine of patients with non-insulin-dependent diabetes. Diabetes 54: 3427–3434.
30. Vestri S, Okamoto MM, de Freitas HS, parecida Dos SR, Nunes MT, et al. (2001) Changes in sodium or glucose filtration rate modulate expression of glucose transporters in renal proximal tubular cells of rat. J Membr Biol 182: 105–112.
31. Defronzo RA (2009) Banting Lecture. From the triumvirate to the ominous octet: a new paradigm for the treatment of type 2 diabetes mellitus. Diabetes 58: 773–795.
32. Nyirjesy P, Sobel JD, Fung A, Mayer C, Capuano G, et al. (2014) Genital mycotic infections with canagliflozin, a sodium glucose co-transporter 2 inhibitor, in patients with type 2 diabetes mellitus: a pooled analysis of clinical studies. Curr Med Res Opin 30: 1109–1119.
